# Design of Novel Iminocoumarins for D‑π‑A System DSSCs: A (TD)DFT Study

**DOI:** 10.1021/acsomega.5c09063

**Published:** 2026-01-28

**Authors:** Patrick L. L. Rocha, Patrick de L. Barbosa, Amanda de A. Borges, Edson Evangelista, Isabela S. de Almeida, Luana da S. M. Forezi, Rodolfo G. Fiorot

**Affiliations:** Department of Organic Chemistry, Institute of Chemistry, 28110 Universidade Federal Fluminense − UFF, Niteroi, Rio de Janeiro 24020-141, Brazil

## Abstract

Dye-sensitized solar cells (DSSCs) are third-generation photovoltaic devices in which photogenerated electrons are injected from an excited dye into a mesoporous TiO_2_ film. The molecular design of the dye therefore plays a central role in tuning the light absorption and charge-transfer efficiency. In this work, we investigate the photovoltaic potential of a new series of 3-benzo­[d]­[1,3]­thiazole-substituted iminocoumarin dyes by comparing their properties with the benchmark coumarin dye NKX-2677. Using DFT and TD-DFT calculations, we evaluated Boltzmann-weighted conformer distributions, absorption/emission profiles, π-electron delocalization, orbital overlap, and the feasibility of electron injection and dye regeneration. The incorporation of 2,2′-bithiophene and benzo­[d]­[1,2,5]­diathiazole π-linkers was found to be essential for narrowing the HOMO–LUMO gap and enhancing molecular planarity. Among the designed sensitizers, A3, C3, and D3 display the most favorable spectral properties, with tunable absorption profiles modulated by substitution at the 7-coumarin position. Their light-harvesting efficiencies (LHE), reported here as a spectral proxy derived from the oscillator strength (*f*), highlight their potential as DSSC photosensitizers. Specifically, A3 shows λ_max_ = 427 nm, *f* = 0.971, LHE = 89.3%; C3 exhibits λ_max_ = 520 nm, *f* = 0.999, LHE = 90.0%; and D3 presents λ_max_ = 457 nm, *f* = 1.248, LHE = 94.4%. These λ_max_/*f*/LHE data indicate strong photon-absorption characteristics and suggest competitive charge-separation behavior relative to that of the reference dye. Furthermore, the visible-light transition dipole moment (TDM) emerges as a robust descriptor for guiding the rational *in-silico* design of future DSSC sensitizers.

## Graphical abstract

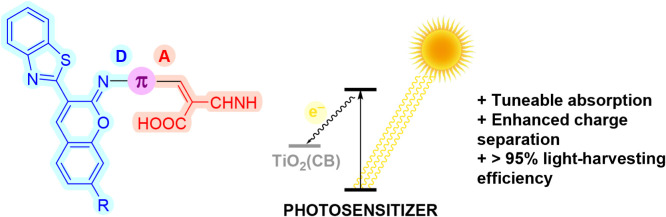

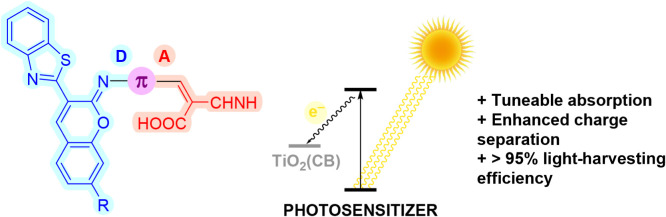

## 1 Introduction

A significant challenge for the 21st century is the production of clean energy while minimizing environmental impact.^1^ According to data from the 2023 Energy Institute (EI) Statistical Review of World Energy, 54% of all energy produced globally is intended for commercial and industrial usage. Smaller scale renewable energy sources still play a minor role compared to natural gas and coal in electricity generation.^2^ However, the same does not apply to residential energy consumption, where a shift toward renewable energy sources has become a global trend. In 2023, renewable energy was projected to make up 25% of the USA’s energy matrix,^3,4^ and already accounts for to 40% of all energy produced in the United Kingdom.^5^

Among the various methods for renewable energy production, solar energy has experienced substantial growth due to its minimal requirement for maintenance, low waste production, and capacity to harness energy from a globally available source: light. Throughout the years, devices capable of converting solar light to electricity have evolved due to the constant innovation of the materials employed to enhance efficiency and performance. Specifically, current third-generation solar cells already show performance comparable to silicon-based devices and approach the Shockley–Queisser efficiency limit.^6^ Recent developments report indoor light-to-energy conversion efficiencies exceeding 30.2% under 1500 lx illumination, without the use of cosensitizers, while also reducing impacts on human health and ecosystems.^6,7^ Among them, Grätzel cells, commonly known as dye-sensitized solar cells (DSSCs), have emerged as one of the most promising developments.^8,9^ Therein, an electric current is generated when sunlight excites the electrons of a sensitizer dye, which are transferred to the semiconduction band of a semiconductor material (commonly, TiO_2_) that binds the sensitizer.^10^ The semiconductor then acts as a medium for electron transport toward a cathode material (commonly, platinum) responsible for promoting the reduction of the cell electrolyte (usually, *I* ^–^/*I*_3_^–^)—which ultimately serves as a reducing agent to regenerate the oxidized dye and restart the process.^11,12^ The general operating mechanism behind DSSCs is summarized in Scheme S1 (see the Supporting Information).

Due to its decisive role in light-to-energy conversion, one of the fundamental areas in the development of DSSC is the design of new photosensitizer molecules.^13^ Among the materials that fulfill the requirements, dyes derived from coumarins (Figure 1a) consist of promising photosensitizers in DSSC, mainly due to their high quantum yields, high oxidation potential at the first excited state, as well as prolonged stability under photoirradiation.^14^ The absorption spectra of coumarin dyes have also been proven versatile for tuning, with extensive experimental–theoretical studies by Zeidler, Liu, Cole, and coworkers of simple coumarin dyes indicating consistent *red shifts* and *blue shift*s promoted by different electron-donating and electron-withdrawing groups (EDG and EWG, respectively) substituted at position 7 of the coumarin scaffold (Figure 1a).^15^ Additionally, for dyes containing *push–pull* moieties such as in the D-π-A systems discussed in the present work, the choice of the employed π-linker (also referred to as π-spacer) has been proven to be non-negligible and a valuable approach for the fine-tuning of the absorption spectra of novel sensitizers—mainly due to the π-linker’s effect on molecular planarity, light-harvesting capacity, and electronic recombination.^16–18^ One recent example was the decisive role of different thiazolo­[5,4-*d*]­thiazole π-linker moieties on the absorption spectra of triphenylamine-based dye sensitizers according to experimental developments by Reginato, Dessì, and coworkers.^19^ Furthermore, a recent *in-silico* assessment by Kandregula and Mandal has found that the choice of the π-conjugated acceptor moieties of D-π-A dyes also influenced the intensity of absorption bands in the visible spectrum and could enhance internal charge transfer (ICT) upon photoexcitation.^20^

**Figure 1:**
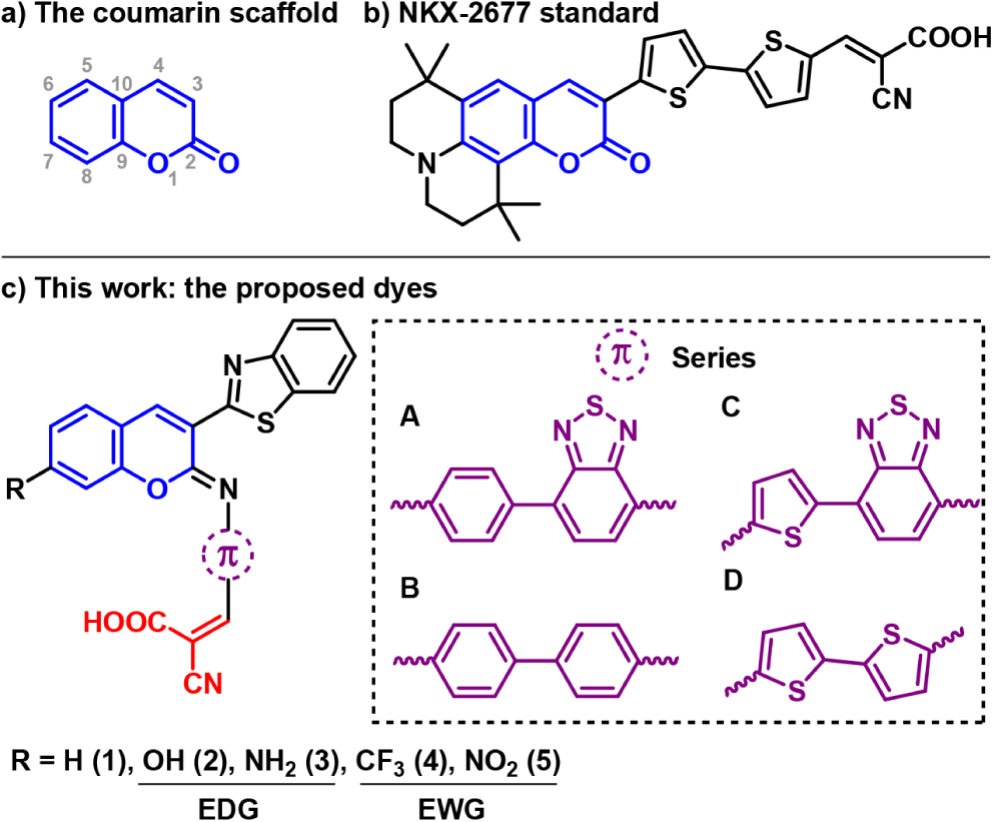
(a) Standard IUPAC numbering for atoms in the coumarin scaffold; (b) NKX-2677 reference dye by Hara and coworkers;^21^ c) Proposed iminocoumarin dye sensitizers in this work. The numerals 1–5 refer to the substituents at position 7 of the coumarin scaffold, while the letters A–D refer to the evaluated π-linker moiety. In blue, the coumarin scaffold (blue) and the “2-cyanoacrylic acid”-like (red) acceptor moiety.

Numerous series of coumarin-derived dyes intended for DSSC applications have already been synthesized.^22–24^ Among them is the remarkable NKX series, which includes dye sensitizer **NKX-2677** (Figure 1b).^24^ Notably, **NKX-2677** presents an intense absorption band (λ_max_) at the 511 nm range in a *t*-BuOH–Acetonitrile 50:50 (wt %) solvent, with an estimated ∼1.857 oscillator strength parameter for the referred S_0_ → S_1_ transition, 0.64 eV open-circuit voltage, and fast electron transfer to the conduction band of the TiO_2_ photoanode (<100 fs, compared to a fluorescence lifetime of ∼1 ns of the dye).^25^ Due to its remarkable performance, **NKX-2677** has been regarded as the most promising of the coumarin dyes for DSSCs,^14^ mentioned in numerous works as a standard to which new proposed dye sensitizers are compared.^25–27^

All considered, four new series of D-π-A iminocoumarin-based dye sensitizers containing a benzo­[*d*]­[1,3]­thiazole moiety at position 3 were proposed (Figure 1c). Benzothiazole is a heterocyclic compound widely used in molecular systems due to its unique electronic and optical properties. This structural group has proven effective in various applications, including the development of materials for electronic and photonic devices such as DSSCs. The presence of the benzothiazole group can significantly influence light absorption properties, charge transfer, and the stability of the compounds, making it a promising candidate for the development of new photosensitizers in DSSCs.^28^

Although theoretical design accelerates the discovery of new DSSC chromophores, its practical relevance depends on whether the proposed structures can be achieved by synthesis. Thus, beyond evaluating electronic, optical, and charge-transfer properties, assessing the synthetic feasibility of the iminocoumarin D-π-A derivatives is crucial. These are a group of synthetically accessible molecules with remarkable interaction with UV–vis light, and several well-established methods enable their construction and functionalization toward D-π-A architectures, including condensations with β-ketoesters, Pechmann-type cyclizations, Knoevenagel reactions to install acceptors, cross-coupling or nucleophilic substitution to introduce π-bridges, and imine formation to incorporate donor groups.^29–35^ These transformations are widely reported for related coumarins, supporting the practical accessibility of the designed molecules.^36^ Consequently, the proposed dyes align with known reactivity patterns, ensuring both theoretical relevance and realistic experimental viability.

To evaluate how the π-linker influences the absorption profile, planarity, and charge-separation characteristics of coumarin-based dyes, four distinct π-conjugated units were inspected: a 4-phenyl–benzo­[c]­[1,2,5]­thiadiazole motif (series **A**), a biphenyl motif (series **B**), a 4-thiophene–benzo­[c]­[1,2,5]­thiadiazole motif (series **C**), and a Th_2_-oligothiophene unit (series **D**), all combined with a 2-cyanoacrylic acid anchoring group. Also, the influence of electron-donating and electron-withdrawing substituents at the 7-position of the coumarin scaffold was assessed, with each entry in the series corresponding to a distinct EDG/EWG unit. The simulated photophysical profiles of the proposed dyes were compared directly with those of the benchmark **NKX-2677** sensitizer to contextualize their potential as candidates for DSSC applications. Within this framework, we aim to systematically compare the A–D π-linker series and the 7-position substituents against **NKX-2677** in terms of planarity, intramolecular charge transfer (ICT), injection and regeneration thermodynamics, and LHE-based spectral proxies.

## 2 Methodology

All gas-phase ground-state geometry optimizations and characterizations by vibrational frequency analysis were carried out by using density functional theory (DFT). We have employed the Coulomb-attenuated hybrid range-separated CAM-B3LYP exchange-correlation functional^37^ with the 6-31+G­(d,p) diffuse function-augmented Pople basis set.^38,39^ For the gas-phase simulation of Franck–Condon excitations for the first 10 singlet excited states, profiling of the expected emission spectra, and characterization of electric and transition dipole moment vectors, time-dependent density functional theory calculations (TD-DFT) were performed for the previously ground-state equilibrium geometries characterized at the same level of theory employing DFT. The choice of functional has been motivated by (i) the consistent small errors relative to experimental results in the characterization of singlet excited states for an extensive list of organic molecules;^40^ (ii) small relative error (1.4%) in TD-DFT estimations of S_0_ → S_1_ excitation energies for dye sensitizers employing similar π-spacer and electron acceptor moieties;^41^ (iii) particularly excellent characterization of intramolecular charge-transfer excitations (in agreement with complete-active-space Møller–Plesset second-order perturbation theory calculations) for various conjugated compounds at a fraction of the computational cost;^42^ and (iv) a significant range-separated Hartree–Fock exchange fraction (19% to 67%, increasing proportionally to interelectronic separation), which diminishes electron self-interaction errors (SIE).^43^

Moreover, the CAM-B3LYP functional has been found particularly reliable for investigations on the nature of photoexcitations,^44^ i.e. orbital overlap analysis and charge-transfer length measurement, as respectively realized in the lambda index (∧) analysis^45^ and charge-transfer length (Δ*r*) analysis.^46^ As for the **NKX-2677** dye, agreement was found between the experimental absorption spectrum (as measured in *t*-BuOH:acetonitrile 50:50 wt % solution^24^) and the gas-phase CAM-B3LYP/6-31+G­(d,p) simulated spectrum, having obtained 6% relative error for the S_0_ → S_1_ excitation energy once the electrostatic Integral Equation Formalism Polarizable Continuum Model (IEFPCM)^47^ was employed to simulate the acetonitrile solvent. Overall, gas-phase CAM-B3LYP/6-31+G­(d,p) simulations are expected to systematically overestimate excitation energies relative to the experimental UV–vis spectra in solution. For further discussion regarding the experimental and the gas-phase and condensed-phase simulations for the reference dye, refer to Section S2 (Figure S1) of the Supporting Information (SI) file.

Alongside the Kohn–Sham molecular orbitals determined at the CAM-B3LYP/6-31+G­(d,p) level, localized natural bond orbitals (NBO) calculations were also performed.^48^ For an accurate representation of multiconfigurational excitations, as well as the distribution of hole and electron pairs, natural transition orbitals (NTOs) were also calculated.^49^ For the plotting of π-electron delocalization across the molecule and investigation of possible delocalization pathways for intramolecular electronic transfer, π-LOL (Localized Orbital Locator) maps were plotted: these consist of employing Schmider and Becke’s traditional LOL functions with Lu and Chen’s general protocol^50^ for identifying π-orbitals in nonplanar molecules assisted by Becke’s orbital composition analysis method.^51^

To ensure that the characterized geometries corresponded to the populated global minima of the potential energy surface—and to ensure that the global minima provided an accurate characterization of a complete Boltzmann population-weighted absorption spectrum, conformational space surveys were employed through the Conformer–Rotamer Ensemble Searching Tool (CREST),^52^ where the GFN2-xTB semiempirical method was employed.^53^ The distinct GFN2-xTB lowest energy conformers were then fully optimized at the DFT: CAM-B3LYP/6-31+G­(d,p) level. Thermodynamic equilibrium between all conformers was assumed, and Boltzmann population calculations were performed for a standard state of 298.15 K and 1 M. The thermodynamic descriptors for electron injection and dye regeneration for the proposed dyes in Grätzel cells were calculated assuming a TiO_2_ semiconductor and *I*_3_^–^/*I* ^–^ electrolyte.^54^

All DFT, TD-DFT, and wave function analysis calculations were performed employing the Multiwfn^55,56^ and Gaussian09 suite of programs.^57^ The UV–vis spectra of the **A**–**D** dye series and the reference **NKX-2677** were generated from the TD-DFT electronic excitation data. Each transition contributes to the overall absorption profile through a Gaussian band-shape function, and the final spectrum is obtained by summing all individual contributions:

1 $$\varepsilon \left(\mathrm{\nu ̃}\right)=\overset{n}{\underset{i=1}{\sum }}\varepsilon_{i}\left(\mathrm{\nu ̃}\right)$$

For each excitation *i*, the corresponding contribution to the molar extinction coefficient using a Gaussian function centered at the excitation energy *ṽ_i_* and weighted by its oscillator strength *f*_*i*_ was calculated as

2 $$\varepsilon_{i}\left(\mathrm{\nu ̃}\right)=1.3062974\times 10^{8}\frac{f_{i}}{\sigma }\exp\left[-{(\frac{\mathrm{\nu ̃}-{\mathrm{\nu ̃}}_{i}}{\sigma })}^{2}\right]$$

where *ṽ* is the wavenumber (cm^–1^), *f_i_* is the oscillator strength, and σ is the Gaussian broadening parameter. In this work, we adopted a value of σ = 900 cm^–1^ (111.586 meV) which provides an appropriate spectral broadening for comparing the absorption features of the studied dyes. The broadened contributions were then summed to generate the simulated UV–vis spectra in terms of the molar extinction coefficient (ε). Additionally, oscillator-strength (*f*) stick spectra were plotted to visualize the position and relative intensity of each electronic transition. Further information regarding this broadening model is available in the Gaussian manual.^58^ Additional UV–vis spectrum data sorting, presented in the SI file, was realized employing the GaussSum application.^59^

## 3 Results and Discussion

### 3.1 Conformational Space Analysis and the Influence of Different π-Linker Moieties on Molecular Planarity

Different conformers of any molecule may differ in the intensity of their vibrational modes, the energies of frontier molecular orbitals, and the distribution of electronic density. Thus, they may interact differently with light. Given that one molecule must interact with one photon (the Stark–Einstein law), if thermodynamic equilibrium is assumed for all the different accessible conformers of a molecule, the experimentally observed absorption spectra must necessarily correspond to a Boltzmann population-weighted average spectrum of the populated conformers at a specific temperature, T (eq 3).^60,61^

3 $$P_{i}=\exp[-\left(\Delta G_{i}-\Delta G_{\min})/\mathrm{RT}\right]/[\underset{j}{\sum }\exp\left(-\left(\Delta G_{j}-\Delta G_{\min}\right)/\mathrm{RT}\right]$$

*P*_*i*_ = Boltzmann population (as a decimal) of conformer *i* at temperature T;

Δ*G*_*i*_ = thermally corrected Gibbs free energy of a conformer *i* at temperature T;

Δ*G*_min_ = thermally corrected Gibbs free energy of the ensemble minimum geometry (the global minimum) at temperature *T*;

Δ*G*_*j*_ = thermally corrected Gibbs free energy of ensemble conformers *j* at temperature *T*;

*R* = universal gas constant.

The proposed series of D-π-A iminocoumarin dye sensitizers presents various rotatable chemical bonds. Their many possible conformers are characterized by the different permutations of the dihedral angles (Φ_*n*_) in the coumarin, π-linker, and anchoring moieties (see Figure 2). To find and characterize the most populated conformer(s), a CREST (Conformer-Rotamer Ensemble Searching Tool) search using the semiempirical GFN2-xTB method for the lowest energy conformers was performed. The xTB-optimized geometries were then fully reoptimized at the DFT CAM-B3LYP/6-31+G­(d,p) level and properly characterized as minima of the potential energy surface through vibrational frequency analysis. Seeking trends among the least energetic (and therefore most populated) conformers’ geometries as influenced by the varying π-linker moieties from series **A**–**D**, the analysis reported herein focuses exclusively on the (**A**–**D**)**1** proposed dyes (R = H at the 7-coumarin position).

**Figure 2:**
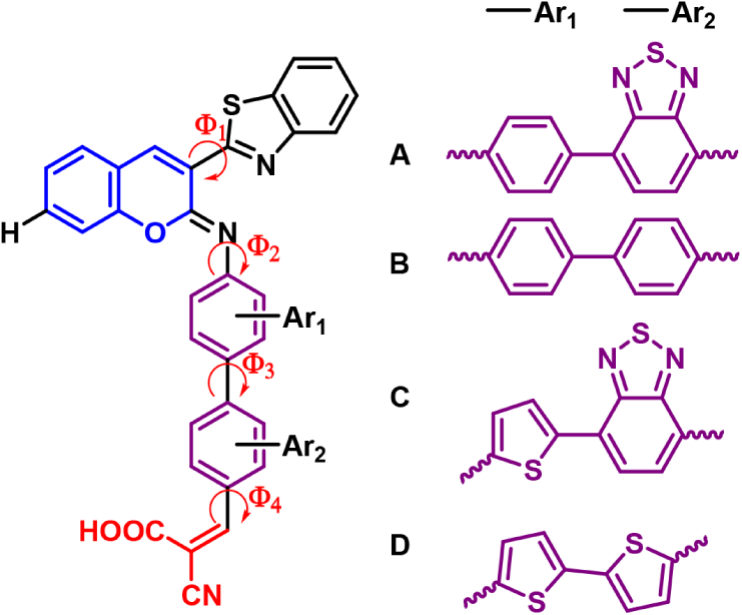
Overview of the conformational space available for the proposed **A1**–**D1** dyes (R = H at the 7-coumarin position).

All atomic position Cartesian coordinates for the evaluated conformers of compounds **A1**–**D1**, their respective Cartesian coordinates, as well as their electronic energy and thermostatic parameters (at 298.15 K, 1 bar, 1 mol L^–1^) are available in the final section of the Supporting Information (SI) file. For compounds **A**–**C1**, three similar local minima conformers were proposed to be populated by CREST employing the GFN2-xTB semiempirical method, while for compound **D1** only two geometries were proposed (Figure 3).

**Figure 3:**
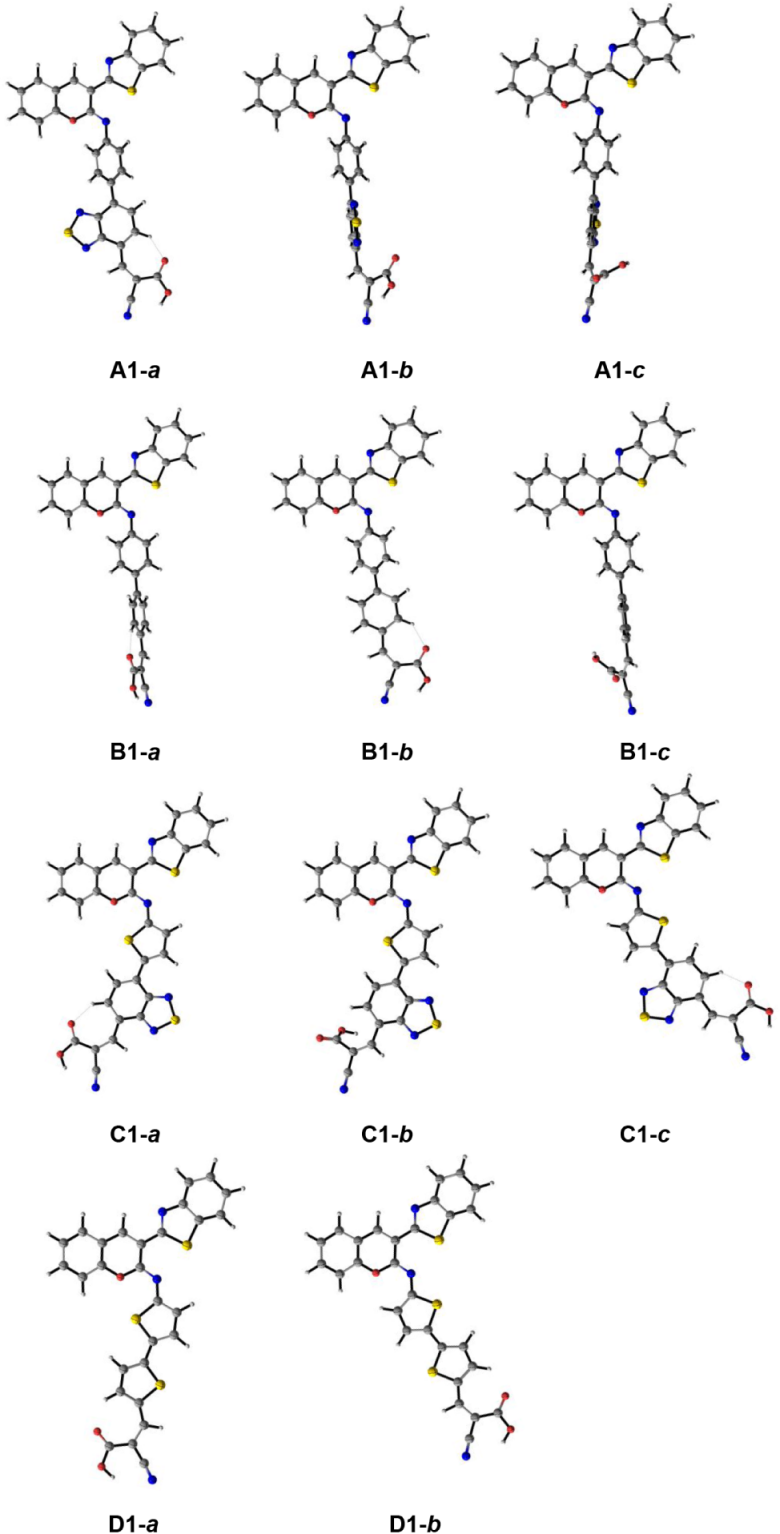
Optimized gas-phase geometries of all of the conformers for **A1**–**D1** at CAM-B3LYP/6-31+G­(d,p). Colors of the atoms: sulfur (yellow), oxygen (red), nitrogen (blue), carbon (gray), hydrogen (white).

Since the degree of planarity plays a key role in the overall performance of sensitizers for DSSC, with charge-transfer processes being favored by the alignment of electronic donor and acceptor moieties,^16,20,23,62^ Lu’s molecular planarity parameter (MPP) was employed to quantify the deviation from planarity for each conformer.^63^ Therein, the most possible fitting plane for any given geometry can be defined as *D* + *Ax* + *By* + *Cz* = 0, where *A*, *B*, and *C* correspond to the normal vectors to the fitting plane (found through the *single-value decomposition* method) and the parameter *D* is evaluated using the *x*, *y*, z coordinates corresponding to the geometric center of the assessed geometry. The MPP then corresponds to the root-mean-square sum of the distances of each of the *n* atoms in the molecule (*d*_*i*_) toward the fitting plane (eq 4), thus providing a general accurate measure of deviation from planarity for any system, with a value equal to 0 expressing that the molecule is perfectly planar (all atomic positions can be described by the fitting plane equation). A more detailed explanation and exemplified calculation of the MPP parameter (for the **NKX-2677** standard dye) is available in Section S3 of the SI (Supporting Information).

4 $$\mathrm{MPP}=\underset{j}{\sum }{[(1/n){(d_{i})}^{2}]}^{1/2}$$

The relative Gibbs-free energies, Boltzmann population analysis at a standard state (298 K, 1 bar, and 1 mol L^–1^), and MPP for each conformer are reported in Table 1.

**Table 1:** Gas-Phase and IEFPCM (Acetonitrile) CAM-B3LYP/6-31+G­(d,p) Relative Gibbs-Free Energies, Boltzmann Population Distribution at a Standard State (298.15 K, 1 Bar), and Molecular Planarity Parameter (MPP) Comparison for the Lowest-Energy Conformers of **A1**–**D1** Compounds (R = H) *versus* the NKX 2677 Standard

Gas phase				
Compound		Δ*G*_rel_, kJ mol^–1^	Boltzmann population of the conformer at a standard state, %	Molecular planarity parameter (MPP) of the conformer, Å
**A1**	*a*	0.0	98.8	0.55
	*b*	3.0	0.6	1.05
	*c*	3.0	0.6	1.14
**B1**	*a*	0.0	50.7	0.50
	*b*	0.13	48.1	1.02
	*c*	9.2	1.2	0.96
**C1**	*a*	0.0	93.3	0.00
	*b*	4.33	0.1	0.01
	*c*	1.56	6.6	0.40
**D1**	*a*	0.0	98.8	0.19
	*b*	10.9	1.2	0.15
**NKX-2677**	*c*	–	100.0	0.85

Solvent (acetonitrile)				
Compound		Δ*G*_rel_, kJ mol^–1^	Boltzmann population of the conformer at a standard state, %	Molecular planarity parameter (MPP) of the conformer, Å
**A1**	*a*	0.0	44.1	0.56
	*b*	0.42	21.6	1.06
	*c*	0.15	34.2	1.11
**B1**	*a*	0.33	24.7	1.04
	*b*	0.18	31.9	0.63
	*c*	0.0	43.3	0.95
**C1**	*a*	0.0	57.8	0.07
	*b*	0.51	24.3	0.40
	*c*	0.69	17.9	0.00
**D1**	*a*	0.0	83.5	0.10
	*b*	0.96	16.5	0.08
NKX-2677	*c*	–	100.0	0.84

For **A1**, after proper optimization at the DFT CAM-B3LYP/6-31+G­(d,p) level, the thermodynamically more stable and therefore more populated conformer (98.8%) **A1**-*a* corresponded to the most planar conformer with MPP = 0.55 Å. In compound **B1**, the biphenylyl-like π-linker moiety twists from planarity to relieve the strain between the two coplanar aromatic rings, resulting in the two most stable near-isoenergetic conformers. The general stability of more planar conformers was reaffirmed for dye **C1**, with DFT calculations indicating that the nonplanar conformer **C1**-*c* (MPP = 0.40) is significantly higher in energy than the other conformers. As for dye **D1**, two populated low-energy conformers were located, differing by almost 180° around the Φ_2_ dihedral angle (Figure 3). For both **C1** and **D1**, the observed thermodynamic preference was in complete agreement, favoring the global minima conformers **C1**-*a* and **D1**-*a* in which the sulfur atom from the thiophene moiety faces the oxygen atom of the imino-coumarin scaffold.

Although the global minima-energy geometries for compounds **A1**, **B1**, **C1**, and **D1** presented a greater degree of planarity (that is, a lower value of MPP—see Table 1 for the measured MPP parameters) than the **NKX-2677** reference dye, the contribution to the latter’s large MPP value is due to the existence of out-of-plane sp^3^-hybridized atoms at the coumarin side chain—in addition to a preference for a slightly twisted “thiophene-thiophene” arrangement of the π-linker moiety (Φ_3_ = 166.7 deg.)—also observed for the ground-state global minimum equilibrium geometry (**D1**-*a*) for dye **D1** (Φ_3_ = 166.1 deg.). For all evaluated dyes, the π-linker has been found to be a non-negligible contributor to molecular planarity, and thus to electronic conjugation and charge delocalization upon photoexcitation, as in similar D-π-A dyes in the literature.^16–18,64,65^

### 3.2 UV–Vis Absorption Spectra and HOMO–LUMO Gap Narrowing

Absorption in the visible region of the UV–vis spectrum is a requirement for a dye sensitizer in DSSC applications. Several interconnected factors may affect a chromophore’s absorption profile, such as electric dipole orientation, vibrational modes’ composition, structure and planarity, electronic density distribution, and relative stability of the frontier molecular orbitals (FMO).^66,67^ In Table 2, for all populated global-minimum geometries of the proposed dye sensitizers at a standard state, the TD-DFT-estimated vertical excitations λ_max_ (assigned to the S_0_ → S_1_ photoexcitation, which corresponds to the most intense absorption line in all systems), the associated oscillator strength *f*, the molecular planarity parameter (MPP), and the TD-DFT configuration composition are reported. For all dyes, the absorption spectrum of the global-minimum conformer was found to dominate under standard-state conditions (see S4 of the Supporting Information file) to accurately represent the Boltzmann population-weighted absorption spectrum at 298.15 K.

**Table 2:** CAM-B3LYP/6-31+G­(d,p) Gas-Phase Absorption Spectra for the Global Minima Conformers for Series **A**–**D** and **NKX-2677** Reference Dye^a^ ^c^

	R	Molecular planarity parameter (Å)	λ_max_ (nm)	*f*	Configuration composition for the S_0_ → S_1_ transition^b^
**A1**	–H	0.55	417	1.056	0.67ψ_(H→L)_ + 0.24ψ_(H–1→L)_
**A2**	–OH	0.52	419	1.062	0.57ψ_(H→L)_ + 0.34ψ_(H–1→L)_
**A3**	–NH_2_	0.53	427	0.971	0.47ψ_(H→L)_ + 0.44ψ_(H–1→L)_
**A4**	–CF_3_	0.54	409	1.066	0.74ψ_(H→L)_ + 0.17ψ_(H–1→L)_
**A5**	–NO_2_	0.88	409	1.006	0.77ψ_(H→L)_
**B1**	–H	0.50	355	0.514	0.44ψ_(H→L)_ + 0.43ψ_(H→L+1)_
**B2**	–OH	0.52	362	0.824	0.33ψ_(H→L)_ + 0.48ψ_(H→L+1)_
**B3**	–NH_2_	0.53	370	0.789	0.23ψ_(H→L)_ + 0.60ψ_(H→L+1)_
**B4**	–CF_3_	0.54	366	0.646	0.69ψ_(H→L)_ + 0.14ψ_(H→L+1)_
**B5**	–NO_2_	0.99	390	0.480	0.80ψ_(H→L)_
**C1**	–H	0.00	500	1.045	0.91ψ_(H→L)_
**C2**	–OH	0.00	504	1.034	0.89ψ_(H→L)_
**C3**	–NH_2_	0.03	520	0.999	0.86ψ_(H→L)_
**C4** ^d^	–CF_3_	0.54	489	1.013	0.92ψ_(H→L)_
**C5**	–NO_2_	0.00	489	0.896	0.90ψ_(H→L)_
**D1**	–H	0.19	444	1.303	0.90ψ_(H→L)_
**D2**	–OH	0.18	446	1.309	0.88ψ_(H→L)_
**D3**	–NH_2_	0.19	457	1.248	0.84ψ_(H→L)_
**D4** ^d^	–CF_3_	–			
**D5**	–NO_2_	0.20	454	0.807	0.79ψ_(H→L)_
NKX-2677	–	0.85	441	1.886	0.77ψ_(H→L)_ + 0.10ψ_(H–1→L)_

a Herein are reported the gas-phase ground-state equilibrium geometries’ measured molecular planarity parameter (MPP), the S_0_ → S_1_ vertical excitation wavelength (λ_max_), its associated oscillator strength *f*, and its configuration composition.

b The contribution of a configuration is herein reported provided that the configuration coefficient is superior or equal to the 0.1 threshold.

c The observed deviation from planarity is exclusively attributed to the −CF_3_ substituent, as can be verified through the Cartesian coordinates available in the final section of the SI.

d The geometry failed to converge at the CAM-B3LYP/6-31+G­(d,p) level of theory.

Among all the proposed dyes, only series **B** failed to present any absorption of light in the visible range of the UV–vis spectrum (with λ_max_ < 400 nm, Table 2) and thus would not be a promising candidate as a dye sensitizer for DSSC application. For these dyes, a significant deviation from planarity (as measured by the MPP parameter) occurs to minimize the repulsion between the two aromatic rings in the π-linker moiety. Comparably, benzo­[*c*]­thiadiazole-substituted series **A**’s red-shifted absorption spectrum (which includes a relatively intense absorption band in the 409–427 nm range) can be attributed to an important stabilization of the LUMO molecular orbital by the benzo­[*c*]­[1,2,5]­thiadiazol-like π-linker moiety (see Table 3a). Our findings reinforce the growing importance in the literature of thiazole, thiophene, and related fused and unfused π-linker moieties in chromophore design through LUMO stabilization.^18–21,27^

**Table 3:** A) CAM-B3LYP/6-31+G­(d,p) Gas-Phase Kohn–Sham Frontier Molecular Orbital (FMOs) Energies and HOMO–LUMO (Δ*E*_H–L_) Energy Gap for the Global Minima Geometries of Dyes (**A**–**D**)**1** Compared **NKX-2677** Reference Dye; B) Reactivity Global Descriptors^a^

3a	A1	B1	C1	D1	NKX-2677
*E*_HOMO–1_ (eV)	–7.8	–7.8	–7.9	–7.8	–7.5
*E*_HOMO_ (eV)	–7.3	–7.3	–6.9	–6.9	–6.6
*E*_LUMO_ (eV)	–2.4	–1.9	–2.6	–2.2	–1.9
*E*_LUMO+1_ (eV)	–1.6	–1.5	–1.7	–1.7	–1.1
Δ*E*_H–L_ (eV)	4.8	5.6	4.3	4.7	4.7

**3b**	**A1**	**B1**	**C1**	**D1**	NKX-2677
*I* (eV)	7.3	7.3	6.9	6.9	6.6
*A* (eV)	2.4	1.9	2.6	2.2	1.9
μ (eV)	–4.9	–4.6	–4.7	–4.6	–4.2
χ (eV)	4.9	4.6	4.7	4.6	4.2
*h* (eV)	4.8	5.4	4.3	4.7	4.7
*S* (eV^–1^)	0.2	0.2	0.2	0.2	0.2
ω (eV)	2.4	2.0	2.6	2.2	1.9
*N* (eV)^[a]^	3.6	3.6	4.0	4.0	4.3

a *I* denotes the molecular ionization potential, *A* corresponds to the electronic affinity, μ is the electronic chemical potential, χ is the Mulliken electrophilicity index, *H* and *S* correspond to chemical hardness and chemical softness, respectively, ω is the electrophilicity index, and *N* is the nucleophilicity index at the TCE scale, measured as *N* = *E*_HOMO_ (dye) – *E*_HOMO_ (tetracyanoethylene, or TCE), where *E*_HOMO_(TCE) = −10.9 ev.

Figure 4a and b shows the relationships between the π-linker torsion angle Φ_3_ and the HOMO–LUMO energy gap with λ_max_ for the (**A–D**)**1** (R = H) dyes, respectively. Comparing the visible-light-absorbing series, a notable trend was the further red-shifted absorption observed in series **C** and **D**, resulting from a larger configuration coefficient associated with the least energetic HOMO → LUMO electronic transition, as well as the narrowing of the HOMO–LUMO gap. In contrast to these more planar series, the less planar series **B** dyes presented less intense absorption of visible light and a more pronounced multiconfigurational composition of the S_0_ → S_1_ photoexcitation. As such, a general observed trend in the CAM-B3LYP/6-31+G­(d,p) simulated spectra is that the contribution of HOMO → LUMO electronic transitions coincided both with a narrowing of the HOMO–LUMO gap and an increase in molecular planarity as enabled by the employed π-linker moiety, with angle Φ_3_ rising in the order **C1** (0 deg.) < **D1** (14 deg.) < **A1** (37 deg.) **< B1** (38 deg.). However, Figure 4a shows no linear relationship between Φ_3_ and λ_max_, indicating that additional structural factors contribute to the observed photophysical trends. Variations in other torsional angles and intrinsic electronic characteristics of the π-linker appear to influence the photophysical response.

**Figure 4:**
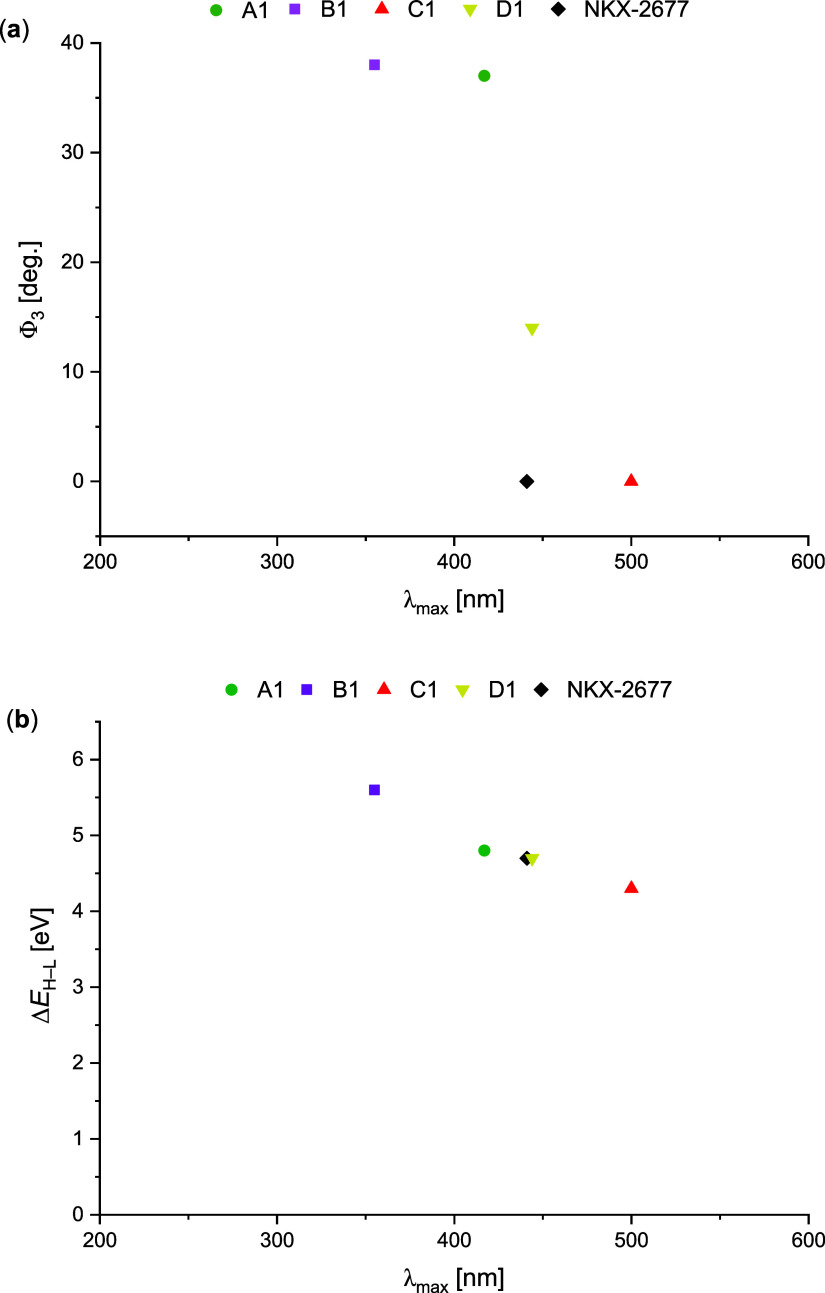
(a) π-Linker torsion angle (Φ_3_) versus λ_max_ for the (**A–D**)**1** (R = H) dyes and the reference **NKX-2677**; (b) HOMO–LUMO energy gap (Δ*E*_H–L_) versus λ_max_ for the (**A–D**)**1** (R = H) dyes and the reference **NKX-2677**. Values were obtained at the TD-CAM-B3LYP/6-31+G­(d,p), gas-phase.

Table 3a presents the Kohn–Sham (KS) ground-state frontier molecular orbital energies for the (**A**–**D**)**1** (R = H) dyes and Table 3b presents the general conceptual DFT reactivity global descriptors expected for the proposed series of dye sensitizers.^68^ While conceptual DFT and reactivity descriptors derived from the Kohn–Sham formalism (Table 3b) offer an imperfect representation of the electronic density—given their strong dependence on the chosen exchange–correlation functional^69^—the corresponding conceptual DFT (CDFT) descriptors calculated for the proposed molecules, such as the molecular ionization potential (*I*) and electron affinity (*A*) (both equal in magnitude to the HOMO and LUMO energies, respectively), provide simple predictors of favorable thermodynamics for electron injection and overall photovoltaic performance, when compared with the **NKX-2677** reference dye.^70^ The full set of ground-state Kohn–Sham frontier molecular orbital (FMO) energies for all evaluated dyes (**A–D**)**1–5**, along with the expressions used to compute the CDFT descriptors, is provided in S5 of the SI.

Overall, only the **B1**–**5** dyes failed to exhibit the desired photophysical profile, as the **B** series was not expected to absorb visible light. Furthermore, the existence of two nearly isoenergetic conformers—each displaying markedly different geometries, vibrational mode compositions, electronic eigenstates, and consequently distinct absorption characteristics—indicates that their UV–vis spectrum at 298.15 K is more accurately represented through a Boltzmann-weighted combination of the individual conformer spectra, which is provided in the SI (Section S4, Figures S5 and S6), supporting the conclusions discussed here.

Figure 5 shows the absorption spectra of the evaluated dyes, comparing only the most red-shifted members of each series (**A3**, **C3**, and **D3**) with the **NKX-2677** reference dye in the gas phase (Figure 5a) and in acetonitrile (Figure 5b), highlighting their robustness to solvent effects. This direct comparison focuses on the “3” derivatives of each series, because these compounds contain a nitrogen atom at position 7, as does **NKX-2677**. Notably, substitution at the 7-position of the coumarin ring in the **C**-series dyes provides an effective strategy for tuning the absorption profile of new C-type benzo­[d]­[1,3]­thiazole imino-coumarin chromophores, with electron-donating groups (EDGs) and electron-withdrawing groups (EWGs) promoting red-shifted and blue-shifted absorptions, respectively (see Figure S12, Sections S10, SI). This behavior is consistent with experimental and theoretical analyses by Zeidler, Liu, and coworkers on various 7-substituted coumarin chromophores,^15^ further supporting the potential of these dyes as photosensitizers and cosensitizers for DSSC applications.

**Figure 5:**
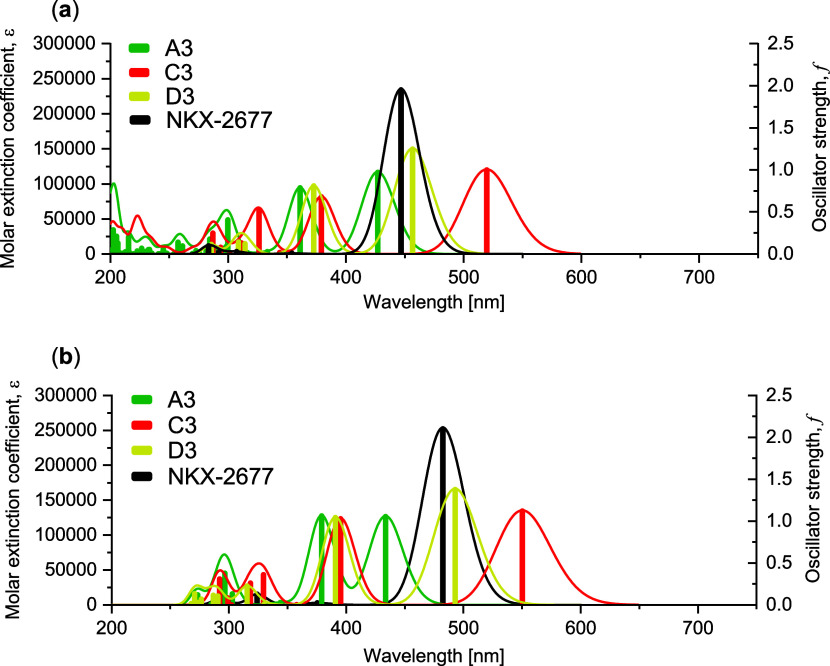
Simulated UV–vis absorption spectra in terms of molar extinction coefficient (ε) and oscillator strength (*f*), calculated at the TD-CAM-B3LYP/6-31+G­(d,p) level, comparing the **NKX-2677** reference dye with the proposed 7-amino analogue series (**A–D**)**3**. (a) Gas-phase simulated UV–vis spectra; (b) IEFPCM (acetonitrile) solvent-phase simulated UV–vis spectra.

In addition to the absorption spectra reported herein, the emission spectra for the proposed dye sensitizers **A1, C1,** and **D1** dyes are available in Section S6 of the Supporting Information (SI) file. Given the significant (≥100 nm) expected Stokes shift observed in specific dyes from series **A** and **D** — and the potential of these materials as organic fluorophores for applications such as imaging^71^ —the emission profile, emission oscillator strengths, and expected Stokes shifts are reported in Section S7 of the SI. The emission profile of series **C** dyes is not included due to the relatively small Stokes shift (∼80 nm) expected for the series.

### 3.3 Electron Delocalization Pathways and Influence of π-Linker Moieties

Due to the decisive role of molecular planarity in promoting visible-light absorption (Table 2), and considering that absorption in the visible region of the UV–vis spectrum is predominantly governed by π–π* and *n*–π* transitions (while charge-transfer and charge-recombination processes rely primarily on π-electronic motion,^16,41,72^ the π-electron delocalization pathways of the global-minimum conformers were mapped using Schmider and Becke’s localized orbital locator (LOL) functions.^51^ Because several of the evaluated dyes are not perfectly planar, π- and σ-orbital mixing becomes unavoidable, preventing a rigorous π-orbital classification based solely on the presence of nodal planes containing the internuclear axis, as would be appropriate for planar systems.^73^ Therefore, natural bond order (NBO) localized orbitals were first computed for each populated global-minimum structure. Becke’s composition analysis (required due to the diffuse functions in the 6-31+G** basis set) was then applied to distinguish π- from σ-orbitals following the protocol of Lu and Chen.^50^ To represent the dominant charge-delocalization pathway from position 7 of the coumarin scaffold toward the “2-cyanoacrylic acid” acceptor unit (highlighted in Figure 1c), the LOL-π function was applied to the (A–D)­2 dyes (R = OH at the 7-coumarin position). These systems were selected as series-specific reference dyes because they are the simplest members containing π-electron-donor groups.

The LOL-π maps for the (**A**–**D**)**2** proposed dyes (with the coplanar 7-OH substituent) are shown in Figure 6. Herein, LOL-π is employed solely as a qualitative indicator of π-electron delocalization along the donor → acceptor axis, complementing the quantitative structural metrics provided by the torsional angles and the molecular planarity parameter (MPP). Because LOL-π values are highly sensitive to the chosen integration path and grid resolution, they do not provide a fully consistent basis for quantitative comparison across dye series. For this reason, our discussion focuses on qualitative features and relative trends rather than numerical averages.

**Figure 6:**
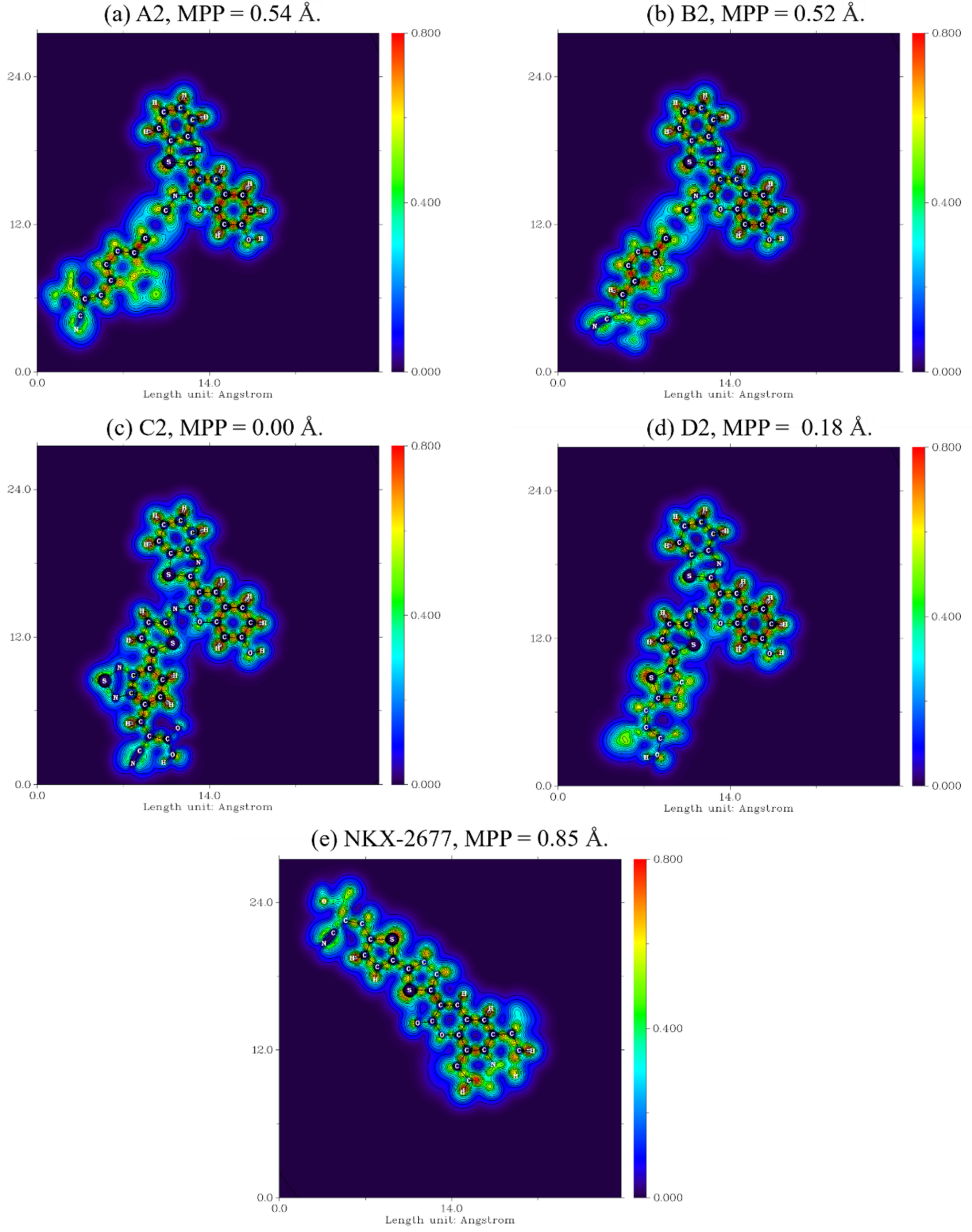
CAM-B3LYP/6-31+G­(d,p) local orbital locator-π (LOL-π) maps employing Becke’s composition analysis method plus molecular planarity parameter (MPP). (a)–(d) maps correspond to the LOL-π profile for the global minima geometry of the proposed (**A**–**D**)**2** dye sensitizers (R = OH substituent at the 7-coumarin position), while (e) map is applied to the **NKX-2677** reference coumarin-based dye. The *x* and *y* axes denote geometric distances (Bohr). In the employed blue-red gradient, redder values represent a higher value of the LOL-π function and, thus, higher Pauli kinetic energy due to π-electron localization.

In the kinetic interpretation of the blue-red color grading of a LOL-π mapping, red denotes regions of greater Pauli kinetic energy density (also known as *excess* kinetic energy) due to π-electron localization. This indicates possible paths for electronic transfer through chemical bonding from the coumarin to electron acceptor moieties. Conversely, blue denotes nonbonding regions, boundary regions (such as electronic inner shells, with zero magnitude of π-electron gradient), and, in general, regions of low π-electronic density and, thus, low Pauli kinetic energy density. An immediate result of the LOL profile (as well as other kinetic energy approaches to wave function analysis) applied exclusively to the π-orbitals is the profiling of π-electron motion confinement. Here, a high (redder) LOL-π value reflects higher Pauli kinetic energy density in regions where π-electron motion confinement is more likely.

For both dye series **A** and **B**, the measured smaller degree of planarity resulting from the employment of both the 4-phenyl-benzo­[*c*]­[1,2,5]­thiadiazol-like linker (**A** series) and biphenyl-like (**B** series) results in two regions of confinement of electronic motion, with a less likely electronic delocalization path toward the acetonitrile acceptor moiety that binds the dye to the TiO_2_ photoanode. As proof of concept of the unfavorable contribution of the biphenyl-like π-linker, the geometry and LOL-π were evaluated for an *o*-Me-substituted analogue of the **B2** dye, further distorting the planarity to an MPP of 0.74 Å (and a dihedral Φ_3_ = 127.86 deg.), in comparison to the original **B2** dye—where MPP = 0.50 Å and Φ_3_ = 143.31 deg.). Predictably, the same profile of π-electronic motion confinement arose, as reported in S8 of the SI file.

Hence, given the previously discussed poor visible-light absorption predicted for the **B**-series dyes and their unfavorable π-linker geometries for promoting electronic delocalization upon photoexcitation, our analysis was subsequently restricted to the **A**, **C**, and **D** series for further screening of viable photosensitizers for DSSC applications.

### 3.4 Light-Harvesting Efficiency (LHE), Charge Separation, and Natural Transition Orbitals (NTOs)

The oscillator strength *f* reflects the coupling between a molecule’s electric dipole and the incident radiation at a specific wavelength. Higher oscillator strengths indicate stronger photon absorption and are therefore important in the design of photosensitizers, as they are commonly used to estimate the light-harvesting efficiency (LHE) of a dye, as expressed in eq 5.^74^ In this context, the LHE at a given wavelength corresponds to the fraction of incident photons (irradiation *I*_0_) absorbed by the dye (absorbed irradiation *I*_A_).^75^ It is important to note, however, that LHE = 1 – 10^–*f*^ is a simplified optical proxy and should not be interpreted as a predictor of actual device performance since it does not account for processes such as electron injection, dye regeneration, charge transport, or recombination. Herein, LHE is used strictly as a descriptor of optical absorption strength. Notably, the intense S_0_ → S_1_ absorption of the **NKX-2677** reference dye (*f* ≈ 1.9, Table 2) contributes to its strong light-harvesting capability, which is one of the factors underlying its suitability for DSSC applications.^65^

5 $$\mathrm{LHE}=I_{A}/I_{0}=1-10^{-f}\left(\%\right)$$

Among all the proposed photosensitizers, series **D** (specifically, compounds **D1**–**3**) exhibited notably high oscillator strength (and thus greater LHE) for the S_0_ → S_1_ photoexcitation—although still lower than that of the reference dye. Since the photoexcitation energy for the S → S_1_ transition (*E*_S0→S1_) in all the proposed dyes is similar to that of the **NKX-2677** reference dye (see Table 4), the decisive contributor to the higher oscillator strengths (and therefore, light-harvesting efficiency) observed for both the reference dye and series **D** is the large square norm of the transition dipole moment (TDM) |μ_S0→S1_|^2^, for the S_0_ → S_1_ photoexcitation. This norm is proportional to the oscillator strength (eq 6) for the quantum absorption oscillator model:^76–78^

6 $$f_{S0\to S1}=\left[2m_{e}\left(E_{Si\to Sj}\right){|\mu_{S0\to S1}|}^{2}\right]/\left(3\mathrm{\hbar e}\right)$$

**Table 4:** TD-DFT: CAM-B3LYP/6–31+G­(d,p) expected Light-Harvesting^a^ Efficiencies (LHE) for the Excitation S_0_ → S_1_ photoexcitation, Excitation Energies (*E*_S0→S1_), Squared Norm of the Electronic Transition Dipole Moment |*μ*_S0→S1_|^2^, Electric Dipole Moment Change Δ*μ*, Charge-Transfer Length Δ*r*, Hole and Electron Overlap Index ∧, and Excitation Type Characterization as Charge Transfer (CT) *versus* Local Excited (LE)

	LHE, %	*E*_S0→S1_ (eV)	|μ_S0→S1_|^2^	Δμ	∧^a^	Δ*r* (Å)^b^	Excitation type
**A1**	91.2	3.0	7.26	19.13	0.46	6.71	CT
**A2**	91.4	3.0	7.35	21.27	0.45	6.94	CT
**A3**	89.3	2.9	6.83	28.53	0.43	6.78	CT
**A4**	91.4	3.0	7.19	13.36	0.51	5.94	CT
**A5**	90.2	3.0	6.79	6.17	0.63	2.67	LE
**C1**	91.0	2.5	8.60	14.97	0.61	3.36	LE
**C2**	90.8	3.7	8.58	16.74	0.59	4.26	CT
**C3**	90.0	4.0	8.56	20.82	0.54	4.82	CT
**C4**	90.4	2.5	8.19	10.64	0.64	3.31	LE
**C5**	87.3	2.5	7.23	4.76	0.68	1.64	LE
**D1**	95.0	2.8	9.54	5.58	0.67	2.65	LE
**D2**	95.1	2.8	9.65	7.26	0.65	3.30	LE
**D3**	94.4	2.7	9.40	10.86	0.60	4.30	CT
**D5**	84.5	2.7	6.06	8.85	0.58	4.28	CT
NKX-2677	98.7	2.8	13.73	11.82	0.59	5.37	CT

a ∧ ≤ 0.6 has been adopted for the classification of a photoexcitation as internal charge transfer (CT), while in.

b A threshold of Δ*r* ≥ 2.0 Å is expected for CT photoexcitations.^46^

Another crucial trait for sensitizer materials is effective charge separation, which minimizes efficiency loss from charge recombination (where electrons injected into the semiconductor conduction band are retransferred to the oxidized dye).^11^ To predict charge separation and assess the likelihood of recombination losses, reference is made to the experimental evaluations reported by Lu and Yu.^79^ Their findings suggest that a stronger predictor of enhanced charge separation in heterojunction solar cells is a greater magnitude of the electric dipole moment change (**Δ**μ) between its excited state (for the herein relevant first singlet excited state, μ_S1_) and ground state (μ_S0_), further reiterated by Borges, Aquino, Lischka, and coworkers for organic photovoltaic devices in eq 7:^80^

7 $$\mathrm{Charge}\;\mathrm{separation},\alpha \Delta \mu =\left|\mu_{S1}-\mu_{S0}\right|$$

Notably, series **A** and **C** are expected to exhibit more pronounced degree of charge separation compared to the **NKX-2677** reference dye and series **D**—both of which containing a Th_2_-oligothiophene (2,2′-bithiophene) moiety as the π-linker and a lesser expected Δμ. The selected π-linker is also found to play a decisive role in promoting charge separation and, consequently, effective charge transfer. In series **D**, as in other D-π-A chromophores designed for DSSC applications, geometry-driven excited-state intramolecular charge transfer (ESICT) and charge separation are observed upon photoexcitation.^81,82^ Specifically, dyes **D1**–**3** exhibited a notable planarization in their ground-state equilibrium geometry upon photoexcitation to the first singlet excited state, S_1_, with the “thiophene–thiophene” dihedral angle (Φ_3_) shifting from 166.01 (deg.) in the S_0_ to 179.91 (deg.) at the S_1_ vibrationally relaxed equilibrium geometry (see geometries D1*–D3* on pages 105–109 of the SI file). As a result, the measured MPP goes from ∼0.20 Å at the S_0_ equilibrium geometry to 0 Å at the S_1_. Likewise, in the reference dye **NKX-2677,** the “thiophene–thiophene” dihedral also planarizes from 166.1 (deg.) at the S_0_ to 179.9 (deg.) at the S_1_ equilibrium geometry. The result of such planarization enabled by the Th_2_-oligothiophene π-linker and the smaller change in the components of the electric dipole moment vectors before and after photoexcitation, in comparison to the π-linkers evaluated for series **A** and **C**, is a smaller |μ_S1_–μ_S0_| and less pronounced charge separation.

Moreover, in the investigation of charge separation upon photoexcitation, the electron–hole overlap index (Λ) was also employed, which measures the range of a photoexcitation and is proportional to the spatial overlap (*O*_*ia*_) between involved occupied orbitals ψ_*i*_(*r*) and virtual orbitals ψ_*a*_(*r*), such as in TD-DFT calculations (eq 8).^45^ Therein, lower values of spatial overlap (closer to zero) are associated with long-range Rydberg and charge-transfer excitations, while larger values (closer to 1) are associated with local excitations.

8 $$\Lambda \alpha O_{ia}=\int \left|\psi_{i}\left(r\right)\right|\left|\psi_{a}\left(r\right)\right|\mathrm{dr}$$

The ∧ index presents a straightforward qualitative diagnostic characterization of the nature of the photoexcitations. Moreover, this index is particularly favorable in the investigation of the nature of excitation simulated through the means of TD-DFT employing the CAM-B3LYP functional (such as in the present work). For a variety of organic molecules, this functional’s description of long-range, nonlocal exact orbital exchange has been found to provide a consistent threshold (around ∧ = 0.60) for the characterization of charge-transfer photoexcitations compared to local excitations, as well as small errors in the estimation of the associated excitation energies of these charge-transfer excitations. In addition to the electron overlap index ∧ (described as qualitative in nature),^45^ the hole–electron distance index (Δ*r*) was also employed for characterizing the nature of these photoexcitations, with Δ*r* being proportional to the distance vector between the occupied and virtual orbital pairs’ centroids involved in a photoexcitation.^46^

Table 4 reports the expected light-harvesting efficiency (LHE) for the S_0_ → S_1_ photoexcitation energies, excitation energy (*E*_S0→S1_), norm of the electric dipole moment change (**Δ**μ), squared norm of the S_0_ → S_1_ transition dipole moment vector |μ_S0→S1_|,^2^ electron overlap index ∧, and the charge-transfer length parameter Δ*r*.

Table 5 reports the *x*, *y*, and *z* components of the electric dipole moment vectors associated with the S_0_ → S_1_ photoexcitation for dyes **A3**, **C3**, and **D3**, as well as for the reference dye **NKX-2677**. Among these systems, dyes **A3** and **C3** exhibit the highest predicted charge-separation magnitudes (Δμ) within their respective series, as shown in Table 4, reaching values comparable to that of the **NKX-2677** standard. In donor–acceptor molecular systems, electronic coupling controls both the efficiency of charge separation and the rate of charge recombination. In DSSCs, this behavior is strongly captured by the density-shift parameter Δμ. Larger Δμ values indicate greater spatial displacement between the hole and electron upon excitation, promoting the formation of the interfacial charge-transfer (CT) state and facilitating faster electron injection into TiO_2_. Conversely, small Δμ values keep the e^–^/h^+^ pair strongly bound, increasing the recombination probability.^83–85^ Thus, the high Δμ predicted for dyes **A3** and **C3** highlights their potential to enhance charge separation at the dye–semiconductor interface. When evaluated together with the thermodynamic driving force for charge injection, Δμ emerges as a decisive descriptor for efficient exciton dissociation and reduced recombination losses. Section S9 of the Supporting Information (SI) presents the decomposition of the *x*, *y*, and *z* components of the transition dipole moment vector associated with S_0_ → S_1_ photoexcitation.

**Table 5:** CAM-B3LYP/6-31+G­(d,p) Composition of the Electric Dipole Moment Vectors at the Ground-State (S_0_) and First Singlet Excited State (S_1_)

	S_0_			S_1_		
	μ_*x*_	μ_*y*_	μ_*z*_	μ_*x*′_	μ_*y*′_	μ_*z*′_
**A3**	2.487091	1.666969	0.568743	7.691116	2.819655	0.449612
**C3**	–4.880620	–2.120597	0.056154	–8.306173	–2.041723	–0.025853
**D3**	2.906674	1.739852	–0.332800	6.187135	1.954285	–0.279101
NKX-2677	–4.880620	–2.120597	0.056154	–8.306173	–2.041723	–0.025853

In addition, all three dyes are expected to exhibit LHE (%) close to or exceeding 90% (also comparable to the reference dye), with **D3** showing particularly comparable performance to the reference photosensitizer. Furthermore, the classification of the S_0_ → S_1_ transitions of the proposed dyes through orbital overlap analysis (as indicated by the electron overlap index ∧) suggests them as long-range, internal charge-transfer excitations for various of the proposed dye sensitizers (**A1**–**4**, **C2, C3**, **D3,** and **D5**). Although Δ*r* values above 2.0 Å typically indicate long-range charge transfer, the **C5** sensitizer transition with Δ*r* = 1.64 Å yet a high oscillator strength (*f* ≈ 0.8) represents a known deviation from this threshold. This behavior is consistent with a mixed local/charge-transfer character, where partial orbital localization boosts the oscillator strength while moderate hole–electron separation keeps Δ*r* below the CT limit.

Finally, to assess the spatial disposition of the hole and electron formed upon charge-transfer S_0_ → S_1_ photoexcitation, natural transition orbitals (NTOs) derived from the one-electron transition density matrix were employed. For this analysis, dyes **A3**, **C3**, and **D3** were selected, which present an amino group at the 7-coumarin as in all evaluated systems—similar to the reference dye **NKX-2677** (Figure 1). The calculated NTO orbitals succeeded in representing the electronic excitations through single dominant orbital pair contributions, thus presenting an accurate portrayal of *hole* (herein labeled as HOMO_NTO_) and *electron* (labeled as LUMO_NTO_) spatial distribution (Figure 7). Seeking to quantify the change in electronic density distribution in “2-cyanoacrylic acid”-like acceptor moiety (Figure 1c) upon photoexcitation relative to the reference dye **NKX-2677** (where the same acceptor moiety is employed in the anchoring unit), interfragmentary Hirshfeld density partition calculations were performed.^86^

**Figure 7:**
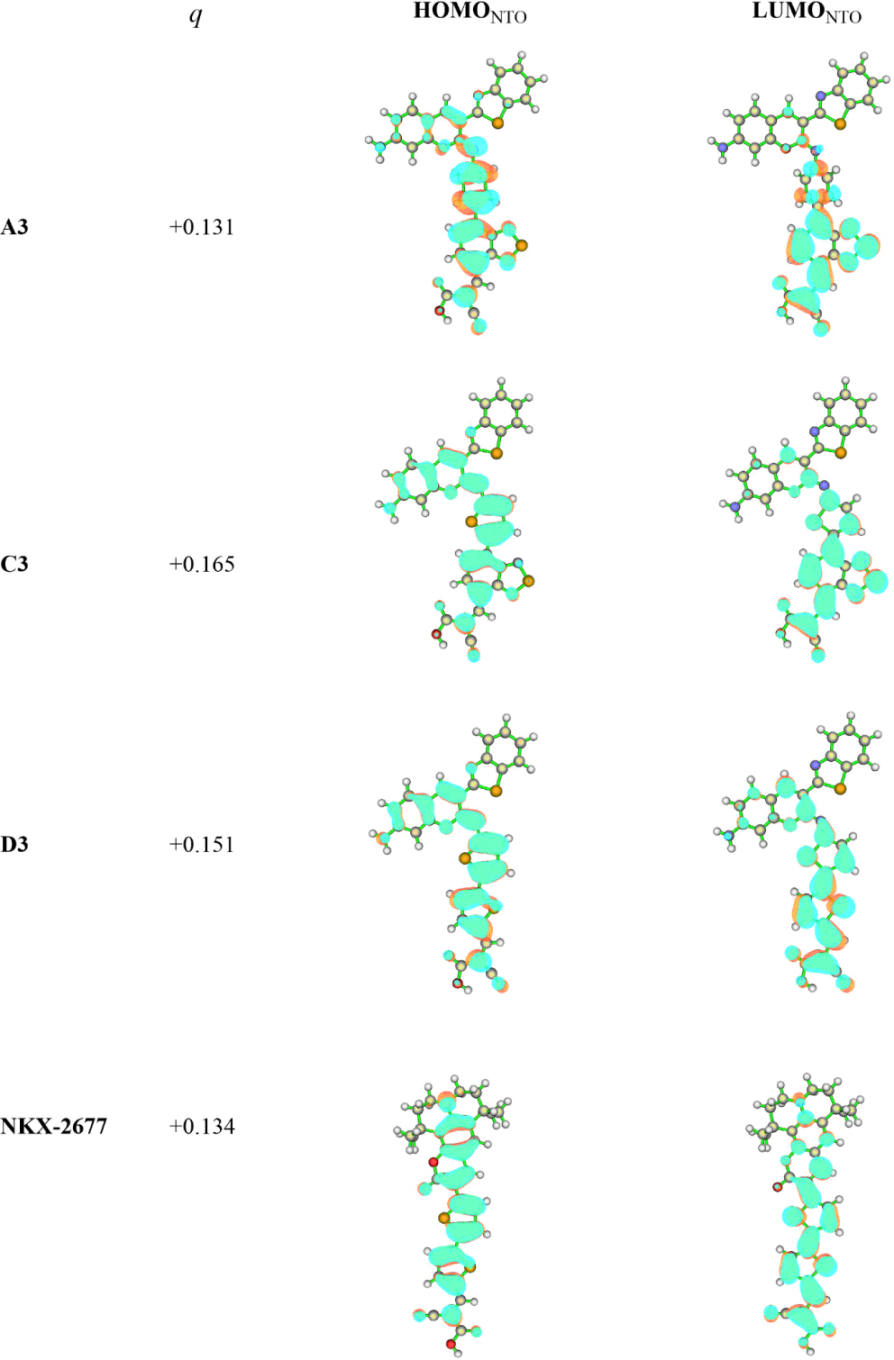
Hirshfeld density partition net electronic population (*q*) change at the “2-cyanoacrylic acid”-like acceptor moiety for the S_0_ → S_1_ photoexcitation and natural transition orbitals (NTO) for the S_0_ → S_1_ photoexcitation in dyes **A3**, **C3**, and **D3** compared to the **NKX-2677** reference dye. Contour value = 0.01500. Colors of the atoms: sulfur (yellow), oxygen (red), nitrogen (blue), carbon (gray), hydrogen (white).

Predictably, for all three of the proposed dyes, the *hole* was localized at the coumarin scaffold, while the *electron* was localized at the electron acceptor and π-linker moieties. As for the S_0_ → S_1_ interfragmentary Hirshfeld density partition calculations for the **A3**, **C3**, and **D3**, the “2-cyanoacrylic acid”-like acceptor moiety (see Figure 1c) showed promisingly similar net population increases of 0.131, 0.165, and 0.151, respectively, comparable to those expected for the **NKX-2677** reference dye (0.134).

To confirm the reliability of the computed photophysical and charge-transfer trends, we performed a minimal methodological sensitivity analysis. Calculations were conducted using alternative functionals (CAM-B3LYP and ωB97X-D) and basis sets (6-31+G­(d,p) and def2-TZVP) for representative dyes **A3**, **C3**, and **D3**. The results of this analysis are provided in S13 of the Supporting Information (Table S8) and confirm that the qualitative trends remain consistent across methods.

Finally, due to the promising properties of the proposed dye sensitizers—including intense visible-light absorption, tunable absorption profiles, and an expected high degree of charge separation—the thermodynamic requirements for their application as sensitizers in a Grätzel cell were assessed relative to the **NKX-2677** standard.

### 3.5 Thermodynamics for Electronic Injection, Dye Regeneration, and Photoinduced Electronic Transfer (PET)

A simple existential condition for any type of heterojunction solar cell is that the injection of electrons from the photoexcited sensitizer (dye) material to the photoanode (Scheme 1a, reaction 1b) must be a thermodynamically favorable process. As such, it is a necessary condition for the LUMO of the dye (*E* ^dye^_LUMO_) to be more positive in energy than the conduction band energy of the semiconductor material (*E* ^TiO2^_CB_), thus leading to a favorable electron injection driving force (Δ*G*_inj_ < 0), as seen in eq 9:^70^

9 $$\Delta G_{\mathrm{inj}}={E^{\mathrm{TiO2}}}_{\mathrm{CB}}-{E^{\mathrm{dye}}}_{\mathrm{LUMO}}$$

Another existential condition is for the spontaneous regeneration of the dye by the cell electrolyte (Scheme 1a, reaction 1d). For that, it is necessary that the sensitizer HOMO energy (*E*_HOMO_) be more negative than the electrolyte’s redox potential (herein, we considered triiodide/iodide *I*_3_^–^/*I* ^–^, for which *E*_redox_ = −4.85 ^87,88^ ), thus providing a favorable cell regeneration driving force (Δ*G*_regen_), as described in eq 10:^89^

10 $$\Delta G_{\mathrm{regen}}=E_{\mathrm{HOMO}}-E_{\mathrm{Redox}}$$

A useful predictor of a less favorable rate of electronic recombination (and thus, the loss of efficiency in the cell) is a more unfavorable free energy of recombination (Δ*G*_rec_), indicated by smaller values of Δ*G*_rec_ as provided by eq 11:^89,90^

11 $$\Delta G_{\mathrm{rec}}={E^{\mathrm{TiO2}}}_{\mathrm{CB}}-E_{\mathrm{HOMO}}$$

To assess the thermodynamics under device conditions, the global minima geometries for dyes **A1**–**5, C1**–**5**, and **D1**–**5** were optimized at the CAM-B3LYP/6-31+G­(d,p) level of theory by employing the IEFPCM implicit solvation simulating the acetonitrile solvent. Then, simulations of the vertical excitations employing TD-DFT at the same level of theory were carried out. The calculated thermodynamic descriptors of electron injection, cell regeneration, and free energy of electronic recombination, as well as the wavelength and oscillator strengths associated with the S_0_ → S_1_ (for all cases, λ_max_) in the presence of the implicit solvent, and frontier molecular orbital energies (FMO), are reported in Table 6. The simulated spectra for the evaluated dyes are available in Section S10 of the Supporting Information file (SI).

**Table 6:** TD-DFT: CAM-B3LYP/6-31+G­(d,p) Employing the IEFPCM Model (Acetonitrile as Solvent) Estimations of λ_max_ and Oscillator Strength for the Proposed Dye Sensitizers, as well as Frontier Molecular Orbital Energies, Electron Injection Driving Force (Δ*G*_inj_), Cell Regeneration Driving Force (Δ*G*_regen_), and Free Energy of Recombination (Δ*G*_rec_)

	λ_max_ (nm)	*f*	*E*_HOMO_ (eV)	*E*_LUMO_ (eV)	Δ*G*_inj_ (eV)	Δ*G*_regen_ (eV)	Δ*G*_rec_ (eV)
**A1**	423	1.2042	–7.2	–2.4	–1.7	–2.4	3.1
**A2**	425	1.1980	–7.1	–2.4	–1.7	–2.3	3.0
**A3**	434	1.0541	–6.8	–2.4	–1.7	–2.0	2.7
**A4**	417	1.1849	–7.4	–2.4	–1.7	–2.6	3.3
**A5**	419	1.1384	–7.4	–2.4	–1.7	–2.6	3.3
**C1**	524	1.1908	–6.8	–2.5	–1.6	–2.0	2.7
**C2**	531	1.1741	–6.7	–2.5	–1.6	–1.9	2.6
**C3**	550	1.1203	–6.6	–2.5	–1.6	–1.8	2.5
**C4**	514	1.1644	–6.9	–2.6	–1.5	–2.1	2.8
**C5**	515	1.0290	–7.0	–2.6	–1.5	–2.2	2.9
**D1**	474	1.4767	–6.8	–2.1	–2.0	–2.0	2.7
**D2**	479	1.4598	–6.7	–2.1	–2.0	–1.9	2.6
**D3**	493	1.3793	–6.5	–2.1	–2.0	–1.7	2.4
**D5**	478	1.0690	–6.9	–2.4	–1.7	–2.1	2.8
NKX-2677	482	2.0294	–6.4	–2.0	–2.1	–1.6	2.3

Overall, our computed values indicate that the proposed dyes fulfill the thermodynamic requirements for spontaneous electronic injection toward the TiO_2_ photoanode, as well as for the spontaneous dye regeneration in the presence of the triiodide/iodide electrolyte (as indicated by negative values for both Δ*G*_inj_ and Δ*G*_regen_), and comparable free energy of recombination (Δ*G*_rec_) to the reference dye—with dyes **A3**, **C3**, and **D3** presenting comparable performance. Promisingly, the presence of the solvent has not altered the observed trends related to the employment of EDG/EWG substituents at position 7 of the coumarin scaffold to promote more *red-shifted* absorption and more *blue-shifted* absorption, respectively.

Finally, it is important to emphasize that this work focuses on molecular-level electronic descriptors in gas and solution phases to establish structure–property relationships within the proposed dye series. Explicit dye–TiO_2_ interfacial modeling and excited-state dynamical analyses—such as adsorption motifs, surface-induced shifts, reorganization energies, and electronic coupling—were not included, as they require solid-state or multiscale computational frameworks beyond the scope of this study. The conclusions should therefore be interpreted within the molecular design context.

## 4 Conclusions

In this study, an *in-silico* assessment of iminocoumarin D-π-A photosensitizers was performed, revealing that π-linker flexibility and planarity strongly control visible-light absorption through HOMO–LUMO gap narrowing and photoinduced π-electron delocalization. When benchmarked against the reference dye **NKX-2677**, the proposed dyes show diverse structure–property trade-offs. Along the λ_max_ axis, at CAM-B3LYP/6-31+G­(d,p), and consistent with the values computed with other computational levels, **C3** exhibits the largest red-shift (λ_max_ = 520 nm), surpassing both **A3** (427 nm) and **D3** (457 nm), although **NKX-2677** (441 nm) remains competitive. Regarding light-harvesting efficiency (LHE), **D3** (94.4%, *f* ≈ 1.24) most closely approaches **NKX-2677** (98.7%, *f* ≈ 1.88), whereas **A3** (89.3%, *f* ≈ 0.971) and **C3** (90%, *f* ≈ 0.999) show moderate efficiency. In terms of charge-transfer metrics, **A3** displays the strongest charge-separation signature (Δμ ≈ 28.5 au, Λ ≈ 0.43, Δ*r* ≈ 6.78 Å), outperforming both **D3** (Δμ ≈ 10.8; Λ ≈ 0.60; Δ*r* ≈ 4.30 Å) and **C3** (Δμ ≈ 20.82; Λ ≈ 0.54; Δ*r* ≈ 4.82 Å). Thermodynamically, **A3**, **C3**, and **D3** all show favorable electron-injection driving forces (Δ*G*_inj_ ≈ −1.7, −1.6 and −2.0, respectively), while **D3** exhibits the lowest recombination driving force (Δ*G*_rec_ ≈ 2.4 eV), approaching **NKX-2677** (2.3 eV).

Overall, the ranking across these four axes positions **D3** as the closest performance analogue to **NKX-2677** due to its strong LHE, favorable λ_max_, and balanced ICT/thermodynamics; **A3** as the dye with the most pronounced charge separation (largest Δμ and Δ*r*); and **C3** as the dye offering the largest red-shift tunability despite moderate LHE. These results reinforce TDM-based screening and conformational sampling as key strategies in the rational design of iminocoumarin photosensitizers.

## Supplementary Material



## Notes

(i) CAM-B3LYP/6-31+G­(d,p) compared absorption and experimental data for dye NKX-2677 both in gas and condensed phases; (ii) example of a step-by-step calculation of the molecular planarity parameter (MPP); (iii) Boltmann population-weighted absorption spectra for the proposed dye series and discussion; (iv) Kohn–Sham frontier molecular orbital energies for all the evaluated dyes; (v) data on the simulated emission spectra; (vi) ecomposition of electric and transition dipole moment vectors for the proposed dyes; (vii) assessment of the IEFPCM (acetonitrile solvent) absorption spectra; (viii) assessment of the photophysical profile for alternative E-stereoisomer for the herein proposed dyes; (ix) the Cartesian coordinates for all computed geometries (ground-state and first singlet excited states) at the CAM-B3LYP/6-31+G­(d,p) level of theory, as well as their respective calculated electronic energy, zero-point energy and thermodynamic coordinates at a standard state of 298.15 K and 1 mol L^–1^ (PDF)
