# Supplementary material for: Design of Novel Iminocoumarins for D‑π‑A System DSSCs: A (TD)DFT Study
Source: ACS Omega. 2026 Jan 28;11(5):7713–28. doi: 10.1021/acsomega.5c09063 (PMC12902854; doi:10.1021/acsomega.5c09063)
Supplement: Supplementary file 1 [file ao5c09063_si_001.pdf]

Supporting Information (SI) for "Design of Novel Iminocoumarins for D-π-A System DSSCs: A (TD)DFT Study"

Patrick L. L. Rocha,[1,†] Patrick de L. Barbosa,[1,†] Amanda de A. Borges,[1] Edson Evangelista,[1] Isabela S. de Almeida,[1] Luana da S. M. Forezi, [1,*] Rodolfo G. Fiorot[1,*]

Department of Organic Chemistry, Institute of Chemistry, Universidade Federal Fluminense – UFF, Niteroi, Rio de Janeiro, 24020-141, Brazil.

[†] Both authors equally contributed to the work.

*Corresponding authors: Rodolfo Goetze Fiorot – rodolfofiorot@id.uff.br; Luana da S. M. Forezi – luanaforezi@id.uff.br.

Summary

S1. General operating mechanism behind DSSCs……………..……………………………….....**3**

S2. CAM-B3LYP/6-31+G(d,p) absorption spectrum for the NKX-2677 reference dye.............**4**

S3. Planarity diagram of NKX2677……………..…………………………………..……..…..**5**

S4. Boltzmann-weighed absorption spectra………………………..…………………..……...**7**

S5. S_0 Kohn-Sham frontier molecular orbital energies and *conceptual* DFT descriptors (CDFT)……………………………………………………………………………….…....…**13**

S6. Emission spectra expected for the proposed dye sensitizers……………………...………....**15**

S7. Series A and D assessment of the first singlet excited state...……………………………………………….……….....**16**

S8. LOL-π function map for the *o*-substituted B2-Me dye sensitizer……………..……….......**17**

S9. Electric and transition dipole moment vectors (*x*,*y*,*z*) composition………..………..….....**18**

S10. Absorption spectra of the proposed dyes in gas phase and employing the IEFPCM implicit solvation model with acetonitrile as solvent….…...**21**

S11. Photophysical profile of the alternative (*E*)-isomers of the proposed A3, C3 and D3 dyes……...………………………………………………………………………………....**23**

S12. Computed gas phase geometries and energies at the CAM-B3LYP/6-31+G(d,p) level…………………………..…………………………………………………………....**24**

S13. Method variation………………………..……………………………………………**25**

S1. General operating mechanism behind DSSCs

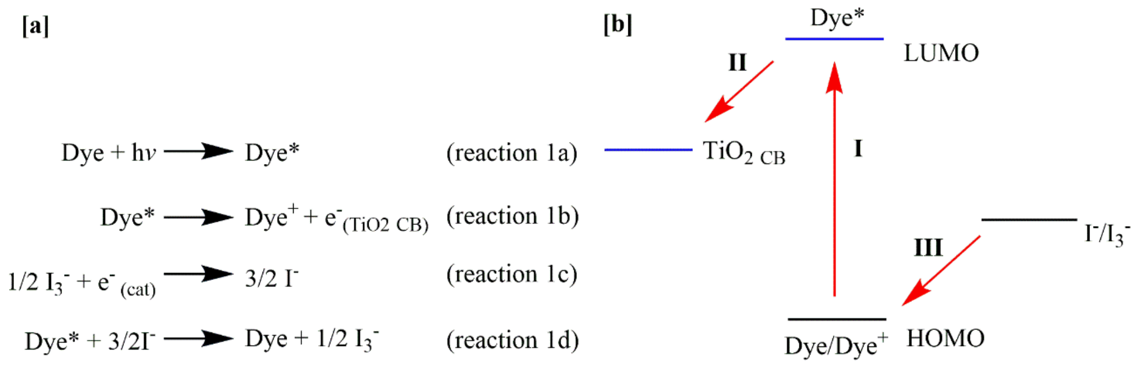

Scheme S1. a) Mechanism of a standard Grätzel cell. Reaction. 1a Corresponds to the photoexcitation process; Reaction. 1b corresponds to the transfer of electrons (injection) from the LUMO orbital of the photoexcited dye towards the conduction band (CB) of the TiO_2 semiconductor; Reaction. 1c corresponds to the reduction of the triiodide electrolyte by the cell cathode (usually, platinum), and Reaction. 1d, the oxidation of the iodide ion back to triiodide and dye regeneration. b) Schematic diagram of electron transfer processes involving the dye sensitizer, where I represents photoexcitation, II represents electronic injection towards the semiconductor conduction band, and III represents the regeneration of the oxidized dye.

As illustrated in **Scheme 1b**, a dye (photosensitizer) for the DSSC must necessarily present i. a LUMO with more positive (higher) energy than the reduction potential of the conduction band of the TiO_2 semiconductor (herein adopted as – 4.1 eV, as to account for the possible slight shifts in the semiconductor conduction band energy due to the possible dissociative and undissociated dye adsorptions)[1] allowing for electron injection, ii. a HOMO with more negative (lower) energy than the oxidation potential of the electrolyte (I^-/I_3^-) acting as a reducing agent, allowing dye regeneration, iii. absorption in the visible region of the electromagnetic spectrum with expressive oscillator strength / high molar absorbance coefficient parameters, and iv. an anchoring unit that binds the dye to the semi-porous TiO_2 semiconductor film and promotes charge transfer from the photoexcited dye towards the photoanode, often presenting hydroxyl and carboxyl groups.[2–5]

S2. CAM-B3LYP/6-31+G(d,p) absorption spectrum for the NKX-2677 reference dye

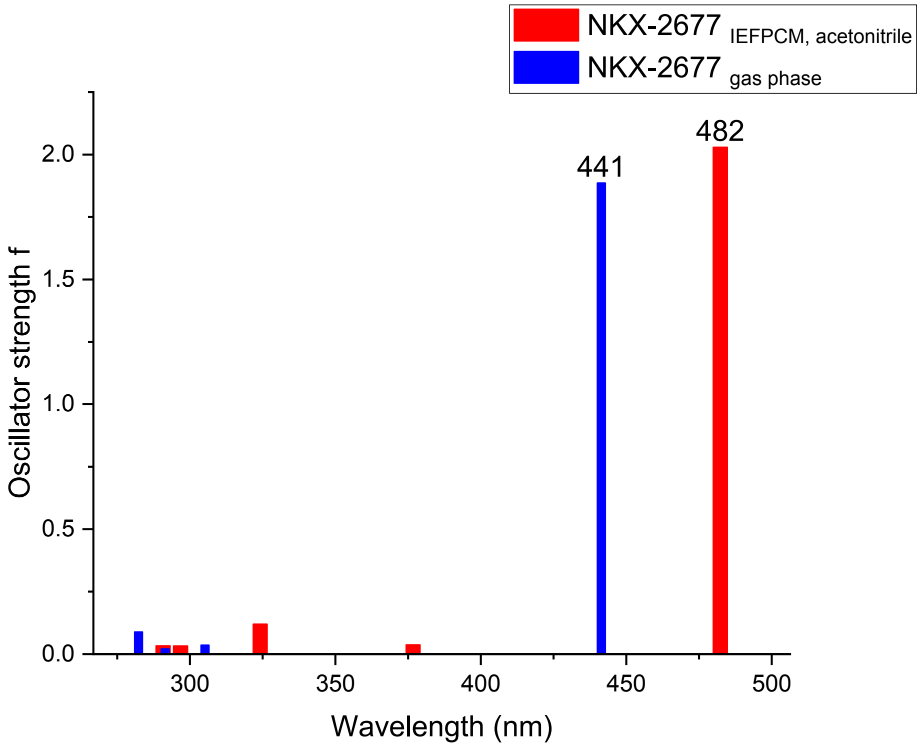

Figure S1. Simulated CAM-B3LYP/6-31+G(d,p) absorption spectra, both gas phase and employing the IEFPCM[6] solvation model with acetonitrile as solvent. Experimental absorption wavelength in a solution containing solvents *t*-BuOH-Acetonitrile 50:50 (wt %) was reported as 511 nm,[7] a relative error of 6% for the DFT: CAM-B3LYP/6-31+G(d,p) theoretical calculations.

The distinction to the observed experimental absorption peak at 511 nm for a mixture of solvents *t*-BuOH-Acetonitrile 50:50 (wt %), measured by Hara and and co-workers, could be both a result of important *red shift* promoted by hydrogen bonding moieties between solute and solvent molecules, as well as CAM-B3LYP's trend to consistently overestimate excitation energies when combined with basis sets consisting of few diffuse and polarizable functions added to Pople's 6-31G set.[7] A relative error of 6% was found comparing theoretical and experimental spectra (**Fig. S1**) once the IEFPCM solvation model was employed to simulate the acetonitrile solvent due to the overestimation of the excitation energy for the first excited state. Therefore, for the present work, it is expected for the gas phase CAM-B3LYP/6-31+G(d,p) to overestimate excitation energies in general. Yet, the CAM-B3LYP functional serves as a vital tool given the goal of the work herein not to necessarily reproduce and research the spectra of the renowned NKX series (for which there are more appropriate methodologies in the literature)[8–10], but to characterize the herein novel imino-coumarin molecules as potential dyes for a varying set of substituents and π-linker, where we seek to assess the nature of visible light absorption for these systems in terms of spatial orbital overlap — for which the CAM-B3LYP functional provides effectives threshold through which photoexcitations can be classified as Rydberg-, internal charge transfer (ICT) and local excitation.[11]

Additionally, the absorption spectrum for the (*Z*)-isomer of the NKX-2677 (both gas phase and condensed phase, simulated through the IEFPCM solvation model) were simulated to assess any difference in the absorption profile of different stereoisomers of the acetonitrile anchoring unit. Barely any difference in the photophysical profile between (*E*)-NKX-2677 (the reference dye) and its (*Z*)-isomer is expected, with the reported gas phase λmax for the $S_0 \rightarrow S_1$ photoexcitation at 443 nm (*f* = 1.946) and acetonitrile-solvated λ_{max} for the $S_0 \rightarrow S_1$ photoexcitation at 482 nm.

S3. Planarity diagram of NKX2677

For the global minimum NKX2677 geometry (**Fig S2**), the fitting plane is defined as:

$$0.00609x - 0.00576y + 0.9996z + 0.00740 = 0 \quad \textbf{(Eq. S1)}$$

Where (0.00609, – 0.00576, 0.9996) correspond to the normal vector to the fitting plane identified through the *single-value decomposition method* (SVD).

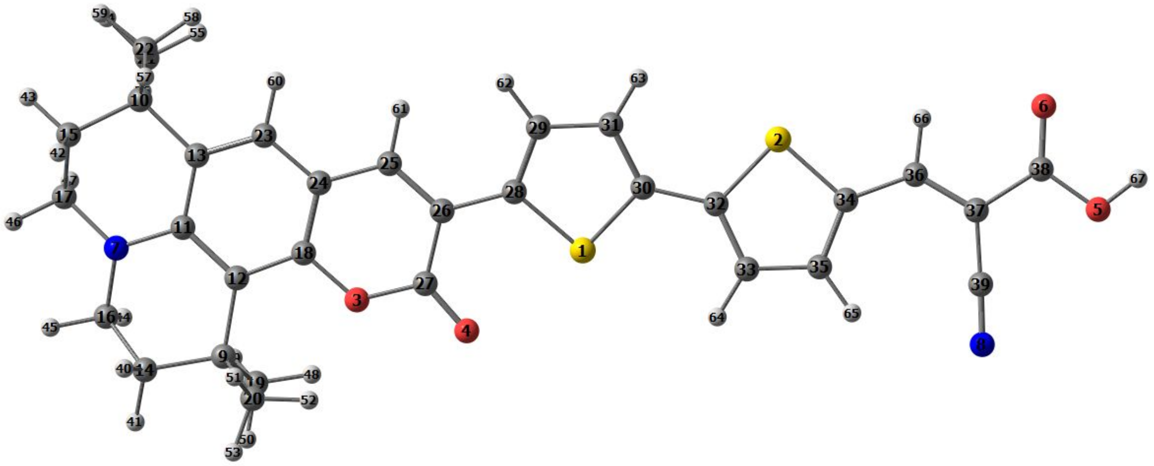

Figure S2. Standard labelling of the NKX-2677 molecule for MPP (Molecular Planarity Parameter) analysis.

The distances of each atom *i* ($d_i = |D + ax_0 + by_0 + cz_0|/[a + b + c]^{1/2}$) to the fitting plane were calculated as:

d: 1(S) 0.02174 Angstrom
d: 2(S) 0.10081 Angstrom
d: 3(O) -0.04767 Angstrom
d: 4(O) 0.00342 Angstrom
d: 5(O) 0.27481 Angstrom
d: 6(O) 0.38714 Angstrom
d: 7(N) 0.08553 Angstrom
d: 8(N) -0.04647 Angstrom

d: 9(C) -0.12160 Angstrom
d: 10(C) -0.03500 Angstrom
d: 11(C) -0.01185 Angstrom
d: 12(C) -0.07289 Angstrom
d: 13(C) -0.06315 Angstrom
d: 14(C) -0.33855 Angstrom
d: 15(C) -0.36685 Angstrom
d: 16(C) 0.47927 Angstrom
d: 17(C) 0.40133 Angstrom
d: 18(C) -0.07703 Angstrom
d: 19(C) 1.19560 Angstrom
d: 20(C) -1.30356 Angstrom
d: 21(C) 1.36046 Angstrom
d: 22(C) -1.07872 Angstrom
d: 23(C) -0.10315 Angstrom
d: 24(C) -0.09559 Angstrom
d: 25(C) -0.11396 Angstrom
d: 26(C) -0.11094 Angstrom
d: 27(C) -0.05020 Angstrom
d: 28(C) -0.15812 Angstrom
d: 29(C) -0.33229 Angstrom
d: 30(C) -0.13393 Angstrom
d: 31(C) -0.31775 Angstrom
d: 32(C) -0.08112 Angstrom
d: 33(C) -0.15665 Angstrom
d: 34(C) 0.05762 Angstrom
d: 35(C) -0.07851 Angstrom
d: 36(C) 0.16135 Angstrom
d: 37(C) 0.16144 Angstrom
d: 38(C) 0.28576 Angstrom
d: 39(C) 0.04800 Angstrom
d: 40(H) -1.39536 Angstrom
d: 41(H) -0.09161 Angstrom
d: 42(H) -1.43866 Angstrom
d: 43(H) -0.14570 Angstrom
d: 44(H) 1.55861 Angstrom
d: 45(H) 0.29703 Angstrom
d: 46(H) 0.12163 Angstrom
d: 47(H) 1.48581 Angstrom
d: 48(H) 1.38202 Angstrom
d: 49(H) 2.05208 Angstrom
d: 50(H) 1.14046 Angstrom
d: 51(H) -2.24063 Angstrom
d: 52(H) -1.16586 Angstrom

d: 53(H) -1.40460 Angstrom
d: 54(H) 1.39239 Angstrom
d: 55(H) 1.60415 Angstrom
d: 56(H) 2.14603 Angstrom
d: 57(H) -2.07745 Angstrom
d: 58(H) -0.84629 Angstrom
d: 59(H) -1.10858 Angstrom
d: 60(H) -0.12955 Angstrom
d: 61(H) -0.13056 Angstrom
d: 62(H) -0.48215 Angstrom
d: 63(H) -0.45935 Angstrom
d: 64(H) -0.28002 Angstrom
d: 65(H) -0.12813 Angstrom
d: 66(H) 0.25569 Angstrom
d: 67(H) 0.35988 Angstrom

The Molecular Planarity Parameter (MPP) would be evaluated as the root-mean square deviation for the 67 (N_{atoms}), to be determined as:

$$\mathrm{MPP} = \sqrt[2]{\frac{1}{67}\sum_{i=1}^{67} di^2} \quad \textbf{(Eq. S2)}$$

The CAM-B3LYP/6-31+G(d,p) evaluated molecular planarity parameter (MPP) for NKX-2677 only populated conformer is 0.85 Angstrom.

S4. Boltzmann-weighed absorption spectra

If thermodynamic equilibrium is assumed for all the different accessible conformers of a molecule, the experimentally observed absorption spectra must necessarily correspond to a Boltzmann population-weighted average spectrum of the populated conformers at a specific temperature T (**Equation S1**). [12,13]

$$P_i = \exp(-(\Delta G_i - \Delta G_{min})/RT)/[\textstyle\sum_j \exp(-(\Delta G_j - \Delta G_{min})/RT] \qquad (1)$$

P_i = Boltzmann population (as a decimal) of a conformer *i* at temperature T;
ΔG_i = Thermally corrected Gibbs free energy of a conformer *i* at temperature T;
ΔG_{min} = Thermally corrected Gibbs free energy of the ensemble minimum geometry (the global minimum) at temperature T;
ΔG_j = Thermally corrected Gibbs free energy of ensemble conformers *j* at temperature T;
R = Universal gas constant.

The complete gallery for the evaluated conformers of dyes **A1-D1**, as well as the associated molecular planarity parameter (MPP) and the relative Gibbs free energy at a standard state (298.15 K, 1 M) is available at **Figure S3**.

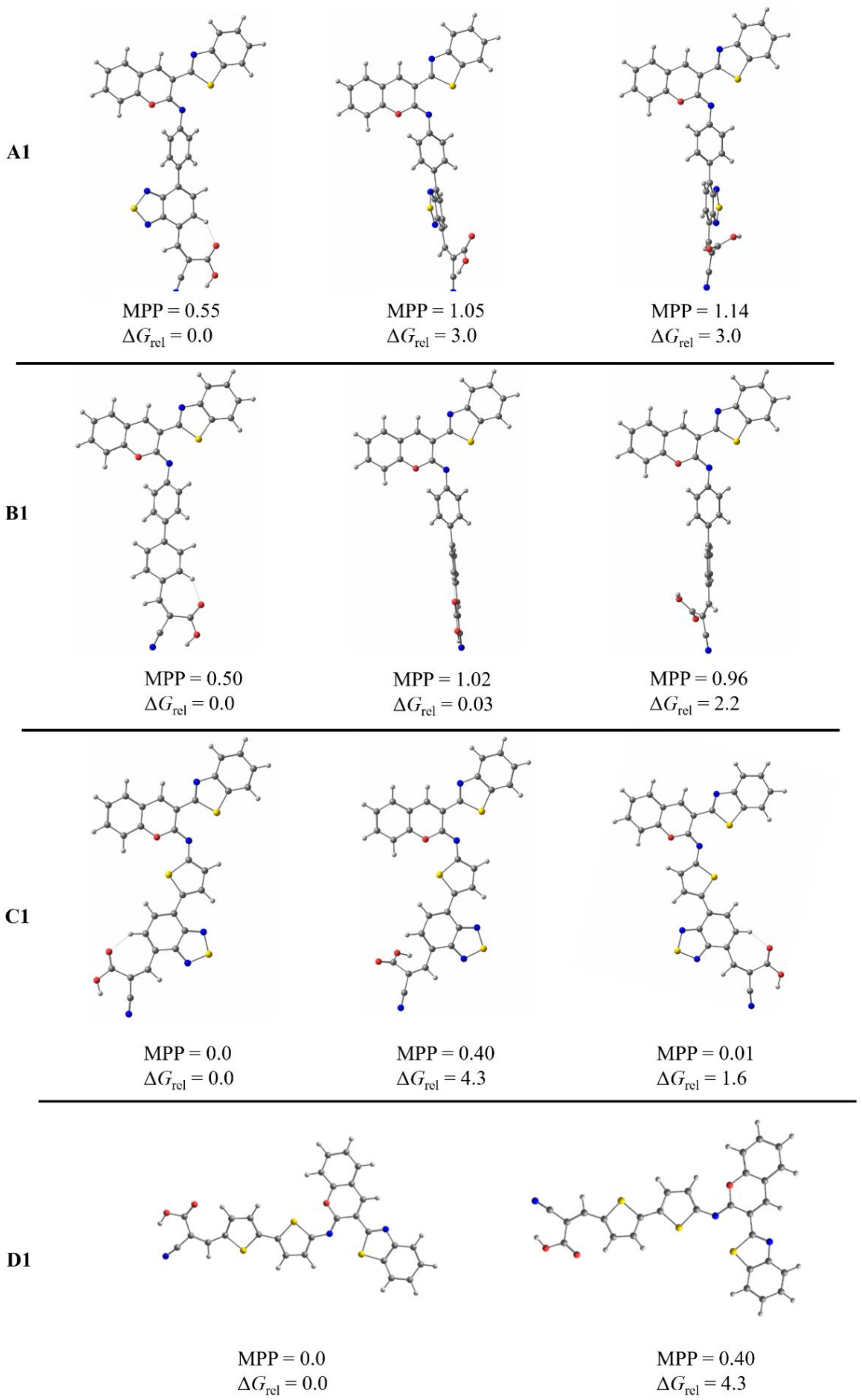

Figure S3. Gallery of conformers for dye sensitizers (**A-D**)1 and associated molecular planarity parameter.

The following plots represent the comparison of the absorption spectra of different conformers (herein identified in accordance to the measured MPP) of the proposed **A-D1** dyes with the Boltzmann population-weighted spectrum:

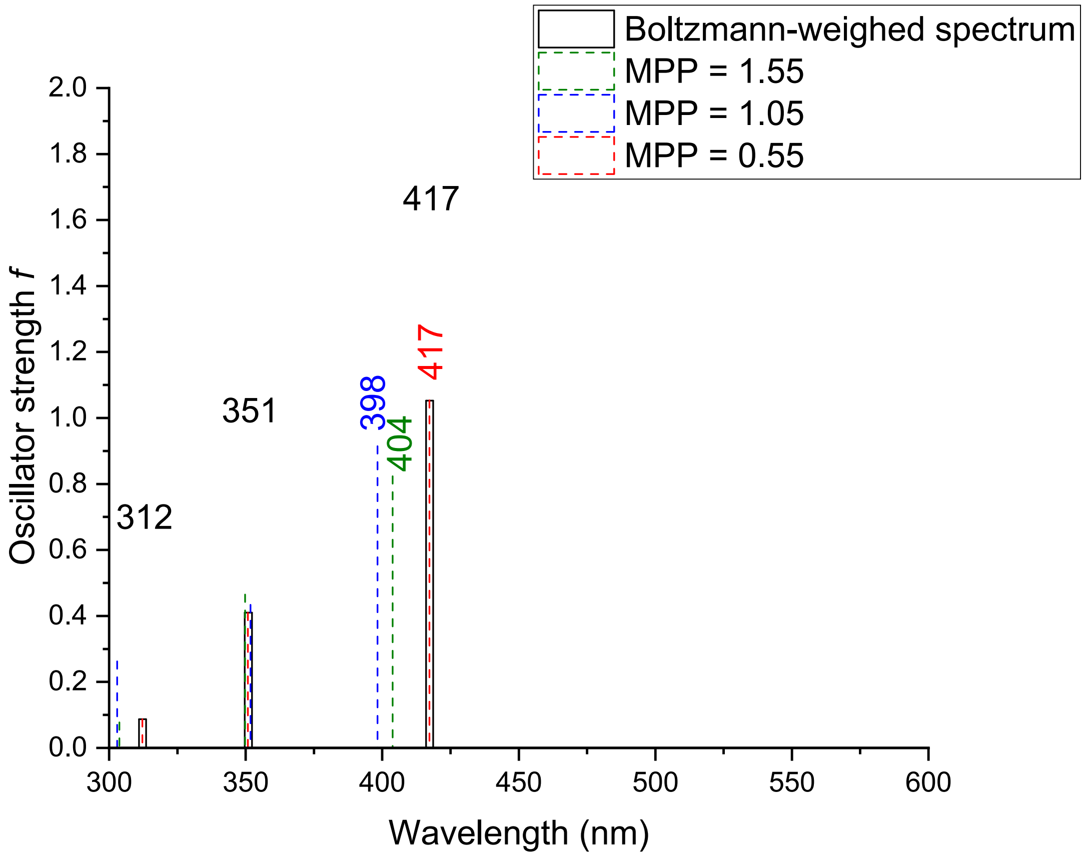

Fig. S4. Boltzmann-weighed absorption spectrum displayed as a transparent bar with 95% spacing for the **A1** dye, while conformer absorption bands correspond to colourful dotted lines. Black labels refer to the Boltzmann-weighted absorption band.

No significant discrepancy (beyond 5%) was found between the Boltzmann-weighed absorption spectrum and the global minima geometry (MPP =0.55) absorption profile.

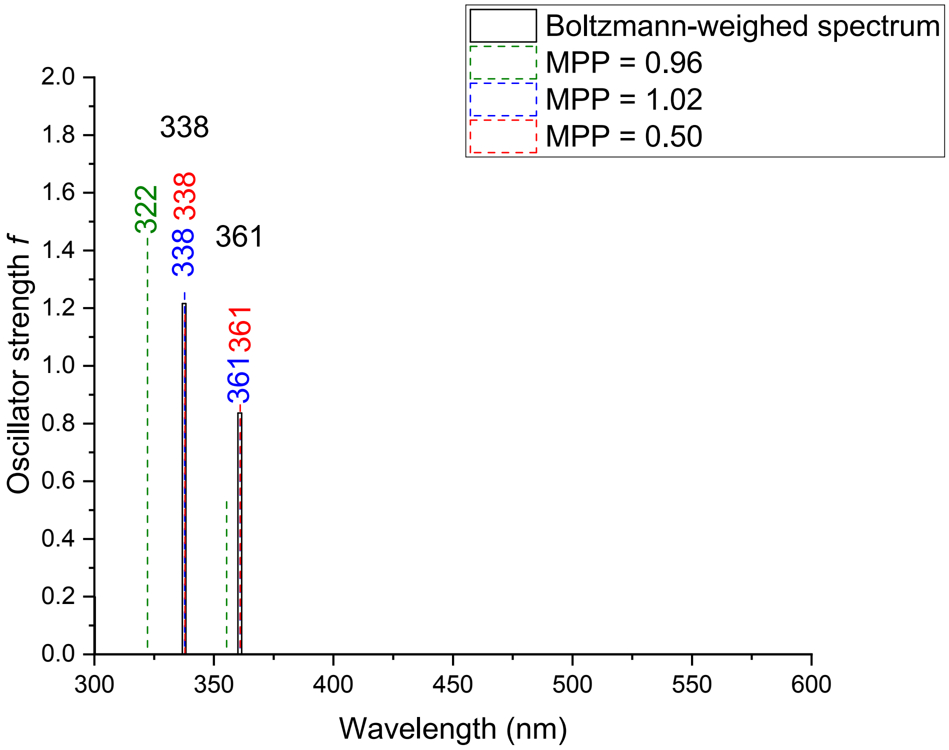

Fig. S5. Boltzmann-weighed absorption spectrum displayed as a bar with 95% spacing for the **B1** dye, while conformer absorption bands correspond to colourful dotted lines. Black labels refer to the Boltzmann-weighted absorption band.

Again, no significant discrepancy (above 5%) was found between the Boltzmann-weighed absorption spectrum and the global minima geometry (MPP =0.50) absorption profile. However, sharp discrepancies began to appear for the least populated geometries.

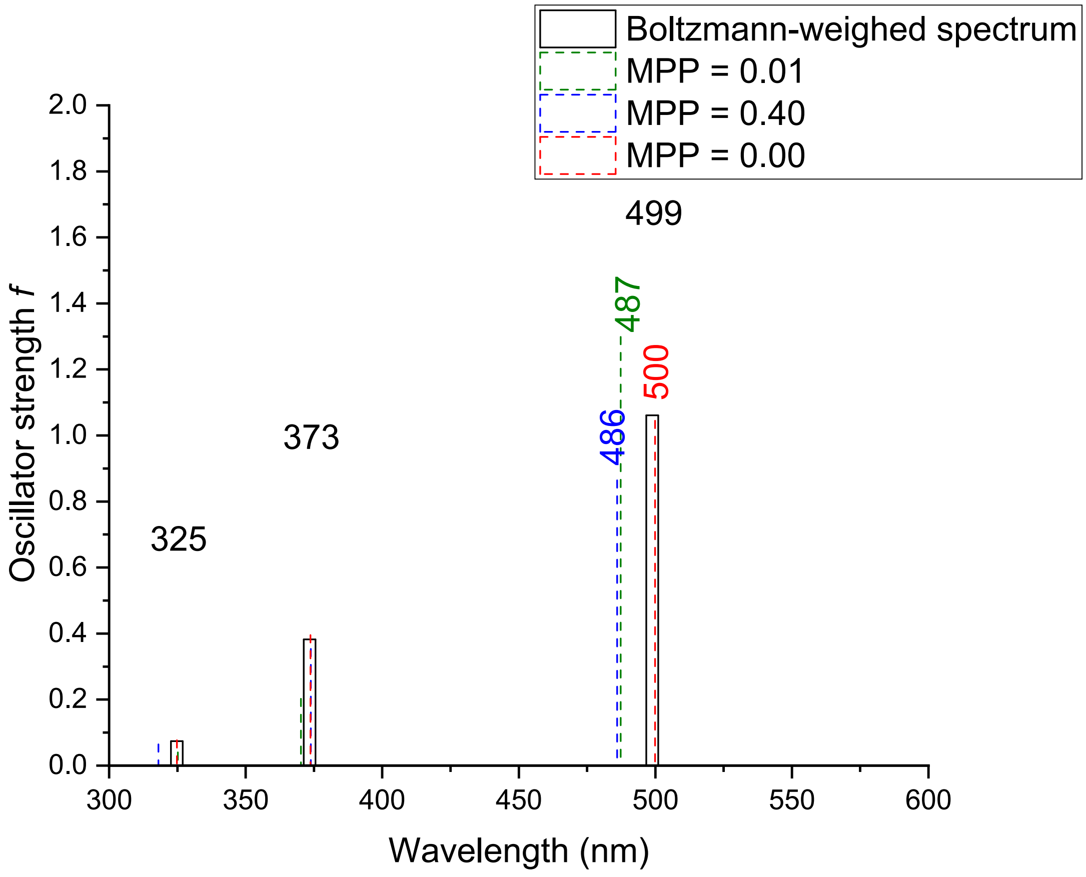

Fig. S6. Boltzmann-weighed absorption spectrum displayed as a bar with 95% spacing for the **C1** dye, while conformer absorption bands correspond to colourful dotted lines. Black labels refer to the Boltzmann-weighted absorption band.

Again, no significant discrepancy (above 5%) was found between the Boltzmann-weighed absorption spectrum and the global minima geometry (MPP =0.55) absorption profile. However, the C2 rotation around the N-C bond is demonstrated to be sufficient for a considerable error in relation to the Boltzmann-weighted spectrum, justifying the importance of performing analysis over global minima geometries of the potential energy surface.

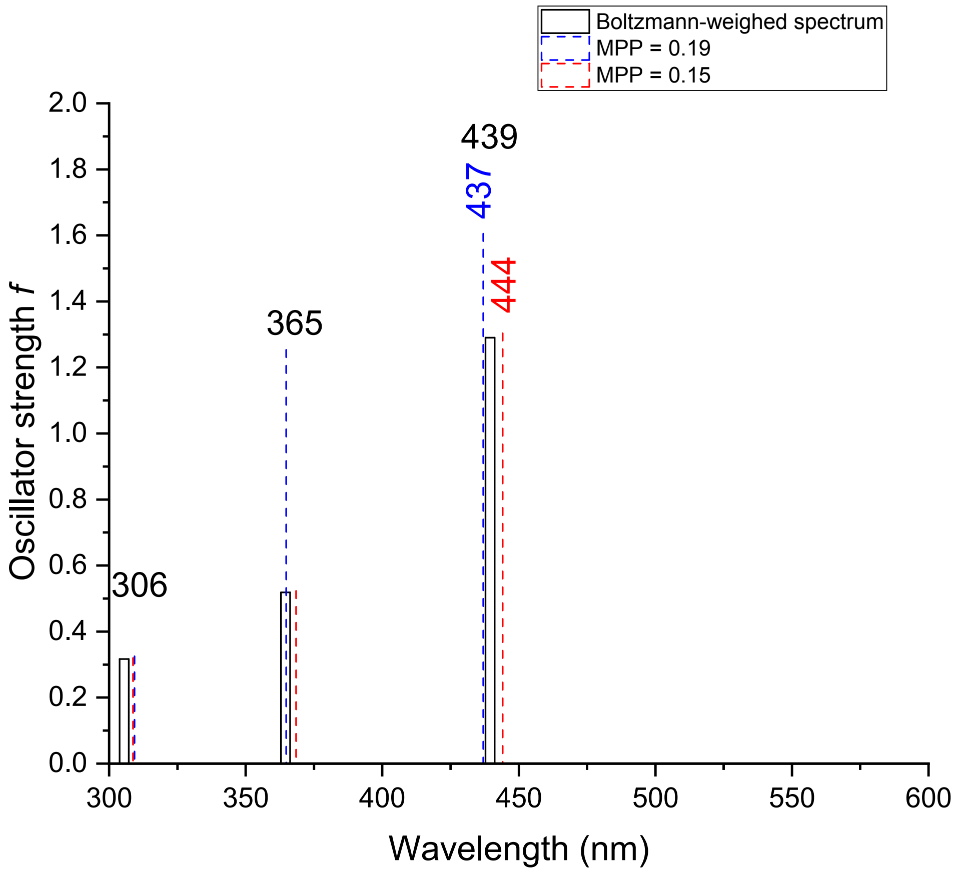

Fig. S7. Boltzmann-weighed absorption spectrum displayed as a bar with 95% spacing for the **D1** dye, while conformer absorption bands correspond to colourful dotted lines. Black labels refer to the Boltzmann-weighted absorption band. An absolute error inferior to 1% was detected between the Boltzmann-weighted spectrum and the the global minimum (MPP = 0.19).

S5. S_0 Kohn-Sham frontier molecular orbital energies and *conceptual* DFT descriptors (CDFT)

Table S1. Ground-state DFT: CAM-B3LYP/6-31+G(d,p) Kohn-Sham frontier molecular orbital energies (FMO) for every proposed dye sensitizer.

A series					
	**A1**	**A2**	**A3**	**A4**	**A5**
E_{HOMO-1} (eV)	-7.8	-7.7	-7.5	-7.9	-8.0
E_{HOMO} (eV)	-7.3	-7.1	-6.9	-7.5	-7.5
E_{LUMO} (eV)	-2.4	-2.4	-2.4	-2.5	-2.5
E_{LUMO+1} (eV)	-1.6	-1.4	-1.3	-1.9	-2.4
**$\Delta E_{HOMO-LUMO}$** (eV)	4.8	4.7	4.5	5.0	5.0
**B series**					
	**B1**	**B2**	**B3**	**B4**	**B5**
E_{HOMO-1} (eV)	-7.8	-7.7	-7.6	-7.9	-8.0
E_{HOMO} (eV)	-7.3	-7.1	-6.9	-7.5	-7.5
E_{LUMO} (eV)	-1.7	-1.9	-1.9	-2.0	-2.5
E_{LUMO+1} (eV)	-1.6	-1.4	-1.3	-1.8	-1.9
**$\Delta E_{HOMO-LUMO}$** (eV)	5.6	5.3	5.0	5.5	5.1
**C series**					
	**C1**	**C2**	**C3**	**C4**	**C5**
E_{HOMO-1} (eV)	-7.9	-7.7	-7.5	-8.0	-8.1
E_{HOMO} (eV)	-6.9	-6.8	-6.7	-7.1	-7.2
E_{LUMO} (eV)	-2.6	-2.5	-2.5	-2.6	-2.7
E_{LUMO+1} (eV)	-1.7	-1.6	-1.4	-2.0	-2.4
**$\Delta E_{HOMO-LUMO}$** (eV)	4.3	4.3	4.2	4.4	4.4
**D series**					
	**D1**	**D2**	**D3**	**D4**[a]	**D5**
E_{HOMO-1} (eV)	-7.8	-7.7	-7.5	---	-8.1
E_{HOMO} (eV)	-6.9	-6.8	-6.7		-7.1
E_{LUMO} (eV)	-2.2	-2.1	-2.1		-2.6
E_{LUMO+1} (eV)	-1.7	-1.5	-1.4		-2.1
**$\Delta E_{HOMO-LUMO}$** (eV)	4.7	4.7	4.6		4.5

[a] – Geometry failed to converge at the CAM-B3LYP/6-31+G(d,p).

Given the frontier molecular orbital energies in **Table 1**, it is possible to calculate the *conceptual* DFT (CDFT) descriptors.[14] Firstly, the ionization potential (I) and the electron affinity (A) corresponds to – E_{HOMO} and – E_{LUMO}, respectively. The electrochemical potential (μ) corresponds then to approximately:

$$\mu = -(I + A)/2 \text{ (Eq. S3)}$$

Analogue to the electrochemical potential is the Mulliken electronegativity (χ), corresponding to $-\mu$. Similarly, the chemical hardness (h) can be approximated as:

$$h = (I - A)/2 \text{ (Eq. S4)}$$

And chemical softness (S) can be calculated as the inverse of chemical hardness:

$$S = n^{-1} \text{ (Eq. S5)}$$

The electrophilicity index (ω) can be calculated employing both electrochemical potential and chemical hardness, consisting in:

$$\omega = -(\mu^2)/(2h) \text{ (Eq. S6)}$$

As for the nucleophilicity index (N) of a compound, it was measured upon the TCE tetracyanoethylene (TCE) scale employing the gas-phase EHOMO for TCE at the same level of theory (E_{HOMO} TCE):

$$N = E_{HOMO} - (E_{HOMO}\text{ TCE}) \text{ (Eq. S7)}$$

<u>**S6**. Emission spectra expected for the proposed dye sensitizers</u>

(A1)

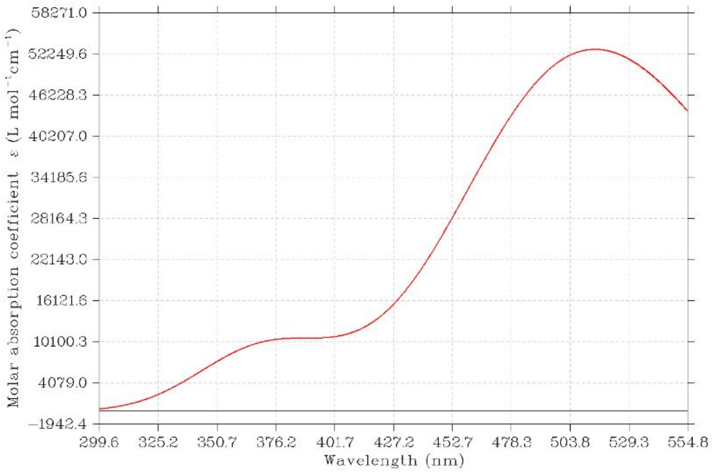

(C1)

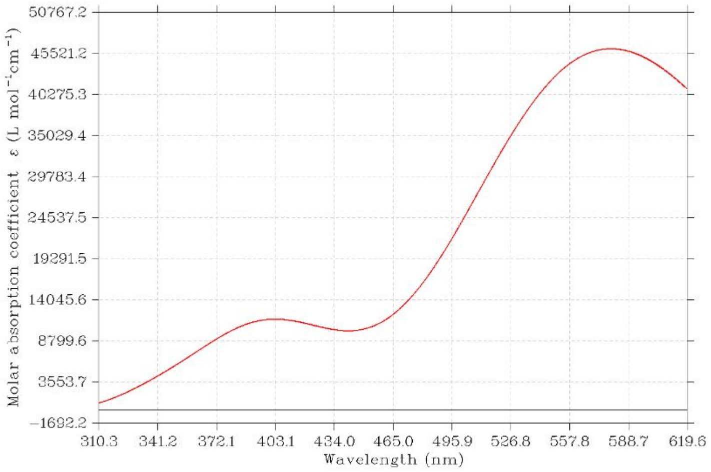

(D1)

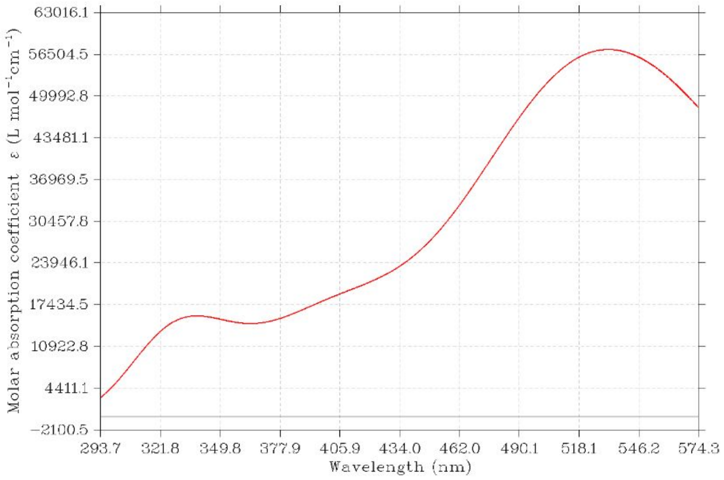

Fig. S8. Emission spectra for the **A1**, **C1** and **D1** dyes.

S7. Series A and D first singlet excited state assessment

Table S2. Series **A** first singlet excited state maximum emission wavelength (CAM-B3LYP/6-31+G(d,p)) reported stokes shift, associated oscillator strength and molecular planarity parameter (MPP).

	-R	Emission $\lambda_{S1\rightarrow S0}$ (nm)	Stokes Shift	f	Molecular planarity parameter (Å)
**A1**	-H	516	99	1.3078	0.44
**A2**	-OH	520	101	1.3317	0.40
**A3**	$-NH_2$	529	102	1.2814	0.38
**A4**	$-CF_3$	508	99	1.3156	0.51
**A5**	$-NO_2$	555	138	0.689	0.20

A notable stokes shift is expected for series, which are also expected to present intense absorption of visible light.

Table S3. Series **D** first singlet excited state maximum emission wavelength (CAM-B3LYP/6-31+G(d,p)) reported stokes shift, associated oscillator strength and molecular planarity parameter (MPP).

	-R	Emission $\lambda_{S1\rightarrow S0}$ (nm)	Stokes Shift	f	Molecular planarity parameter (Å)
**D1**	-H	547	103	1.418	0.00
**D2**	-OH	552	106	1.453	0.00
**D3**	$-NH_2$	568	111	1.431	0.02
**D4**[a]	$-CF_3$			---	
**D5**	$-NO_2$	643	189	0.562	0.00

[a] – Ground-state geometry failed to converge at the DFT: CAM-B3LYP/6-31+G(d,p) level.

MPP at ground-state equilibrium geometries had been measured as 0.20 Å, indicating a planarization of the dye geometry upon photoexcitation. Similar results for series **A**, where the original MPP at the ground-state minimum vibrational level had been measured as around ~0.50 Å (see **Table S2**).

<u>**S8**. LOL-π function map for the *o*-substituted B2-Me dye sensitizer</u>

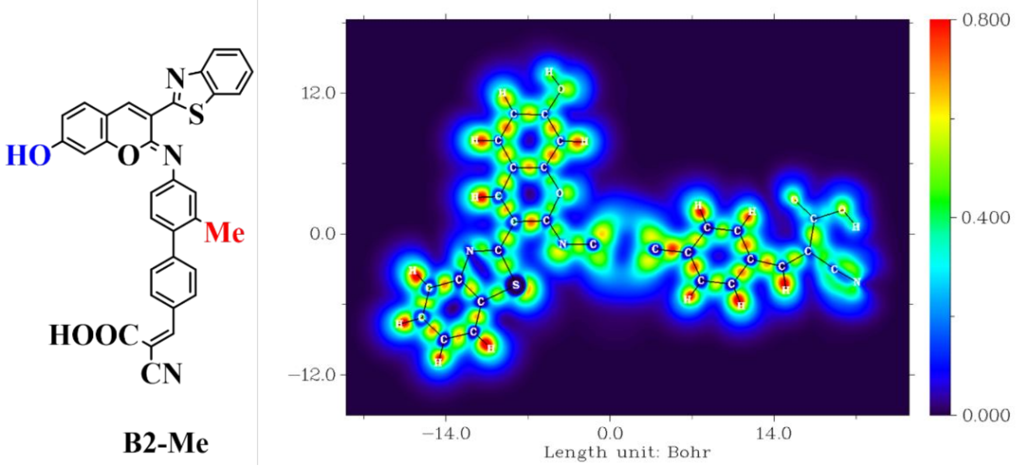

Figure S9. CAM-B3LYP/6-31+G(d,p) color-filled local orbital locator-*π* (LOL-*π*) maps employing Becke's composition analysis method for the test **B2-Me** dye. The cartesian coordinates matrix for the referred system are available at the final section of the **SI** file.

S9. Electric and transition dipole moment vectors (*x*,*y*,*z*) composition

Table S4. CAM-B3LYP/6-31+G(d,p) composition of the electric dipole moment vectors at the ground-state (S_0) and first singlet excited state (S_1).

	S_0			S_1		
	μ_x	μ_y	μ_z	$\mu_{x'}$	$\mu_{y'}$	$\mu_{z'}$
**A3**	2.487091	1.666969	0.568743	7.691116	2.819655	0.449612
**B3**	2.906674	1.739852	-0.332800	6.187135	1.954285	-0.279101
**C3**	-4.880620	-2.120597	0.056154	-8.306173	-2.041723	-0.025853
**D3**	2.906674	1.739852	-0.332800	6.187135	1.954285	-0.279101
**NKX-2677**	-4.880620	-2.120597	0.056154	-8.306173	-2.041723	-0.025853

Table S5. CAM-B3LYP/6-31+G(d,p) composition of transition dipole moment vectors for the $S_0 \rightarrow S_1$ photoexcitation.

	$S_0 \rightarrow S_1$		
	μ_x	μ_y	μ_z
**A3**	3.6588767	0.5116733	0.1059871
**B3**	-4.1081162	-1.3858500	-0.0077587
**C3**	-4.0729380	0.7348563	-0.0082509
**D3**	-4.1081162	-1.3858500	-0.0077587
**NKX-2677**	-5.2346590	-0.2242755	-0.0029647

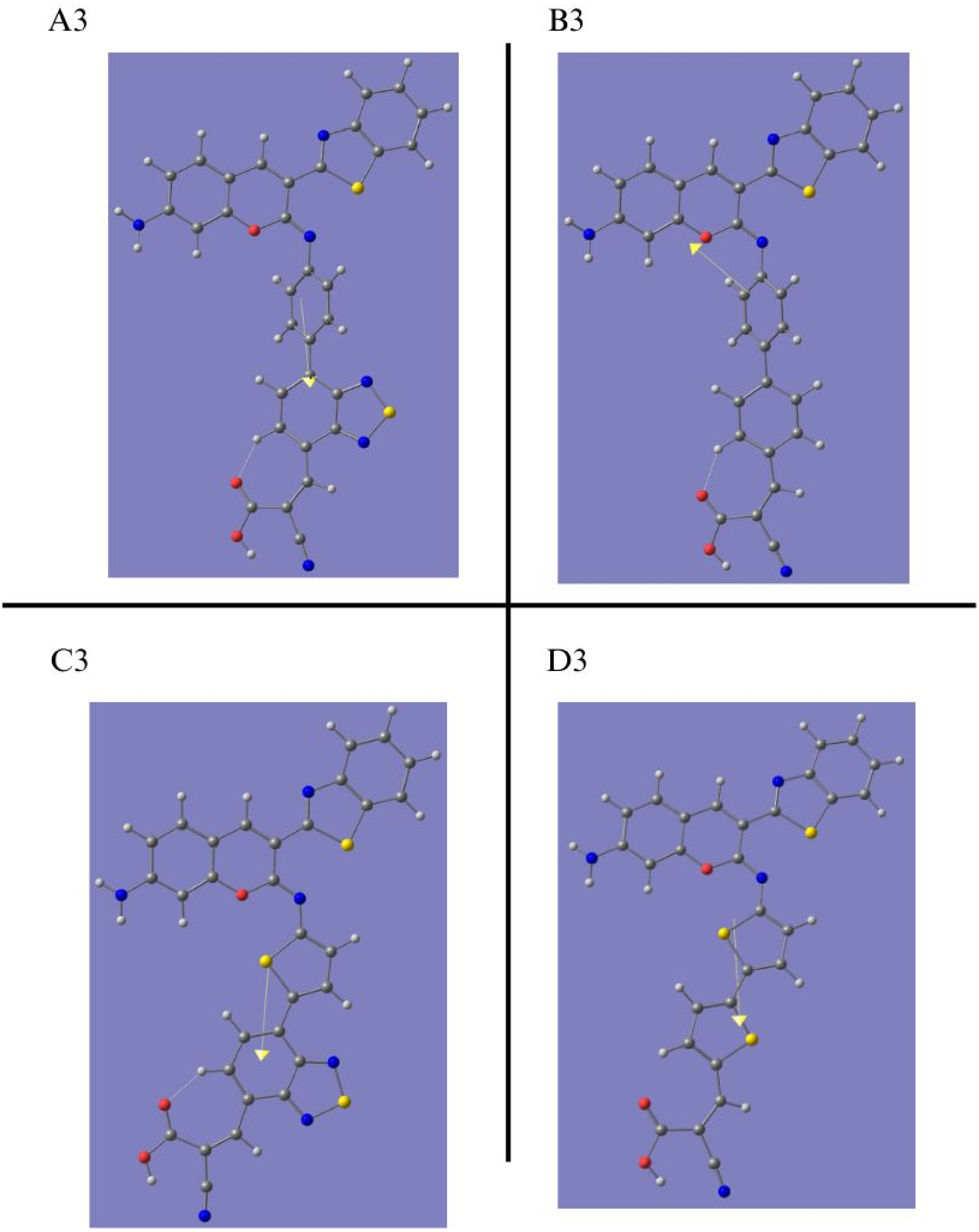

Fig S10. Represented transition dipole moment vector $\vec{\mu}$ for proposed dye sensitizers (**A-D**)**3**. The direction chosen to be indicated by the tip of the arrow represents the direction of the negative of the transition dipole moment vector.

NKX-2677

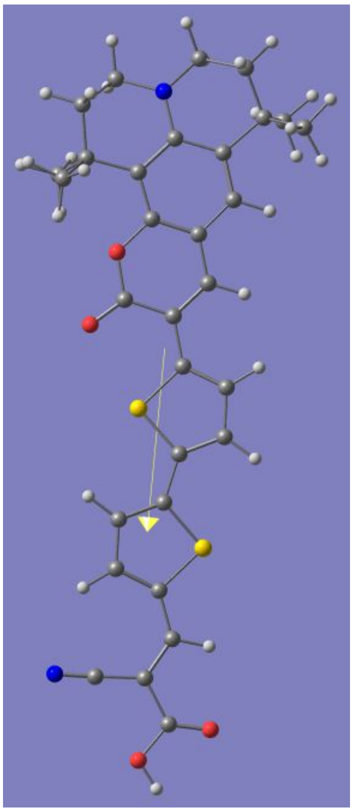

Fig S11. Represented transition dipole moment vector $\vec{\mu}$ for reference dye sensitizer **NKX-2677.** The direction chosen to be indicated by the tip of the arrow represents the direction of the negative of the transition dipole moment vector.

S10. Absorption spectra of the proposed dyes in gas phase and employing the IEFPCM implicit solvation model with acetonitrile as solvent

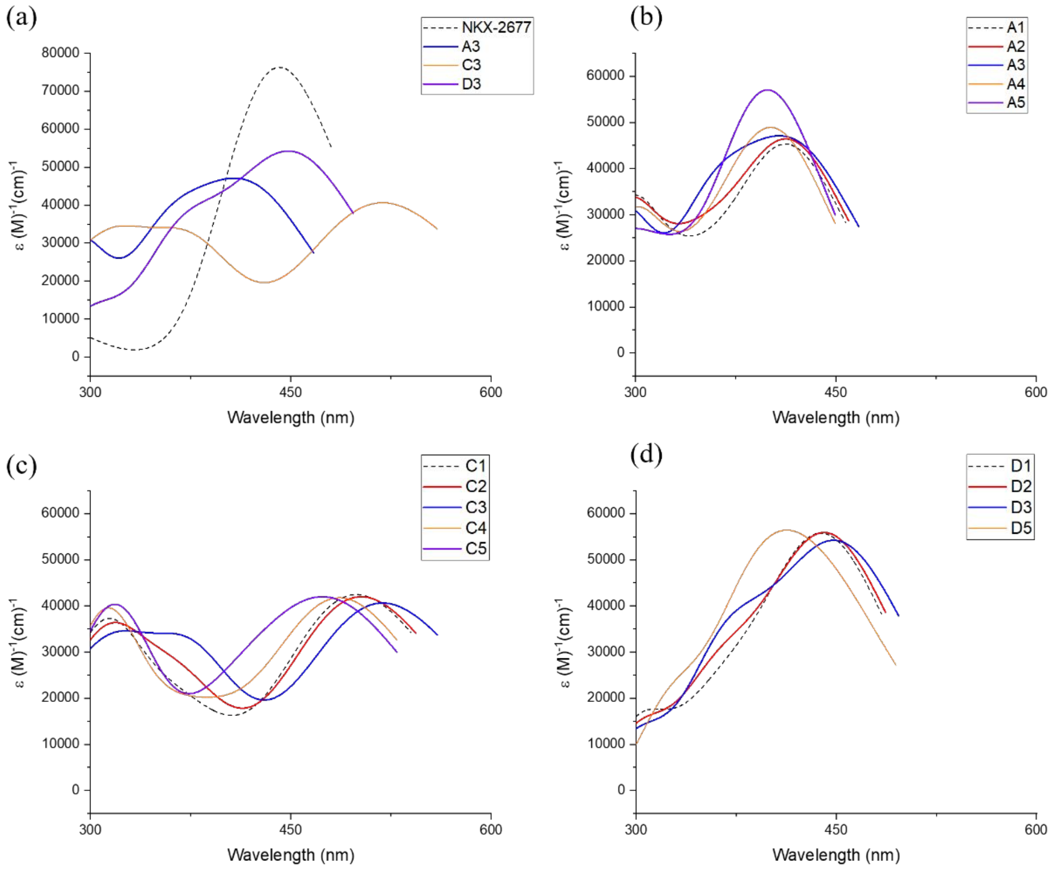

Figure S12. a) Comparison of the absorption spectra for **NKX-2677** reference dye *versus* the 7-amino-analogues on the proposed series (**A-D**)**3**. b)-d) Effect of different EDG/EWG substituents at position 7 of the coumarin for series **A**, **C** and **D**, respectively: 1, R = H; 2, R = OH; 3, R = NH_2; 4, R = CF_3; 5, R = NO_2. The dashed lines represent the R = H (**A-D**)**1** dye. We have employed a gaussian broadening function with full width half maximum (FWHM) at 0.66667 eV.

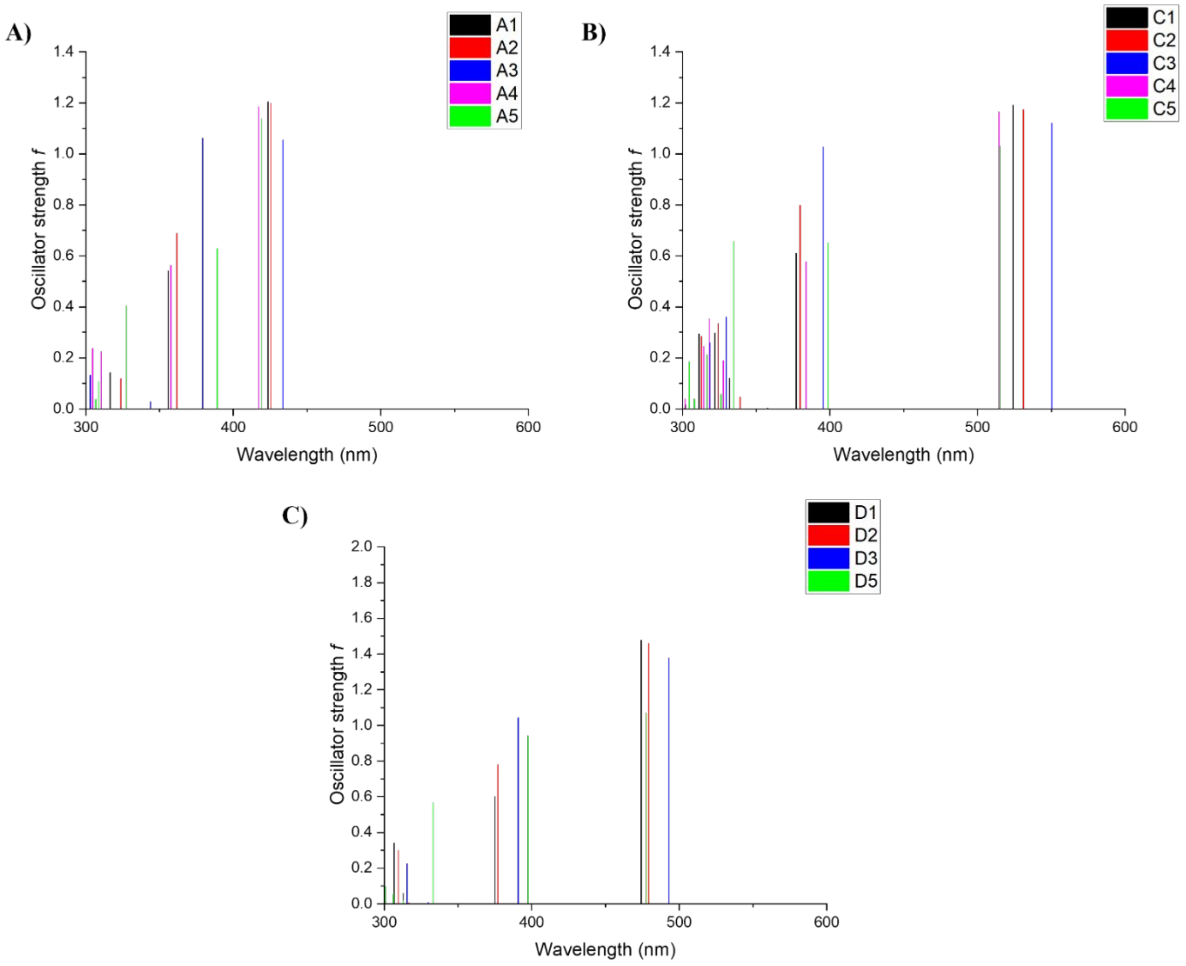

Figure S13. Simulated CAM-B3LYP/6-31+G(d,p) absorption spectra of the global minima equilibrium geometries for compounds from the A) series A; B) series C; C) series D derived from optimization calculations employing the IEFPCM[6] solvation model with acetonitrile as solvent. Similarly to gas-phase calculations, geometry optimization failed for the D4 dye at the CAM-B3LYP/6-31+G(d,p) due to convergence failure.

S11. Photophysical profile of the alternative (*E*)-isomers of the proposed A3, C3 and D3 dyes

Much alike the little to no difference in the absorption spectra for the *E*/*Z* stereoisomers in the acetonitrile anchoring unit of the **NKX-2677** (**Fig. S9)** was observed, the same similarity is expected for the alternative (*E*) stereoisomers of the proposed dyes **A3**, **C3** and **D3** (**Table S5**):

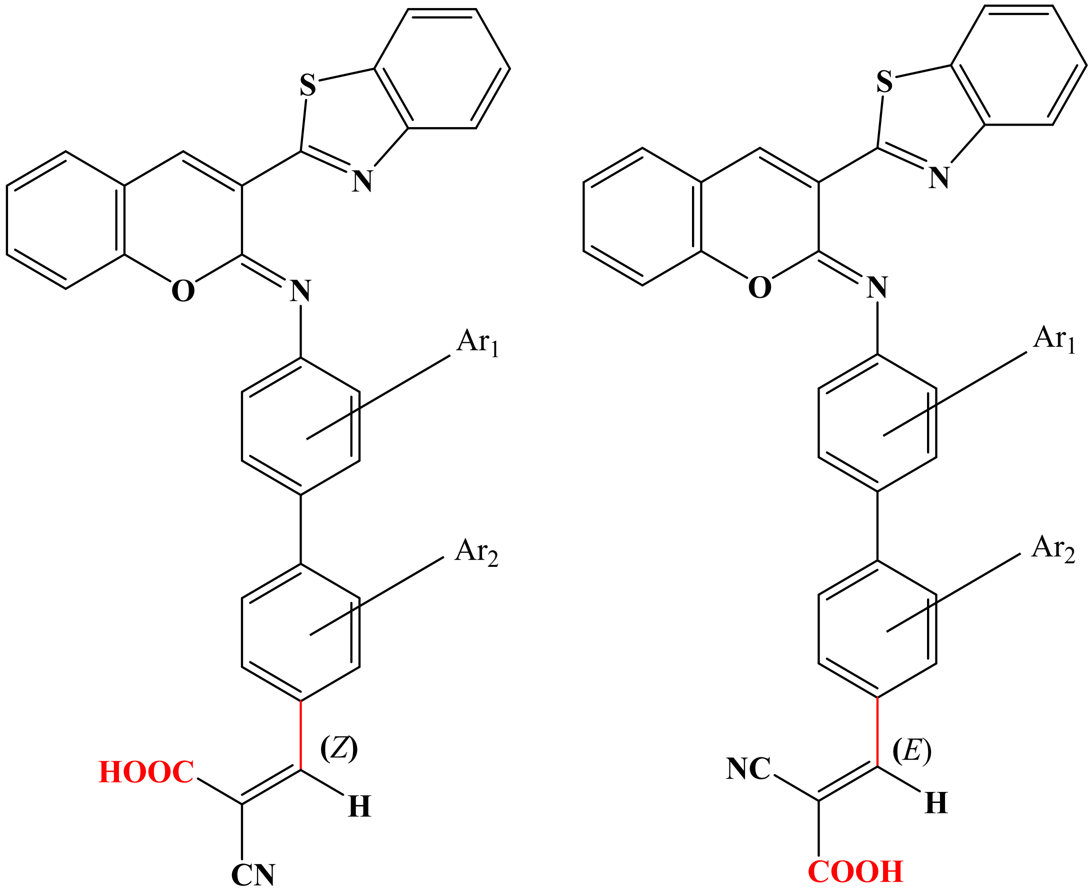

Fig. S14. Molecular structures for the *E*/*Z* stereochemistry in the acetonitrile anchoring unit. Left, the proposed (*Z*)-stereochemistry of dye series **A-D**. Right, the alternative herein reported (*E*)-stereochemistry.

Table S6. CAM-B3LYP/6-31+G(d,p) expected spectrum for the (*E*) stereoisomers for the proposed **A3**, **C3** and **D3** dyes. λ_{max} corresponds to the absorption band associated with the S0→S1 photoexcitation, with *f* representing the associated oscillator strength.

	λ_{max} (nm)	*f*
**(*E*)-A3**	429	0.8947
**(*E*)-C3**	521	0.9304
**(*E*)-D3**	454	1.1525

S12. Computed gas phase geometries and energies at the CAM-B3LYP/6-31+G(d,p) level

The following geometries were reported first as lowest energy conformers in their respective potential energy surfaces through the CREST (Conformer-Rotamer Ensemble Sampling Tool) protocol using the GFN2-xTB semi-empirical method, and then optimized at the CAM-B3LYP/6-31+G(d,p) level of theory in the gas phase, which confirmed them as the lowest energy, most populated conformers for each molecule. Underneath the cartesian coordinates for the CAM-B3LYP/6-31+G(d,p) gas-phase optimized geometries, the thermally-corrected Gibbs free energy (*G*) at a standard state of T = 298.15 K and mol L^{-1} is reported. *G* can be expressed as the sum:

$$G = E_{elec} + E_{vib} + E_{rot} + E_{trans} + ZPE + RT - TS \quad \textbf{(Eq. S8)}$$

E_{elec} = Internal energy due to electronic motion;
E_{vib} = Vibrational contribution to electronic energy;
E_{rot} = Rotational contribution to electronic energy;
E_{trans} = Translational contribution to electronic energy;
ZPE = Zero point energy correction;
TS = Total energetic contribution of entropy at 298.15 K;
R = Universal gas constant.

In the aforementioned expression, the reported thermally-corrected enthalpy (*H*) at a standard state can be described by the terms:

$$H = E_{elec} + E_{vib} + E_{rot} + E_{trans} + ZPE + RT \quad \textbf{(Eq. S9)}$$

Lastly, the reported E_{Total} term refers to the sum of the electronic energy at the optimized equilibrium distance at the CAM-B3LYP/6-31+G(d,p) level of theory plus the vibrational, rotational and translational energetic contributions:

$$E_{Total} = E_{elec} + E_{vib} + E_{rot} + E_{trans} \quad \textbf{(Eq. S10)}$$

All energies are provided in Hartree. Colour scheme for atoms: carbon (gray), hydrogen (white), nitrogen (blue), oxygen (red), fluorine (cyan) and sulfur (yellow).

S13. Method variation

Table S8. TD-DFT: vertical excitation wavelength (λ_{max}), electric dipole moment change $\Delta\mu$, charge transfer length Δr, hole and electron overlap index Λ obtained for different methods in gas phase.

		**CAM-B3LYP/6-31+G(d,p)**		
**Compound**	**λ_{max} (nm)**	**$\Delta\mu$**	**Λ**	**Δr (Å)**
**A3**	427	28.53	0.43	6.78
**C3**	520	20.82	0.54	4.82
**D3**	457	10.86	0.60	4.30
		**CAM-B3LYP/ def2-TZVP**		
**Compound**	**λ_{max} (nm)**	**$\Delta\mu$**	**Λ**	**Δr (Å)**
**A3**	414	25.16	0.44	6.50
**C3**	506	19.97	0.54	4.88
**D3**	450	9.41	0.61	4.14
		**ωB97X-D/6-31+G(d,p)**		
**Compound**	**λ_{max} (nm)**	**$\Delta\mu$**	**Λ**	**Δr (Å)**
**A3**	408	14.27	0.46	6.25
**C3**	498	14.39	0.53	4.99
**D3**	437	6.76	0.59	4.44
		**ωB97X-D/def2-TZVP**		
**Compound**	**λ_{max} (nm)**	**$\Delta\mu$**	**Λ**	**Δr (Å)**
**A3**	395	12.17	0.48	5.64
**C3**	486	13.56	0.53	5.02
**D3**	436	5.72	0.61	4.18

NKX 2677 (Hara and co-workers, 2005) reference dye sensitizer and the NKX series dyes

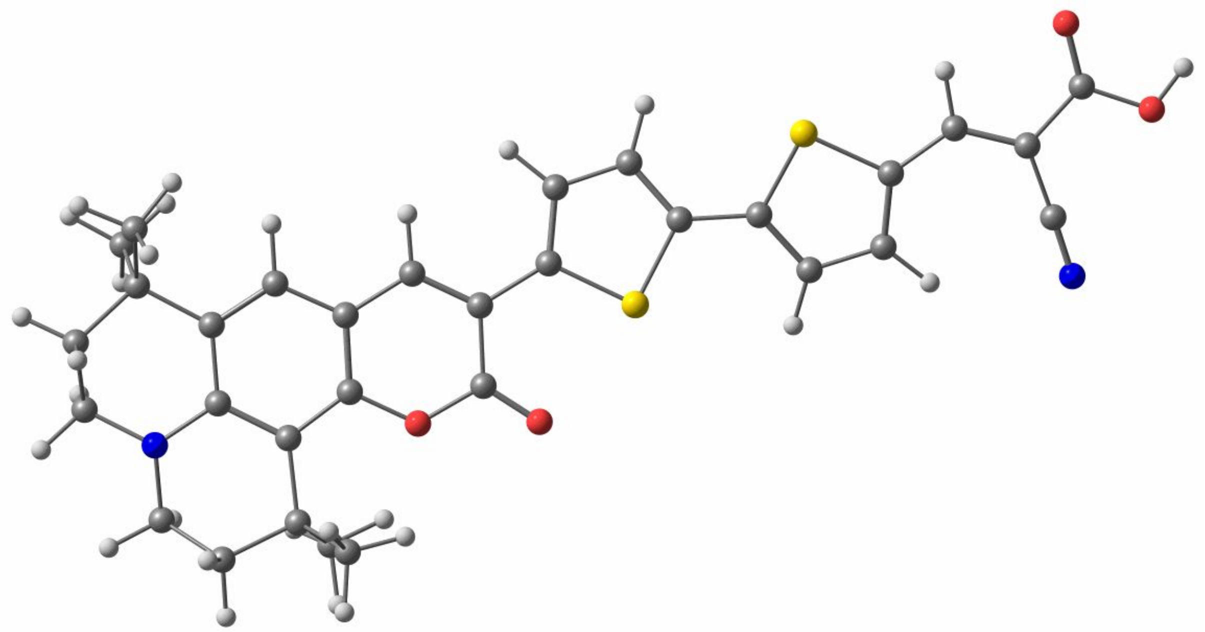

(*E*)-NKX-2677, reference coumarin dye sensitizer ground-state geometry

16	1.741437000	0.650801000	0.010964000
16	5.608742000	-1.395799000	0.054686000
8	-2.673445000	1.497659000	-0.026672000
8	-0.569086000	2.151389000	0.015375000
8	11.739360000	0.122637000	0.200093000
8	10.746393000	-1.886629000	0.306898000
7	-7.330102000	0.398693000	0.128572000
7	9.432961000	2.645779000	-0.092608000
6	-5.300101000	2.520660000	-0.078707000
6	-6.738656000	-2.473060000	-0.012116000
6	-6.005725000	0.020481000	0.020943000
6	-4.988918000	1.014390000	-0.040567000
6	-5.672128000	-1.372161000	-0.040427000
6	-6.809168000	2.723092000	-0.285298000
6	-8.107643000	-1.861950000	-0.332121000
6	-7.635893000	1.758062000	0.532018000
6	-8.347438000	-0.586457000	0.444898000
6	-3.669607000	0.562729000	-0.055353000
6	-4.870986000	3.193263000	1.239809000

6	-4.603988000	3.225325000	-1.260888000
6	-6.764945000	-3.133312000	1.379740000
6	-6.454670000	-3.558101000	-1.063861000
6	-4.345093000	-1.730933000	-0.090580000
6	-3.311164000	-0.787458000	-0.083876000
6	-1.929075000	-1.129845000	-0.112637000
6	-0.941513000	-0.192862000	-0.110235000
6	-1.333565000	1.213802000	-0.038994000
6	0.479796000	-0.531937000	-0.168034000
6	1.018560000	-1.786669000	-0.352726000
6	2.977176000	-0.556750000	-0.159195000
6	2.428847000	-1.803396000	-0.346872000
6	4.379012000	-0.194883000	-0.112844000
6	4.936390000	1.068092000	-0.184492000
6	6.881464000	-0.199187000	0.010634000
6	6.340179000	1.068291000	-0.114892000
6	8.235049000	-0.647770000	0.103533000
6	9.391080000	0.068716000	0.100720000
6	10.662875000	-0.680830000	0.212970000
6	9.434719000	1.490834000	-0.004797000
1	-7.060812000	2.574801000	-1.341471000
1	-7.068649000	3.755681000	-0.030816000
1	-8.165438000	-1.632613000	-1.402291000
1	-8.896702000	-2.588069000	-0.110341000
1	-7.458400000	1.895353000	1.611114000
1	-8.701957000	1.926292000	0.357235000
1	-9.316652000	-0.158245000	0.173563000
1	-8.379002000	-0.785346000	1.528468000
1	-3.802392000	3.068748000	1.419003000
1	-5.410014000	2.779955000	2.097221000
1	-5.081447000	4.267169000	1.192139000
1	-4.806681000	2.700556000	-2.199775000
1	-3.526229000	3.298177000	-1.129323000
1	-5.002329000	4.241269000	-1.353647000
1	-7.519765000	-3.926733000	1.411696000
1	-5.794761000	-3.579697000	1.614962000
1	-6.991133000	-2.413886000	2.170866000
1	-6.343616000	-3.122261000	-2.060791000
1	-5.550892000	-4.130166000	-0.840230000
1	-7.286767000	-4.269086000	-1.092749000
1	-4.072507000	-2.780664000	-0.124685000
1	-1.675563000	-2.185416000	-0.136870000
1	0.421737000	-2.677195000	-0.504087000
1	3.017218000	-2.701115000	-0.497232000

1	4.343402000	1.967182000	-0.299071000
1	6.939103000	1.967679000	-0.162983000
1	8.389186000	-1.721338000	0.190749000
1	12.523174000	-0.443872000	0.277131000

Eelec = -2404.204679
$H_{(298.15\ K)}$ = -2403.635368
$G_{(298.15\ K)}$ = -2403.741456
ZPE (Zero-point energy correction) = 0.533458

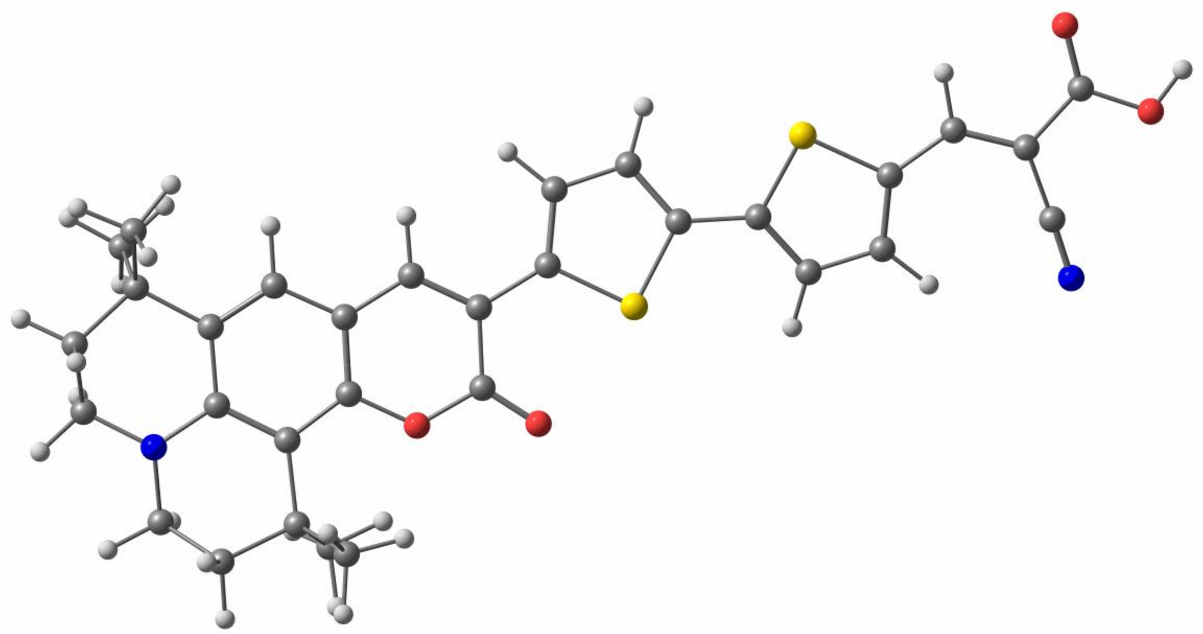

Acetonitrile-solvated (*E*)-NKX-2677 reference coumarin dye sensitizer ground-state geometry, solvent simulated through the IEFPCM model.

16	1.743640000	0.657870000	0.008711000
16	5.616200000	-1.409295000	0.046688000
8	-2.671396000	1.499757000	-0.006669000
8	-0.574280000	2.162428000	0.046117000
8	11.742114000	0.143024000	0.079093000
8	10.773068000	-1.881000000	0.148041000
7	-7.323857000	0.392819000	0.107771000
7	9.471402000	2.648868000	-0.058106000
6	-5.307562000	2.523229000	-0.099751000
6	-6.740210000	-2.473762000	-0.048763000
6	-6.008988000	0.020323000	0.001240000
6	-4.989629000	1.018764000	-0.051582000
6	-5.670207000	-1.376659000	-0.065241000
6	-6.813503000	2.717359000	-0.333650000
6	-8.095383000	-1.860848000	-0.418560000
6	-7.649334000	1.758528000	0.480962000
6	-8.367124000	-0.590427000	0.354545000
6	-3.673384000	0.566501000	-0.047594000
6	-4.906717000	3.202946000	1.223844000
6	-4.596506000	3.226719000	-1.273536000
6	-6.806387000	-3.107148000	1.354414000
6	-6.431012000	-3.578498000	-1.071951000
6	-4.345438000	-1.733450000	-0.099264000
6	-3.310502000	-0.784986000	-0.072440000
6	-1.935949000	-1.125974000	-0.076060000

6	-0.943874000	-0.184543000	-0.048502000
6	-1.339448000	1.211627000	-0.001196000
6	0.475890000	-0.533696000	-0.062862000
6	1.013384000	-1.801190000	-0.132721000
6	2.980664000	-0.564626000	-0.057401000
6	2.424043000	-1.819892000	-0.129465000
6	4.381751000	-0.205731000	-0.039769000
6	4.935535000	1.064074000	-0.084479000
6	6.886293000	-0.207447000	0.013873000
6	6.337131000	1.064114000	-0.054234000
6	8.238578000	-0.650261000	0.055671000
6	9.398367000	0.068330000	0.045924000
6	10.676810000	-0.669977000	0.096444000
6	9.444904000	1.490863000	-0.011449000
1	-7.046340000	2.558035000	-1.392533000
1	-7.082548000	3.750572000	-0.093786000
1	-8.112491000	-1.630781000	-1.490170000
1	-8.892643000	-2.585926000	-0.227973000
1	-7.496289000	1.909389000	1.560313000
1	-8.711567000	1.914757000	0.281049000
1	-9.314839000	-0.150933000	0.033436000
1	-8.457279000	-0.793537000	1.431970000
1	-3.842790000	3.075183000	1.429552000
1	-5.466540000	2.794891000	2.069931000
1	-5.115648000	4.276541000	1.165309000
1	-4.788986000	2.701552000	-2.214277000
1	-3.519728000	3.297041000	-1.130269000
1	-4.992924000	4.242841000	-1.370499000
1	-7.574057000	-3.887957000	1.381039000
1	-5.847579000	-3.563563000	1.616410000
1	-7.041677000	-2.370454000	2.126903000
1	-6.297810000	-3.161809000	-2.074617000
1	-5.534032000	-4.147227000	-0.815411000
1	-7.264898000	-4.286786000	-1.105268000
1	-4.067751000	-2.781399000	-0.131961000
1	-1.684227000	-2.181343000	-0.102505000
1	0.417530000	-2.702761000	-0.190501000
1	3.005014000	-2.733161000	-0.186920000
1	4.340938000	1.967124000	-0.144741000
1	6.928873000	1.968534000	-0.086039000
1	8.394528000	-1.725544000	0.102599000
1	12.546289000	-0.401526000	0.113578000

$Eelec$ = -2404.2290648

$H_{(298.15\ K)}$ = -2403.660646
$G_{(298.15\ K)}$ = -2403.767049
ZPE (Zero-point energy correction) = 0.532505

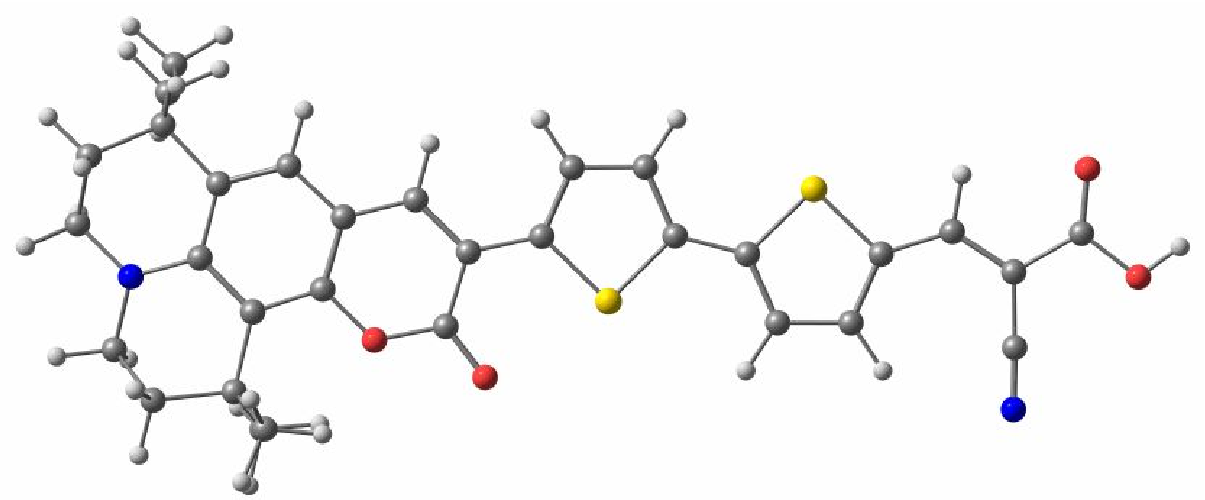

(*E*)-NKX 2677*, reference coumarin dye sensitizer at the first singlet excited state

6	5.600730000	1.367835000	0.105789000
6	4.770059000	0.298663000	0.061409000
6	3.324684000	0.515997000	0.096047000
8	2.876822000	1.811506000	0.167706000
6	3.711080000	2.887257000	0.223115000
6	3.123541000	4.137978000	0.309004000
6	3.952477000	5.252471000	0.363253000
6	5.347224000	5.114156000	0.332381000
6	5.905623000	3.852767000	0.247392000
6	5.098822000	2.708564000	0.191012000
7	2.497514000	-0.446991000	0.081253000
6	1.105140000	-0.307693000	0.061452000
6	0.356370000	-1.113581000	0.925122000
6	-1.027879000	-1.066987000	0.909177000
6	-1.715274000	-0.232236000	0.017548000
6	-0.958731000	0.564653000	-0.852067000
6	0.426423000	0.526366000	-0.835001000
6	-3.188962000	-0.238290000	-0.013857000
6	5.336472000	-1.057723000	-0.023940000
7	6.621535000	-1.237482000	-0.046838000
16	4.366834000	-2.527054000	-0.109614000
6	5.844510000	-3.445600000	-0.183875000
6	6.946009000	-2.574058000	-0.136035000
6	8.248252000	-3.088094000	-0.179642000
6	6.020312000	-4.827898000	-0.274800000
6	8.420622000	-4.456961000	-0.270159000
6	7.313871000	-5.321830000	-0.317608000
6	-3.915169000	-1.385390000	0.212124000
6	-5.331932000	-1.442053000	0.208388000

6	-6.122139000	-0.335954000	-0.033783000
6	-5.408866000	0.895442000	-0.278352000
6	-3.967194000	0.944786000	-0.271177000
7	-3.489730000	2.167333000	-0.498140000
7	-5.968869000	2.076944000	-0.515925000
16	-4.762157000	3.151947000	-0.708495000
6	-7.565948000	-0.247240000	-0.078227000
6	-8.613042000	-1.103821000	0.093037000
6	-9.923903000	-0.534933000	-0.053804000
7	-11.013919000	-0.153419000	-0.155137000
6	-8.598081000	-2.562524000	0.418316000
8	-7.595387000	-3.217833000	0.571723000
8	-9.797366000	-3.156291000	0.542648000
1	6.671187000	1.193334000	0.077098000
1	2.046282000	4.245870000	0.336491000
1	5.981944000	5.994248000	0.376149000
1	6.984675000	3.737629000	0.224534000
1	0.880217000	-1.767323000	1.613816000
1	-1.583574000	-1.676589000	1.614108000
1	-1.460322000	1.218641000	-1.553873000
1	0.989425000	1.141309000	-1.527341000
1	9.092128000	-2.407949000	-0.141934000
1	5.168574000	-5.498977000	-0.311791000
1	9.422546000	-4.872004000	-0.305140000
1	7.474375000	-6.392620000	-0.388771000
1	-3.383439000	-2.315105000	0.380191000
1	-5.812429000	-2.392262000	0.392610000
1	-7.883369000	0.767818000	-0.305541000
1	-10.539186000	-2.547749000	0.401022000
8	3.347954000	6.464283000	0.447168000
1	4.003626000	7.171004000	0.484238000

Eelec = -2404.1971395

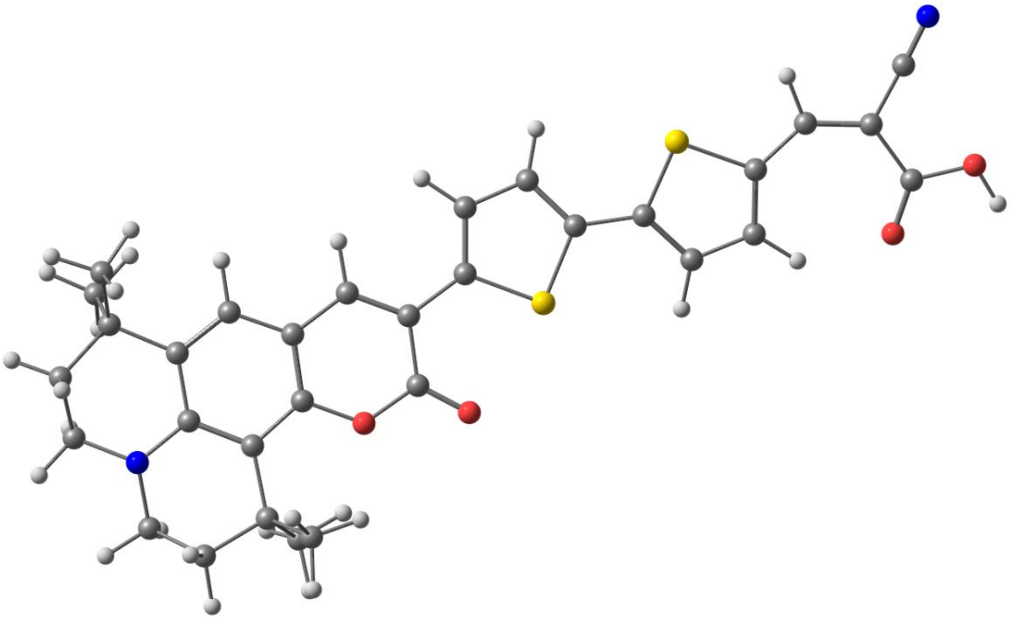

(*Z*)-NKX-2677, ground-state optimized geometry of the *Z*-stereoisomer of the reference dye

16	1.763089000	0.590388000	0.009217000
16	5.610536000	-1.475121000	-0.200841000
8	-2.638308000	1.483794000	-0.068123000
8	-0.525812000	2.110816000	-0.039943000
8	11.149305000	1.356940000	0.183032000
8	9.033187000	2.045242000	0.404119000
7	-7.312503000	0.454465000	0.026789000
7	11.435353000	-1.922836000	-0.469201000
6	-5.248616000	2.537150000	-0.239762000
6	-6.758970000	-2.429244000	0.025071000
6	-5.991572000	0.054675000	-0.032432000
6	-4.959571000	1.031069000	-0.116329000
6	-5.676959000	-1.343723000	-0.021572000
6	-6.749601000	2.747609000	-0.492035000
6	-8.114485000	-1.813455000	-0.341392000
6	-7.608066000	1.836413000	0.353897000
6	-8.346167000	-0.502176000	0.376080000
6	-3.647029000	0.562005000	-0.073717000
6	-4.840134000	3.272358000	1.051540000
6	-4.515367000	3.168696000	-1.440742000
6	-6.820462000	-3.040262000	1.438124000
6	-6.468673000	-3.553085000	-0.983233000
6	-4.354368000	-1.721812000	-0.019349000
6	-3.307508000	-0.792591000	-0.026667000
6	-1.930606000	-1.153950000	0.006567000
6	-0.929618000	-0.231153000	0.004073000

6	-1.302804000	1.181844000	-0.035419000
6	0.487565000	-0.590743000	0.040214000
6	1.011794000	-1.864209000	0.095229000
6	2.984370000	-0.641842000	0.076234000
6	2.421371000	-1.895585000	0.115776000
6	4.389701000	-0.292752000	0.089994000
6	4.957820000	0.947479000	0.316476000
6	6.900668000	-0.301971000	0.000393000
6	6.361353000	0.946206000	0.267018000
6	8.230014000	-0.808892000	-0.141456000
6	9.482804000	-0.262515000	-0.075406000
6	9.820958000	1.145838000	0.192866000
6	10.580196000	-1.162975000	-0.290506000
1	-6.978848000	2.547993000	-1.544789000
1	-7.000709000	3.795074000	-0.297004000
1	-8.154143000	-1.629428000	-1.421066000
1	-8.916854000	-2.518505000	-0.100285000
1	-7.452674000	2.026427000	1.428419000
1	-8.667441000	2.009385000	0.146197000
1	-9.306776000	-0.074060000	0.075957000
1	-8.390335000	-0.652432000	1.467028000
1	-5.033249000	4.345062000	0.942638000
1	-3.778041000	3.142487000	1.262612000
1	-5.405133000	2.912065000	1.916132000
1	-4.895025000	4.184270000	-1.595279000
1	-4.705874000	2.598431000	-2.355326000
1	-3.439632000	3.231836000	-1.288760000
1	-7.582825000	-3.825878000	1.480995000
1	-5.859767000	-3.486206000	1.710171000
1	-7.057597000	-2.291769000	2.198587000
1	-5.575670000	-4.126869000	-0.723417000
1	-7.308321000	-4.255451000	-1.003556000
1	-6.334088000	-3.152978000	-1.992144000
1	-4.096282000	-2.775581000	0.003195000
1	-1.692980000	-2.213052000	0.034033000
1	0.405129000	-2.760303000	0.126902000
1	2.998951000	-2.810826000	0.174167000
1	4.369695000	1.832210000	0.528000000
1	6.981689000	1.814725000	0.425758000
1	8.253833000	-1.876250000	-0.352644000
1	11.289800000	2.299184000	0.366440000

Eelec = -2404.198032

$H_{(298.15\ K)}$ = -2403.628682

$G_{(298.15\ K)}$ = -2403.735449
ZPE (Zero-point energy correction) = 0.533531

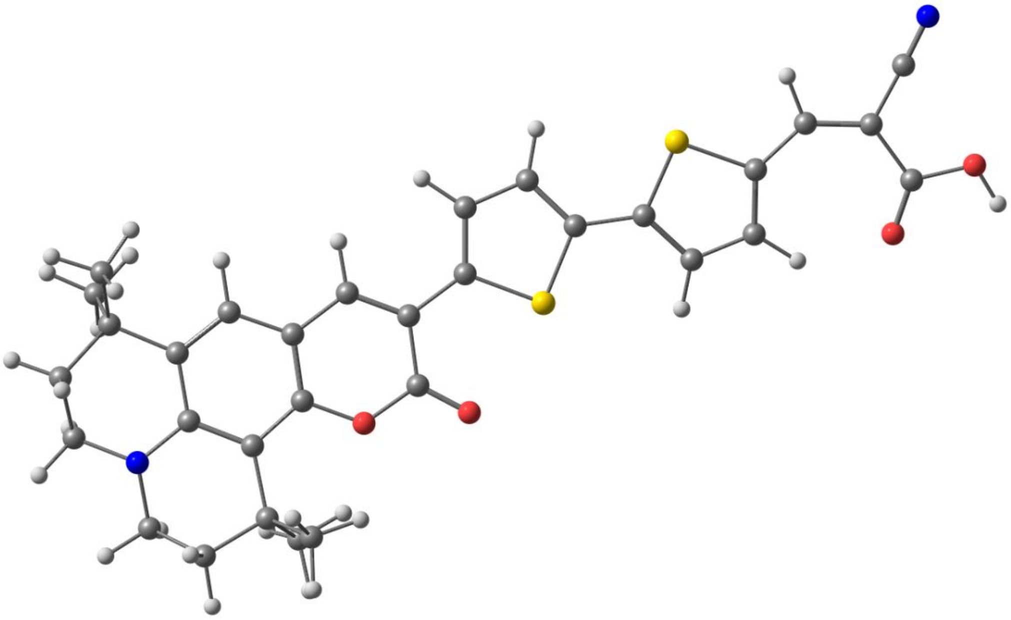

(*Z*)-NKX-2677, optimized geometry of the stereoisomer in the presence of an acetonitrile solvent (simulated through IEFPCM).

16	1.765812000	0.599503000	0.013635000
16	5.615657000	-1.498779000	-0.134075000
8	-2.638762000	1.490497000	-0.059656000
8	-0.534213000	2.130605000	-0.026125000
8	11.157949000	1.354489000	0.159802000
8	9.040536000	2.072497000	0.281063000
7	-7.303587000	0.440640000	0.079653000
7	11.459395000	-1.926821000	-0.254917000
6	-5.262960000	2.539390000	-0.201028000
6	-6.752322000	-2.436853000	0.019636000
6	-5.992885000	0.049972000	-0.010573000
6	-4.962128000	1.034338000	-0.095912000
6	-5.670141000	-1.352300000	-0.027115000
6	-6.766184000	2.741546000	-0.445494000
6	-8.101213000	-1.820421000	-0.367486000
6	-7.614290000	1.822702000	0.401900000
6	-8.356079000	-0.522319000	0.364251000
6	-3.651195000	0.567767000	-0.070132000
6	-4.856995000	3.263577000	1.097184000
6	-4.541408000	3.189411000	-1.398996000
6	-6.825868000	-3.030602000	1.439653000
6	-6.454059000	-3.572287000	-0.972687000
6	-4.349405000	-1.724945000	-0.042029000
6	-3.303791000	-0.787860000	-0.043917000
6	-1.933440000	-1.144390000	-0.026473000

6	-0.930679000	-0.213802000	-0.026747000
6	-1.310184000	1.187524000	-0.037642000
6	0.484906000	-0.579306000	-0.013937000
6	1.007703000	-1.855131000	-0.022583000
6	2.988662000	-0.639098000	0.017344000
6	2.417586000	-1.890028000	-0.004486000
6	4.393093000	-0.296195000	0.039335000
6	4.959744000	0.960475000	0.189208000
6	6.905019000	-0.313534000	0.003799000
6	6.360311000	0.953292000	0.169657000
6	8.232344000	-0.822707000	-0.075195000
6	9.485318000	-0.263743000	-0.020882000
6	9.829064000	1.155317000	0.153465000
6	10.585744000	-1.172407000	-0.149040000
1	-6.998749000	2.545898000	-1.498302000
1	-7.024140000	3.785780000	-0.244319000
1	-8.118292000	-1.624815000	-1.445909000
1	-8.906315000	-2.529642000	-0.151941000
1	-7.461292000	2.011382000	1.475294000
1	-8.674348000	1.983467000	0.194103000
1	-9.300601000	-0.083497000	0.033233000
1	-8.442464000	-0.688545000	1.448296000
1	-5.053255000	4.336582000	0.998016000
1	-3.795121000	3.131188000	1.310409000
1	-5.423481000	2.893806000	1.956377000
1	-4.924938000	4.205962000	-1.535207000
1	-4.738914000	2.631307000	-2.319514000
1	-3.464015000	3.251575000	-1.256350000
1	-7.599626000	-3.804425000	1.486208000
1	-5.871261000	-3.487077000	1.716373000
1	-7.057050000	-2.270929000	2.190859000
1	-5.563071000	-4.142950000	-0.699981000
1	-7.295177000	-4.272648000	-0.987628000
1	-6.315827000	-3.184364000	-1.986131000
1	-4.083607000	-2.776486000	-0.035965000
1	-1.694025000	-2.202866000	-0.012242000
1	0.401392000	-2.751341000	-0.040542000
1	2.988145000	-2.811596000	-0.001909000
1	4.371938000	1.861027000	0.317661000
1	6.976179000	1.832480000	0.276011000
1	8.262836000	-1.901683000	-0.210337000
1	11.321238000	2.305068000	0.278167000

Eelec = -2404.2228692

$H_{(298.15\ K)}$ = -2403.654441
$G_{(298.15\ K)}$ = -2403.761403
ZPE (Zero-point energy correction) = 0.532533

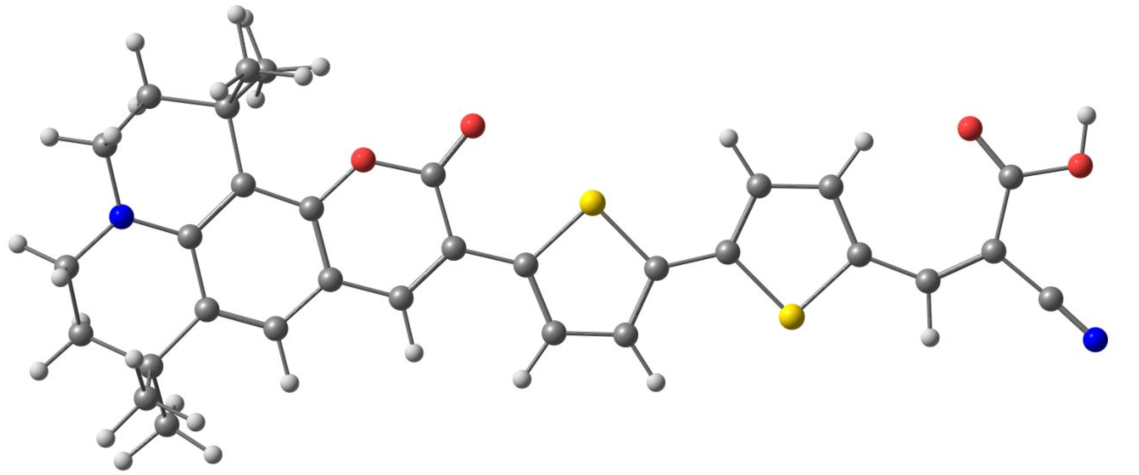

(*Z*)-NKX-2677*, stereoisomer of the reference dye's first singlet excited state optimized geometry.

16	1.763710000	0.665670000	-0.006243000
16	5.586023000	-1.530809000	-0.023914000
8	-2.638623000	1.503539000	-0.024180000
8	-0.534708000	2.151218000	0.008678000
8	11.137055000	1.358080000	0.076997000
8	9.019026000	2.076684000	0.075431000
7	-7.286013000	0.423169000	0.114513000
7	11.417811000	-1.985494000	0.011000000
6	-5.252448000	2.534400000	-0.134345000
6	-6.731921000	-2.448794000	-0.027344000
6	-5.976427000	0.037610000	-0.001095000
6	-4.946167000	1.028143000	-0.067568000
6	-5.650488000	-1.364281000	-0.054958000
6	-6.755972000	2.734400000	-0.377056000
6	-8.076423000	-1.824231000	-0.416458000
6	-7.602600000	1.799063000	0.454280000
6	-8.343754000	-0.545478000	0.345360000
6	-3.636332000	0.571440000	-0.055122000
6	-4.848916000	3.224950000	1.182937000
6	-4.531495000	3.219771000	-1.313088000
6	-6.814872000	-3.059073000	1.385240000
6	-6.431098000	-3.572703000	-1.031586000
6	-4.331202000	-1.730920000	-0.085376000
6	-3.277807000	-0.792745000	-0.064277000
6	-1.920440000	-1.150539000	-0.060782000
6	-0.904077000	-0.196462000	-0.040432000
6	-1.297460000	1.212952000	-0.017797000
6	0.477222000	-0.522546000	-0.038205000

6	1.017094000	-1.828046000	-0.061024000
6	2.989360000	-0.593074000	-0.023680000
6	2.395023000	-1.870694000	-0.053123000
6	4.358959000	-0.286170000	-0.008402000
6	4.963156000	0.986775000	0.020957000
6	6.887169000	-0.340675000	0.009099000
6	6.345005000	0.960047000	0.030625000
6	8.204118000	-0.850226000	0.008893000
6	9.469802000	-0.279538000	0.030238000
6	9.803477000	1.143041000	0.061968000
6	10.564845000	-1.199365000	0.020022000
1	-6.987102000	2.556215000	-1.433404000
1	-7.018435000	3.774116000	-0.158206000
1	-8.078925000	-1.600841000	-1.489566000
1	-8.884608000	-2.538943000	-0.230571000
1	-7.450627000	1.972435000	1.531006000
1	-8.664160000	1.957230000	0.248720000
1	-9.281706000	-0.095449000	0.008214000
1	-8.454650000	-0.743407000	1.422785000
1	-5.060633000	4.297518000	1.117425000
1	-3.783247000	3.104839000	1.381112000
1	-5.400388000	2.822044000	2.037446000
1	-4.927618000	4.233942000	-1.429953000
1	-4.714260000	2.680381000	-2.247720000
1	-3.456922000	3.294826000	-1.160205000
1	-7.592614000	-3.829588000	1.422642000
1	-5.864087000	-3.523785000	1.660007000
1	-7.040260000	-2.308450000	2.147155000
1	-5.545863000	-4.151592000	-0.757901000
1	-7.273707000	-4.270743000	-1.063838000
1	-6.279519000	-3.174046000	-2.038628000
1	-4.064534000	-2.781995000	-0.110834000
1	-1.672893000	-2.206116000	-0.075340000
1	0.399561000	-2.716432000	-0.083528000
1	2.973489000	-2.786546000	-0.068117000
1	4.382741000	1.901934000	0.034598000
1	6.983303000	1.830305000	0.052535000
1	8.243810000	-1.937182000	-0.012723000
1	11.261549000	2.319481000	0.098003000

$Eelec$ = -2404.1906665

Gas-phase global minima geometries for dyes of the proposed series A-D

A1, benzothiadiazole-containing dye with a H atom at the 7-coumarin position at the ground state

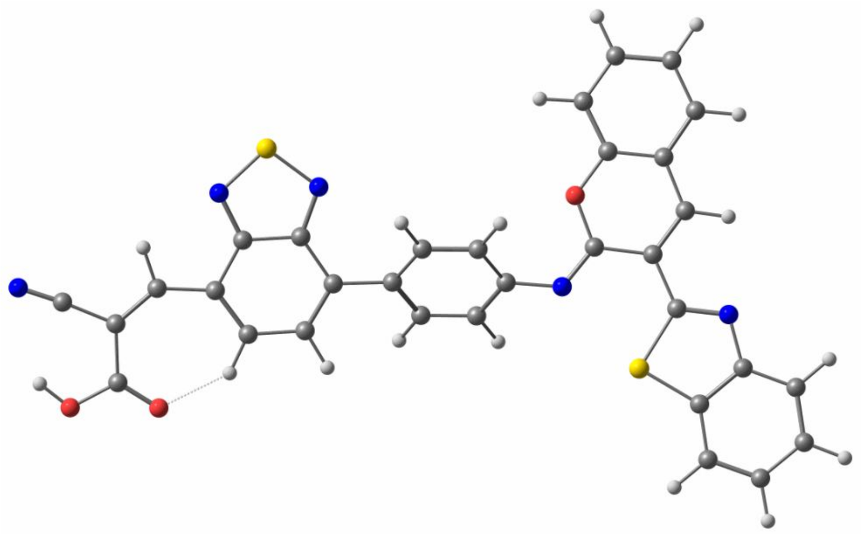

6	-5.654821000	1.670753000	0.150562000
6	-4.847732000	0.586501000	0.089177000
6	-3.395661000	0.771020000	0.124058000
8	-2.920136000	2.051828000	0.212261000
6	-3.733311000	3.146422000	0.285687000
6	-3.124821000	4.390306000	0.389588000
6	-3.928796000	5.518973000	0.462403000
6	-5.322801000	5.406918000	0.432036000
6	-5.912359000	4.158692000	0.329557000
6	-5.121824000	3.002713000	0.254935000
7	-2.594215000	-0.213339000	0.093917000
6	-1.198647000	-0.106469000	0.073526000
6	-0.467151000	-0.933547000	0.931682000
6	0.917862000	-0.918216000	0.913341000
6	1.622463000	-0.094864000	0.024638000
6	0.882758000	0.723410000	-0.839544000
6	-0.502902000	0.716621000	-0.819863000
6	3.095681000	-0.134391000	-0.009465000
6	-5.442919000	-0.757462000	-0.014856000
7	-6.731544000	-0.907495000	-0.039116000
16	-4.506774000	-2.246340000	-0.121763000
6	-6.004575000	-3.129976000	-0.207245000
6	-7.086293000	-2.234747000	-0.146436000
6	-8.400138000	-2.718202000	-0.195838000
6	-6.211739000	-4.506772000	-0.316947000
6	-8.603304000	-4.081236000	-0.304846000

6	-7.516126000	-4.970184000	-0.365172000
6	3.795664000	-1.299121000	0.208254000
6	5.210884000	-1.388236000	0.202198000
6	6.025805000	-0.299123000	-0.034287000
6	5.340587000	0.949680000	-0.270619000
6	3.900400000	1.032115000	-0.260917000
7	3.450871000	2.266652000	-0.480017000
7	5.927309000	2.119337000	-0.502210000
16	4.745527000	3.222884000	-0.686798000
6	7.471391000	-0.243119000	-0.080234000
6	8.498660000	-1.124202000	0.085819000
6	9.822073000	-0.584858000	-0.060170000
7	10.920358000	-0.227841000	-0.161248000
6	8.450564000	-2.583709000	0.404878000
8	7.433135000	-3.216233000	0.556740000
8	9.635891000	-3.205396000	0.525173000
1	-6.728917000	1.521379000	0.121566000
1	-2.042918000	4.453113000	0.414441000
1	-3.467010000	6.497192000	0.544995000
1	-5.939916000	6.296622000	0.490112000
1	-6.993017000	4.057508000	0.306817000
1	-1.004275000	-1.578853000	1.618105000
1	1.460794000	-1.543931000	1.614086000
1	1.397910000	1.369040000	-1.539286000
1	-1.053313000	1.347564000	-1.508009000
1	-9.228232000	-2.019589000	-0.148034000
1	-5.375573000	-5.196505000	-0.363949000
1	-9.614265000	-4.473218000	-0.344516000
1	-7.701047000	-6.035954000	-0.450822000
1	3.243062000	-2.217471000	0.371437000
1	5.669710000	-2.350291000	0.380095000
1	7.811683000	0.765462000	-0.303180000
1	10.391483000	-2.613678000	0.385046000

$Eelec$ = -2524.620617
$H_{(298.15\ K)}$ = -2524.160494
$G_{(298.15\ K)}$ = -2524.264445
ZPE (Zero-point energy correction) = 0.426521

A2, benzothiadiazole-containing dye with an OH substituent at the 7-coumarin position at the ground state

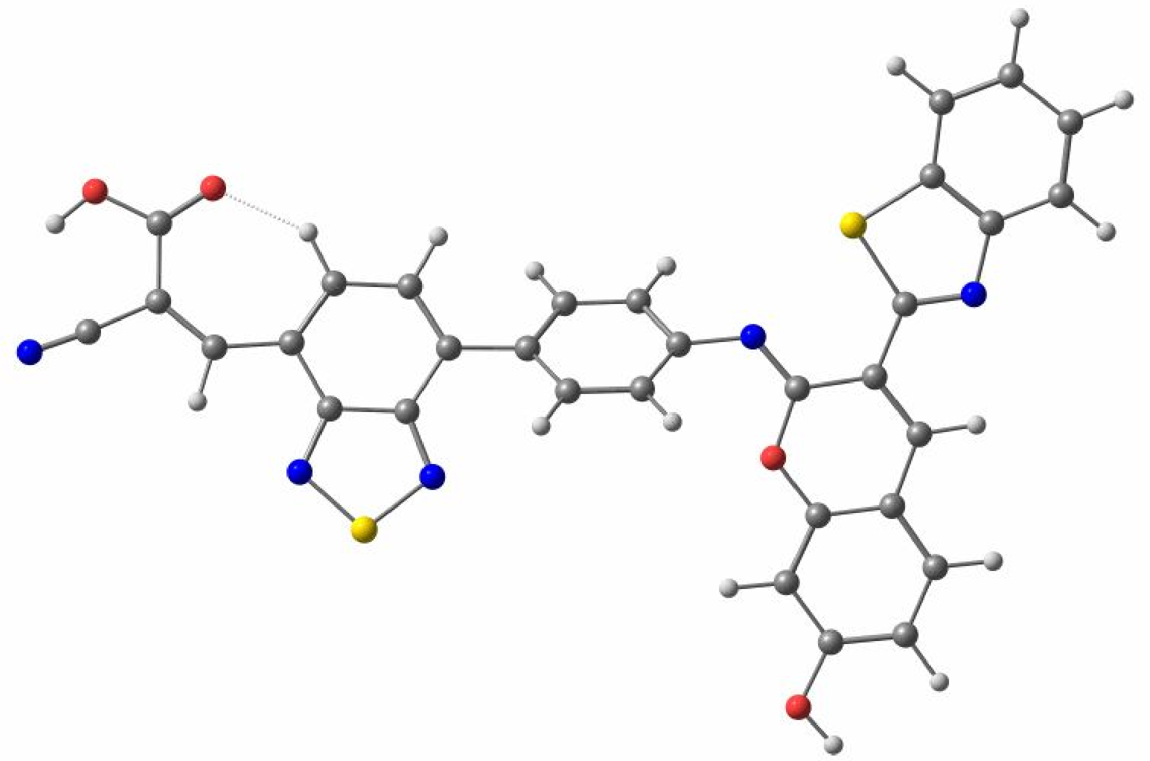

6	5.600730000	1.367835000	0.105789000
6	4.770059000	0.298663000	0.061409000
6	3.324684000	0.515997000	0.096047000
8	2.876822000	1.811506000	0.167706000
6	3.711080000	2.887257000	0.223115000
6	3.123541000	4.137978000	0.309004000
6	3.952477000	5.252471000	0.363253000
6	5.347224000	5.114156000	0.332381000
6	5.905623000	3.852767000	0.247392000
6	5.098822000	2.708564000	0.191012000
7	2.497514000	-0.446991000	0.081253000
6	1.105140000	-0.307693000	0.061452000
6	0.356370000	-1.113581000	0.925122000
6	-1.027879000	-1.066987000	0.909177000
6	-1.715274000	-0.232236000	0.017548000
6	-0.958731000	0.564653000	-0.852067000
6	0.426423000	0.526366000	-0.835001000
6	-3.188962000	-0.238290000	-0.013857000
6	5.336472000	-1.057723000	-0.023940000
7	6.621535000	-1.237482000	-0.046838000
16	4.366834000	-2.527054000	-0.109614000
6	5.844510000	-3.445600000	-0.183875000

6	6.946009000	-2.574058000	-0.136035000
6	8.248252000	-3.088094000	-0.179642000
6	6.020312000	-4.827898000	-0.274800000
6	8.420622000	-4.456961000	-0.270159000
6	7.313871000	-5.321830000	-0.317608000
6	-3.915169000	-1.385390000	0.212124000
6	-5.331932000	-1.442053000	0.208388000
6	-6.122139000	-0.335954000	-0.033783000
6	-5.408866000	0.895442000	-0.278352000
6	-3.967194000	0.944786000	-0.271177000
7	-3.489730000	2.167333000	-0.498140000
7	-5.968869000	2.076944000	-0.515925000
16	-4.762157000	3.151947000	-0.708495000
6	-7.565948000	-0.247240000	-0.078227000
6	-8.613042000	-1.103821000	0.093037000
6	-9.923903000	-0.534933000	-0.053804000
7	-11.013919000	-0.153419000	-0.155137000
6	-8.598081000	-2.562524000	0.418316000
8	-7.595387000	-3.217833000	0.571723000
8	-9.797366000	-3.156291000	0.542648000
1	6.671187000	1.193334000	0.077098000
1	2.046282000	4.245870000	0.336491000
1	5.981944000	5.994248000	0.376149000
1	6.984675000	3.737629000	0.224534000
1	0.880217000	-1.767323000	1.613816000
1	-1.583574000	-1.676589000	1.614108000
1	-1.460322000	1.218641000	-1.553873000
1	0.989425000	1.141309000	-1.527341000
1	9.092128000	-2.407949000	-0.141934000
1	5.168574000	-5.498977000	-0.311791000
1	9.422546000	-4.872004000	-0.305140000
1	7.474375000	-6.392620000	-0.388771000
1	-3.383439000	-2.315105000	0.380191000
1	-5.812429000	-2.392262000	0.392610000
1	-7.883369000	0.767818000	-0.305541000
1	-10.539186000	-2.547749000	0.401022000
8	3.347954000	6.464283000	0.447168000
1	4.003626000	7.171004000	0.484238000

Eelec = -2599.827549

$H_{(298.15\ K)}$ = -2599.362164

$G_{(298.15\ K)}$ = -2599.468836

ZPE (Zero-point energy correction) = 0.430518

A3, benzothiadiazole-containing dye with an NH2 substituent at the 7-coumarin position at the ground state

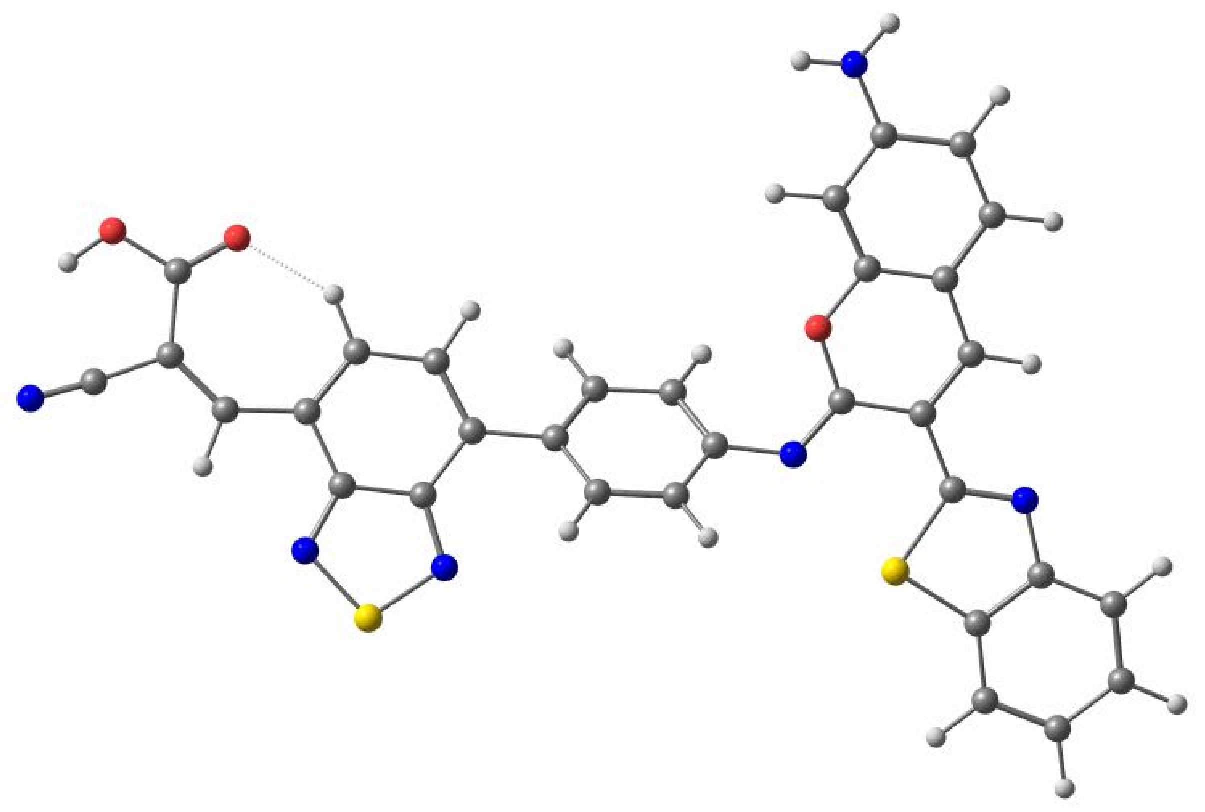

6	5.450370000	1.834908000	0.049721000
6	4.726795000	0.687592000	0.004971000
6	3.270050000	0.764321000	0.051270000
8	2.699778000	2.010407000	0.136378000
6	3.427136000	3.162176000	0.191906000
6	2.721428000	4.347938000	0.293054000
6	3.424522000	5.555128000	0.350970000
6	4.835058000	5.535604000	0.303777000
6	5.511877000	4.340009000	0.204957000
6	4.824453000	3.116682000	0.146777000
7	2.535059000	-0.272415000	0.035606000
6	1.137064000	-0.258701000	0.023159000
6	0.384177000	0.498498000	-0.883837000
6	-0.997805000	0.408699000	-0.891991000
6	-1.681029000	-0.432392000	-0.001838000
6	-0.919684000	-1.205644000	0.884612000
6	0.463588000	-1.122676000	0.892650000
6	-3.154179000	-0.463774000	-0.000949000
6	5.421801000	-0.605244000	-0.091864000
7	6.718179000	-0.661172000	-0.127289000

16	4.597060000	-2.161979000	-0.173357000
6	6.155867000	-2.934030000	-0.263483000
6	7.168547000	-1.960270000	-0.223342000
6	8.513554000	-2.346768000	-0.279267000
6	6.463226000	-4.292692000	-0.358715000
6	8.816414000	-3.692810000	-0.373528000
6	7.798033000	-4.660229000	-0.413158000
6	-3.903015000	0.661857000	-0.261842000
6	-5.319812000	0.686217000	-0.284899000
6	-6.089498000	-0.433324000	-0.036959000
6	-5.353003000	-1.643616000	0.241628000
6	-3.910595000	-1.659711000	0.262909000
7	-3.410274000	-2.866951000	0.520160000
7	-5.890445000	-2.833551000	0.488929000
16	-4.663329000	-3.877018000	0.722702000
6	-7.531015000	-0.553827000	-0.020300000
6	-8.593543000	0.280191000	-0.209892000
6	-9.894157000	-0.317533000	-0.089395000
7	-10.977619000	-0.722226000	-0.009520000
6	-8.605102000	1.740352000	-0.526963000
8	-7.614949000	2.418915000	-0.661510000
8	-9.815074000	2.309221000	-0.665005000
1	6.532499000	1.764057000	0.011903000
1	1.637694000	4.321337000	0.324527000
1	5.382765000	6.471758000	0.342963000
1	6.597061000	4.333370000	0.172214000
1	0.888284000	1.143616000	-1.594070000
1	-1.556277000	0.983312000	-1.623896000
1	-1.414539000	-1.879215000	1.572294000
1	1.047039000	-1.728561000	1.577190000
1	9.288349000	-1.588559000	-0.247618000
1	5.679582000	-5.042497000	-0.389744000
1	9.853442000	-4.009153000	-0.417740000
1	8.060184000	-5.710566000	-0.487688000
1	-3.389353000	1.600222000	-0.437548000
1	-5.818393000	1.621880000	-0.493729000
1	-7.830402000	-1.575623000	0.201365000
1	-10.545366000	1.683554000	-0.538609000
7	2.750637000	6.754152000	0.412700000
1	3.262630000	7.574854000	0.694572000
1	1.779190000	6.745400000	0.680255000

$Eelec$ = -2579.964424

$H_{(298.15\ K)}$ = -2579.486282

$G_{(298.15\ K)}$ = -2579.593345
ZPE (Zero-point energy correction) = 0.442929

A4, benzothiadiazole-containing dye with a CF3 substituent at the 7-coumarin position at the ground state

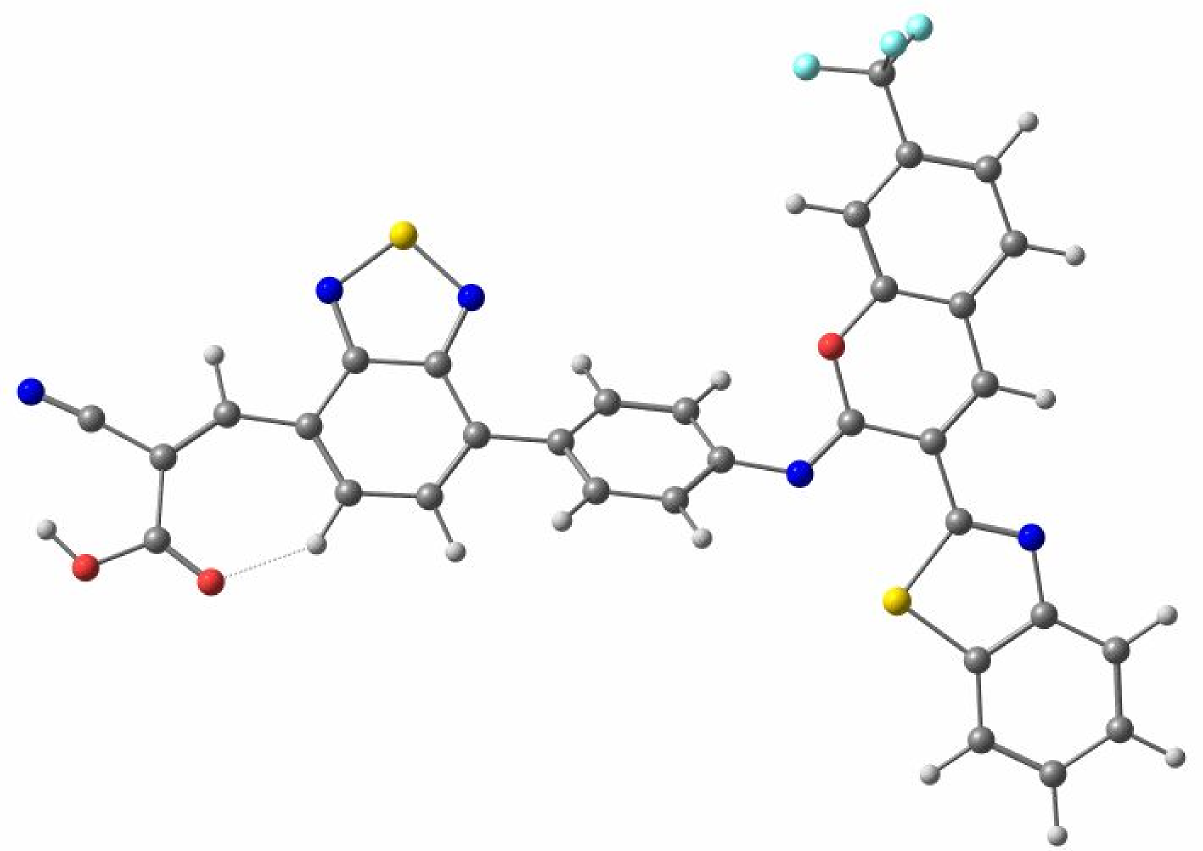

6	5.417730000	0.455228000	0.048185000
6	4.513809000	-0.549762000	0.020349000
6	3.082965000	-0.232334000	0.043012000
8	2.727372000	1.092421000	0.079277000
6	3.638482000	2.104843000	0.118554000
6	3.144442000	3.401591000	0.170582000
6	4.051512000	4.449281000	0.205833000
6	5.430740000	4.214011000	0.191098000
6	5.899722000	2.915042000	0.140264000
6	5.007070000	1.834296000	0.103242000
7	2.195743000	-1.137590000	0.048370000
6	0.814082000	-0.902593000	0.025876000
6	0.015623000	-1.617002000	0.923416000
6	-1.361851000	-1.467966000	0.910414000
6	-1.990475000	-0.621516000	-0.012333000
6	-1.183837000	0.080815000	-0.917292000
6	0.195017000	-0.058449000	-0.902659000
6	-3.461531000	-0.516664000	-0.036785000
6	4.982338000	-1.946226000	-0.035537000

7	6.252429000	-2.211237000	-0.049692000
16	3.916573000	-3.346443000	-0.096997000
6	5.328478000	-4.363010000	-0.148002000
6	6.486680000	-3.567425000	-0.112065000
6	7.752071000	-4.168212000	-0.140471000
6	5.410550000	-5.755873000	-0.212281000
6	7.831215000	-5.546493000	-0.204371000
6	6.667996000	-6.335382000	-0.240188000
6	-4.270757000	-1.596866000	0.229409000
6	-5.688333000	-1.544532000	0.234572000
6	-6.392218000	-0.388961000	-0.038280000
6	-5.588417000	0.775496000	-0.325970000
6	-4.147526000	0.713983000	-0.328034000
7	-3.578960000	1.888163000	-0.597194000
7	-6.057600000	1.988602000	-0.598639000
16	-4.773743000	2.960703000	-0.832272000
6	-7.825991000	-0.189801000	-0.077729000
6	-8.934124000	-0.956332000	0.128622000
6	-10.198683000	-0.293002000	-0.029555000
7	-11.256948000	0.167779000	-0.136979000
6	-9.029053000	-2.400531000	0.503325000
8	-8.078222000	-3.125716000	0.671546000
8	-10.269010000	-2.894902000	0.656307000
1	6.473531000	0.207518000	0.029501000
1	2.074867000	3.570943000	0.181596000
1	6.122610000	5.047954000	0.216061000
1	6.966667000	2.717650000	0.127123000
1	0.494049000	-2.279652000	1.636235000
1	-1.957252000	-2.005451000	1.641026000
1	-1.640979000	0.741060000	-1.643204000
1	0.797982000	0.484030000	-1.621591000
1	8.639657000	-3.545784000	-0.112109000
1	4.515901000	-6.368893000	-0.240301000
1	8.802516000	-6.029373000	-0.227404000
1	6.756499000	-7.415643000	-0.290497000
1	-3.810381000	-2.558728000	0.425427000
1	-6.238882000	-2.448704000	0.451439000
1	-8.065637000	0.838718000	-0.337400000
1	-10.963337000	-2.236004000	0.499706000
6	3.559147000	5.866609000	0.297897000
9	4.208017000	6.676537000	-0.563351000
9	2.244425000	5.971502000	0.033720000
9	3.756659000	6.380201000	1.531408000

E_{elec} = -2861.602753
$H_{(298.15\ K)}$ = -2861.134406
$G_{(298.15\ K)}$ = -2861.249035
ZPE (Zero-point energy correction) = 0.430963

A5, benzothiadiazole-containing dye with an NO2 substituent at the 7-coumarin position at the ground state

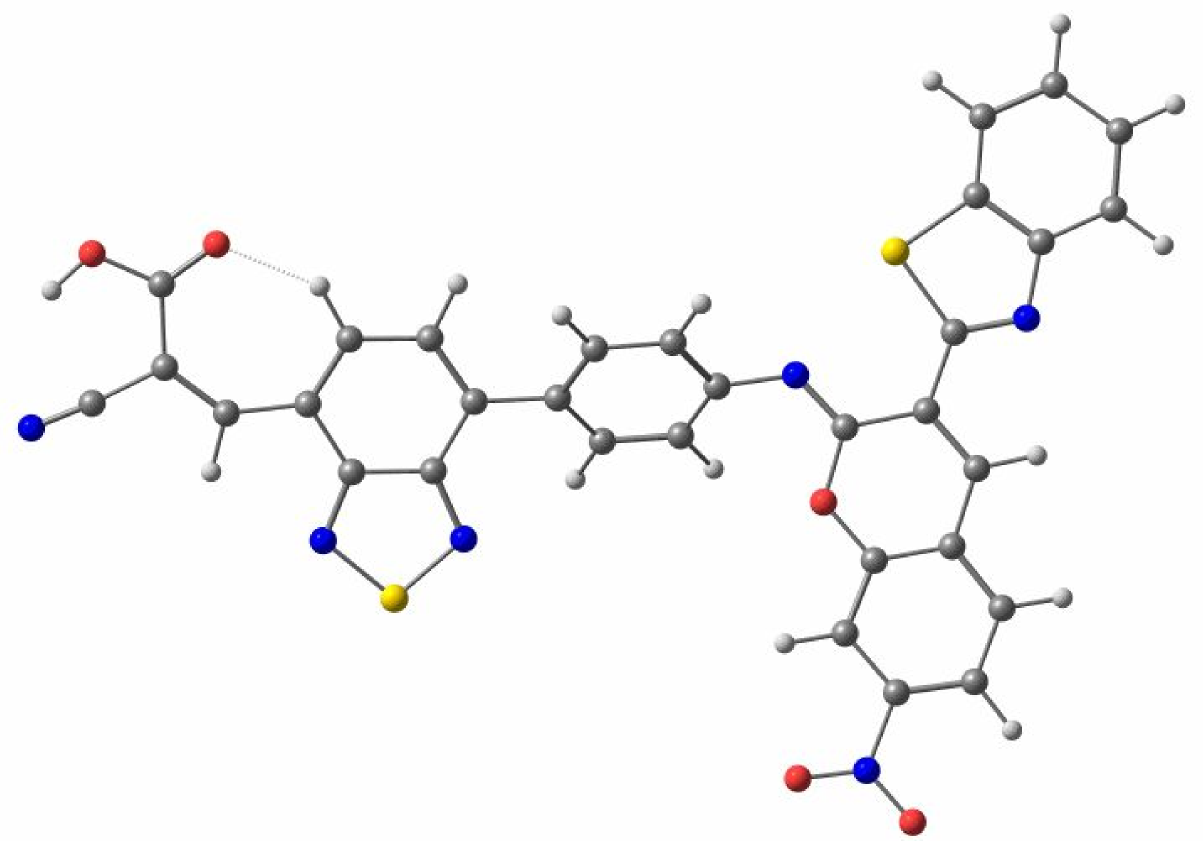

6	-5.461386000	4.563286000	0.217635000
6	-4.081596000	4.750370000	0.229324000
6	-3.177739000	3.702424000	0.189360000
6	-3.696373000	2.418225000	0.134619000
6	-5.073062000	2.174811000	0.120685000
6	-5.947488000	3.269557000	0.163166000
8	-2.806318000	1.388368000	0.091745000
6	-3.189116000	0.070295000	0.052484000
6	-4.627112000	-0.217712000	0.031390000
6	-5.511448000	0.804250000	0.063249000
7	-2.323329000	-0.854173000	0.053789000
6	-0.934960000	-0.658940000	0.033782000
6	-0.159192000	-1.437637000	0.897122000
6	1.222220000	-1.331592000	0.888699000
6	1.876509000	-0.465758000	0.002670000
6	1.092322000	0.300762000	-0.868965000
6	-0.290462000	0.205621000	-0.857694000
6	3.350453000	-0.406222000	-0.020768000
6	4.125219000	-1.522723000	0.191438000

6	5.543969000	-1.515194000	0.193545000
6	6.282926000	-0.370591000	-0.027046000
6	5.515652000	0.831012000	-0.255566000
6	4.073815000	0.814683000	-0.255661000
6	7.722475000	-0.214644000	-0.061975000
6	8.806054000	-1.026122000	0.096203000
6	-5.123564000	-1.604435000	-0.026900000
7	-6.399331000	-1.841591000	-0.037783000
6	-6.663078000	-3.191855000	-0.103577000
6	-5.522177000	-4.011975000	-0.145808000
16	-4.088756000	-3.026486000	-0.096340000
6	-7.941500000	-3.764981000	-0.129756000
6	-8.050283000	-5.140776000	-0.197826000
6	-6.904143000	-5.954361000	-0.239964000
6	-5.634410000	-5.402644000	-0.214268000
7	6.021911000	2.040721000	-0.471598000
16	4.768667000	3.062245000	-0.654224000
7	3.541565000	2.017431000	-0.466962000
7	-3.553039000	6.121916000	0.286721000
8	-2.338972000	6.262166000	0.289766000
8	-4.360979000	7.038056000	0.327110000
6	8.856188000	-2.491622000	0.388808000
8	7.883236000	-3.193755000	0.525264000
8	10.080187000	-3.033750000	0.501878000
6	10.090741000	-0.395766000	-0.031885000
7	11.162974000	0.035873000	-0.119686000
1	-6.119252000	5.421396000	0.251296000
1	-2.110045000	3.877799000	0.203091000
1	-7.017345000	3.089995000	0.153270000
1	-6.571918000	0.577918000	0.046013000
1	-0.658236000	-2.116044000	1.580337000
1	1.800760000	-1.919388000	1.593606000
1	1.569766000	0.977552000	-1.565948000
1	-0.874980000	0.798664000	-1.551149000
1	3.635153000	-2.477852000	0.343746000
1	6.066342000	-2.445384000	0.365277000
1	7.993815000	0.818437000	-0.266515000
1	-8.815329000	-3.123671000	-0.096502000
1	-9.031664000	-5.602801000	-0.219263000
1	-7.016215000	-7.032249000	-0.293506000
1	-4.753413000	-6.034822000	-0.247221000
1	10.794751000	-2.390071000	0.375925000

Eelec = -2729.062128

$H_{(298.15\ K)}$ = -2728.596903
$G_{(298.15\ K)}$ = -2728.708027
ZPE (Zero-point energy correction) = 0.428957

B1, *p*-phenylynyl-containing dye with a H atom at the 7-coumarin position at the ground state

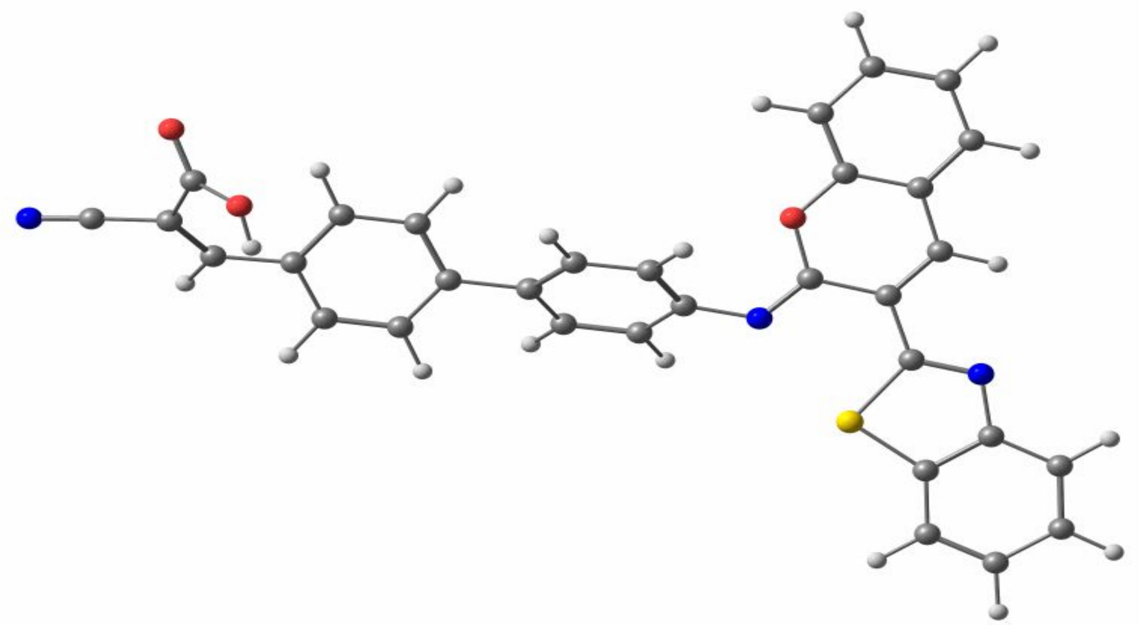

6	4.891507000	2.015828000	-0.002110000
6	4.211076000	0.846560000	-0.014581000
6	2.750724000	0.867580000	-0.120911000
8	2.136562000	2.089928000	-0.190287000
6	2.822881000	3.270767000	-0.188868000
6	2.082941000	4.441916000	-0.284093000
6	2.756003000	5.655480000	-0.282511000
6	4.150806000	5.698925000	-0.187076000
6	4.872746000	4.521489000	-0.093810000
6	4.215896000	3.282475000	-0.093642000
7	2.067227000	-0.201143000	-0.166643000
6	0.667902000	-0.250910000	-0.224543000
6	-0.163693000	0.448334000	0.657683000
6	-1.540529000	0.292435000	0.595772000
6	-2.135124000	-0.556285000	-0.344929000
6	-1.295625000	-1.257393000	-1.217279000
6	0.082654000	-1.117942000	-1.151762000
6	-3.606353000	-0.712212000	-0.406540000
6	4.949817000	-0.424767000	0.081357000
7	6.244356000	-0.429838000	0.168975000
16	4.185374000	-2.012402000	0.097170000
6	5.768167000	-2.724351000	0.236934000
6	6.742475000	-1.711629000	0.257574000
6	8.098956000	-2.045618000	0.361020000
6	6.125467000	-4.072007000	0.318165000

6	8.450769000	-3.380113000	0.441257000
6	7.470400000	-4.387201000	0.420170000
6	-4.456532000	0.383378000	-0.205979000
6	-5.834457000	0.245244000	-0.254418000
6	-6.417928000	-1.003204000	-0.510791000
6	-5.569375000	-2.093617000	-0.748775000
6	-4.192477000	-1.954365000	-0.681708000
6	-7.865778000	-1.211959000	-0.598510000
6	-8.858306000	-0.569576000	0.052579000
6	-10.218456000	-0.884387000	-0.278658000
7	-11.307526000	-1.169479000	-0.545651000
6	-8.704802000	0.499998000	1.105094000
8	-9.356843000	1.507870000	1.102403000
8	-7.815617000	0.254273000	2.085363000
1	5.972977000	1.987094000	0.077828000
1	1.003173000	4.384736000	-0.360540000
1	2.190435000	6.578216000	-0.357668000
1	4.665074000	6.653533000	-0.187423000
1	5.955734000	4.540958000	-0.020765000
1	0.273130000	1.101374000	1.404362000
1	-2.163049000	0.823373000	1.309200000
1	-1.725136000	-1.910032000	-1.970646000
1	0.727342000	-1.671357000	-1.825892000
1	8.843288000	-1.257061000	0.375979000
1	5.372145000	-4.852646000	0.302439000
1	9.496739000	-3.657074000	0.521857000
1	7.771363000	-5.427705000	0.484964000
1	-4.030337000	1.366871000	-0.040134000
1	-6.455913000	1.125264000	-0.130288000
1	-8.177733000	-1.987845000	-1.294041000
1	-7.376606000	-0.599418000	1.963112000
1	-5.998941000	-3.066538000	-0.969951000
1	-3.561391000	-2.823916000	-0.830392000

Eelec = -2018.190984
$H_{(298.15\ K)}$ = -2017.723294
$G_{(298.15\ K)}$ = -2017.823497
ZPE (Zero-point energy correction) = 0.435757

B2, *p*-phenylynyl-containing dye with an OH substituent at the 7-coumarin position at the ground state

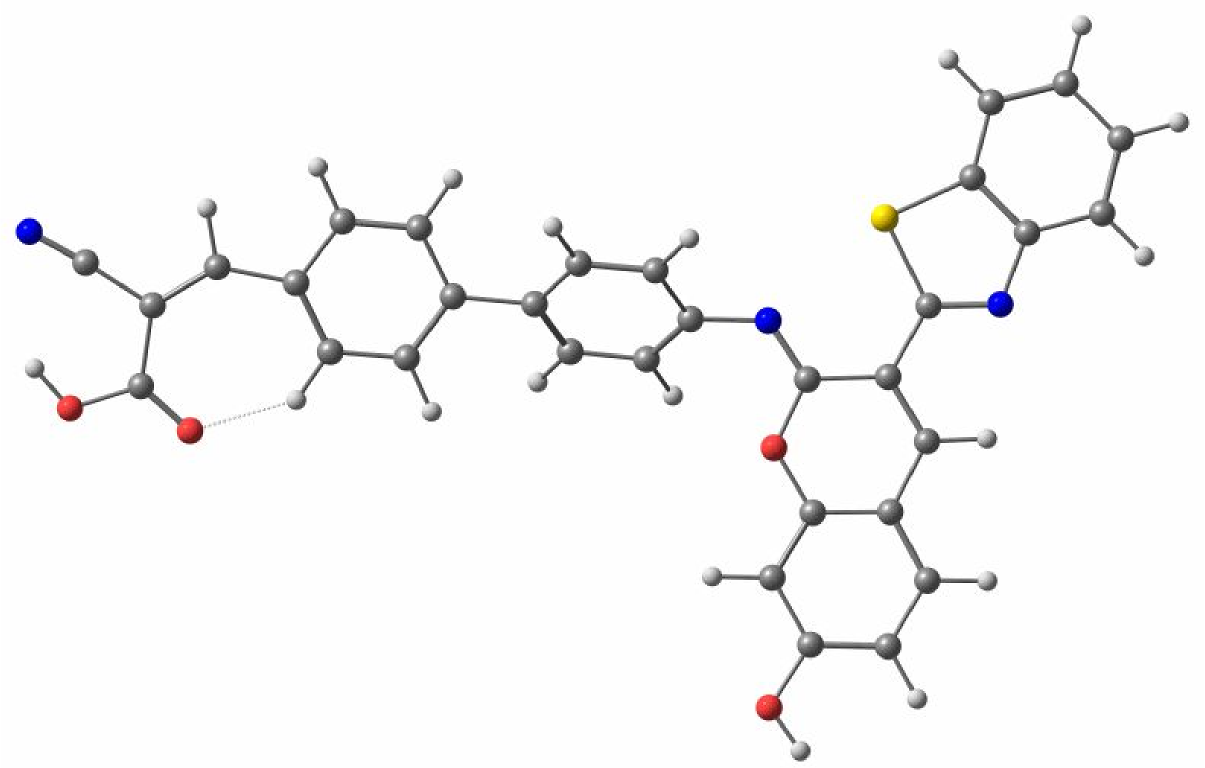

6	-4.939036000	1.745093000	0.007535000
6	-4.242343000	0.583491000	0.009087000
6	-2.781420000	0.626496000	0.054218000
8	-2.181721000	1.861724000	0.085371000
6	-2.881581000	3.030676000	0.094327000
6	-2.149384000	4.204684000	0.143248000
6	-2.838987000	5.411640000	0.149845000
6	-4.239938000	5.440040000	0.108471000
6	-4.944747000	4.252031000	0.061095000
6	-4.280544000	3.018388000	0.053011000
7	-2.076874000	-0.428784000	0.083244000
6	-0.676157000	-0.459247000	0.082185000
6	0.111721000	0.266383000	-0.819232000
6	1.491658000	0.129317000	-0.812679000
6	2.133897000	-0.727813000	0.088686000
6	1.337599000	-1.456909000	0.979119000
6	-0.043548000	-1.334592000	0.970068000
6	3.606912000	-0.863958000	0.093049000
6	-4.967117000	-0.697279000	-0.037537000
7	-6.264237000	-0.722520000	-0.070272000
16	-4.180355000	-2.274482000	-0.062117000
6	-5.757201000	-3.011295000	-0.123889000

6	-6.746285000	-2.012846000	-0.118692000
6	-8.100497000	-2.368125000	-0.160122000
6	-6.097349000	-4.364847000	-0.169918000
6	-8.435604000	-3.708818000	-0.205721000
6	-7.440600000	-4.701320000	-0.210773000
6	4.437114000	0.240144000	-0.143303000
6	5.817466000	0.131059000	-0.143938000
6	6.432852000	-1.110571000	0.097522000
6	5.595085000	-2.219074000	0.336203000
6	4.218192000	-2.101948000	0.333881000
6	7.856449000	-1.396257000	0.129067000
6	9.021105000	-0.702838000	-0.024066000
6	10.227629000	-1.472268000	0.102041000
7	11.240004000	-2.030398000	0.190792000
6	9.232236000	0.750937000	-0.305687000
8	8.346639000	1.558911000	-0.439774000
8	10.511893000	1.153075000	-0.410395000
1	-6.022433000	1.699095000	-0.027805000
1	-1.067216000	4.184582000	0.179234000
1	-4.764934000	6.390653000	0.114861000
1	-6.029651000	4.265811000	0.030334000
1	-0.361409000	0.926073000	-1.537164000
1	2.078967000	0.681715000	-1.539246000
1	1.804602000	-2.117273000	1.702761000
1	-0.654483000	-1.908313000	1.658497000
1	-8.856684000	-1.590759000	-0.155562000
1	-5.332258000	-5.134143000	-0.174159000
1	-9.479889000	-4.001694000	-0.238244000
1	-7.728260000	-5.746964000	-0.247346000
1	3.991210000	1.215423000	-0.306634000
1	6.430428000	1.002304000	-0.319942000
1	8.037760000	-2.450383000	0.329384000
1	11.148849000	0.432405000	-0.287386000
1	3.606729000	-2.983233000	0.494113000
1	6.040706000	-3.193297000	0.516000000
8	-2.093653000	6.544245000	0.198578000
1	-2.659336000	7.325734000	0.203000000

Eelec = -2093.402836

$H_{(298.15\ K)}$ = -2092.929523

$G_{(298.15\ K)}$ = -2093.031532

ZPE (Zero-point energy correction) = 0.440356

B2-Me, *p*-phenylynyl-containing dye with an OH substituent at the 7-coumarin position and an *o*-methyl substituent at the phenylynyl unit at the ground state

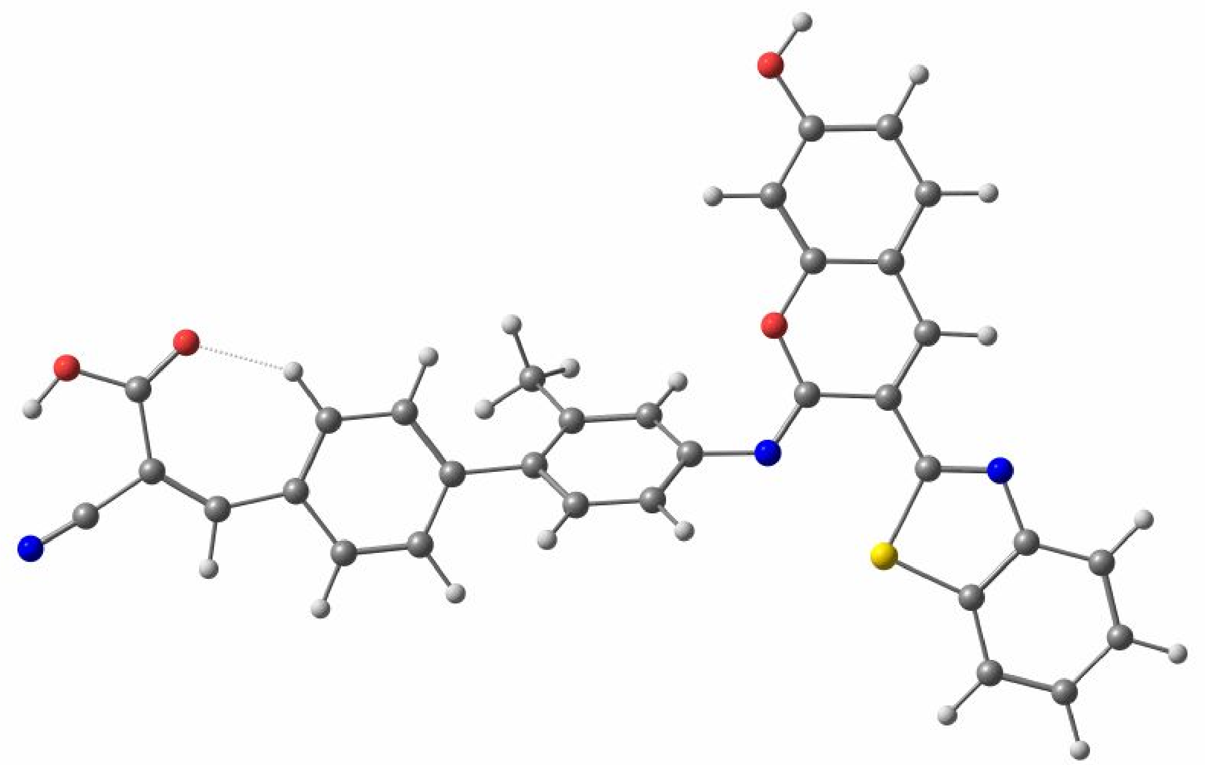

6	-5.012520000	1.710933000	-0.094219000
6	-4.310436000	0.553068000	-0.075036000
6	-2.848420000	0.602839000	-0.074922000
8	-2.253971000	1.841596000	-0.089720000
6	-2.959299000	3.006451000	-0.118377000
6	-2.232137000	4.184442000	-0.144937000
6	-2.927068000	5.387988000	-0.171528000
6	-4.328598000	5.409635000	-0.172091000
6	-5.028511000	4.217900000	-0.146291000
6	-4.358881000	2.987644000	-0.118907000
7	-2.138405000	-0.448264000	-0.079837000
6	-0.736389000	-0.465713000	-0.037536000
6	-0.062594000	-1.294967000	-0.935398000
6	1.318954000	-1.385386000	-0.883786000
6	2.068909000	-0.675261000	0.060721000
6	1.394039000	0.136355000	0.992909000
6	0.004986000	0.225553000	0.924084000
6	3.546241000	-0.827181000	0.048505000
6	-5.029428000	-0.731497000	-0.051304000
7	-6.326883000	-0.764014000	-0.055530000
16	-4.234645000	-2.304304000	-0.008498000

6	-5.808499000	-3.050022000	0.006033000
6	-6.802732000	-2.057121000	-0.024345000
6	-8.155543000	-2.420116000	-0.021885000
6	-6.142204000	-4.405585000	0.038826000
6	-8.484215000	-3.762796000	0.010852000
6	-7.484135000	-4.749741000	0.041124000
6	4.401570000	0.279845000	-0.012783000
6	5.778828000	0.138663000	-0.058813000
6	6.362788000	-1.140853000	-0.042559000
6	5.499756000	-2.253445000	0.014735000
6	4.125690000	-2.102426000	0.057664000
6	7.779704000	-1.461990000	-0.080601000
6	8.959714000	-0.780766000	-0.136354000
6	10.147525000	-1.588819000	-0.152884000
7	11.146210000	-2.177499000	-0.168769000
6	9.205857000	0.694402000	-0.180885000
8	8.340001000	1.534123000	-0.178784000
8	10.494699000	1.077474000	-0.225217000
1	-6.096262000	1.659837000	-0.092024000
1	-1.149321000	4.169520000	-0.148593000
1	-4.857949000	6.357613000	-0.193824000
1	-6.113921000	4.226264000	-0.147999000
1	-0.632695000	-1.855978000	-1.667569000
1	1.836484000	-2.013696000	-1.602250000
1	-0.518301000	0.835889000	1.652668000
1	-8.915700000	-1.646978000	-0.045639000
1	-5.373126000	-5.170560000	0.062296000
1	-9.527335000	-4.061600000	0.013279000
1	-7.766774000	-5.797090000	0.066729000
1	3.977458000	1.277203000	-0.047320000
1	6.412479000	1.011057000	-0.114547000
1	7.935618000	-2.538703000	-0.057277000
1	11.114414000	0.331698000	-0.223618000
1	3.488252000	-2.978451000	0.115987000
1	5.921487000	-3.254358000	0.031994000
8	-2.185989000	6.524528000	-0.197280000
1	-2.755455000	7.302934000	-0.219512000
6	2.111393000	0.891080000	2.086949000
1	2.970990000	0.337053000	2.469859000
1	2.480680000	1.860791000	1.737673000
1	1.432846000	1.084408000	2.920862000

$Eelec$ = -2132.694652

$H_{(298.15\ K)}$ = -2132.191836

$G_{(298.15\ K)}$ = -2132.297620
ZPE (Zero-point energy correction) = 0.468169

B3, *p*-phenylynyl-containing dye with an NH2 substituent at the 7-coumarin position at the ground state

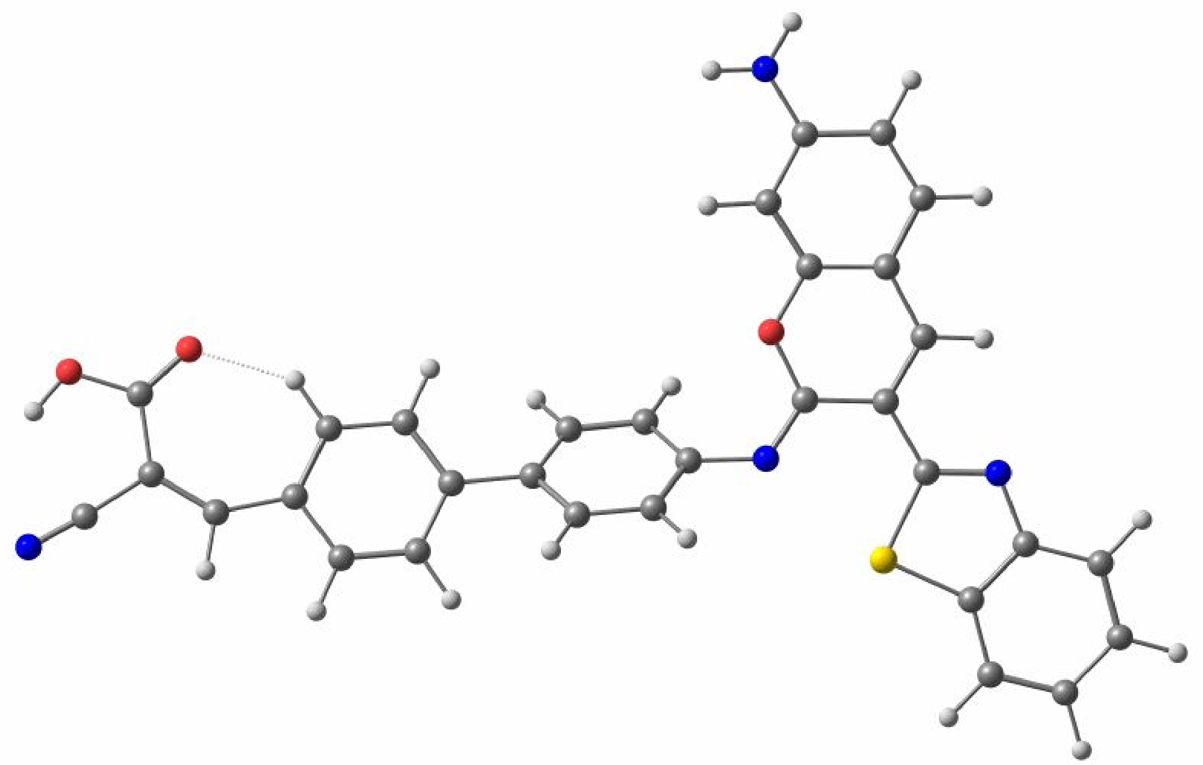

6	4.928587000	1.757793000	0.001985000
6	4.242196000	0.587195000	0.007554000
6	2.783495000	0.618452000	0.057990000
8	2.173719000	1.848581000	0.092757000
6	2.863824000	3.024183000	0.097374000
6	2.120550000	4.189929000	0.151635000
6	2.784465000	5.420432000	0.156709000
6	4.194688000	5.444323000	0.105340000
6	4.909509000	4.267910000	0.053779000
6	4.261815000	3.021847000	0.048972000
7	2.083411000	-0.441410000	0.089491000
6	0.683932000	-0.474081000	0.088512000
6	0.048413000	-1.328654000	0.994786000
6	-1.332698000	-1.452064000	1.002321000
6	-2.127332000	-0.744188000	0.093422000
6	-1.482606000	0.092034000	-0.826017000
6	-0.102831000	0.229137000	-0.832114000
6	-3.600039000	-0.879853000	0.097804000
6	4.978114000	-0.685463000	-0.040330000
7	6.275547000	-0.702023000	-0.077532000
16	4.202865000	-2.269650000	-0.060313000

6	5.785193000	-2.995021000	-0.126567000
6	6.766638000	-1.989012000	-0.126085000
6	8.123074000	-2.334766000	-0.171849000
6	6.135224000	-4.345866000	-0.172161000
6	8.468205000	-3.673195000	-0.216959000
6	7.480887000	-4.673034000	-0.217316000
6	-4.429360000	0.218082000	-0.169519000
6	-5.809648000	0.110585000	-0.169192000
6	-6.426830000	-1.123627000	0.104112000
6	-5.590288000	-2.226376000	0.372473000
6	-4.213326000	-2.110551000	0.369892000
6	-7.850471000	-1.406649000	0.139820000
6	-9.014162000	-0.714149000	-0.026244000
6	-10.221918000	-1.479602000	0.111435000
7	-11.235370000	-2.034473000	0.208198000
6	-9.223156000	0.735192000	-0.330166000
8	-8.336709000	1.540797000	-0.473808000
8	-10.502249000	1.137050000	-0.443595000
1	6.012303000	1.720307000	-0.037922000
1	1.038310000	4.129798000	0.188161000
1	4.712020000	6.398406000	0.103355000
1	5.994267000	4.294831000	0.017240000
1	0.657561000	-1.885704000	1.698344000
1	-1.801131000	-2.096052000	1.739738000
1	0.372941000	0.870956000	-1.564574000
1	8.873565000	-1.551850000	-0.170990000
1	5.375674000	-5.120707000	-0.172832000
1	9.514638000	-3.958076000	-0.252838000
1	7.775972000	-5.716640000	-0.253652000
1	-3.982089000	1.188066000	-0.358526000
1	-6.421563000	0.977598000	-0.368179000
1	-8.033570000	-2.456735000	0.358914000
1	-11.140046000	0.418622000	-0.311967000
1	-3.603023000	-2.987951000	0.553981000
1	-6.037128000	-3.195353000	0.576111000
1	-2.067988000	0.626035000	-1.567871000
7	2.072180000	6.598801000	0.170753000
1	2.557869000	7.446081000	0.418532000
1	1.102243000	6.569399000	0.442410000

$Eelec$ = -2073.539800
$H_{(298.15\ K)}$ = -2073.053723
$G_{(298.15\ K)}$ = -2073.156215
ZPE (Zero-point energy correction) = 0.452770

<u>B4, benzothiadiazole-containing dye with an CF3 substituent at the 7-coumarin position at the ground state</u>

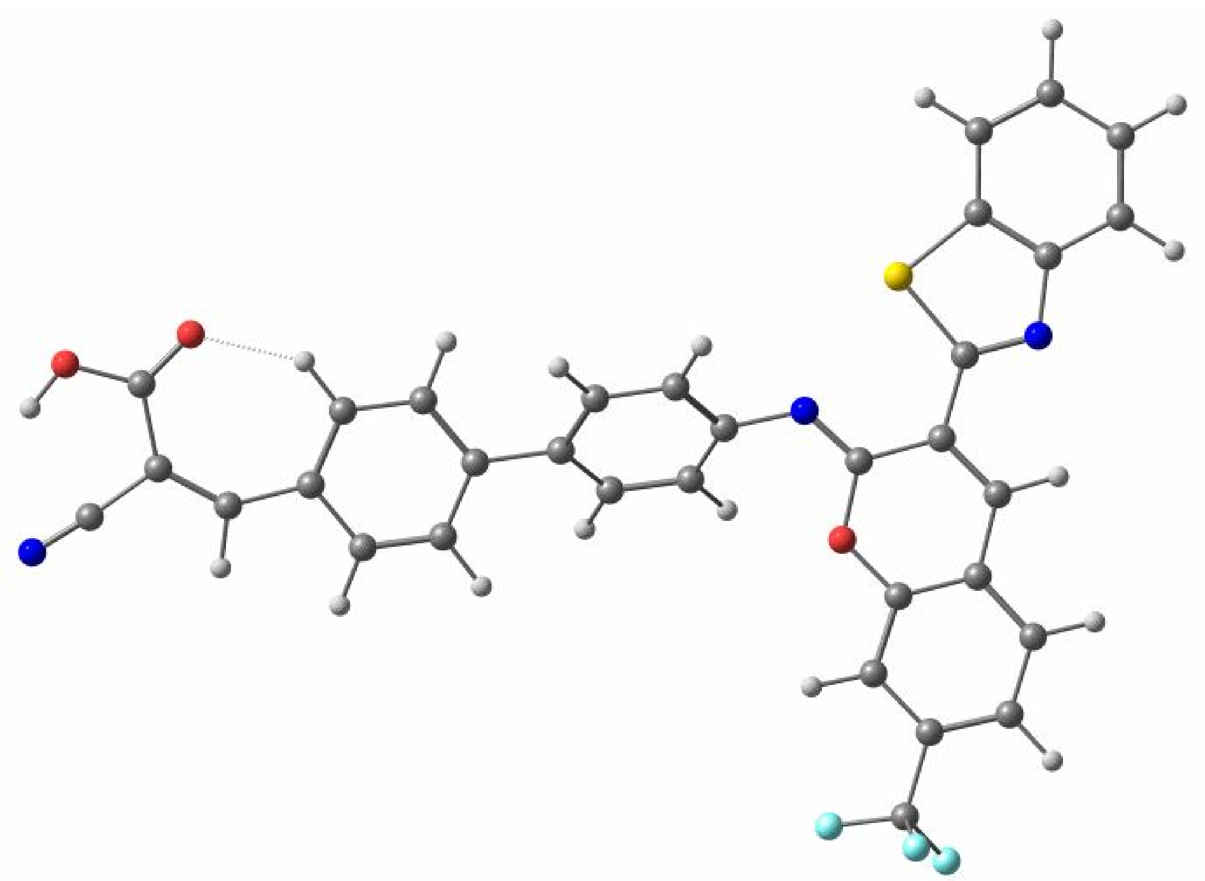

6	-4.990868000	0.457189000	0.013413000
6	-4.048758000	-0.512209000	0.001662000
6	-2.630685000	-0.140207000	0.004932000
8	-2.326355000	1.199129000	0.003513000
6	-3.276313000	2.175657000	0.028853000
6	-2.833799000	3.491634000	0.046346000
6	-3.780592000	4.504266000	0.066024000
6	-5.149411000	4.215328000	0.069781000
6	-5.567189000	2.897885000	0.053282000
6	-4.633566000	1.852129000	0.032699000
7	-1.711184000	-1.011668000	0.026341000
6	-0.336487000	-0.735275000	-0.007222000
6	0.488894000	-1.447614000	0.867280000
6	1.863212000	-1.264517000	0.847796000
6	2.461669000	-0.384240000	-0.060659000
6	1.629805000	0.306394000	-0.949490000

6	0.252986000	0.140065000	-0.926110000
6	3.929329000	-0.195984000	-0.086800000
6	-4.463430000	-1.926741000	-0.016410000
7	-5.722492000	-2.240444000	-0.013058000
16	-3.345121000	-3.286165000	-0.053805000
6	-4.717272000	-4.357000000	-0.068079000
6	-5.904885000	-3.605712000	-0.040915000
6	-7.146456000	-4.254883000	-0.043534000
6	-4.746338000	-5.753175000	-0.098047000
6	-7.173106000	-5.636327000	-0.073457000
6	-5.980748000	-6.380831000	-0.100676000
6	4.801257000	-1.269859000	0.136033000
6	6.176816000	-1.110550000	0.118064000
6	6.743254000	0.152800000	-0.129064000
6	5.863307000	1.230426000	-0.355225000
6	4.491470000	1.063415000	-0.334262000
6	8.155901000	0.489925000	-0.178719000
6	9.345936000	-0.160687000	-0.037636000
6	10.522656000	0.651149000	-0.180180000
7	11.513034000	1.245118000	-0.282458000
6	9.612521000	-1.605436000	0.246398000
8	8.757610000	-2.443717000	0.392239000
8	10.906430000	-1.960714000	0.339133000
1	-6.036339000	0.168514000	0.009934000
1	-1.771751000	3.703039000	0.043709000
1	-5.873408000	5.021794000	0.082085000
1	-6.625651000	2.658786000	0.054767000
1	0.032599000	-2.138724000	1.567599000
1	2.479024000	-1.803899000	1.560012000
1	-0.367681000	0.675448000	-1.635079000
1	-8.057024000	-3.666296000	-0.022181000
1	-3.829045000	-6.332052000	-0.119306000
1	-8.125276000	-6.156423000	-0.076084000
1	-6.028091000	-7.464589000	-0.124160000
1	4.393689000	-2.261119000	0.303264000
1	6.823275000	-1.959119000	0.284689000
1	8.296571000	1.549399000	-0.383330000
1	11.516263000	-1.218511000	0.206986000
1	3.846384000	1.922106000	-0.485526000
1	6.270822000	2.220250000	-0.539848000
1	2.067394000	0.967560000	-1.690647000
6	-3.341342000	5.940586000	0.123687000
9	-2.039216000	6.091400000	-0.180474000
9	-4.044862000	6.712380000	-0.729207000

9	-3.521859000	6.464911000	1.355295000

Eelec = -2355.177958
$H_{(298.15\ K)}$ = -2354.701667
$G_{(298.15\ K)}$ = -2354.811431
ZPE (Zero-point energy correction) = 0.440821

<u>B5, benzothiadiazole-containing dye with an NO2-substituent at the 7-coumarin position at the ground state</u>

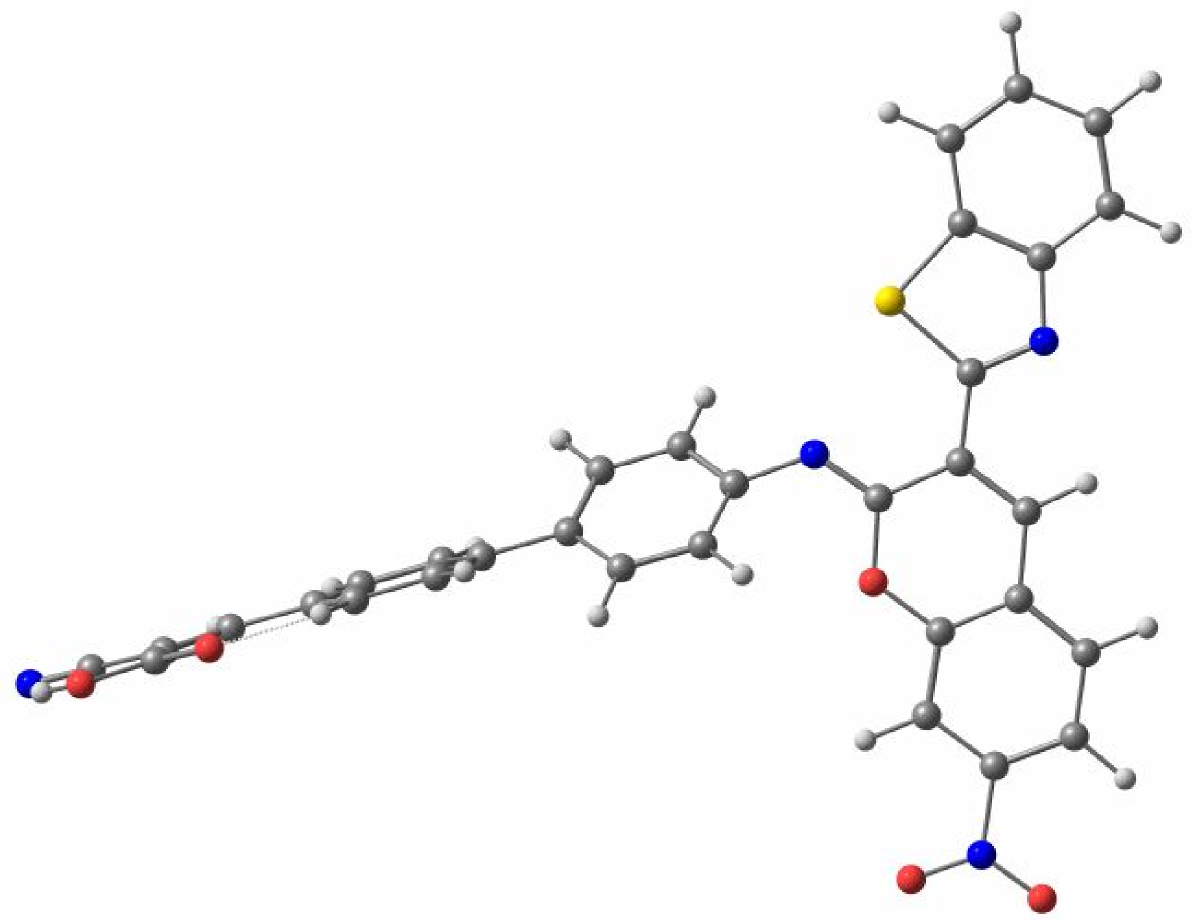

6	-4.753597000	4.805552000	0.086124000
6	-3.369235000	4.923119000	-0.004872000
6	-2.521292000	3.829946000	-0.059056000
6	-3.102229000	2.572187000	-0.019027000
6	-4.486564000	2.398351000	0.071417000
6	-5.302852000	3.536620000	0.123600000
8	-2.266511000	1.498015000	-0.069040000
6	-2.715881000	0.199570000	-0.057693000
6	-4.163603000	-0.015230000	0.041394000

6	-4.992907000	1.050389000	0.104194000
7	-1.903357000	-0.767231000	-0.148350000
6	-0.506753000	-0.651045000	-0.211298000
6	0.166548000	-1.460056000	-1.130849000
6	1.550380000	-1.433656000	-1.213099000
6	2.309338000	-0.628488000	-0.356317000
6	1.628337000	0.155006000	0.582227000
6	0.243093000	0.154363000	0.652874000
6	3.787122000	-0.613423000	-0.434071000
6	4.572979000	-0.525781000	0.722344000
6	5.956770000	-0.510870000	0.666108000
6	6.618597000	-0.585249000	-0.572508000
6	5.824732000	-0.673571000	-1.733764000
6	4.444177000	-0.687158000	-1.669276000
6	8.053158000	-0.580466000	-0.806451000
6	9.189717000	-0.527271000	-0.055331000
6	-4.728640000	-1.376263000	0.076128000
7	-6.012299000	-1.549116000	0.155593000
6	-6.342731000	-2.885874000	0.185030000
6	-5.246351000	-3.763602000	0.125641000
16	-3.768172000	-2.850482000	0.026802000
6	-7.645914000	-3.394238000	0.267333000
6	-7.823342000	-4.764403000	0.288893000
6	-6.721437000	-5.635930000	0.229398000
6	-5.428023000	-5.148476000	0.147232000
7	-2.773426000	6.267338000	-0.046199000
8	-1.556060000	6.346084000	-0.121217000
8	-3.532734000	7.224046000	-0.002517000
6	9.345013000	-0.448690000	1.430918000
8	8.428275000	-0.419202000	2.214168000
8	10.607701000	-0.410405000	1.892276000
6	10.425098000	-0.551520000	-0.788500000
7	11.457992000	-0.567590000	-1.314728000
1	-5.366232000	5.696303000	0.124673000
1	-1.448713000	3.952313000	-0.132599000
1	-6.378161000	3.410916000	0.193294000
1	-6.060948000	0.877506000	0.178472000
1	-0.414017000	-2.111479000	-1.774923000
1	2.049897000	-2.078508000	-1.928920000
1	2.189000000	0.798834000	1.252140000
1	-0.259180000	0.776880000	1.383804000
1	4.090460000	-0.493805000	1.693376000
1	6.536017000	-0.451717000	1.575384000
1	6.307337000	-0.722245000	-2.705679000

1	3.866390000	-0.729631000	-2.586210000
1	8.274395000	-0.635209000	-1.870478000
1	-8.484955000	-2.708723000	0.312575000
1	-8.824867000	-5.176616000	0.352721000
1	-6.887016000	-6.708079000	0.248379000
1	-4.581352000	-5.825241000	0.101983000
1	11.272694000	-0.438955000	1.187159000

Eelec = -2222.637489
$H_{(298.15\ K)}$ = -2222.164347
$G_{(298.15\ K)}$ = -2222.270759
ZPE (Zero-point energy correction) = 0.438781

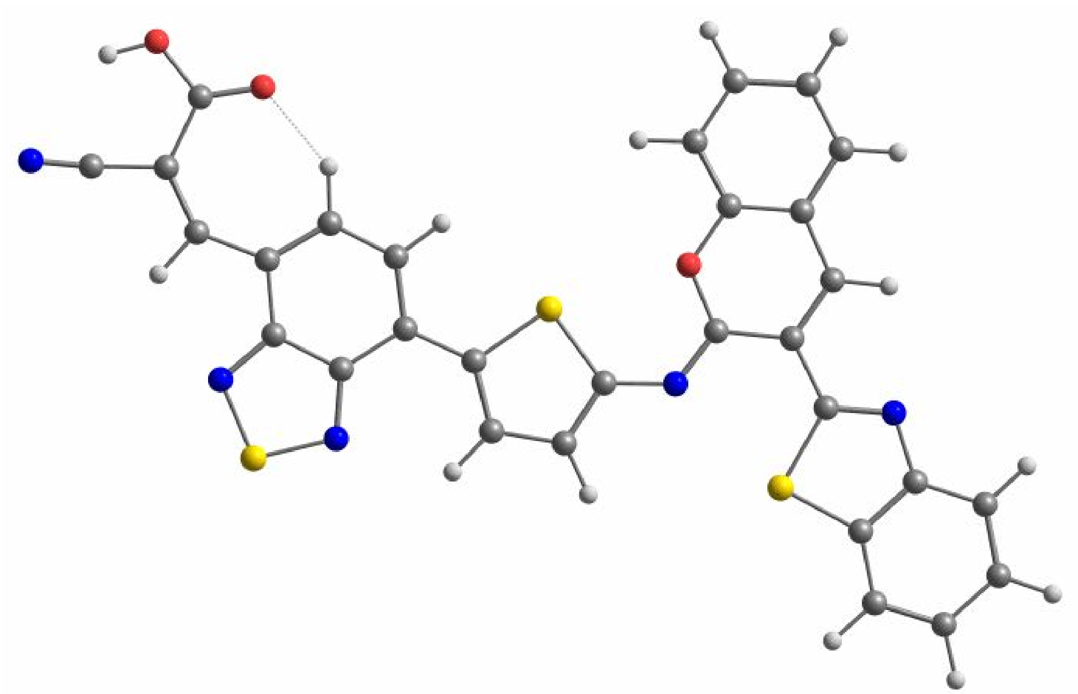

C1, benzothiadiazole- and thiophene-containing dye with a H at the 7-coumarin position at the ground state

6	4.767096000	2.391597000	0.000036000
6	4.367061000	1.096937000	0.000002000
6	2.941151000	0.795840000	0.000031000
8	2.064686000	1.842778000	0.000108000
6	2.462184000	3.151903000	0.000135000
6	1.472530000	4.125004000	0.000201000
6	1.855614000	5.458604000	0.000229000
6	3.208886000	5.813575000	0.000193000
6	4.181533000	4.829145000	0.000129000
6	3.820843000	3.473380000	0.000099000

7	2.501015000	-0.402087000	-0.000015000
6	-2.739523000	-0.947936000	0.000105000
6	5.381484000	0.026983000	-0.000061000
7	6.644879000	0.320566000	-0.000107000
16	5.007605000	-1.694461000	-0.000069000
6	6.716935000	-2.022682000	-0.000147000
6	7.430310000	-0.811845000	-0.000157000
6	8.830992000	-0.823235000	-0.000216000
6	7.379664000	-3.251907000	-0.000197000
6	9.485574000	-2.040775000	-0.000264000
6	8.764739000	-3.247454000	-0.000254000
6	-3.319217000	0.307538000	0.000036000
6	-4.710798000	0.543210000	0.000022000
6	-5.645259000	-0.476568000	0.000048000
6	-5.097401000	-1.812144000	0.000165000
6	-3.673200000	-2.049990000	0.000202000
7	-3.367562000	-3.344570000	0.000330000
7	-5.810202000	-2.933243000	0.000260000
16	-4.761996000	-4.176570000	0.000424000
6	-7.085402000	-0.381564000	-0.000110000
6	-8.009476000	0.623986000	-0.000221000
6	-9.385895000	0.215142000	-0.000495000
7	-10.518751000	-0.032037000	-0.000691000
6	-7.797304000	2.101728000	-0.000110000
8	-6.714636000	2.638169000	0.000324000
8	-8.906081000	2.862271000	-0.000528000
1	5.830293000	2.606057000	0.000014000
1	0.430333000	3.828019000	0.000229000
1	1.094023000	6.231066000	0.000279000
1	3.494468000	6.859593000	0.000215000
1	5.234837000	5.091691000	0.000101000
1	9.372645000	0.116248000	-0.000224000
1	6.827713000	-4.185911000	-0.000188000
1	10.570191000	-2.068720000	-0.000310000
1	9.301275000	-4.190572000	-0.000292000
1	-2.678812000	1.182461000	-0.000029000
1	-5.061096000	1.565439000	0.000003000
1	-7.538714000	-1.370104000	-0.000185000
1	-9.723488000	2.340182000	-0.000837000
6	0.813657000	-2.121750000	-0.000054000
6	1.189822000	-0.796771000	0.000003000
16	-0.226329000	0.239075000	0.000101000
6	-1.301403000	-1.138204000	0.000070000
6	-0.580435000	-2.314798000	-0.000016000

1	1.547264000	-2.917250000	-0.000120000
1	-1.055388000	-3.284726000	-0.000046000

Eelec = -2845.417793
H (298.15 K) = -2844.991739
G (298.15 K) = -2845.096138
ZPE (Zero-point energy correction) = 0.392694

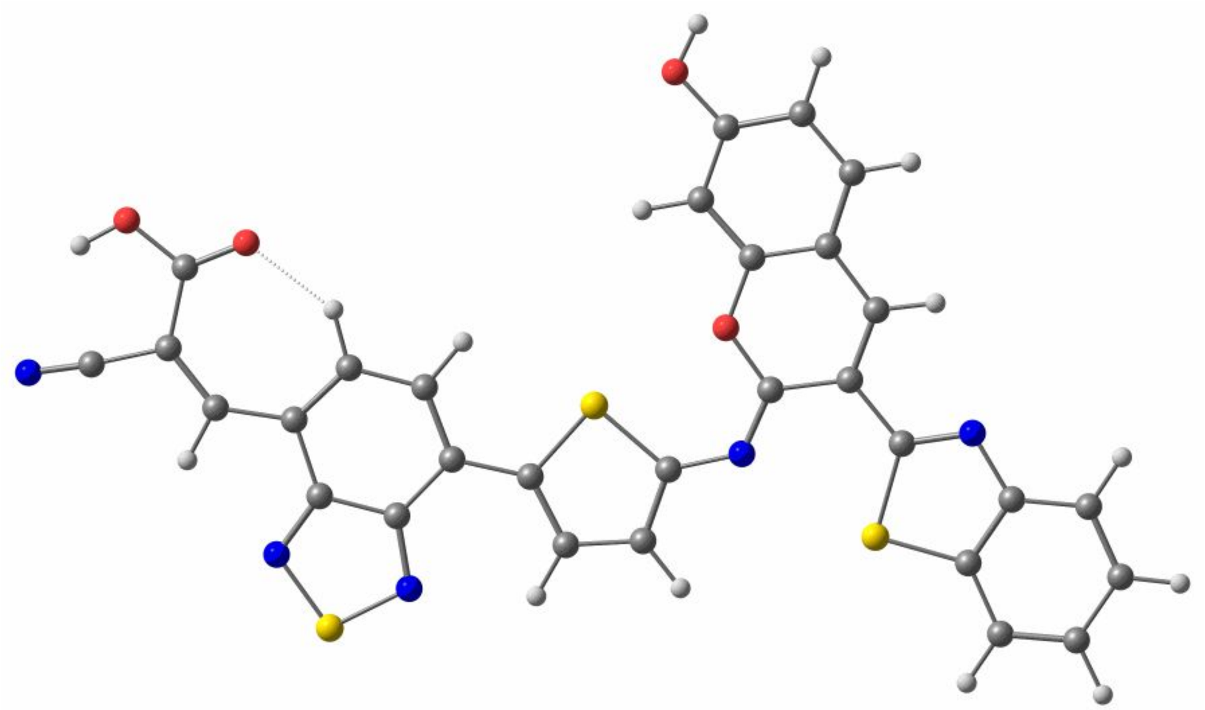

C2, benzothiadiazole- and thiophene-containing dye with an OH at the 7-coumarin position at the ground state

6	-4.757262000	2.195845000	-0.000067000
6	-4.352072000	0.900910000	-0.000027000
6	-2.926793000	0.607711000	-0.000026000
8	-2.053440000	1.661665000	-0.000071000
6	-2.454871000	2.966259000	-0.000111000
6	-1.466286000	3.934180000	-0.000152000
6	-1.854453000	5.269201000	-0.000192000
6	-3.211449000	5.623622000	-0.000190000
6	-4.175762000	4.634400000	-0.000150000
6	-3.819061000	3.278577000	-0.000109000
7	-2.475427000	-0.585900000	0.000017000
6	2.768776000	-1.097007000	0.000028000
6	-5.361984000	-0.171680000	0.000014000
7	-6.627161000	0.115180000	0.000011000

16	-4.979405000	-1.891749000	0.000067000
6	-6.687405000	-2.228872000	0.000092000
6	-7.406719000	-1.021590000	0.000055000
6	-8.807165000	-1.040629000	0.000065000
6	-7.343663000	-3.461346000	0.000140000
6	-9.455711000	-2.261676000	0.000112000
6	-8.728948000	-3.464516000	0.000149000
6	3.341123000	0.161986000	0.000107000
6	4.731332000	0.405703000	0.000110000
6	5.671896000	-0.608667000	0.000040000
6	5.131845000	-1.947444000	-0.000045000
6	3.709032000	-2.193622000	-0.000052000
7	3.411030000	-3.489951000	-0.000132000
7	5.851221000	-3.064322000	-0.000119000
16	4.810325000	-4.313858000	-0.000222000
6	7.111356000	-0.505887000	0.000046000
6	8.030453000	0.504316000	0.000101000
6	9.408845000	0.102340000	0.000081000
7	10.542942000	-0.139229000	0.000068000
6	7.810547000	1.980811000	0.000178000
8	6.725159000	2.511435000	0.000209000
8	8.915594000	2.747203000	0.000221000
1	-5.821842000	2.404140000	-0.000066000
1	-0.418155000	3.662832000	-0.000153000
1	-3.499005000	6.670792000	-0.000222000
1	-5.227648000	4.901788000	-0.000149000
1	-9.353860000	-0.104042000	0.000035000
1	-6.786696000	-4.392399000	0.000169000
1	-10.540209000	-2.294975000	0.000120000
1	-9.260454000	-4.410485000	0.000186000
1	2.695810000	1.033316000	0.000179000
1	5.075397000	1.430085000	0.000176000
1	7.569824000	-1.492046000	-0.000009000
1	9.735486000	2.229082000	0.000192000
6	-0.776606000	-2.293700000	0.000070000
6	-1.161632000	-0.971148000	0.000027000
16	0.247819000	0.073990000	-0.000011000
6	1.332046000	-1.296287000	0.000027000
6	0.618604000	-2.477564000	0.000069000
1	-1.505088000	-3.093899000	0.000100000
1	1.099788000	-3.444403000	0.000097000
8	-0.864834000	6.194949000	-0.000231000
1	-1.229348000	7.088261000	-0.000256000

Eelec = -2920.624674
$H_{(298.15\ K)}$ = -2920.193382
$G_{(298.15\ K)}$ = -2920.300493
ZPE (Zero-point energy correction) = 0.396659

C3, benzothiadiazole- and thiophene-containing dye with a NH2 at the 7-coumarin position at the ground state

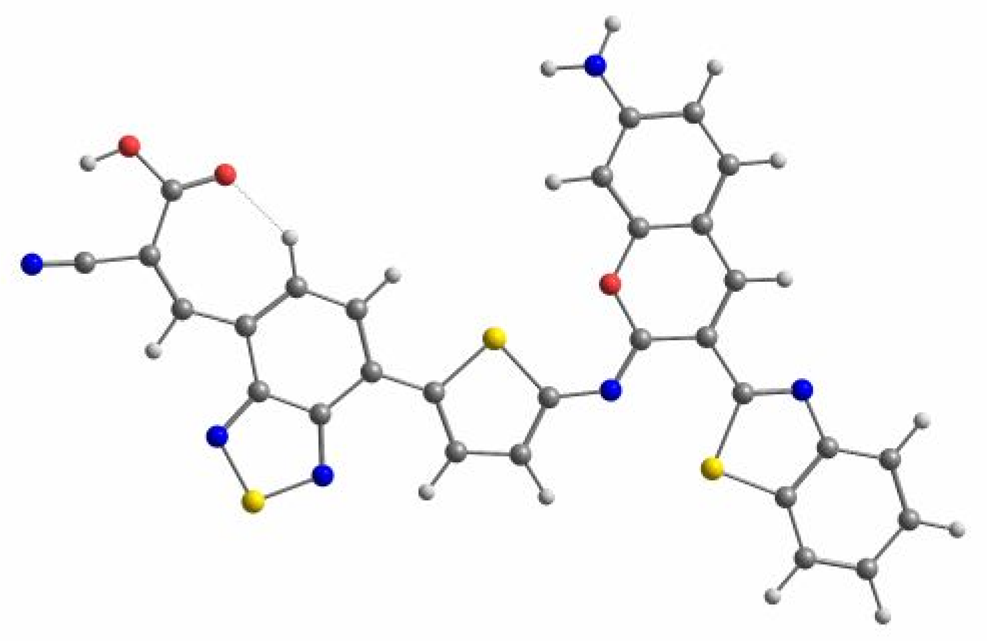

6	-4.741793000	2.206058000	-0.003823000
6	-4.348430000	0.904108000	-0.002586000
6	-2.928637000	0.600873000	-0.006983000
8	-2.047472000	1.647501000	-0.012814000
6	-2.437096000	2.956202000	-0.014566000
6	-1.439062000	3.912783000	-0.019135000
6	-1.796736000	5.265107000	-0.020057000
6	-3.165068000	5.617177000	-0.015540000
6	-4.136331000	4.642053000	-0.010344000
6	-3.798615000	3.277519000	-0.009784000
7	-2.481973000	-0.596989000	-0.005121000
6	2.759558000	-1.113721000	-0.006550000
6	-5.367563000	-0.157699000	0.003830000
7	-6.630900000	0.138269000	0.008177000
16	-4.998400000	-1.881754000	0.006157000
6	-6.709314000	-2.205725000	0.013503000
6	-7.418983000	-0.992728000	0.013681000
6	-8.819400000	-1.001341000	0.019146000
6	-7.375086000	-3.432857000	0.018725000
6	-9.477688000	-2.217438000	0.024302000

6	-8.760582000	-3.425791000	0.024096000
6	3.331206000	0.146821000	0.000544000
6	4.719605000	0.392233000	0.003435000
6	5.663010000	-0.620557000	-0.001027000
6	5.125096000	-1.960233000	-0.009554000
6	3.702430000	-2.208576000	-0.012032000
7	3.406157000	-3.505103000	-0.020086000
7	5.845918000	-3.076068000	-0.015637000
16	4.806533000	-4.327199000	-0.023973000
6	7.100687000	-0.512445000	-0.000261000
6	8.016352000	0.501988000	0.012914000
6	9.396098000	0.105474000	0.006263000
7	10.531303000	-0.131172000	0.002075000
6	7.791799000	1.976841000	0.035320000
8	6.705096000	2.505886000	0.044122000
8	8.894577000	2.746818000	0.046930000
1	-5.804966000	2.422232000	-0.000176000
1	-0.400512000	3.601760000	-0.026595000
1	-3.444494000	6.665837000	-0.021277000
1	-5.185131000	4.922302000	-0.006475000
1	-9.358884000	-0.060556000	0.019159000
1	-6.825179000	-4.368146000	0.018540000
1	-10.562452000	-2.242020000	0.028509000
1	-9.299250000	-4.367714000	0.028171000
1	2.683762000	1.016418000	0.004682000
1	5.062825000	1.416818000	0.010075000
1	7.563436000	-1.496579000	-0.012837000
1	9.715887000	2.230912000	0.038349000
6	-0.785412000	-2.308092000	-0.006656000
6	-1.170649000	-0.984394000	-0.006913000
16	0.241224000	0.058755000	-0.008689000
6	1.324963000	-1.312878000	-0.007923000
6	0.608838000	-2.493341000	-0.007416000
1	-1.514823000	-3.107393000	-0.005687000
1	1.088687000	-3.460967000	-0.007292000
7	-0.832827000	6.242150000	-0.062292000
1	-1.092255000	7.190656000	0.155200000
1	0.123096000	5.995820000	0.138365000

$Eelec$ = -2900.7623314
$H_{(298.15\ K)}$ = -2900.318296
$G_{(298.15\ K)}$ = -2900.425356
ZPE (Zero-point energy correction) = 0.409023

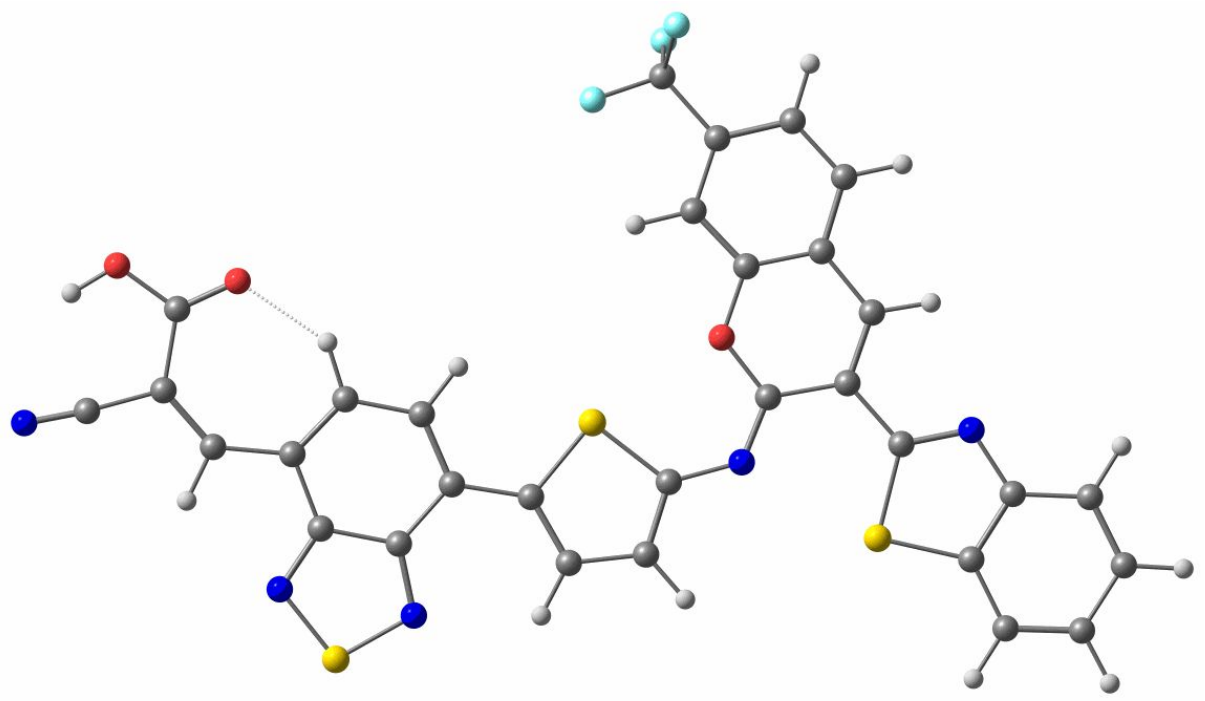

C4, benzothiadiazole- and thiophene-containing dye with a CF3 at the 7-coumarin position at the ground state

6	-4.762639000	1.628471000	-0.004942000
6	-4.326674000	0.346743000	-0.008736000
6	-2.891766000	0.081905000	-0.006486000
8	-2.041852000	1.155131000	-0.000062000
6	-2.477492000	2.449060000	0.004661000
6	-1.512532000	3.447008000	0.011066000
6	-1.935536000	4.766865000	0.013893000
6	-3.296947000	5.091061000	0.011139000
6	-4.238998000	4.080645000	0.005278000
6	-3.842739000	2.735013000	0.002080000
7	-2.421387000	-1.101654000	-0.010213000
6	2.831296000	-1.539214000	-0.005392000
6	-5.311921000	-0.750791000	-0.014633000
7	-6.582062000	-0.488036000	-0.017078000
16	-4.895272000	-2.461296000	-0.017795000
6	-6.595431000	-2.831829000	-0.022551000
6	-7.339077000	-1.639203000	-0.021582000
6	-8.739245000	-1.685045000	-0.024901000
6	-7.227224000	-4.077425000	-0.026775000
6	-9.362800000	-2.918377000	-0.029049000
6	-8.611731000	-4.106812000	-0.029973000
6	3.386283000	-0.274567000	-0.044198000
6	4.774592000	-0.012366000	-0.045173000
6	5.726353000	-1.014057000	-0.008340000

6	5.203369000	-2.358832000	0.032703000
6	3.784155000	-2.623238000	0.034368000
7	3.502712000	-3.922786000	0.076677000
7	5.937019000	-3.465768000	0.072865000
16	4.912635000	-4.727667000	0.109894000
6	7.166297000	-0.894558000	-0.005041000
6	8.072324000	0.125450000	-0.035890000
6	9.455798000	-0.259248000	-0.014884000
7	10.592479000	-0.487162000	-0.000673000
6	7.833982000	1.599194000	-0.089126000
8	6.741490000	2.113787000	-0.113808000
8	8.928581000	2.378840000	-0.110172000
1	-5.830844000	1.815173000	-0.006832000
1	-0.462120000	3.184830000	0.011010000
1	-3.605710000	6.130129000	0.010908000
1	-5.297819000	4.317756000	0.001751000
1	-9.304091000	-0.759363000	-0.024098000
1	-6.652412000	-4.997468000	-0.027471000
1	-10.446304000	-2.973735000	-0.031597000
1	-9.124782000	-5.062864000	-0.033219000
1	2.730395000	0.588262000	-0.078183000
1	5.105311000	1.015886000	-0.077540000
1	7.635820000	-1.874762000	0.031390000
1	9.755647000	1.872755000	-0.087380000
6	-0.698823000	-2.784893000	-0.010784000
6	-1.100970000	-1.468719000	-0.008943000
16	0.292918000	-0.403529000	-0.003212000
6	1.395137000	-1.758209000	-0.004373000
6	0.699735000	-2.949209000	-0.007635000
1	-1.415812000	-3.595457000	-0.014297000
1	1.194958000	-3.908870000	-0.007806000
6	-0.928775000	5.883174000	0.047395000
9	-0.950966000	6.531200000	1.231719000
9	0.328386000	5.447212000	-0.142438000
9	-1.182065000	6.807744000	-0.901125000

Eelec = -3182.399179
$H_{(298.15\ K)}$ = -3181.964846
$G_{(298.15\ K)}$ = -3182.079492
ZPE (Zero-point energy correction) = 0.397219

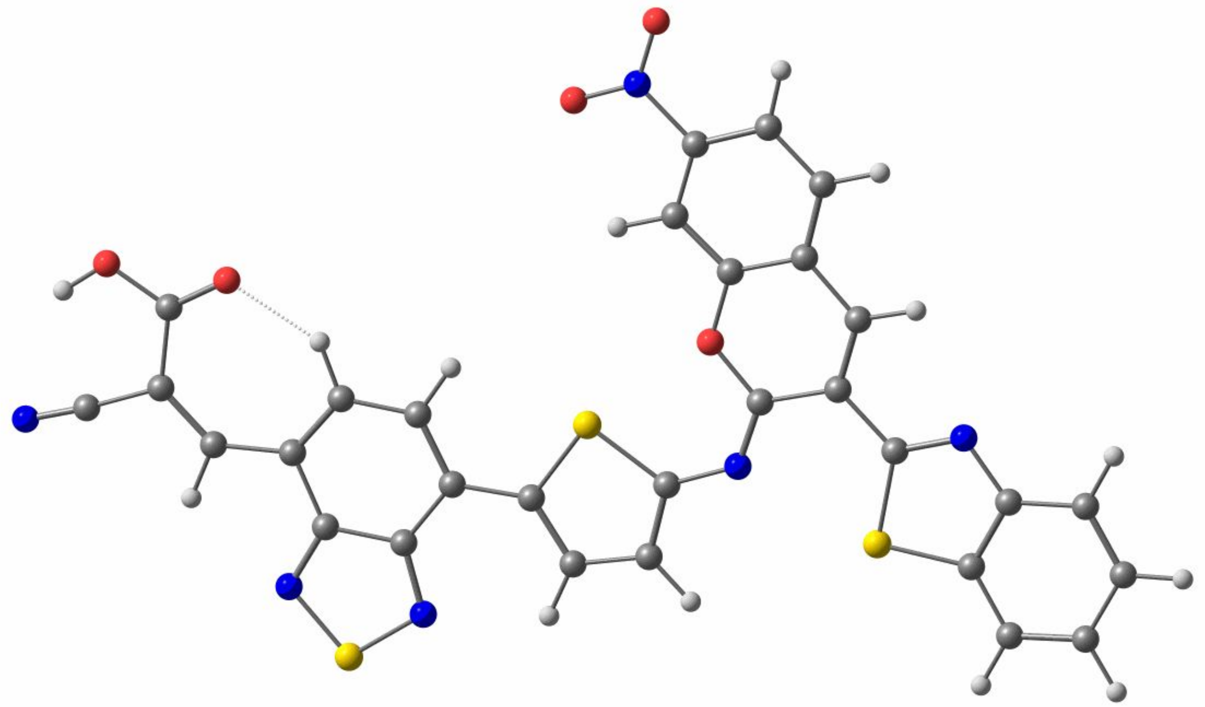

C5, benzothiadiazole- and thiophene-containing dye with a NO2 at the 7-coumarin position at the ground state

6	-4.777707000	1.847799000	-0.000059000
6	-4.342938000	0.565895000	-0.000019000
6	-2.907833000	0.297608000	-0.000006000
8	-2.054637000	1.369802000	-0.000043000
6	-2.487923000	2.663724000	-0.000082000
6	-1.523798000	3.658385000	-0.000114000
6	-1.967607000	4.969801000	-0.000154000
6	-3.316743000	5.313996000	-0.000164000
6	-4.254134000	4.297216000	-0.000132000
6	-3.855731000	2.952938000	-0.000091000
7	-2.441357000	-0.886211000	0.000036000
6	2.809671000	-1.352828000	0.000091000
6	-5.329742000	-0.530227000	0.000011000
7	-6.599216000	-0.263579000	-0.000004000
16	-4.917899000	-2.241347000	0.000063000
6	-6.618787000	-2.606964000	0.000068000
6	-7.359364000	-1.412267000	0.000027000
6	-8.759850000	-1.454024000	0.000022000
6	-7.254014000	-3.850935000	0.000104000
6	-9.386510000	-2.685520000	0.000058000
6	-8.638444000	-3.876096000	0.000098000
6	3.372689000	-0.091809000	0.000182000
6	4.763551000	0.161055000	0.000197000

6	5.707607000	-0.847920000	0.000129000
6	5.175317000	-2.189623000	0.000029000
6	3.754458000	-2.444304000	0.000010000
7	3.464287000	-3.742718000	-0.000085000
7	5.901523000	-3.302240000	-0.000051000
16	4.868857000	-4.557631000	-0.000163000
6	7.149365000	-0.740084000	0.000148000
6	8.063034000	0.272818000	0.000230000
6	9.443596000	-0.123261000	0.000173000
7	10.578329000	-0.360827000	0.000119000
6	7.835680000	1.749779000	0.000362000
8	6.746762000	2.271845000	-0.000118000
8	8.935850000	2.521398000	-0.000282000
1	-5.845437000	2.036459000	-0.000068000
1	-0.468068000	3.422239000	-0.000107000
1	-3.603538000	6.357146000	-0.000196000
1	-5.313086000	4.532616000	-0.000139000
1	-9.322113000	-0.526796000	-0.000009000
1	-6.681902000	-4.772620000	0.000135000
1	-10.470116000	-2.738165000	0.000054000
1	-9.154242000	-4.830653000	0.000126000
1	2.723209000	0.776603000	0.000256000
1	5.100986000	1.187661000	0.000276000
1	7.610831000	-1.724735000	0.000091000
1	9.759397000	2.009146000	-0.000345000
6	-0.728192000	-2.578137000	0.000097000
6	-1.122304000	-1.259953000	0.000057000
16	0.277032000	-0.202264000	0.000035000
6	1.371015000	-1.562972000	0.000078000
6	0.669652000	-2.750208000	0.000108000
1	-1.449564000	-3.384845000	0.000117000
1	1.159850000	-3.712375000	0.000135000
7	-0.961938000	6.043506000	-0.000190000
8	0.215369000	5.716634000	-0.000183000
8	-1.367036000	7.196494000	-0.000226000

$Eelec$ = -3049.858151
$H_{(298.15\ K)}$ = -3049.426952
$G_{(298.15\ K)}$ = -3049.539121
ZPE (Zero-point energy correction) = 0.395184

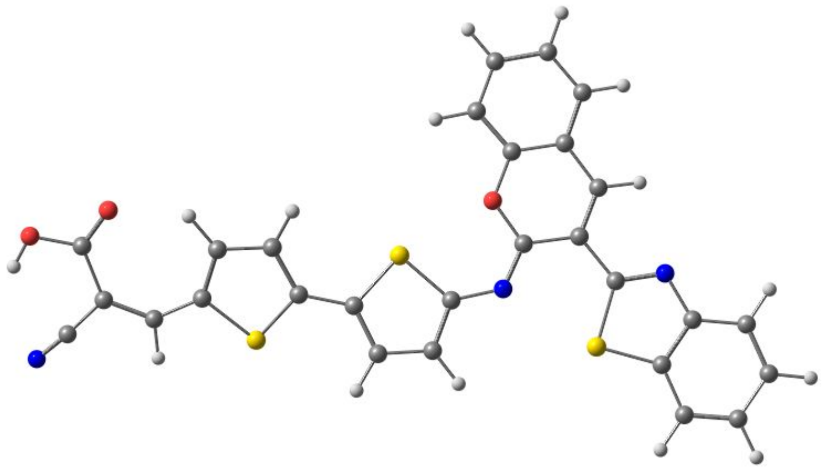

D1, oligothyophene-containing dye with a H at the 7-coumarin position at the ground state

6	2.224924000	3.088496000	-0.072171000
6	1.312384000	4.133876000	-0.094949000
6	1.795698000	5.434165000	-0.130583000
6	3.171797000	5.685155000	-0.143322000
6	4.066691000	4.629574000	-0.120758000
6	3.603996000	3.305799000	-0.084799000
6	4.465988000	2.155501000	-0.060458000
6	3.969273000	0.895527000	-0.027311000
7	1.993968000	-0.456551000	0.014826000
6	2.524443000	0.702795000	-0.015068000
8	1.728768000	1.813694000	-0.036946000
6	0.655885000	-0.748811000	0.031204000
6	-1.847193000	-0.890128000	0.065321000
6	-1.229202000	-2.119037000	0.069457000
6	0.177790000	-2.039547000	0.050848000
6	-5.772518000	-0.713791000	-0.002952000
6	-5.285728000	0.549521000	0.297213000
6	-3.885019000	0.606931000	0.347446000
6	-3.264209000	-0.602838000	0.088276000
16	-4.434059000	-1.827560000	-0.234501000
6	-7.078485000	-1.267838000	-0.156173000
6	-8.351388000	-0.768369000	-0.081882000
6	4.900003000	-0.247762000	-0.004068000
16	4.397354000	-1.935683000	0.042202000
6	6.076928000	-2.392256000	0.045297000
6	6.879558000	-1.239160000	0.011015000
7	6.181921000	-0.050976000	-0.016082000
6	6.645078000	-3.667621000	0.075557000
6	8.026530000	-3.767754000	0.070977000
6	8.836239000	-2.619350000	0.036703000
6	8.275315000	-1.356259000	0.006652000
6	-8.760613000	0.630588000	0.211303000
8	-7.985739000	1.530088000	0.435786000

8	-10.083502000	0.879829000	0.220123000
6	-9.419951000	-1.693539000	-0.313184000
7	-10.335509000	-2.383677000	-0.489384000
1	0.250572000	3.917437000	-0.085465000
1	3.536099000	6.706046000	-0.171161000
1	5.136926000	4.810849000	-0.130707000
1	5.542288000	2.289013000	-0.069014000
1	-1.774641000	-3.055027000	0.101283000
1	0.847085000	-2.889739000	0.056574000
1	-5.944407000	1.385128000	0.478394000
1	-3.334630000	1.508858000	0.585346000
1	-7.060525000	-2.331332000	-0.387976000
1	6.024288000	-4.556965000	0.102094000
1	8.490421000	-4.748361000	0.094248000
1	9.915643000	-2.729085000	0.033905000
1	8.886183000	-0.460631000	-0.019987000
1	-10.616192000	0.092501000	0.029180000
16	-0.678185000	0.397340000	0.030889000
1	1.094979000	6.262060000	-0.148921000

Eelec = -2659.788596

$H_{(298.15\ K)}$ = -2659.388799

$G_{(298.15\ K)}$ = -2659.487103

ZPE (Zero-point energy correction) = 0.368639

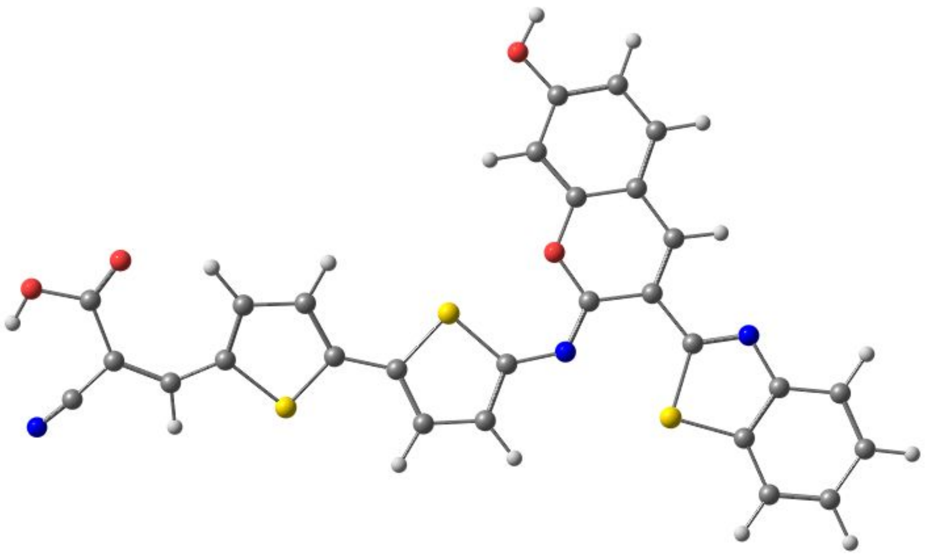

<u>D2, oligothiophene-containing dye with an OH at the 7-coumarin position at the ground state</u>

6	2.214600000	2.886938000	-0.053460000
6	1.302721000	3.927291000	-0.070650000
6	1.791365000	5.228634000	-0.098892000
6	3.171044000	5.478784000	-0.109932000
6	4.057250000	4.418850000	-0.092821000
6	3.598449000	3.094611000	-0.064145000
6	4.452227000	1.943649000	-0.045483000
6	3.950208000	0.683750000	-0.018799000
7	1.964967000	-0.655206000	0.016438000
6	2.506495000	0.499000000	-0.008066000
8	1.714708000	1.616868000	-0.025420000
6	0.624891000	-0.937478000	0.030893000
6	-1.879404000	-1.060991000	0.061670000
6	-1.270015000	-2.294279000	0.063426000
6	0.137442000	-2.224882000	0.046514000
6	-5.803530000	-0.859457000	-0.004352000
6	-5.308031000	0.403072000	0.285382000
6	-3.907048000	0.451907000	0.333615000
6	-3.294251000	-0.764017000	0.083451000
16	-4.472405000	-1.983732000	-0.228351000
6	-7.112809000	-1.406156000	-0.152737000
6	-8.382863000	-0.898885000	-0.078803000
6	4.875975000	-0.462133000	-0.000901000
16	4.364218000	-2.148117000	0.036327000
6	6.041663000	-2.613917000	0.037816000
6	6.850297000	-1.464881000	0.010009000
7	6.159203000	-0.272475000	-0.011228000

6	6.602742000	-3.892338000	0.061672000
6	7.983796000	-4.000473000	0.057278000
6	8.799470000	-2.856415000	0.029464000
6	8.245208000	-1.589984000	0.005770000
6	-8.783297000	0.503513000	0.209037000
8	-8.002885000	1.399727000	0.427273000
8	-10.104959000	0.760200000	0.220575000
6	-9.457050000	-1.818958000	-0.303843000
7	-10.376839000	-2.504755000	-0.475147000
1	0.236953000	3.736823000	-0.063189000
1	3.537538000	6.500759000	-0.132053000
1	5.126449000	4.605068000	-0.101512000
1	5.529462000	2.070829000	-0.052932000
1	-1.822058000	-3.226523000	0.091683000
1	0.800708000	-3.079793000	0.050510000
1	-5.960920000	1.244517000	0.460472000
1	-3.350788000	1.352544000	0.562657000
1	-7.101627000	-2.470929000	-0.379071000
1	5.976901000	-4.778303000	0.083207000
1	8.442149000	-4.983790000	0.075619000
1	9.878321000	-2.971766000	0.026642000
1	8.861165000	-0.697680000	-0.015832000
1	-10.642309000	-0.025114000	0.034559000
16	-0.701008000	0.218172000	0.032337000
8	0.874651000	6.227091000	-0.114930000
1	1.306490000	7.089633000	-0.134865000

Eelec = -2734.995552

$H_{(298.15\ K)}$ = -2734.590515

$G_{(298.15\ K)}$ = -2734.691519

ZPE (Zero-point energy correction) = 0.372609

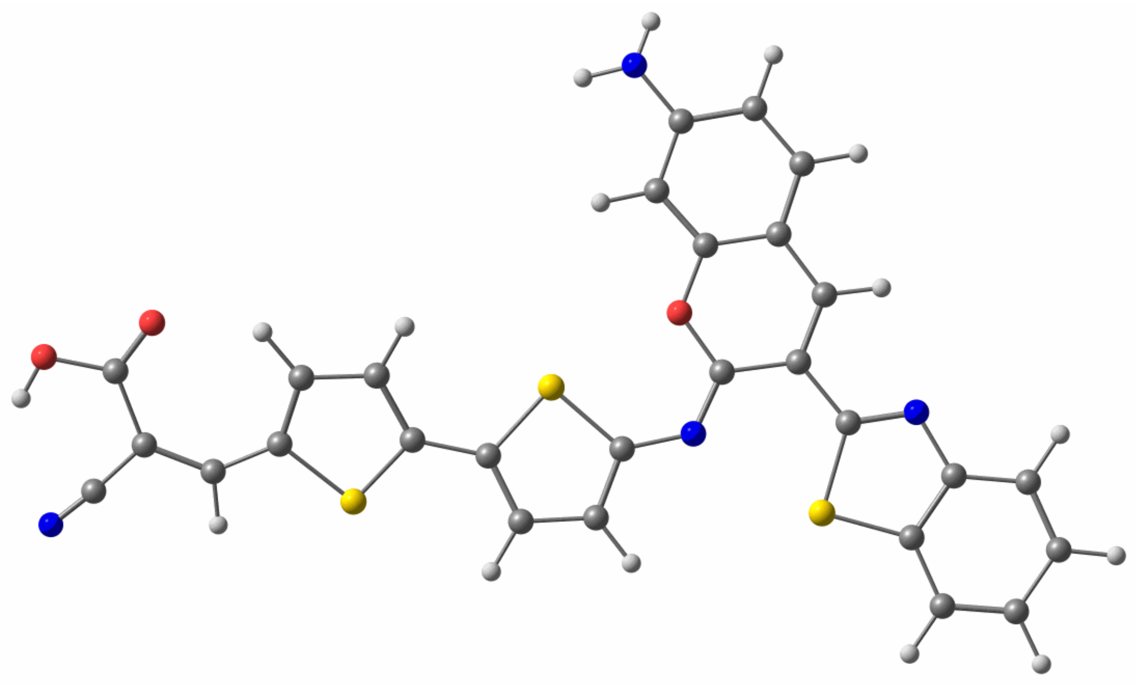

D3, oligothiophene-containing dye with a NH2 at the 7-coumarin position at the ground state

6	2.202392000	2.880674000	-0.053218000
6	1.282291000	3.912371000	-0.074905000
6	1.744422000	5.232181000	-0.104923000
6	3.135379000	5.476745000	-0.112156000
6	4.027683000	4.428599000	-0.091595000
6	3.584568000	3.095293000	-0.061872000
6	4.442552000	1.953632000	-0.042416000
6	3.949690000	0.686942000	-0.017332000
7	1.971827000	-0.664042000	0.013420000
6	2.509945000	0.493926000	-0.008464000
8	1.711365000	1.606454000	-0.025124000
6	0.633754000	-0.949066000	0.026909000
6	-1.872496000	-1.077685000	0.061677000
6	-1.259917000	-2.309520000	0.051416000
6	0.147065000	-2.237551000	0.032792000
6	-5.796893000	-0.874605000	-0.000676000
6	-5.299849000	0.382706000	0.310187000
6	-3.899525000	0.429442000	0.359919000
6	-3.286579000	-0.782837000	0.089328000
16	-4.465925000	-1.996142000	-0.242977000
6	-7.106547000	-1.416165000	-0.156061000
6	-8.375927000	-0.905964000	-0.079338000
6	4.883606000	-0.450501000	0.000333000
16	4.382349000	-2.140739000	0.035616000

6	6.062998000	-2.596243000	0.038358000
6	6.864231000	-1.441973000	0.012347000
7	6.166032000	-0.253602000	-0.008586000
6	6.632017000	-3.870955000	0.061652000
6	8.013915000	-3.970977000	0.058536000
6	8.822150000	-2.821883000	0.032521000
6	8.259744000	-1.558767000	0.009377000
6	-8.773221000	0.494703000	0.218162000
8	-7.991541000	1.388163000	0.444695000
8	-10.094374000	0.754918000	0.228315000
6	-9.452117000	-1.821001000	-0.314168000
7	-10.373722000	-2.502475000	-0.493188000
1	0.222487000	3.684093000	-0.064821000
1	3.495746000	6.500278000	-0.129839000
1	5.095193000	4.625773000	-0.098792000
1	5.519189000	2.087072000	-0.048292000
1	-1.809994000	-3.243188000	0.073186000
1	0.811843000	-3.091241000	0.028965000
1	-5.952680000	1.221145000	0.499338000
1	-3.341922000	1.324503000	0.606542000
1	-7.098044000	-2.478680000	-0.392932000
1	6.011523000	-4.760754000	0.081861000
1	8.478233000	-4.951519000	0.076527000
1	9.901771000	-2.930212000	0.030706000
1	8.870194000	-0.662628000	-0.010771000
1	-10.633109000	-0.027914000	0.035812000
16	-0.695564000	0.204066000	0.040522000
7	0.858422000	6.282732000	-0.089101000
1	-0.110405000	6.104640000	-0.300212000
1	1.192719000	7.199994000	-0.337108000

$Eelec$ = -2715.132903

$H_{(298.15\ K)}$ = -2714.715115

$G_{(298.15\ K)}$ = -2714.816547

ZPE (Zero-point energy correction) = 0.384987

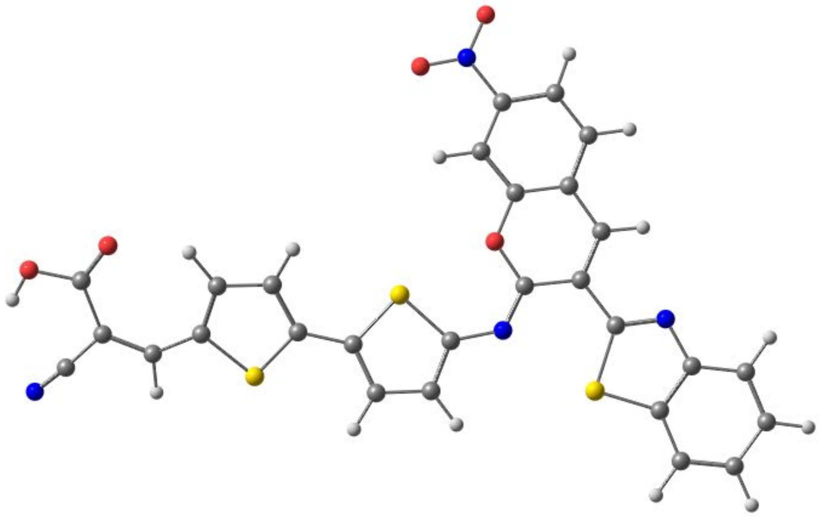

D5, oligothiophene-containing dye with a NO2 at the 7-coumarin position at the ground state

6	2.240275000	2.551296000	-0.038234000
6	1.354102000	3.615696000	-0.050857000
6	1.895532000	4.889777000	-0.077621000
6	3.266681000	5.130992000	-0.092238000
6	4.124563000	4.046163000	-0.079254000
6	3.625921000	2.736096000	-0.052016000
6	4.462089000	1.564403000	-0.037377000
6	3.932011000	0.319251000	-0.011706000
7	1.924734000	-0.983720000	0.024881000
6	2.480832000	0.159894000	0.001767000
8	1.710275000	1.293996000	-0.011701000
6	0.580462000	-1.252526000	0.040434000
6	-1.921832000	-1.350513000	0.072283000
6	-1.326297000	-2.590233000	0.075638000
6	0.082270000	-2.534705000	0.057962000
6	-5.842828000	-1.114199000	-0.008461000
6	-5.341324000	0.135528000	0.318987000
6	-3.938797000	0.172692000	0.375123000
6	-3.335727000	-1.039566000	0.094101000
16	-4.520432000	-2.241117000	-0.258731000
6	-7.156532000	-1.649766000	-0.180188000
6	-8.422372000	-1.138396000	-0.093868000
6	4.833221000	-0.848179000	0.002213000
16	4.293221000	-2.523217000	0.035657000
6	5.961511000	-3.016581000	0.033081000
6	6.790186000	-1.881507000	0.007186000
7	6.119129000	-0.678744000	-0.009632000
6	6.500963000	-4.304855000	0.052473000

6	7.879498000	-4.434670000	0.045572000
6	8.715293000	-3.304270000	0.019684000
6	8.183443000	-2.029098000	0.000403000
6	-8.816055000	0.256996000	0.241552000
8	-8.029619000	1.138401000	0.493092000
8	-10.135319000	0.520872000	0.258130000
6	-9.501353000	-2.044351000	-0.353615000
7	-10.423897000	-2.718974000	-0.551052000
1	0.283632000	3.459827000	-0.040839000
1	3.631438000	6.149273000	-0.113186000
1	5.198237000	4.200795000	-0.090024000
1	5.540931000	1.672150000	-0.047092000
1	-1.888343000	-3.516162000	0.106996000
1	0.736835000	-3.396379000	0.063207000
1	-5.988458000	0.976737000	0.515093000
1	-3.377974000	1.062760000	0.633009000
1	-7.149591000	-2.706479000	-0.441494000
1	5.860879000	-5.180472000	0.072511000
1	8.321671000	-5.425360000	0.060407000
1	9.791798000	-3.438677000	0.014870000
1	8.814086000	-1.147176000	-0.019654000
1	-10.678056000	-0.253210000	0.042910000
16	-0.732625000	-0.083530000	0.040012000
7	0.973555000	6.035857000	-0.090906000
8	-0.225142000	5.798728000	-0.073037000
8	1.463708000	7.154883000	-0.118844000

Eelec = -2864.229335

$H_{(298.15\ K)}$ = -2863.824408

$G_{(298.15\ K)}$ = -2863.929566

ZPE (Zero-point energy correction) = 0.371113

Gas-phase first singlet excited state optimized geometries for dye series A-D

First singlet excited state (S_1) A1*, benzothiadiazole-containing dye with a H atom at the 7-coumarin position at the first singlet excited state

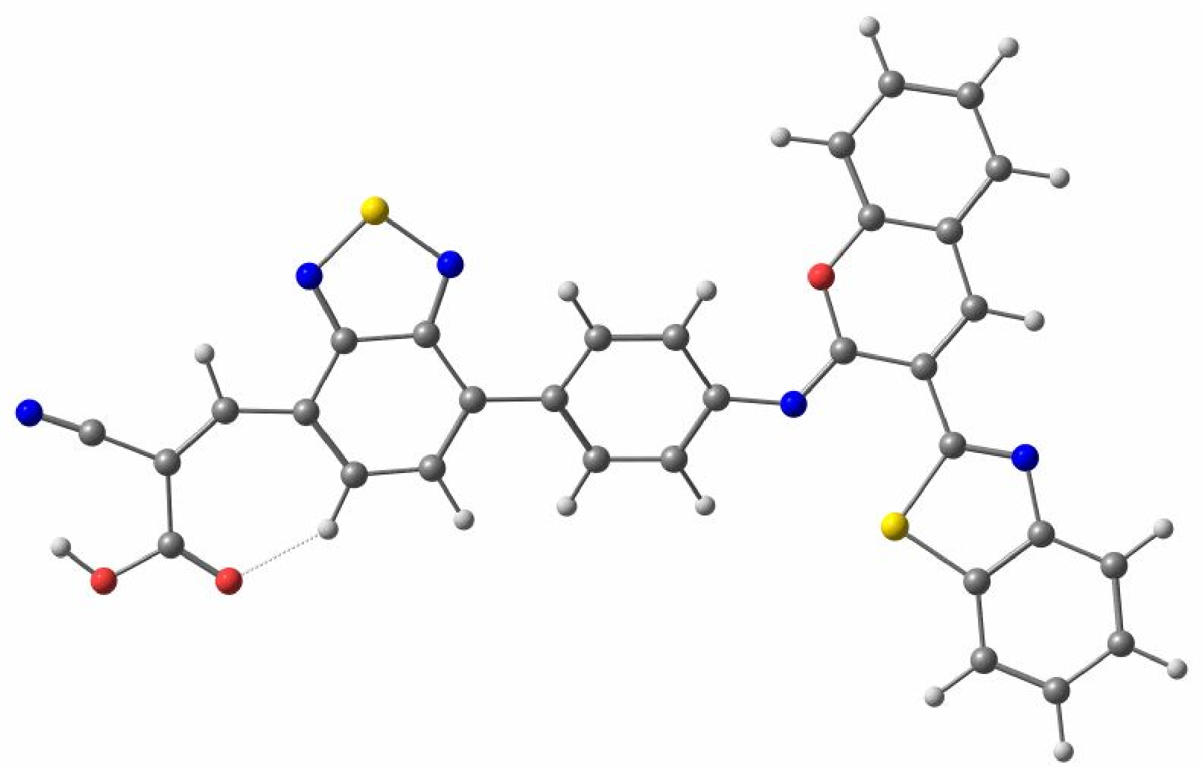

6	-5.747177000	1.580492000	0.113961000
6	-4.859170000	0.547092000	0.097891000
6	-3.443824000	0.834014000	0.182500000
8	-3.055546000	2.137344000	0.273512000
6	-3.937377000	3.177163000	0.318279000
6	-3.415268000	4.457762000	0.448589000
6	-4.295580000	5.528202000	0.485907000
6	-5.678955000	5.323167000	0.396317000
6	-6.182387000	4.042213000	0.271034000
6	-5.313257000	2.938411000	0.230432000
7	-2.565430000	-0.107089000	0.213134000
6	-1.206731000	0.008499000	0.150779000
6	-0.457218000	-1.070962000	0.683542000
6	0.911061000	-1.073106000	0.647515000
6	1.646659000	-0.004408000	0.050940000
6	0.880775000	1.060796000	-0.506364000
6	-0.490794000	1.070081000	-0.460454000
6	3.087167000	-0.025404000	0.007458000
6	-5.366523000	-0.832520000	-0.013826000

7	-6.642898000	-1.054758000	-0.077550000
16	-4.345925000	-2.264763000	-0.086073000
6	-5.786976000	-3.232676000	-0.216574000
6	-6.919076000	-2.400025000	-0.190705000
6	-8.201651000	-2.956685000	-0.278276000
6	-5.911233000	-4.619073000	-0.327676000
6	-8.323069000	-4.328874000	-0.389535000
6	-7.185554000	-5.154553000	-0.414300000
6	3.802581000	-1.227728000	0.259807000
6	5.178670000	-1.342757000	0.236205000
6	6.021041000	-0.237995000	-0.035865000
6	5.349969000	1.015467000	-0.306800000
6	3.901647000	1.124423000	-0.294756000
7	3.476860000	2.355542000	-0.554271000
7	5.960732000	2.155801000	-0.577156000
16	4.803135000	3.302855000	-0.805240000
6	7.443866000	-0.207429000	-0.074064000
6	8.466838000	-1.121377000	0.106644000
6	9.790839000	-0.597990000	-0.031946000
7	10.893393000	-0.245302000	-0.128535000
6	8.405387000	-2.571826000	0.423165000
8	7.387150000	-3.209855000	0.572490000
8	9.590116000	-3.205474000	0.548140000
1	-6.805479000	1.353518000	0.043466000
1	-2.342323000	4.594137000	0.519912000
1	-3.905066000	6.535169000	0.586762000
1	-6.353667000	6.171346000	0.427681000
1	-7.251983000	3.870394000	0.204384000
1	-1.002322000	-1.888406000	1.141956000
1	1.434485000	-1.899755000	1.109560000
1	1.402137000	1.880602000	-0.978691000
1	-1.030697000	1.890647000	-0.912949000
1	-9.069098000	-2.306302000	-0.256961000
1	-5.036768000	-5.260733000	-0.347497000
1	-9.308108000	-4.778190000	-0.458802000
1	-7.307321000	-6.229031000	-0.502969000
1	3.244862000	-2.134990000	0.450878000
1	5.637458000	-2.302122000	0.424812000
1	7.810788000	0.789968000	-0.302741000
1	10.347307000	-2.615575000	0.410770000

Eelec = -2524.522747

A2*, benzothiadiazole-containing dye with an OH substituent at the 7-coumarin positionat the first singlet excited state

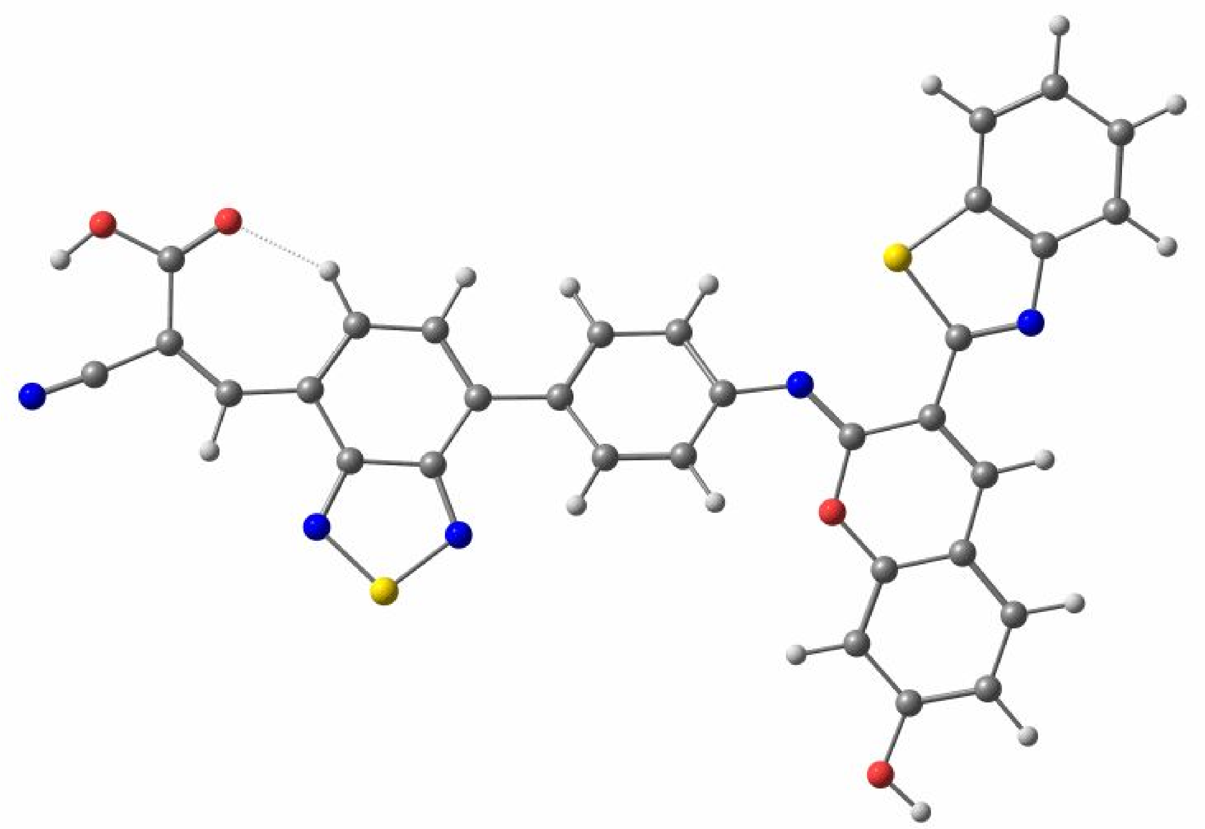

6	5.693595000	1.238067000	0.056268000
6	4.769666000	0.232236000	0.062300000
6	3.368792000	0.568036000	0.141540000
8	3.024011000	1.890009000	0.206583000
6	3.937218000	2.898310000	0.226659000
6	3.453444000	4.190991000	0.327740000
6	4.370932000	5.235141000	0.339591000
6	5.751607000	4.984305000	0.254406000
6	6.206431000	3.686344000	0.158295000
6	5.307313000	2.606124000	0.142326000
7	2.456214000	-0.339854000	0.190302000
6	1.102100000	-0.183715000	0.132477000
6	0.325781000	-1.252395000	0.649972000
6	-1.042039000	-1.219202000	0.620026000
6	-1.752307000	-0.121813000	0.045655000
6	-0.961387000	0.933725000	-0.494372000
6	0.410251000	0.907606000	-0.454814000
6	-3.193473000	-0.106307000	0.006091000
6	5.233032000	-1.163447000	-0.021149000

7	6.501881000	-1.428486000	-0.079274000
16	4.166458000	-2.563159000	-0.066524000
6	5.575574000	-3.580014000	-0.176515000
6	6.734051000	-2.784126000	-0.165847000
6	7.997651000	-3.384046000	-0.241238000
6	5.654690000	-4.971480000	-0.260165000
6	8.074506000	-4.761546000	-0.325351000
6	6.910943000	-5.550127000	-0.334973000
6	-3.937813000	-1.294286000	0.237243000
6	-5.316832000	-1.374695000	0.215411000
6	-6.132339000	-0.245136000	-0.033218000
6	-5.430762000	0.996215000	-0.282071000
6	-3.979936000	1.068881000	-0.271838000
7	-3.525344000	2.293842000	-0.509168000
7	-6.013167000	2.156564000	-0.529277000
16	-4.827805000	3.278120000	-0.738256000
6	-7.553884000	-0.178731000	-0.066235000
6	-8.599059000	-1.070453000	0.099391000
6	-9.910007000	-0.511423000	-0.022528000
7	-11.003866000	-0.129511000	-0.106723000
6	-8.572646000	-2.527935000	0.384694000
8	-7.570436000	-3.195307000	0.513599000
8	-9.772738000	-3.133961000	0.504107000
1	6.743226000	0.972267000	-0.009680000
1	2.390763000	4.387457000	0.397948000
1	6.453655000	5.812653000	0.266345000
1	7.270976000	3.485540000	0.095150000
1	0.851622000	-2.091447000	1.091542000
1	-1.584615000	-2.040502000	1.069429000
1	-1.463278000	1.775701000	-0.948445000
1	0.967745000	1.723201000	-0.894178000
1	8.885918000	-2.762065000	-0.231869000
1	4.759717000	-5.584513000	-0.268231000
1	9.044505000	-5.243827000	-0.384837000
1	6.997569000	-6.629546000	-0.402395000
1	-3.402494000	-2.218688000	0.409432000
1	-5.798524000	-2.326015000	0.386869000
1	-7.896630000	0.831912000	-0.273309000
1	-10.515024000	-2.521701000	0.384438000
8	3.871379000	6.487604000	0.436868000
1	4.578104000	7.144904000	0.439379000

Eelec = -2599.730289

A3*, benzothiadiazole-containing dye with an NH2 substituent at the 7-coumarin position at the first singlet excited state

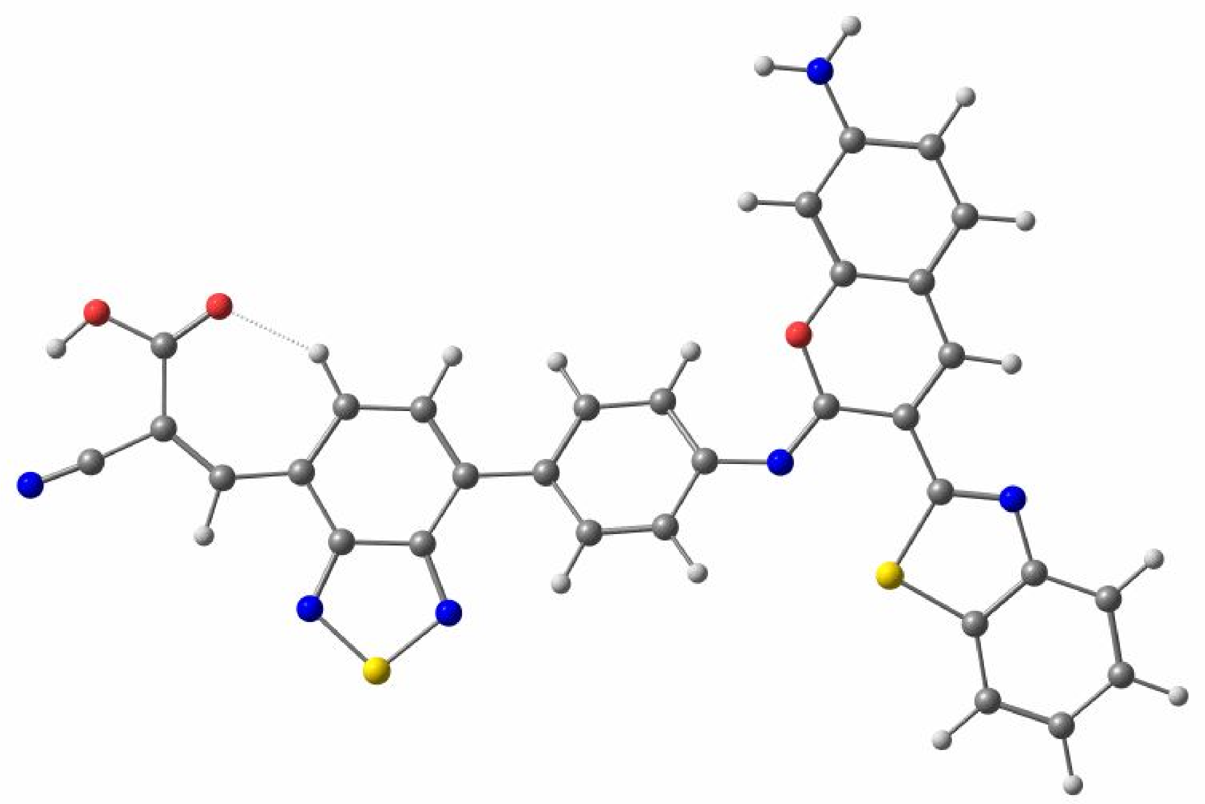

6	5.540203000	1.744184000	-0.009797000
6	4.725491000	0.640953000	-0.000399000
6	3.302125000	0.826459000	0.073423000
8	2.818536000	2.107740000	0.134996000
6	3.619171000	3.205912000	0.157028000
6	3.002374000	4.437734000	0.262464000
6	3.793157000	5.592943000	0.279003000
6	5.202998000	5.470668000	0.188344000
6	5.790433000	4.234305000	0.089351000
6	5.014035000	3.056835000	0.071938000
7	2.485610000	-0.171913000	0.120441000
6	1.124291000	-0.156049000	0.061295000
6	0.322333000	0.871293000	-0.506132000
6	-1.042710000	0.754071000	-0.534614000
6	-1.724167000	-0.378703000	0.002563000
6	-0.907367000	-1.418541000	0.531540000
6	0.459389000	-1.309833000	0.549151000
6	-3.166693000	-0.440424000	0.000237000
6	5.336888000	-0.694966000	-0.073620000
7	6.627767000	-0.823028000	-0.126759000

16	4.426837000	-2.201791000	-0.108796000
6	5.937583000	-3.062388000	-0.205269000
6	7.003924000	-2.146428000	-0.200066000
6	8.324852000	-2.608143000	-0.266766000
6	6.166209000	-4.437922000	-0.274788000
6	8.549667000	-3.970187000	-0.336808000
6	7.477616000	-4.879245000	-0.341042000
6	-3.940599000	0.730608000	-0.204047000
6	-5.323060000	0.769827000	-0.211905000
6	-6.109516000	-0.389541000	-0.024607000
6	-5.375353000	-1.618338000	0.194891000
6	-3.922696000	-1.646865000	0.216581000
7	-3.435677000	-2.865272000	0.421911000
7	-5.925924000	-2.804267000	0.386845000
16	-4.710871000	-3.894912000	0.583851000
6	-7.528319000	-0.496797000	-0.028229000
6	-8.595360000	0.372242000	-0.180937000
6	-9.892233000	-0.227508000	-0.116861000
7	-10.976993000	-0.641618000	-0.077493000
6	-8.604624000	1.840036000	-0.399138000
8	-7.620723000	2.543506000	-0.469359000
8	-9.819494000	2.416779000	-0.524425000
1	6.612037000	1.588988000	-0.072784000
1	1.921658000	4.491395000	0.331434000
1	5.814422000	6.367131000	0.198519000
1	6.870583000	4.149485000	0.025423000
1	0.791869000	1.740587000	-0.944879000
1	-1.609481000	1.541414000	-1.014848000
1	-1.381554000	-2.306803000	0.922787000
1	1.070390000	-2.106567000	0.958552000
1	9.141186000	-1.894310000	-0.261539000
1	5.342198000	-5.143515000	-0.278599000
1	9.566130000	-4.345887000	-0.389110000
1	7.679737000	-5.943750000	-0.397496000
1	-3.429956000	1.676778000	-0.327737000
1	-5.828542000	1.712876000	-0.358638000
1	-7.846726000	-1.524467000	0.127900000
1	-10.544931000	1.777300000	-0.453530000
7	3.220006000	6.828114000	0.360254000
1	3.790866000	7.646525000	0.490848000
1	2.234327000	6.924607000	0.540032000

$Eelec$ = -2579.869122

A4*, benzothiadiazole-containing dye with a CF3 substituent at the 7-coumarin position at the first singlet excited state

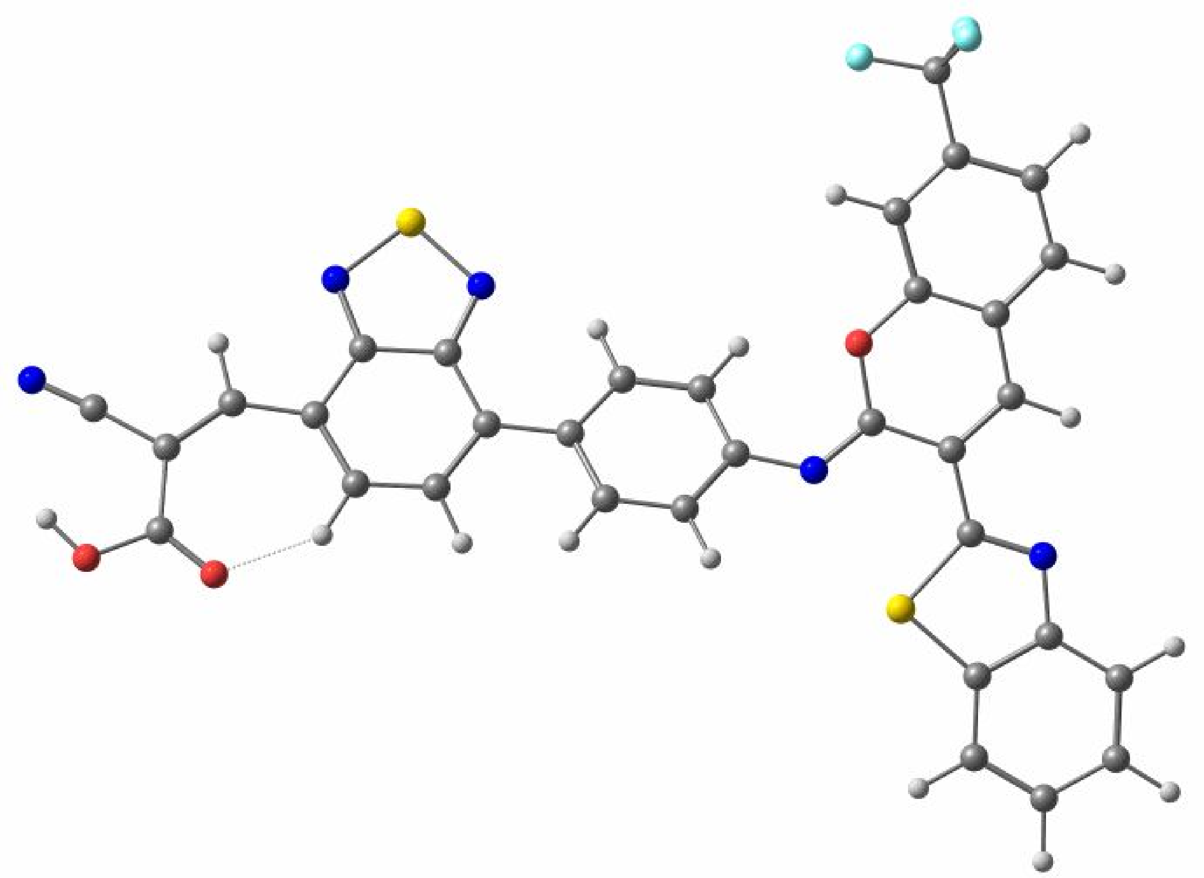

6	5.462861000	0.241717000	0.005901000
6	4.460028000	-0.679212000	0.024592000
6	3.086572000	-0.227739000	0.103505000
8	2.852326000	1.117278000	0.148976000
6	3.851070000	2.044265000	0.158586000
6	3.479287000	3.380076000	0.245050000
6	4.480319000	4.337310000	0.246575000
6	5.831793000	3.974798000	0.165339000
6	6.179001000	2.642029000	0.083748000
6	5.187918000	1.645310000	0.079362000
7	2.106441000	-1.056431000	0.171511000
6	0.768722000	-0.786906000	0.112387000
6	-0.094420000	-1.739077000	0.710106000
6	-1.453806000	-1.582878000	0.679882000
6	-2.066662000	-0.471305000	0.024399000
6	-1.187227000	0.464286000	-0.595757000
6	0.176318000	0.315075000	-0.555594000
6	-3.498484000	-0.327828000	-0.014354000

6	4.800715000	-2.112100000	-0.044428000
7	6.042058000	-2.482661000	-0.108868000
16	3.620421000	-3.417204000	-0.063991000
6	4.938024000	-4.549322000	-0.171565000
6	6.159373000	-3.853542000	-0.179850000
6	7.368119000	-4.557724000	-0.256757000
6	4.899655000	-5.943764000	-0.238083000
6	7.328451000	-5.937332000	-0.323555000
6	6.102481000	-6.625635000	-0.314480000
6	-4.347088000	-1.417863000	0.326091000
6	-5.726803000	-1.374095000	0.311578000
6	-6.439448000	-0.202456000	-0.041575000
6	-5.630643000	0.941583000	-0.403361000
6	-4.179664000	0.884224000	-0.398852000
7	-3.619740000	2.036232000	-0.746660000
7	-6.108459000	2.121929000	-0.756867000
16	-4.829823000	3.108720000	-1.066448000
6	-7.849808000	-0.011035000	-0.082143000
6	-8.968559000	-0.785622000	0.166818000
6	-10.225343000	-0.125493000	-0.009226000
7	-11.281047000	0.343390000	-0.130631000
6	-9.070438000	-2.206853000	0.590406000
8	-8.129651000	-2.944264000	0.783165000
8	-10.317722000	-2.688910000	0.765729000
1	6.487237000	-0.108214000	-0.058914000
1	2.432308000	3.648736000	0.311064000
1	6.597227000	4.742516000	0.168393000
1	7.221615000	2.348194000	0.023053000
1	0.356361000	-2.587499000	1.212531000
1	-2.063941000	-2.315440000	1.191317000
1	-1.613668000	1.311559000	-1.112691000
1	0.803966000	1.039583000	-1.056828000
1	8.305204000	-4.012104000	-0.262076000
1	3.956724000	-6.480015000	-0.231733000
1	8.253924000	-6.500135000	-0.383912000
1	6.098178000	-7.709236000	-0.368616000
1	-3.897333000	-2.367378000	0.583865000
1	-6.291326000	-2.257722000	0.570534000
1	-8.101833000	1.002258000	-0.384407000
1	-11.004784000	-2.028075000	0.588212000
6	4.134610000	5.797835000	0.345136000
9	4.628924000	6.492174000	-0.700276000
9	2.808359000	6.010621000	0.377087000
9	4.657673000	6.352128000	1.458254000

Eelec = -2861.503208

A5*, benzothiadiazole-containing dye with an NO2 substituent at the 7-coumarin position at the first singlet excited state

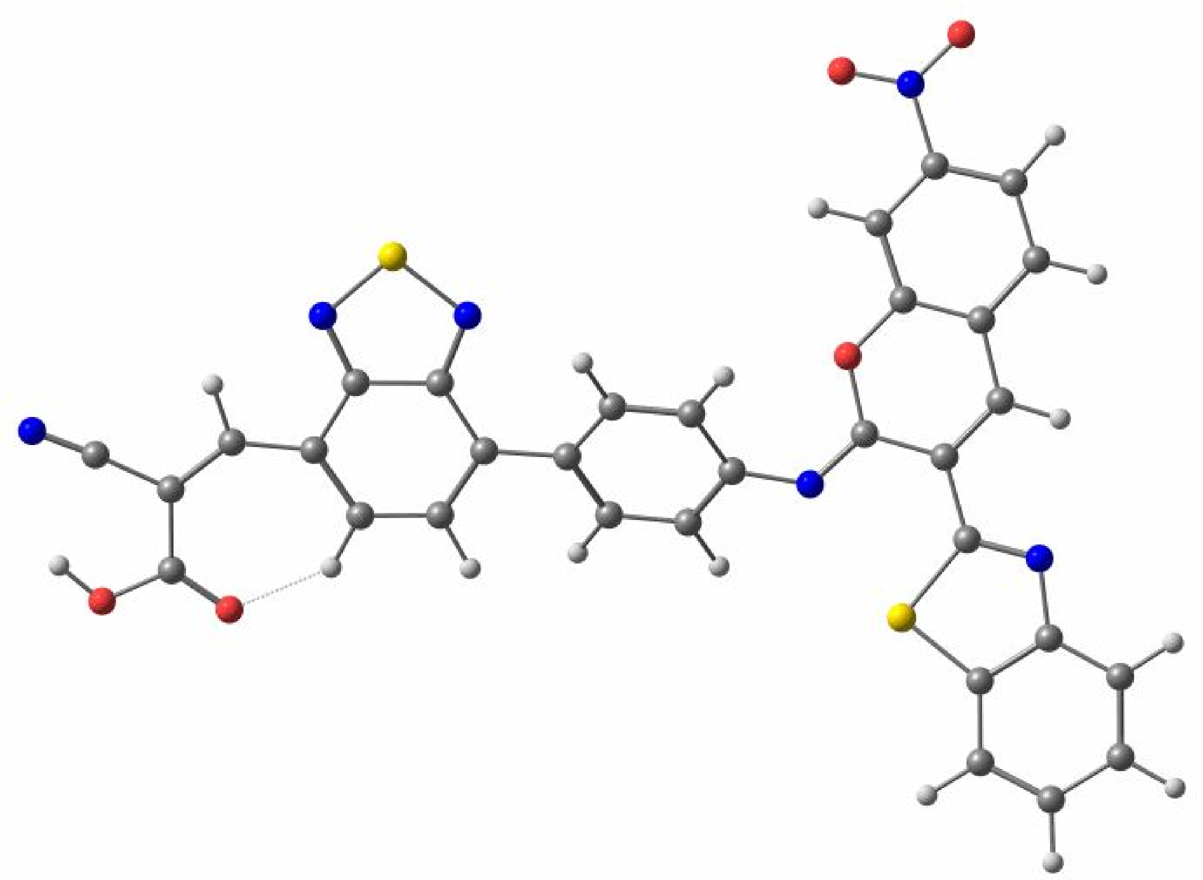

6	5.752200000	4.417924000	-0.241075000
6	4.386956000	4.694614000	-0.323878000
6	3.418241000	3.703984000	-0.311338000
6	3.854723000	2.394148000	-0.208009000
6	5.213486000	2.058498000	-0.123719000
6	6.156741000	3.102207000	-0.142164000
8	2.903986000	1.416633000	-0.186418000
6	3.207556000	0.086955000	-0.115704000
6	4.595064000	-0.295937000	-0.035798000
6	5.555111000	0.675228000	-0.033607000
7	2.263678000	-0.788005000	-0.172786000
6	0.919726000	-0.569902000	-0.118138000
6	0.089899000	-1.484278000	-0.815457000

6	-1.272248000	-1.363856000	-0.777405000
6	-1.915781000	-0.335266000	-0.020183000
6	-1.067171000	0.553076000	0.708766000
6	0.297558000	0.438200000	0.666338000
6	-3.349623000	-0.247228000	0.018328000
6	-4.147387000	-1.371288000	-0.345608000
6	-5.522541000	-1.391011000	-0.320837000
6	-6.286694000	-0.250772000	0.046124000
6	-5.531348000	0.927615000	0.413808000
6	-4.079435000	0.931060000	0.414458000
6	-7.700759000	-0.124770000	0.097470000
6	-8.781963000	-0.950877000	-0.160123000
6	4.999111000	-1.708923000	0.046650000
7	6.255688000	-2.025292000	0.114745000
6	6.431123000	-3.388847000	0.195449000
6	5.240788000	-4.137019000	0.190719000
16	3.875910000	-3.064059000	0.072762000
6	7.668739000	-4.040254000	0.279478000
6	7.687833000	-5.419647000	0.357049000
6	6.492498000	-6.159844000	0.351721000
6	5.261539000	-5.531047000	0.268025000
7	-6.065823000	2.083451000	0.764440000
16	-4.827167000	3.121858000	1.071502000
7	-3.566575000	2.105501000	0.757553000
7	3.952795000	6.090823000	-0.429002000
8	2.751278000	6.307032000	-0.507911000
8	4.819836000	6.953618000	-0.458502000
6	-8.813156000	-2.364612000	-0.618834000
8	-7.835697000	-3.043166000	-0.845588000
8	-10.033743000	-2.911990000	-0.788934000
6	-10.067315000	-0.360116000	0.043152000
7	-11.146270000	0.046721000	0.185722000
1	6.461795000	5.234606000	-0.257161000
1	2.365926000	3.946873000	-0.380518000
1	7.211704000	2.858215000	-0.079148000
1	6.594655000	0.374108000	0.028875000
1	0.568691000	-2.260606000	-1.400636000
1	-1.873936000	-2.044178000	-1.365787000
1	-1.521326000	1.324787000	1.310903000
1	0.912564000	1.107343000	1.253112000
1	-3.648324000	-2.294194000	-0.612501000
1	-6.051089000	-2.295187000	-0.583652000
1	-8.001052000	0.869244000	0.419118000
1	8.581568000	-3.454987000	0.281717000

1	8.636386000	-5.941851000	0.423082000
1	6.534471000	-7.242137000	0.414436000
1	4.342557000	-6.107299000	0.264251000
1	-10.755033000	-2.297028000	-0.584137000

Eelec = -2728.959712

C1*, benzo[*c*]thiadiazole and thiophene-containing dye with a H at the 7-coumarin position at the first singlet excited state

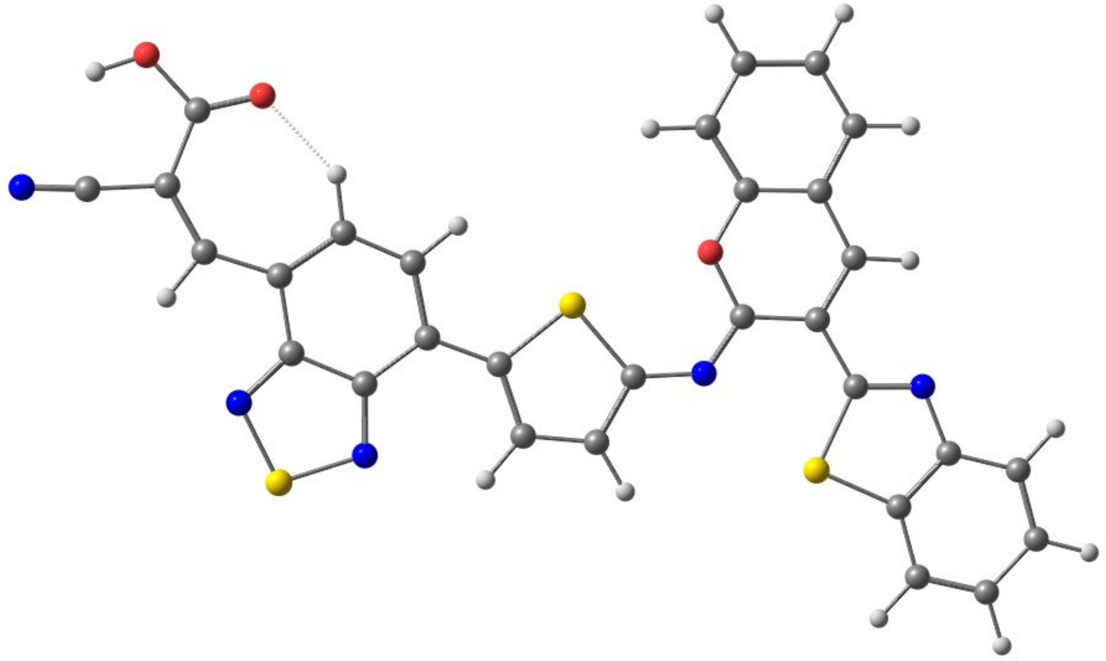

6	-4.743172000	2.393359000	-0.000058000
6	-4.334778000	1.088084000	-0.000032000
6	-2.928176000	0.802575000	-0.000036000
8	-2.049462000	1.841242000	-0.000066000
6	-2.440133000	3.148332000	-0.000092000
6	-1.448225000	4.119686000	-0.000121000
6	-1.832063000	5.452283000	-0.000147000
6	-3.187755000	5.808345000	-0.000144000
6	-4.162642000	4.829089000	-0.000115000
6	-3.804121000	3.469026000	-0.000088000
7	-2.466178000	-0.410996000	-0.000012000
6	2.725985000	-0.967144000	-0.000020000
6	-5.352668000	0.021049000	-0.000001000
7	-6.613400000	0.324691000	0.000001000

16	-4.991676000	-1.702057000	0.000033000
6	-6.703165000	-2.017810000	0.000054000
6	-7.407594000	-0.801556000	0.000032000
6	-8.808367000	-0.801895000	0.000043000
6	-7.374295000	-3.242270000	0.000087000
6	-9.471415000	-2.014527000	0.000075000
6	-8.759208000	-3.226466000	0.000097000
6	3.309251000	0.324427000	-0.000046000
6	4.672551000	0.560440000	-0.000045000
6	5.618732000	-0.487104000	-0.000018000
6	5.077150000	-1.830058000	0.000000000
6	3.648878000	-2.072896000	0.000001000
7	3.336527000	-3.361776000	0.000024000
7	5.793063000	-2.942627000	0.000022000
16	4.748300000	-4.209741000	0.000041000
6	7.040843000	-0.384503000	0.000014000
6	7.967386000	0.639629000	-0.000008000
6	9.339394000	0.231881000	0.000090000
7	10.472989000	-0.020299000	0.000174000
6	7.760149000	2.111181000	-0.000119000
8	6.681969000	2.662554000	0.000022000
8	8.874943000	2.870542000	0.000229000
1	-5.807460000	2.601166000	-0.000054000
1	-0.406026000	3.822565000	-0.000123000
1	-1.071008000	6.225199000	-0.000170000
1	-3.470181000	6.855233000	-0.000164000
1	-5.215186000	5.093717000	-0.000112000
1	-9.343041000	0.141499000	0.000025000
1	-6.829781000	-4.180498000	0.000104000
1	-10.556096000	-2.034890000	0.000084000
1	-9.302868000	-4.165413000	0.000123000
1	2.655054000	1.189853000	-0.000066000
1	5.036352000	1.577460000	-0.000073000
1	7.505732000	-1.367382000	0.000069000
1	9.688239000	2.342368000	0.000318000
6	-0.794323000	-2.137740000	0.000014000
6	-1.181692000	-0.783463000	-0.000010000
16	0.242622000	0.254115000	-0.000036000
6	1.316602000	-1.139359000	-0.000014000
6	0.571488000	-2.337759000	0.000012000
1	-1.539179000	-2.923046000	0.000032000
1	1.059210000	-3.301564000	0.000029000

$Eelec$ = -2845.4119179

C2*, benzo[*c*]thiadiazole and thiophene-containing dye at the 7-coumarin position at the first singlet excited state

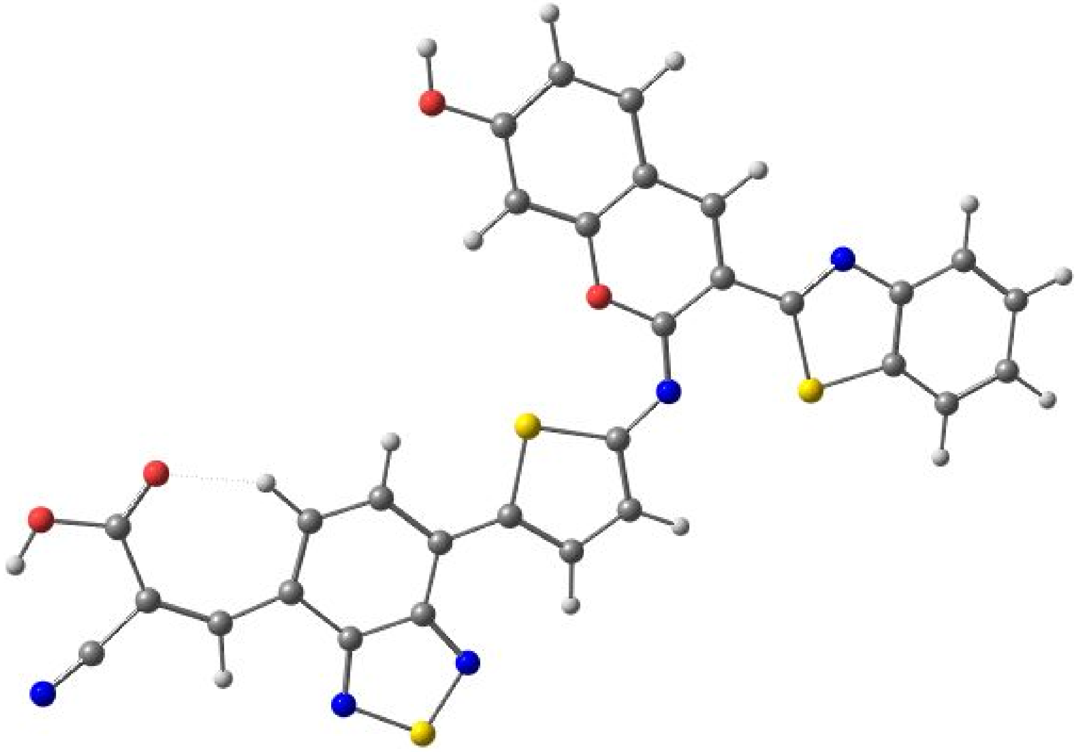

6	-4.735917000	2.196913000	-0.000062000
6	-4.319916000	0.891141000	-0.000033000
6	-2.914458000	0.615024000	-0.000027000
8	-2.039223000	1.662630000	-0.000053000
6	-2.435777000	2.964220000	-0.000083000
6	-1.448244000	3.932043000	-0.000108000
6	-1.839721000	5.266565000	-0.000137000
6	-3.200973000	5.619491000	-0.000143000
6	-4.164654000	4.633762000	-0.000118000
6	-3.806694000	3.273527000	-0.000088000
7	-2.440273000	-0.593579000	0.000003000
6	2.756308000	-1.115187000	0.000022000
6	-5.332579000	-0.179116000	-0.000009000
7	-6.595387000	0.117356000	-0.000017000
16	-4.961805000	-1.900336000	0.000032000
6	-6.671797000	-2.225952000	0.000039000
6	-7.383033000	-1.013664000	0.000009000
6	-8.783693000	-1.022442000	0.000008000
6	-7.335769000	-3.454126000	0.000067000
6	-9.439915000	-2.238980000	0.000036000

6	-8.720915000	-3.446700000	0.000066000
6	3.332018000	0.179458000	-0.000003000
6	4.694157000	0.423298000	-0.000005000
6	5.646515000	-0.618647000	0.000018000
6	5.112477000	-1.964723000	0.000044000
6	3.685454000	-2.215553000	0.000046000
7	3.380445000	-3.506381000	0.000072000
7	5.834701000	-3.073248000	0.000068000
16	4.796948000	-4.346186000	0.000091000
6	7.067746000	-0.508456000	0.000022000
6	7.989568000	0.520278000	0.000001000
6	9.363456000	0.119206000	0.000025000
7	10.498363000	-0.127339000	0.000045000
6	7.774912000	1.990384000	-0.000039000
8	6.694178000	2.536606000	0.000032000
8	8.886305000	2.755508000	0.000073000
1	-5.801827000	2.396932000	-0.000066000
1	-0.399340000	3.663240000	-0.000104000
1	-3.487596000	6.666919000	-0.000166000
1	-5.216498000	4.900400000	-0.000122000
1	-9.323994000	-0.082234000	-0.000015000
1	-6.785677000	-4.389115000	0.000090000
1	-10.524489000	-2.265444000	0.000036000
1	-9.259010000	-4.388850000	0.000087000
1	2.673071000	1.041314000	-0.000020000
1	5.051743000	1.442552000	-0.000025000
1	7.537670000	-1.488945000	0.000045000
1	9.701819000	2.230828000	0.000095000
6	-0.756968000	-2.308476000	0.000047000
6	-1.153540000	-0.957107000	0.000013000
16	0.264592000	0.089960000	-0.000013000
6	1.347540000	-1.296403000	0.000023000
6	0.610359000	-2.499362000	0.000053000
1	-1.496334000	-3.098989000	0.000067000
1	1.104150000	-3.460040000	0.000078000
8	-0.855469000	6.191456000	-0.000161000
1	-1.216390000	7.086661000	-0.000181000

$Eelec$ = -2920.6188603

C3*, benzo[*c*]thiadiazole and thiophene-containing dye at with a H at the 7-coumarin position at the first singlet excited state

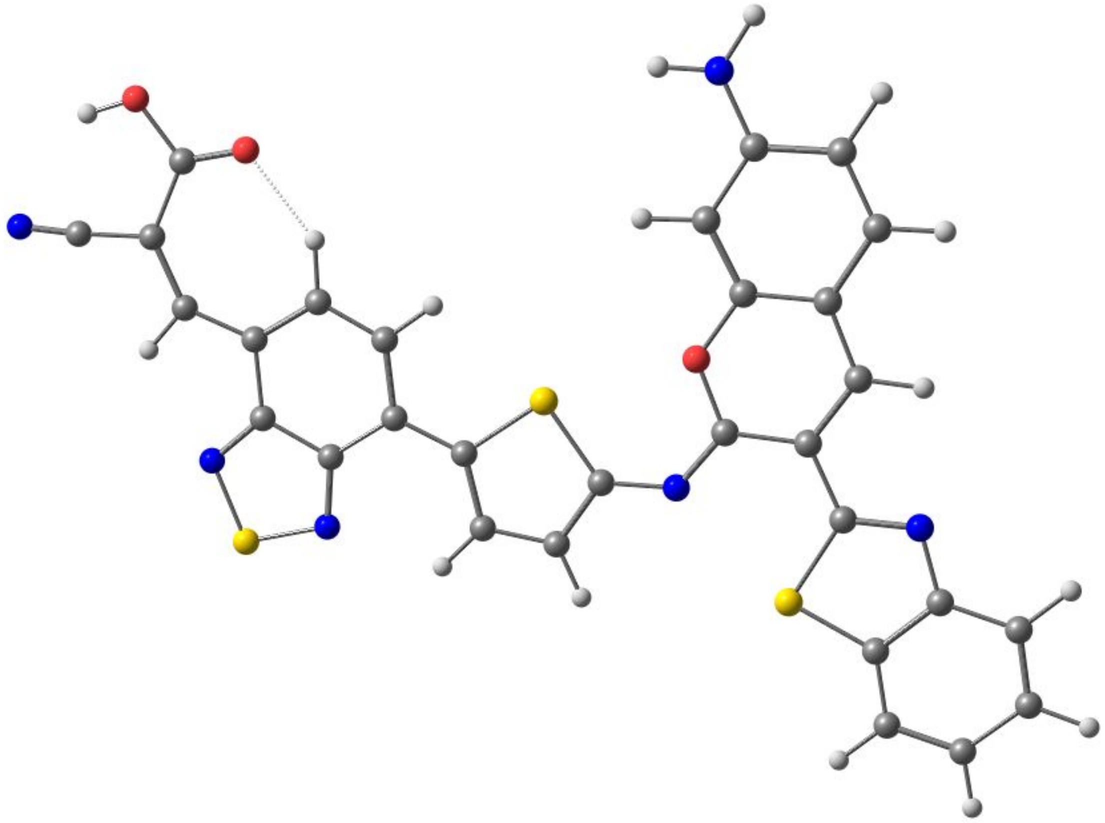

6	-4.741793000	2.206058000	-0.003823000
6	-4.348430000	0.904108000	-0.002586000
6	-2.928637000	0.600873000	-0.006983000
8	-2.047472000	1.647501000	-0.012814000
6	-2.437096000	2.956202000	-0.014566000
6	-1.439062000	3.912783000	-0.019135000
6	-1.796736000	5.265107000	-0.020057000
6	-3.165068000	5.617177000	-0.015540000
6	-4.136331000	4.642053000	-0.010344000
6	-3.798615000	3.277519000	-0.009784000
7	-2.481973000	-0.596989000	-0.005121000
6	2.759558000	-1.113721000	-0.006550000
6	-5.367563000	-0.157699000	0.003830000
7	-6.630900000	0.138269000	0.008177000
16	-4.998400000	-1.881754000	0.006157000
6	-6.709314000	-2.205725000	0.013503000
6	-7.418983000	-0.992728000	0.013681000
6	-8.819400000	-1.001341000	0.019146000
6	-7.375086000	-3.432857000	0.018725000
6	-9.477688000	-2.217438000	0.024302000
6	-8.760582000	-3.425791000	0.024096000

6	3.331206000	0.146821000	0.000544000
6	4.719605000	0.392233000	0.003435000
6	5.663010000	-0.620557000	-0.001027000
6	5.125096000	-1.960233000	-0.009554000
6	3.702430000	-2.208576000	-0.012032000
7	3.406157000	-3.505103000	-0.020086000
7	5.845918000	-3.076068000	-0.015637000
16	4.806533000	-4.327199000	-0.023973000
6	7.100687000	-0.512445000	-0.000261000
6	8.016352000	0.501988000	0.012914000
6	9.396098000	0.105474000	0.006263000
7	10.531303000	-0.131172000	0.002075000
6	7.791799000	1.976841000	0.035320000
8	6.705096000	2.505886000	0.044122000
8	8.894577000	2.746818000	0.046930000
1	-5.804966000	2.422232000	-0.000176000
1	-0.400512000	3.601760000	-0.026595000
1	-3.444494000	6.665837000	-0.021277000
1	-5.185131000	4.922302000	-0.006475000
1	-9.358884000	-0.060556000	0.019159000
1	-6.825179000	-4.368146000	0.018540000
1	-10.562452000	-2.242020000	0.028509000
1	-9.299250000	-4.367714000	0.028171000
1	2.683762000	1.016418000	0.004682000
1	5.062825000	1.416818000	0.010075000
1	7.563436000	-1.496579000	-0.012837000
1	9.715887000	2.230912000	0.038349000
6	-0.785412000	-2.308092000	-0.006656000
6	-1.170649000	-0.984394000	-0.006913000
16	0.241224000	0.058755000	-0.008689000
6	1.324963000	-1.312878000	-0.007923000
6	0.608838000	-2.493341000	-0.007416000
1	-1.514823000	-3.107393000	-0.005687000
1	1.088687000	-3.460967000	-0.007292000
7	-0.832827000	6.242150000	-0.062292000
1	-1.092255000	7.190656000	0.155200000
1	0.123096000	5.995820000	0.138365000

Eelec = -2900.7623314

C4*, benzo[*c*]thiadiazole and thiophene-containing dye at with a H at the 7-coumarin position at the first singlet excited state

6	-4.738037000	1.636770000	-0.008248000
6	-4.294195000	0.342363000	-0.006053000
6	-2.881939000	0.091834000	-0.005129000
8	-2.027539000	1.153836000	-0.007130000
6	-2.454166000	2.448611000	-0.008143000
6	-1.485150000	3.442455000	-0.009150000
6	-1.905791000	4.763099000	-0.011685000
6	-3.269054000	5.089741000	-0.012704000
6	-4.215648000	4.086356000	-0.011436000
6	-3.824618000	2.734766000	-0.008903000
7	-2.389452000	-1.109134000	-0.002296000
6	2.810093000	-1.560350000	-0.000709000
6	-5.284061000	-0.750854000	-0.004352000
7	-6.551640000	-0.477290000	-0.006013000
16	-4.882016000	-2.464469000	0.000507000
6	-6.585154000	-2.821019000	0.000348000
6	-7.318640000	-1.621983000	-0.003463000
6	-8.719101000	-1.655531000	-0.004268000
6	-7.226744000	-4.061317000	0.003420000
6	-9.352692000	-2.883593000	-0.001218000
6	-8.611499000	-4.078202000	0.002604000
6	3.371280000	-0.258845000	-0.006673000
6	4.730681000	0.000445000	-0.007110000
6	5.695267000	-1.029372000	-0.001494000
6	5.177530000	-2.381038000	0.004308000
6	3.754381000	-2.650065000	0.004644000
7	3.466497000	-3.943950000	0.010358000
7	5.913188000	-3.481065000	0.009735000
16	4.892486000	-4.765399000	0.014939000
6	7.116616000	-0.903238000	-0.000348000
6	8.025269000	0.135128000	-0.005195000
6	9.404182000	-0.249474000	-0.000343000
7	10.541550000	-0.483202000	0.003199000
6	7.791734000	1.603833000	-0.015103000
8	6.702970000	2.132825000	-0.020918000
8	8.891730000	2.382919000	-0.017562000
1	-5.806942000	1.817614000	-0.009014000
1	-0.435102000	3.178123000	-0.010580000
1	-3.573315000	6.130284000	-0.017133000
1	-5.273288000	4.327806000	-0.013558000
1	-9.276116000	-0.725187000	-0.007206000
1	-6.660142000	-4.986330000	0.006406000
1	-10.436531000	-2.930108000	-0.001745000
1	-9.132686000	-5.029781000	0.004986000

1	2.702995000	0.595740000	-0.011442000
1	5.075954000	1.024039000	-0.012108000
1	7.597303000	-1.878413000	0.006016000
1	9.715279000	1.870945000	-0.012451000
6	-0.683581000	-2.800713000	0.003358000
6	-1.097988000	-1.453153000	-0.001133000
16	0.304285000	-0.386593000	-0.004514000
6	1.406181000	-1.758419000	0.000038000
6	0.684365000	-2.972654000	0.004131000
1	-1.412995000	-3.600328000	0.005858000
1	1.191338000	-3.926433000	0.007504000
6	-0.897570000	5.878843000	0.014080000
9	-0.920078000	6.532533000	1.194736000
9	0.357378000	5.439083000	-0.174410000
9	-1.153059000	6.796687000	-0.939756000

Eelec = -3182.3925638

<u>C5*, benzo[*c*]thiadiazole and thiophene-containing dye at with a H at the 7-coumarin position at the first singlet excited state</u>

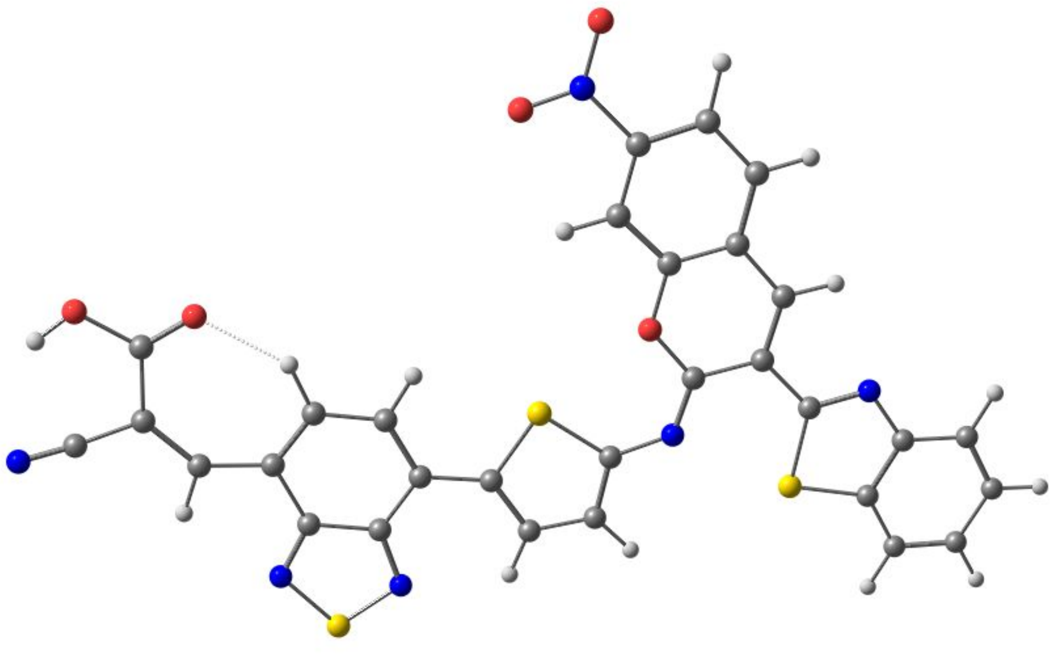

6	-4.733446000	1.876891000	-0.000047000
6	-4.304286000	0.563887000	-0.000020000
6	-2.906485000	0.300702000	-0.000016000

8	-2.035261000	1.343239000	-0.000039000
6	-2.437830000	2.649082000	-0.000067000
6	-1.455653000	3.620085000	-0.000093000
6	-1.872830000	4.944809000	-0.000122000
6	-3.224675000	5.306423000	-0.000125000
6	-4.182006000	4.316709000	-0.000100000
6	-3.812792000	2.952697000	-0.000071000
7	-2.418752000	-0.912404000	0.000008000
6	2.774515000	-1.376724000	0.000040000
6	-5.308463000	-0.510897000	0.000002000
7	-6.572536000	-0.217975000	-0.000004000
16	-4.934914000	-2.233010000	0.000038000
6	-6.644116000	-2.561659000	0.000047000
6	-7.357452000	-1.350394000	0.000021000
6	-8.758137000	-1.361544000	0.000021000
6	-7.305747000	-3.790976000	0.000074000
6	-9.412085000	-2.579203000	0.000048000
6	-8.690942000	-3.785632000	0.000074000
6	3.346244000	-0.086885000	0.000032000
6	4.713858000	0.161865000	0.000037000
6	5.668278000	-0.867083000	0.000048000
6	5.139316000	-2.212988000	0.000061000
6	3.716286000	-2.473217000	0.000057000
7	3.423860000	-3.766038000	0.000071000
7	5.866981000	-3.320478000	0.000079000
16	4.839670000	-4.591023000	0.000085000
6	7.096262000	-0.754769000	0.000042000
6	8.011625000	0.270991000	0.000021000
6	9.388825000	-0.124445000	0.000006000
7	10.523813000	-0.366580000	-0.000009000
6	7.786805000	1.744193000	0.000011000
8	6.699457000	2.273376000	0.000073000
8	8.889303000	2.515939000	-0.000037000
1	-5.800004000	2.069321000	-0.000050000
1	-0.404139000	3.365698000	-0.000090000
1	-3.489670000	6.355680000	-0.000148000
1	-5.235570000	4.574244000	-0.000103000
1	-9.299835000	-0.422196000	0.000001000
1	-6.754329000	-4.725157000	0.000094000
1	-10.496563000	-2.607612000	0.000049000
1	-9.227481000	-4.728644000	0.000095000
1	2.688830000	0.775999000	0.000021000
1	5.062166000	1.184681000	0.000035000
1	7.565969000	-1.735208000	0.000050000

1	9.711582000	2.001861000	-0.000062000
6	-0.720664000	-2.608072000	0.000037000
6	-1.134610000	-1.254744000	0.000017000
16	0.274761000	-0.192503000	0.000009000
6	1.368926000	-1.566651000	0.000034000
6	0.643691000	-2.780121000	0.000046000
1	-1.452267000	-3.405446000	0.000045000
1	1.146581000	-3.735968000	0.000062000
7	-0.860010000	5.991213000	-0.000150000
8	0.316514000	5.644694000	-0.000160000
8	-1.243203000	7.156401000	-0.000185000

Eelec = -3049.8497877

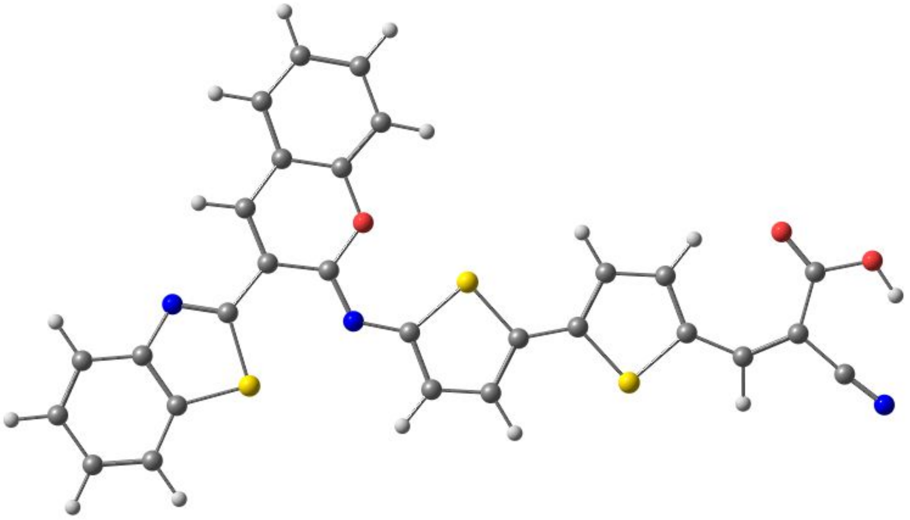

D1*, oligothyophene-containing dye with a H at the 7-coumarin position at the first singlet excited state

6	2.228338000	3.092227000	0.000046000
6	1.317684000	4.138560000	0.000044000
6	1.803835000	5.439070000	0.000118000
6	3.183368000	5.685400000	0.000193000
6	4.078783000	4.632682000	0.000193000
6	3.617026000	3.302105000	0.000120000
6	4.465613000	2.159031000	0.000109000
6	3.946554000	0.884242000	0.000010000
7	1.962883000	-0.461255000	-0.000177000
6	2.532577000	0.714712000	-0.000066000
8	1.733923000	1.819916000	-0.000019000
6	0.661255000	-0.722810000	-0.000199000

6	-1.858571000	-0.864900000	-0.000104000
6	-1.215467000	-2.125323000	-0.000339000
6	0.157073000	-2.045223000	-0.000379000
6	-5.758096000	-0.745151000	0.000045000
6	-5.262540000	0.574223000	0.000247000
6	-3.883201000	0.647083000	0.000228000
6	-3.235193000	-0.605955000	-0.000011000
16	-4.419234000	-1.892234000	-0.000185000
6	-7.058074000	-1.297685000	0.000300000
6	-8.339237000	-0.766183000	0.000222000
6	4.874734000	-0.259932000	-0.000024000
16	4.376057000	-1.950347000	-0.000241000
6	6.056747000	-2.403504000	-0.000048000
6	6.856562000	-1.247808000	0.000069000
7	6.156264000	-0.060636000	0.000071000
6	6.627298000	-3.677754000	-0.000041000
6	8.009256000	-3.774025000	0.000086000
6	8.816283000	-2.623446000	0.000203000
6	8.252449000	-1.361223000	0.000196000
6	-8.734929000	0.657941000	-0.000435000
8	-7.957375000	1.587829000	-0.000914000
8	-10.060242000	0.916735000	-0.000507000
6	-9.412476000	-1.706401000	0.000733000
7	-10.331031000	-2.418304000	0.001133000
1	0.255408000	3.923576000	-0.000015000
1	3.548222000	6.706688000	0.000248000
1	5.148716000	4.814503000	0.000249000
1	5.542830000	2.278594000	0.000165000
1	-1.769237000	-3.056319000	-0.000470000
1	0.834769000	-2.888900000	-0.000535000
1	-5.932390000	1.420958000	0.000319000
1	-3.334805000	1.581742000	0.000382000
1	-7.060967000	-2.385864000	0.000609000
1	6.008927000	-4.569111000	-0.000145000
1	8.475431000	-4.753787000	0.000088000
1	9.895890000	-2.730638000	0.000300000
1	8.861113000	-0.463753000	0.000290000
1	-10.597364000	0.109734000	-0.000083000
16	-0.681557000	0.438137000	0.000033000
1	1.106010000	6.269500000	0.000119000

$Eelec$ = -2659.695271

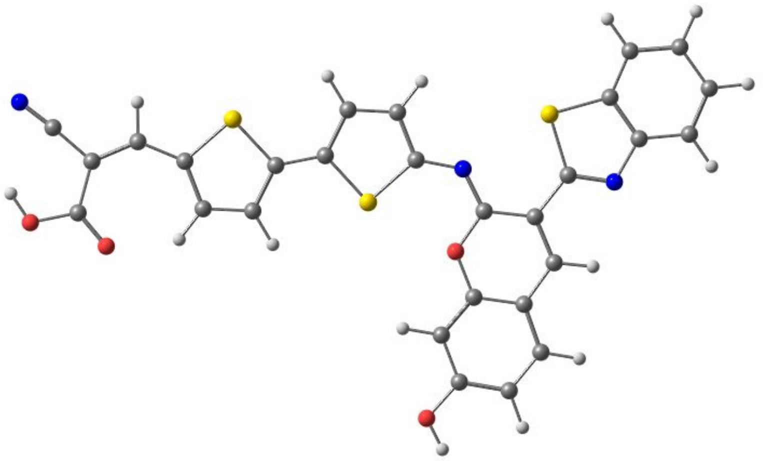

<u>D2*, oligothiophene-containing dye with an OH at the 7-coumarin position at the first singlet excited state</u>

6	2.223201000	2.891971000	0.000109000
6	1.318420000	3.936812000	0.000073000
6	1.815491000	5.236543000	0.000087000
6	3.199518000	5.478102000	0.000132000
6	4.081591000	4.417377000	0.000155000
6	3.617290000	3.088611000	0.000147000
6	4.455553000	1.942390000	0.000142000
6	3.927579000	0.670546000	0.000066000
7	1.932102000	-0.657254000	-0.000120000
6	2.513148000	0.511158000	0.000024000
8	1.720776000	1.627386000	0.000123000
6	0.628001000	-0.910458000	-0.000096000
6	-1.893182000	-1.034559000	0.000037000
6	-1.258830000	-2.299258000	-0.000262000
6	0.114812000	-2.228404000	-0.000319000
6	-5.792598000	-0.889448000	0.000164000
6	-5.287890000	0.427210000	0.000421000
6	-3.908374000	0.491183000	0.000439000
6	-3.267944000	-0.765937000	0.000162000
16	-4.460238000	-2.045308000	-0.000084000
6	-7.095313000	-1.432721000	0.000489000
6	-8.374099000	-0.892254000	0.000209000
6	4.848877000	-0.478392000	0.000025000
16	4.337754000	-2.164812000	-0.000015000
6	6.015295000	-2.630361000	-0.000131000
6	6.823578000	-1.480572000	-0.000021000
7	6.132023000	-0.288175000	0.000074000

6	6.576368000	-3.908725000	-0.000263000
6	7.957626000	-4.015479000	-0.000284000
6	8.773083000	-2.870954000	-0.000175000
6	8.218539000	-1.604536000	-0.000044000
6	-8.760366000	0.533432000	-0.000834000
8	-7.977829000	1.459460000	-0.001276000
8	-10.084804000	0.800705000	-0.001339000
6	-9.452934000	-1.825496000	0.000866000
7	-10.375926000	-2.531952000	0.001353000
1	0.251347000	3.752845000	0.000034000
1	3.570132000	6.499011000	0.000152000
1	5.151359000	4.599016000	0.000185000
1	5.533901000	2.052983000	0.000184000
1	-1.819115000	-3.226321000	-0.000419000
1	0.786342000	-3.077059000	-0.000525000
1	-5.952370000	1.278187000	0.000512000
1	-3.354153000	1.422468000	0.000672000
1	-7.105900000	-2.520855000	0.001011000
1	5.951278000	-4.795393000	-0.000332000
1	8.416423000	-4.998717000	-0.000378000
1	9.851887000	-2.986079000	-0.000194000
1	8.834011000	-0.711697000	0.000035000
1	-10.626072000	-0.003475000	-0.000869000
16	-0.706664000	0.260058000	0.000242000
8	0.906908000	6.238730000	0.000073000
1	1.340869000	7.100807000	-0.000067000

Eelec = -2734.902631

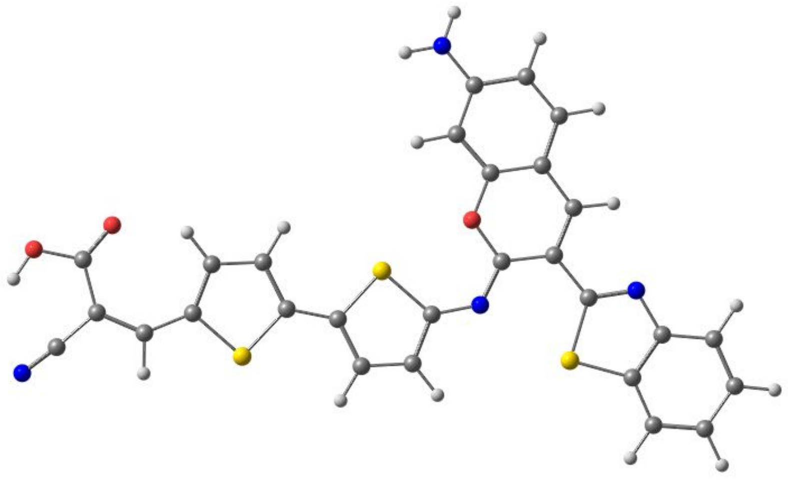

D3*, oligothiophene-containing dye with a NH2 at the 7-coumarin position at the first singlet excited state

6	2.209508000	2.883801000	0.003823000
6	1.299148000	3.920505000	0.004502000
6	1.772097000	5.239722000	0.001166000
6	3.169451000	5.474874000	-0.003365000
6	4.054756000	4.424886000	-0.004271000
6	3.604036000	3.087018000	-0.000660000
6	4.446514000	1.951315000	-0.001823000
6	3.927043000	0.672136000	0.000813000
7	1.939221000	-0.667276000	0.005641000
6	2.516101000	0.503829000	0.004474000
8	1.715613000	1.617139000	0.006503000
6	0.636387000	-0.925516000	0.006916000
6	-1.886944000	-1.055383000	0.006905000
6	-1.249878000	-2.317073000	0.007446000
6	0.125024000	-2.242998000	0.007481000
6	-5.789508000	-0.904756000	0.001404000
6	-5.281350000	0.410398000	0.003014000
6	-3.901403000	0.470836000	0.005484000
6	-3.263101000	-0.786304000	0.005749000
16	-4.457599000	-2.063309000	0.003267000
6	-7.093042000	-1.442457000	-0.001086000
6	-8.371030000	-0.896318000	-0.004239000
6	4.857477000	-0.467527000	-0.000951000
16	4.358538000	-2.157866000	0.001588000
6	6.039579000	-2.611287000	-0.002321000
6	6.839541000	-1.455567000	-0.005281000
7	6.139726000	-0.268195000	-0.004414000
6	6.609908000	-3.885431000	-0.002793000

6	7.991994000	-3.982461000	-0.006258000
6	8.799124000	-2.832123000	-0.009223000
6	8.235374000	-1.569707000	-0.008764000
6	-8.751974000	0.529235000	-0.006898000
8	-7.967996000	1.454904000	-0.006671000
8	-10.076419000	0.801110000	-0.009993000
6	-9.453218000	-1.825208000	-0.005596000
7	-10.379118000	-2.528157000	-0.006909000
1	0.237644000	3.700129000	0.010210000
1	3.534967000	6.496823000	-0.003122000
1	5.123224000	4.615391000	-0.007984000
1	5.524319000	2.068116000	-0.004951000
1	-1.807626000	-3.245679000	0.007722000
1	0.797656000	-3.090857000	0.007695000
1	-5.944410000	1.262468000	0.002289000
1	-3.344876000	1.400716000	0.007335000
1	-7.108464000	-2.530554000	-0.000479000
1	5.991145000	-4.776540000	-0.000487000
1	8.457783000	-4.962417000	-0.006663000
1	9.878753000	-2.939405000	-0.011866000
1	8.844414000	-0.672456000	-0.010981000
1	-10.619098000	-0.002123000	-0.009847000
16	-0.703329000	0.241649000	0.006903000
7	0.901104000	6.289967000	0.023445000
1	-0.087937000	6.137149000	-0.085130000
1	1.234944000	7.231419000	-0.098820000

Eelec = -2715.041755

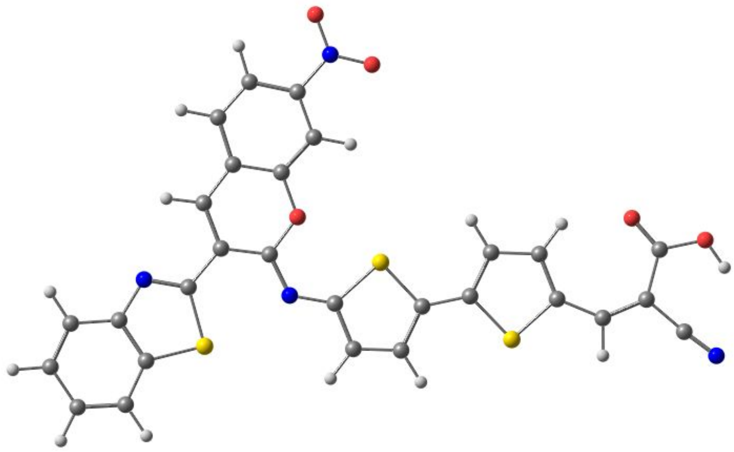

D5*, oligothiophene-containing dye with a NO2 at the 7-coumarin position at the first singlet excited state

6	2.149529000	2.517915000	-0.000340000
6	1.211755000	3.524869000	-0.000481000
6	1.680448000	4.836875000	-0.000102000
6	3.059982000	5.130916000	0.000361000
6	3.971582000	4.108792000	0.000475000
6	3.547277000	2.748062000	0.000137000
6	4.399327000	1.640773000	0.000242000
6	3.903317000	0.329203000	-0.000095000
7	1.924995000	-1.036129000	-0.000966000
6	2.501259000	0.151354000	-0.000575000
8	1.690097000	1.226882000	-0.000699000
6	0.622956000	-1.270051000	-0.000964000
6	-1.892556000	-1.365218000	-0.000514000
6	-1.281345000	-2.632200000	-0.001180000
6	0.093839000	-2.585013000	-0.001417000
6	-5.789048000	-1.167180000	0.000173000
6	-5.282163000	0.132490000	0.000636000
6	-3.891021000	0.181878000	0.000458000
6	-3.277855000	-1.076071000	-0.000168000
16	-4.478412000	-2.329823000	-0.000530000
6	-7.106114000	-1.719956000	0.000276000
6	-8.369253000	-1.190385000	0.000475000
6	4.847388000	-0.784317000	0.000066000
16	4.386053000	-2.490748000	-0.000388000
6	6.077764000	-2.907786000	0.000180000
6	6.851288000	-1.733595000	0.000615000
7	6.128409000	-0.560872000	0.000531000
6	6.674213000	-4.168740000	0.000219000

6	8.058919000	-4.237470000	0.000699000
6	8.839973000	-3.070330000	0.001134000
6	8.248967000	-1.819715000	0.001095000
6	-8.754970000	0.249229000	0.000599000
8	-7.958362000	1.156840000	0.000813000
8	-10.070828000	0.518706000	0.000442000
6	-9.454774000	-2.124070000	0.000496000
7	-10.381307000	-2.821782000	0.000535000
1	0.150430000	3.317082000	-0.000867000
1	3.369312000	6.168023000	0.000619000
1	5.035053000	4.321894000	0.000830000
1	5.475065000	1.773370000	0.000593000
1	-1.851704000	-3.553388000	-0.001436000
1	0.754824000	-3.441331000	-0.001873000
1	-5.930730000	0.995289000	0.001081000
1	-3.329304000	1.108268000	0.000776000
1	-7.107745000	-2.808215000	0.000126000
1	6.074445000	-5.072806000	-0.000123000
1	8.545190000	-5.207379000	0.000737000
1	9.921755000	-3.153579000	0.001502000
1	8.839022000	-0.909897000	0.001427000
1	-10.623786000	-0.277818000	0.000335000
16	-0.700779000	-0.090913000	-0.000279000
7	0.726737000	5.909461000	-0.000184000
8	-0.473381000	5.616830000	-0.000528000
8	1.154027000	7.066734000	0.000099000

Eelec = -2864.142174

Gas-phase conformational analysis geometries for dye series A-D

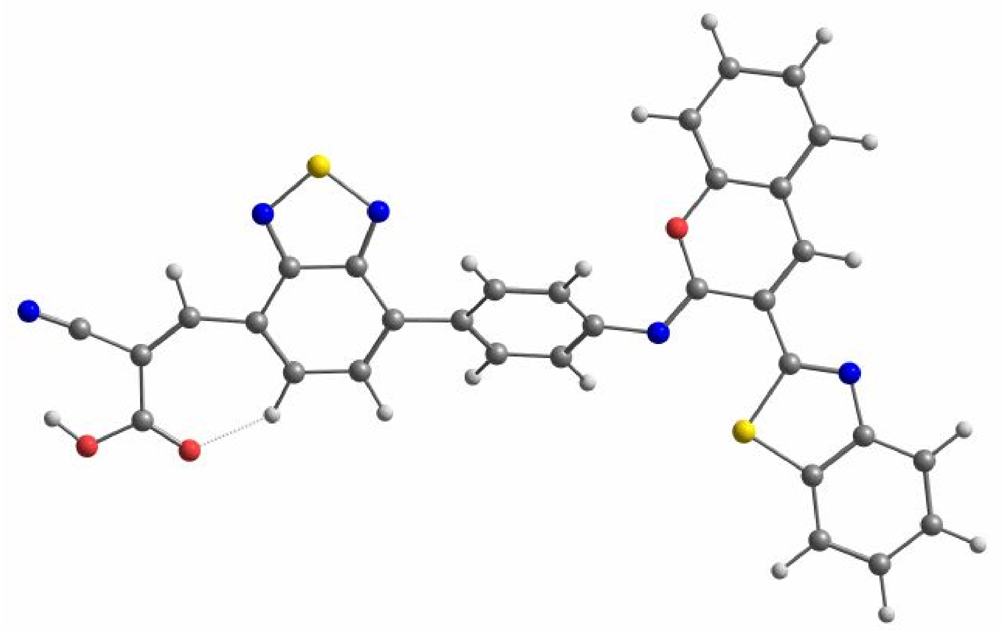

A1-*a*, MPP = 0.55

6	-5.654821000	1.670753000	0.150562000
6	-4.847732000	0.586501000	0.089177000
6	-3.395661000	0.771020000	0.124058000
8	-2.920136000	2.051828000	0.212261000
6	-3.733311000	3.146422000	0.285687000
6	-3.124821000	4.390306000	0.389588000
6	-3.928796000	5.518973000	0.462403000
6	-5.322801000	5.406918000	0.432036000
6	-5.912359000	4.158692000	0.329557000
6	-5.121824000	3.002713000	0.254935000
7	-2.594215000	-0.213339000	0.093917000
6	-1.198647000	-0.106469000	0.073526000
6	-0.467151000	-0.933547000	0.931682000
6	0.917862000	-0.918216000	0.913341000
6	1.622463000	-0.094864000	0.024638000
6	0.882758000	0.723410000	-0.839544000
6	-0.502902000	0.716621000	-0.819863000
6	3.095681000	-0.134391000	-0.009465000
6	-5.442919000	-0.757462000	-0.014856000
7	-6.731544000	-0.907495000	-0.039116000
16	-4.506774000	-2.246340000	-0.121763000
6	-6.004575000	-3.129976000	-0.207245000
6	-7.086293000	-2.234747000	-0.146436000

6	-8.400138000	-2.718202000	-0.195838000
6	-6.211739000	-4.506772000	-0.316947000
6	-8.603304000	-4.081236000	-0.304846000
6	-7.516126000	-4.970184000	-0.365172000
6	3.795664000	-1.299121000	0.208254000
6	5.210884000	-1.388236000	0.202198000
6	6.025805000	-0.299123000	-0.034287000
6	5.340587000	0.949680000	-0.270619000
6	3.900400000	1.032115000	-0.260917000
7	3.450871000	2.266652000	-0.480017000
7	5.927309000	2.119337000	-0.502210000
16	4.745527000	3.222884000	-0.686798000
6	7.471391000	-0.243119000	-0.080234000
6	8.498660000	-1.124202000	0.085819000
6	9.822073000	-0.584858000	-0.060170000
7	10.920358000	-0.227841000	-0.161248000
6	8.450564000	-2.583709000	0.404878000
8	7.433135000	-3.216233000	0.556740000
8	9.635891000	-3.205396000	0.525173000
1	-6.728917000	1.521379000	0.121566000
1	-2.042918000	4.453113000	0.414441000
1	-3.467010000	6.497192000	0.544994000
1	-5.939916000	6.296622000	0.490112000
1	-6.993017000	4.057508000	0.306817000
1	-1.004275000	-1.578853000	1.618105000
1	1.460794000	-1.543931000	1.614086000
1	1.397910000	1.369040000	-1.539286000
1	-1.053313000	1.347564000	-1.508009000
1	-9.228232000	-2.019589000	-0.148034000
1	-5.375573000	-5.196505000	-0.363949000
1	-9.614265000	-4.473218000	-0.344516000
1	-7.701047000	-6.035954000	-0.450822000
1	3.243062000	-2.217471000	0.371437000
1	5.669710000	-2.350291000	0.380095000
1	7.811683000	0.765462000	-0.303180000
1	10.391483000	-2.613678000	0.385046000

$Eelec$ = -2524.620617
$H_{(298.15\ K)}$ = -2524.160494
$G_{(298.15\ K)}$ = -2524.264441
ZPE (Zero-point energy correction) = 0.426521

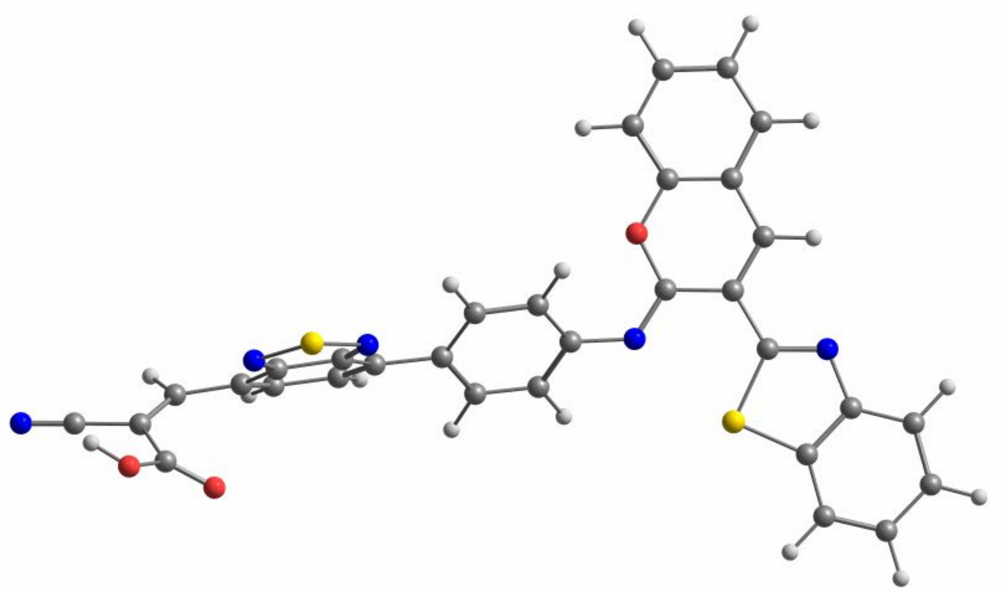

A1-*b*, MPP = 1.05

6	5.663256000	1.745094000	0.074748000
6	4.837778000	0.678187000	-0.027466000
6	3.395452000	0.896064000	-0.158048000
8	2.947350000	2.191037000	-0.165305000
6	3.780285000	3.269004000	-0.072895000
6	3.201208000	4.530752000	-0.107483000
6	4.024926000	5.643441000	-0.011576000
6	5.410086000	5.498064000	0.116973000
6	5.970641000	4.232636000	0.148404000
6	5.159708000	3.092584000	0.052962000
7	2.581957000	-0.070293000	-0.280555000
6	1.189580000	0.054591000	-0.364331000
6	0.524930000	-0.725114000	-1.315838000
6	-0.856776000	-0.690618000	-1.414364000
6	-1.625844000	0.096702000	-0.547692000
6	-0.956104000	0.843251000	0.430072000
6	0.427721000	0.833529000	0.514570000
6	-3.094389000	0.135729000	-0.695524000
6	5.403067000	-0.682410000	-0.001476000
7	6.684011000	-0.862018000	0.102801000
16	4.438724000	-2.153975000	-0.094146000
6	5.912101000	-3.074176000	0.023100000
6	7.009195000	-2.200975000	0.119123000
6	8.308080000	-2.715620000	0.221464000
6	6.089141000	-4.459516000	0.027379000
6	8.481355000	-4.087111000	0.226053000
6	7.378944000	-4.953896000	0.129807000

6	-3.692745000	0.106495000	-1.933135000
6	-5.100632000	0.116048000	-2.115257000
6	-5.989796000	0.114929000	-1.068411000
6	-5.421391000	0.198969000	0.244256000
6	-3.990291000	0.209527000	0.428193000
7	-3.638498000	0.300569000	1.710620000
7	-6.098330000	0.310838000	1.382354000
16	-5.004919000	0.384340000	2.580386000
6	-7.423508000	0.098726000	-1.331669000
6	-8.404702000	-0.517174000	-0.638830000
6	-9.768935000	-0.281208000	-1.012881000
7	-10.888476000	-0.103068000	-1.252307000
6	-8.178187000	-1.518341000	0.461934000
8	-7.207405000	-2.224093000	0.508880000
8	-9.157508000	-1.630246000	1.376254000
1	6.729291000	1.570142000	0.173528000
1	2.125777000	4.619959000	-0.210150000
1	3.585676000	6.635096000	-0.037847000
1	6.043197000	6.375281000	0.190852000
1	7.044390000	4.105656000	0.246112000
1	1.110925000	-1.362591000	-1.968780000
1	-1.349197000	-1.316759000	-2.151367000
1	-1.524837000	1.441843000	1.130161000
1	0.922954000	1.425181000	1.275445000
1	9.148207000	-2.033788000	0.295017000
1	5.241072000	-5.132185000	-0.046272000
1	9.480342000	-4.503135000	0.305079000
1	7.540289000	-6.026908000	0.136063000
1	-3.066997000	0.097042000	-2.818521000
1	-5.483612000	0.098878000	-3.131588000
1	-7.737312000	0.669517000	-2.203129000
1	-9.880120000	-1.003841000	1.223558000

$Eelec$ = -2524.615286
$H_{(298.15\ K)}$ = -2524.155738
$G_{(298.15\ K)}$ = -2524.259643
ZPE (Zero-point energy correction) = 0.425803

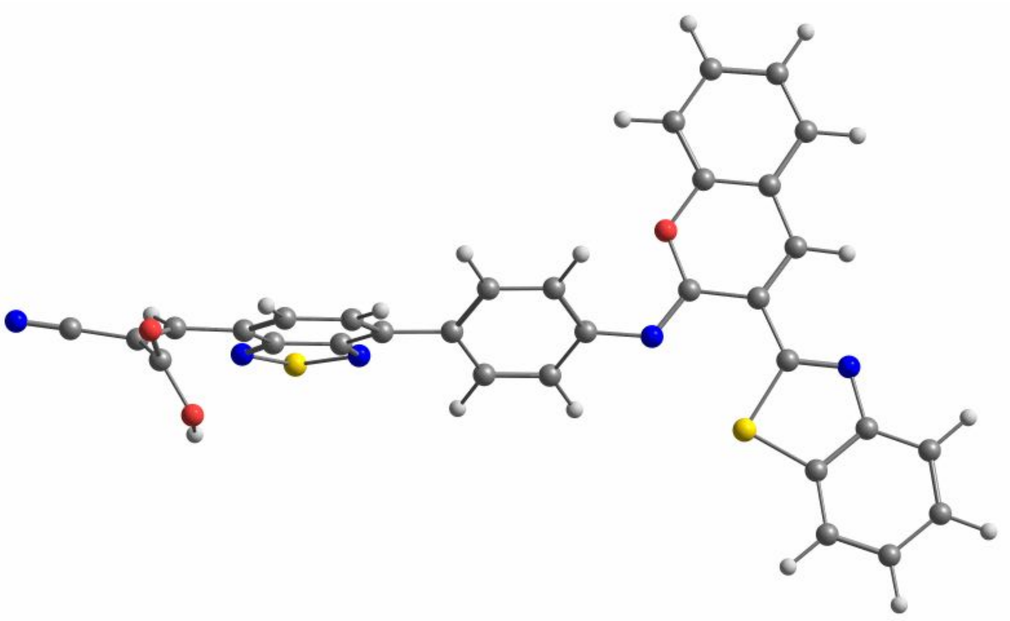

A1-*c*, MPP = 1.14

6	-5.567194000	1.804236000	-0.299656000
6	-4.769068000	0.717860000	-0.184308000
6	-3.317327000	0.898423000	-0.121823000
8	-2.832418000	2.177201000	-0.192142000
6	-3.637331000	3.275406000	-0.298498000
6	-3.021269000	4.519021000	-0.344544000
6	-3.816435000	5.650884000	-0.455403000
6	-5.209500000	5.542241000	-0.519087000
6	-5.806877000	4.294429000	-0.470517000
6	-5.025245000	3.135458000	-0.358456000
7	-2.524556000	-0.083920000	0.012183000
6	-1.128172000	0.016495000	0.033786000
6	-0.392517000	0.682104000	-0.953771000
6	0.992676000	0.673291000	-0.917388000
6	1.691669000	0.016617000	0.104258000
6	0.947792000	-0.640444000	1.092201000
6	-0.437304000	-0.658149000	1.044566000
6	3.167293000	-0.001531000	0.099678000
6	-5.373190000	-0.624882000	-0.126998000
7	-6.661712000	-0.770296000	-0.173403000
16	-4.448121000	-2.119567000	-0.004803000
6	-5.950632000	-2.999198000	-0.023088000
6	-7.025269000	-2.098227000	-0.116226000
6	-8.341395000	-2.577095000	-0.144279000
6	-6.167282000	-4.377358000	0.042619000
6	-8.553915000	-3.941509000	-0.079480000
6	-7.473810000	-4.836267000	0.013205000

6	3.889424000	-0.067411000	-1.069002000
6	5.309592000	-0.064109000	-1.117241000
6	6.090519000	-0.018546000	0.012770000
6	5.387763000	0.065269000	1.260125000
6	3.946516000	0.069134000	1.307812000
7	3.475387000	0.168601000	2.550443000
7	5.957667000	0.152276000	2.459010000
16	4.752403000	0.243532000	3.548672000
6	7.548922000	0.031418000	0.028227000
6	8.407974000	-0.473853000	-0.880851000
6	9.811507000	-0.218700000	-0.745261000
7	10.946261000	-0.028920000	-0.620791000
6	8.022725000	-1.271595000	-2.103598000
8	8.306901000	-0.936230000	-3.220099000
8	7.340965000	-2.409331000	-1.871569000
1	-6.641021000	1.657549000	-0.346564000
1	-1.940365000	4.579978000	-0.290170000
1	-3.348456000	6.628970000	-0.491514000
1	-5.819821000	6.434338000	-0.605212000
1	-6.887023000	4.196108000	-0.517260000
1	-0.909730000	1.205786000	-1.749378000
1	1.540928000	1.212714000	-1.683091000
1	1.457508000	-1.155218000	1.896758000
1	-1.007210000	-1.194199000	1.795564000
1	-9.163922000	-1.873970000	-0.215957000
1	-5.336624000	-5.071625000	0.114136000
1	-9.566759000	-4.330043000	-0.100366000
1	-7.665982000	-5.903064000	0.062303000
1	3.357393000	-0.139214000	-2.010886000
1	5.774621000	-0.077995000	-2.097081000
1	7.980360000	0.551343000	0.879267000
1	7.228843000	-2.562678000	-0.922717000

$Eelec$ = -2524.614037
$H_{(298.15\ K)}$ = -2524.154256
$G_{(298.15\ K)}$ = -2524.259673
ZPE (Zero-point energy correction) = 0.425865

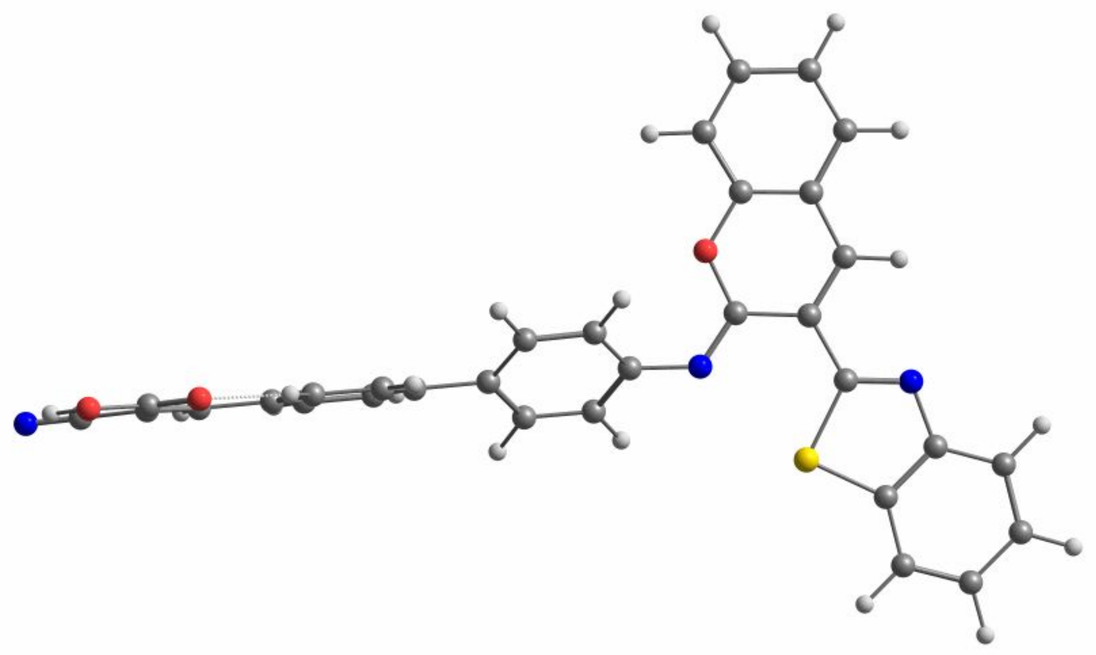

B1-*a*, MPP = 1.02

6	-5.082991000	1.892355000	-0.089343000
6	-4.332282000	0.768451000	-0.029942000
6	-2.876111000	0.882680000	0.074909000
8	-2.338486000	2.142715000	0.099529000
6	-3.095745000	3.278068000	0.050051000
6	-2.429225000	4.495280000	0.099574000
6	-3.175192000	5.664325000	0.047950000
6	-4.569616000	5.618547000	-0.051613000
6	-5.217824000	4.396226000	-0.098642000
6	-4.486482000	3.200784000	-0.047952000
7	-2.128701000	-0.139622000	0.160160000
6	-0.729247000	-0.105148000	0.221059000
6	-0.097964000	-0.919481000	1.166041000
6	1.285511000	-0.971115000	1.243258000
6	2.086718000	-0.240100000	0.358678000
6	1.446877000	0.548918000	-0.604453000
6	0.063843000	0.624842000	-0.671947000
6	3.562648000	-0.303804000	0.434538000
6	-4.991879000	-0.548469000	-0.074242000
7	-6.283846000	-0.636060000	-0.156998000
16	-4.131608000	-2.085683000	-0.031680000
6	-5.667968000	-2.898055000	-0.138625000
6	-6.702488000	-1.948352000	-0.194728000
6	-8.036052000	-2.368309000	-0.281074000
6	-5.942269000	-4.267195000	-0.167548000
6	-8.305649000	-3.723884000	-0.309619000

6	-7.265466000	-4.667635000	-0.253429000
6	4.351489000	-0.277883000	-0.723554000
6	5.734110000	-0.331122000	-0.668466000
6	6.393817000	-0.417544000	0.570895000
6	5.597369000	-0.448137000	1.733716000
6	4.218169000	-0.390693000	1.670292000
6	7.826064000	-0.481448000	0.803901000
6	8.964514000	-0.479034000	0.052680000
6	10.197091000	-0.562662000	0.785798000
7	11.228103000	-0.627306000	1.312197000
6	9.123438000	-0.397504000	-1.432413000
8	8.209576000	-0.322579000	-2.216144000
8	10.387239000	-0.409829000	-1.893447000
1	-6.160459000	1.794209000	-0.168287000
1	-1.348265000	4.507387000	0.179777000
1	-5.141215000	6.539151000	-0.090802000
1	-6.299668000	4.346360000	-0.174301000
1	-0.712139000	-1.513611000	1.833937000
1	1.749502000	-1.620009000	1.979189000
1	2.040240000	1.137217000	-1.297037000
1	-0.406527000	1.250878000	-1.421149000
1	-8.827242000	-1.627836000	-0.323639000
1	-5.142564000	-4.999161000	-0.124760000
1	-9.332794000	-4.067097000	-0.376424000
1	-7.502195000	-5.726306000	-0.277948000
1	3.870063000	-0.239739000	-1.694839000
1	6.314174000	-0.315658000	-1.579017000
1	8.044898000	-0.548282000	1.867775000
1	11.049571000	-0.472927000	-1.188090000
1	-2.667232000	6.622171000	0.086633000
1	3.640642000	-0.387769000	2.588295000
1	6.078157000	-0.507000000	2.706043000

Eelec = -2018.195719
$H_{(298.15\ K)}$ = -2017.727694
$G_{(298.15\ K)}$ = -2017.827297
ZPE (Zero-point energy correction) = 0.436308

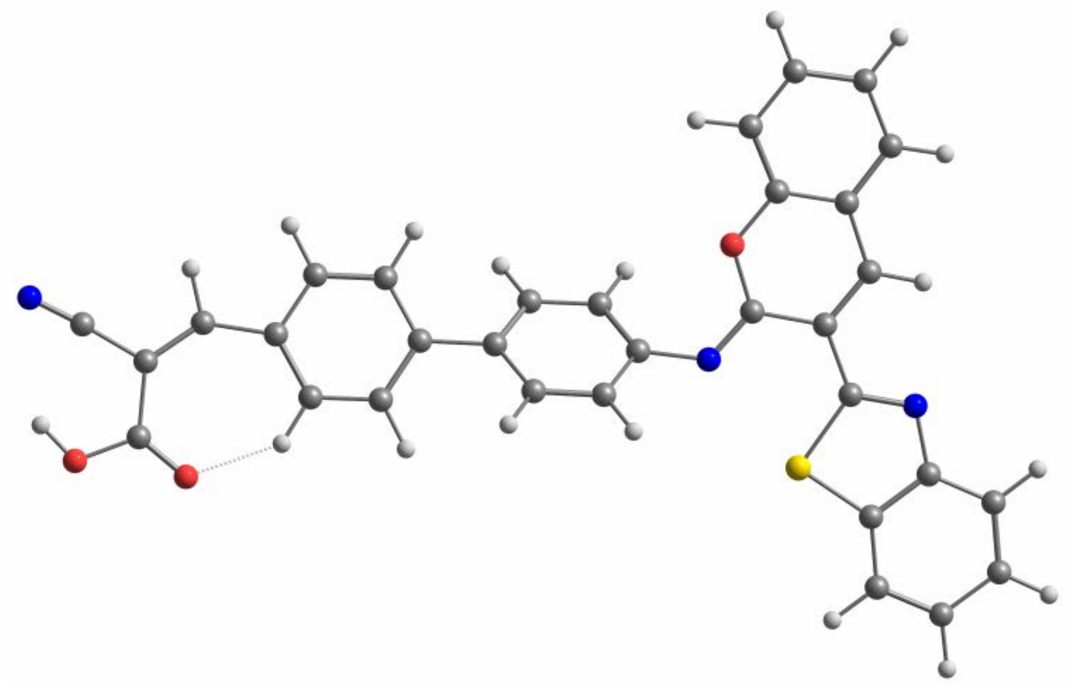

<u>B1-*b* MPP = 0.50</u>

6	5.229863000	1.725127000	0.056825000
6	4.385061000	0.668754000	0.034402000
6	2.939714000	0.904561000	0.050565000
8	2.509213000	2.205238000	0.080401000
6	3.360912000	3.272106000	0.116174000
6	2.797369000	4.540376000	0.164693000
6	3.640517000	5.642126000	0.197561000
6	5.029579000	5.479365000	0.182413000
6	5.574392000	4.207560000	0.135668000
6	4.743639000	3.078348000	0.102243000
7	2.106702000	-0.052969000	0.055524000
6	0.713443000	0.090805000	0.026133000
6	0.039827000	0.929118000	-0.870003000
6	-1.346535000	0.959068000	-0.894381000
6	-2.108087000	0.164221000	-0.029609000
6	-1.426044000	-0.677555000	0.856370000
6	-0.040510000	-0.723017000	0.877212000
6	-3.586588000	0.206189000	-0.058410000
6	4.933314000	-0.698416000	-0.009001000
7	6.216101000	-0.893902000	-0.015043000
16	3.946503000	-2.156907000	-0.064655000
6	5.413023000	-3.094698000	-0.100774000
6	6.524990000	-2.235899000	-0.065658000
6	7.821453000	-2.766298000	-0.083146000
6	5.572659000	-4.481155000	-0.153210000

6	7.977539000	-4.138846000	-0.135520000
6	6.860324000	-4.991184000	-0.170516000
6	-4.349240000	-0.952674000	0.139773000
6	-5.733640000	-0.930070000	0.118059000
6	-6.422033000	0.275439000	-0.108647000
6	-5.652054000	1.439165000	-0.309090000
6	-4.270754000	1.408341000	-0.284097000
6	-7.860245000	0.471865000	-0.161677000
6	-8.981289000	-0.296308000	-0.045968000
6	-10.231338000	0.398541000	-0.181311000
7	-11.274709000	0.894387000	-0.280061000
6	-9.105351000	-1.766266000	0.202396000
8	-8.172916000	-2.518192000	0.344129000
8	-10.358513000	-2.251294000	0.266538000
1	6.298128000	1.537034000	0.042187000
1	1.718353000	4.642993000	0.179386000
1	5.677996000	6.348068000	0.208810000
1	6.650842000	4.067075000	0.125498000
1	0.605346000	1.543468000	-1.560758000
1	-1.845769000	1.594235000	-1.619322000
1	-1.985684000	-1.295741000	1.550844000
1	0.480912000	-1.384756000	1.560250000
1	8.673126000	-2.095479000	-0.055489000
1	4.713218000	-5.142780000	-0.180430000
1	8.974410000	-4.566998000	-0.149901000
1	7.008384000	-6.065336000	-0.211613000
1	-3.845839000	-1.901651000	0.290154000
1	-6.293296000	-1.841516000	0.265807000
1	-8.104093000	1.516606000	-0.343964000
1	-11.037541000	-1.570725000	0.140283000
1	3.213545000	6.638696000	0.236801000
1	-3.713312000	2.329396000	-0.415344000
1	-6.155163000	2.387168000	-0.477019000

$Eelec$ = -2018.195890
$H_{(298.15\ K)}$ = -2017.727860
$G_{(298.15\ K)}$ = -2017.827348
ZPE (Zero-point energy correction) = 0.436325

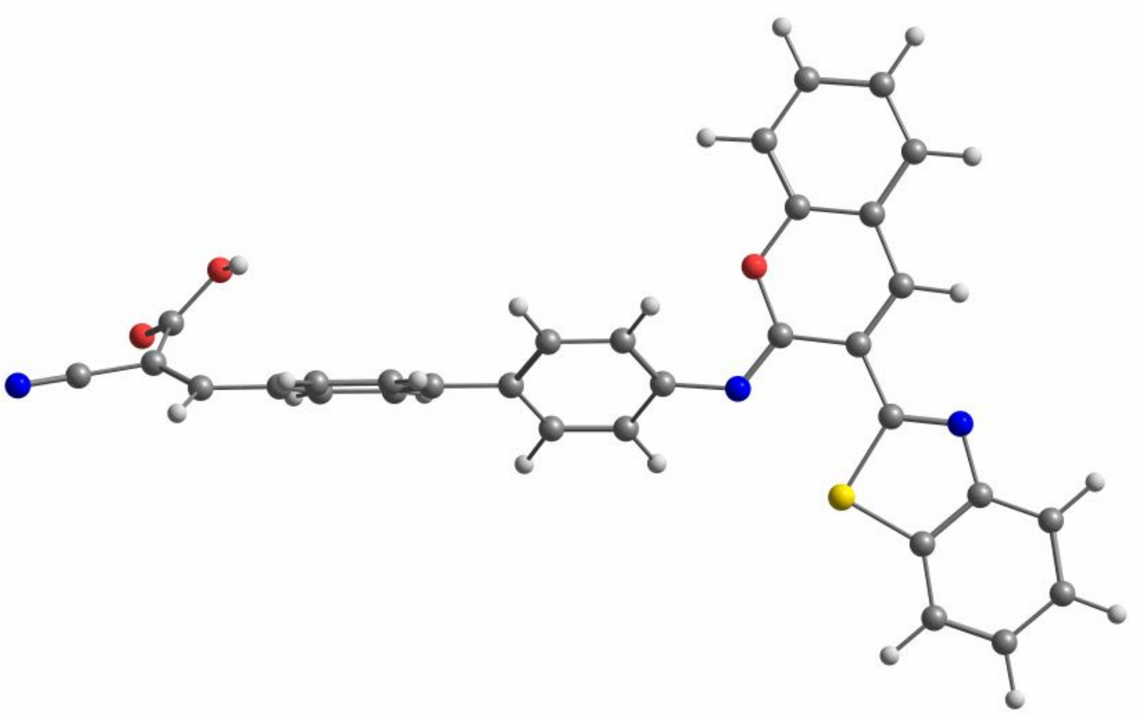

B1-*c*, MPP = 0.96

6	4.931771000	1.972349000	-0.063783000
6	4.228689000	0.816704000	-0.043101000
6	2.766627000	0.867512000	0.020774000
8	2.175387000	2.102614000	0.063482000
6	2.883356000	3.270134000	0.030285000
6	2.163314000	4.457067000	0.060756000
6	2.858964000	5.657514000	0.031930000
6	4.256362000	5.672475000	-0.026862000
6	4.958118000	4.479622000	-0.058297000
6	4.278247000	3.253438000	-0.030644000
7	2.059158000	-0.186327000	0.019187000
6	0.662301000	-0.212028000	0.124840000
6	-0.047308000	0.474596000	1.116829000
6	-1.423967000	0.337388000	1.215813000
6	-2.140527000	-0.472613000	0.327622000
6	-1.422491000	-1.159057000	-0.657664000
6	-0.043325000	-1.045131000	-0.748284000
6	-3.611257000	-0.605669000	0.434569000
6	4.945539000	-0.470226000	-0.080232000
7	6.241351000	-0.501208000	-0.139060000
16	4.153261000	-2.043707000	-0.041655000
6	5.725837000	-2.788035000	-0.109757000
6	6.718210000	-1.793963000	-0.157870000
6	8.070260000	-2.155245000	-0.218798000

6	6.060468000	-4.143892000	-0.121457000
6	8.399676000	-3.497799000	-0.229728000
6	7.401312000	-4.486109000	-0.181304000
6	-4.414405000	-0.686944000	-0.710324000
6	-5.791890000	-0.809335000	-0.617630000
6	-6.421732000	-0.851024000	0.633829000
6	-5.617403000	-0.803352000	1.781386000
6	-4.241857000	-0.670211000	1.683963000
6	-7.869633000	-1.003143000	0.803399000
6	-8.872541000	-0.561350000	0.015459000
6	-10.220039000	-0.927655000	0.346689000
7	-11.299350000	-1.217275000	0.646274000
6	-8.745638000	0.278364000	-1.230743000
8	-9.376682000	0.053453000	-2.226843000
8	-7.909419000	1.331175000	-1.165421000
1	6.014459000	1.921305000	-0.108243000
1	1.080680000	4.422068000	0.102335000
1	4.788300000	6.617085000	-0.049389000
1	6.042692000	4.476960000	-0.105937000
1	0.483192000	1.115149000	1.811757000
1	-1.954130000	0.893267000	1.982900000
1	-1.943884000	-1.821872000	-1.340917000
1	0.510010000	-1.604870000	-1.494449000
1	8.828682000	-1.380977000	-0.255845000
1	5.293207000	-4.910079000	-0.084240000
1	9.441894000	-3.795917000	-0.276364000
1	7.684940000	-5.533432000	-0.190982000
1	-3.953666000	-0.639832000	-1.691159000
1	-6.375084000	-0.891639000	-1.528179000
1	-8.171481000	-1.559848000	1.687787000
1	-7.479844000	1.393652000	-0.300553000
1	2.308952000	6.592385000	0.053824000
1	-3.643063000	-0.650541000	2.588215000
1	-6.080659000	-0.874115000	2.761400000

Eelec = -2018.190962
$H_{(298.15\ K)}$ = -2017.723291
$G_{(298.15\ K)}$ = -2017.823799
ZPE (Zero-point energy correction) = 0.435740

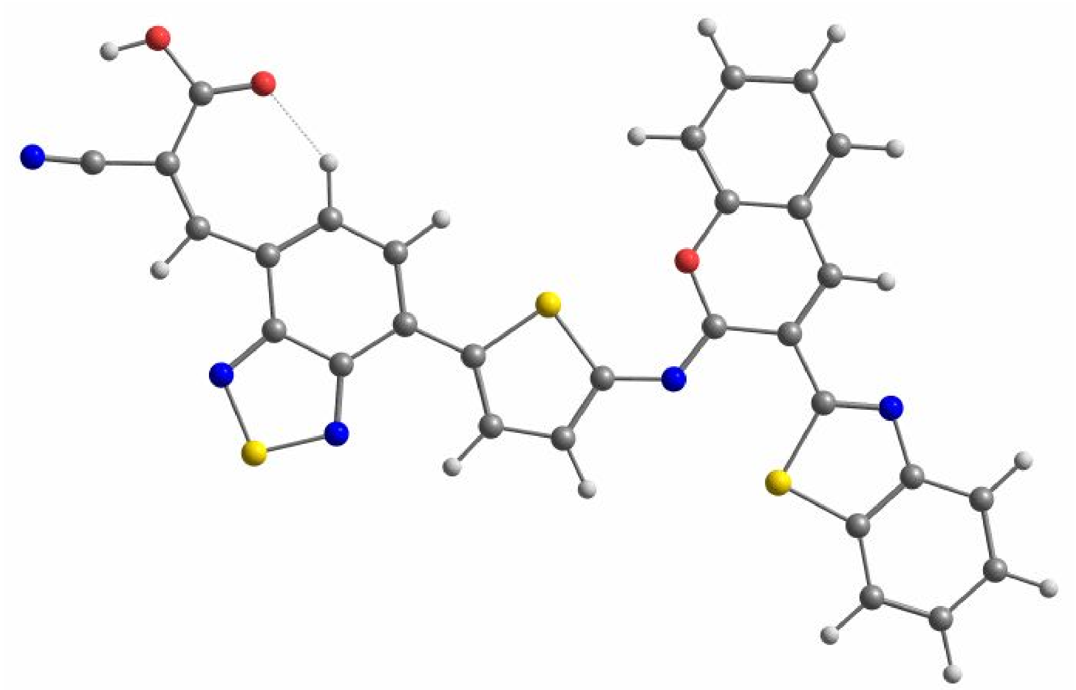

C1-*a*, MPP = 0.00

6	4.767096000	2.391597000	0.000036000
6	4.367061000	1.096937000	0.000002000
6	2.941151000	0.795840000	0.000031000
8	2.064686000	1.842778000	0.000108000
6	2.462184000	3.151903000	0.000135000
6	1.472530000	4.125004000	0.000201000
6	1.855614000	5.458604000	0.000229000
6	3.208886000	5.813575000	0.000193000
6	4.181533000	4.829145000	0.000129000
6	3.820843000	3.473380000	0.000099000
7	2.501015000	-0.402087000	-0.000015000
6	-2.739523000	-0.947936000	0.000105000
6	5.381484000	0.026983000	-0.000061000
7	6.644879000	0.320566000	-0.000107000
16	5.007605000	-1.694461000	-0.000069000
6	6.716935000	-2.022682000	-0.000147000
6	7.430310000	-0.811845000	-0.000157000
6	8.830992000	-0.823235000	-0.000216000
6	7.379664000	-3.251907000	-0.000197000
6	9.485574000	-2.040775000	-0.000264000
6	8.764739000	-3.247454000	-0.000254000
6	-3.319217000	0.307538000	0.000036000
6	-4.710798000	0.543210000	0.000022000
6	-5.645259000	-0.476568000	0.000048000
6	-5.097401000	-1.812144000	0.000165000

6	-3.673200000	-2.049990000	0.000202000
7	-3.367562000	-3.344570000	0.000330000
7	-5.810202000	-2.933243000	0.000260000
16	-4.761996000	-4.176570000	0.000424000
6	-7.085402000	-0.381564000	-0.000110000
6	-8.009476000	0.623986000	-0.000221000
6	-9.385895000	0.215142000	-0.000495000
7	-10.518751000	-0.032037000	-0.000691000
6	-7.797304000	2.101728000	-0.000110000
8	-6.714636000	2.638169000	0.000324000
8	-8.906081000	2.862271000	-0.000528000
1	5.830293000	2.606057000	0.000014000
1	0.430333000	3.828019000	0.000229000
1	1.094023000	6.231066000	0.000279000
1	3.494468000	6.859593000	0.000215000
1	5.234837000	5.091691000	0.000101000
1	9.372645000	0.116248000	-0.000224000
1	6.827713000	-4.185911000	-0.000188000
1	10.570191000	-2.068720000	-0.000310000
1	9.301275000	-4.190572000	-0.000292000
1	-2.678812000	1.182461000	-0.000029000
1	-5.061096000	1.565439000	0.000003000
1	-7.538714000	-1.370104000	-0.000185000
1	-9.723488000	2.340182000	-0.000837000
6	0.813657000	-2.121750000	-0.000054000
6	1.189822000	-0.796771000	0.000003000
16	-0.226329000	0.239075000	0.000101000
6	-1.301403000	-1.138204000	0.000070000
6	-0.580435000	-2.314798000	-0.000016000
1	1.547264000	-2.917250000	-0.000120000
1	-1.055388000	-3.284726000	-0.000046000

Eelec = -2845.417793
$H_{(298.15\ K)}$ = -2844.991739
$G_{(298.15\ K)}$ = -2845.096138
ZPE (Zero-point energy correction) = 0.392694

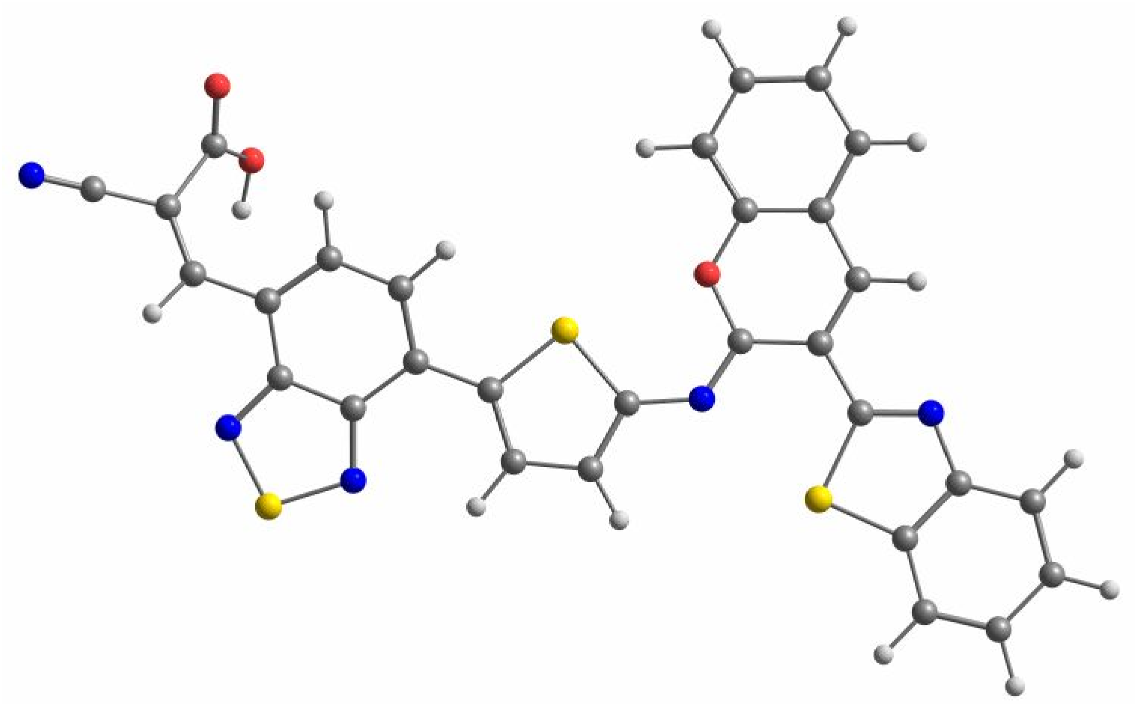

C1-*b*, MPP = 0.40

6	-4.688360000	2.385190000	0.018660000
6	-4.291763000	1.089816000	-0.000873000
6	-2.866562000	0.784873000	-0.023860000
8	-1.986846000	1.829822000	-0.020590000
6	-2.381341000	3.139791000	0.001163000
6	-1.389515000	4.110644000	0.003968000
6	-1.769100000	5.445162000	0.024783000
6	-3.121318000	5.803261000	0.042775000
6	-4.096287000	4.821025000	0.040757000
6	-3.739077000	3.464575000	0.020073000
7	-2.430178000	-0.413640000	-0.046998000
6	2.809263000	-0.979290000	-0.188820000
6	-5.308681000	0.022229000	0.000340000
7	-6.571262000	0.318323000	0.023209000
16	-4.938806000	-1.699935000	-0.028565000
6	-6.648681000	-2.024582000	-0.007363000
6	-7.359193000	-0.812364000	0.019629000
6	-8.759741000	-0.820861000	0.040574000
6	-7.314132000	-3.252321000	-0.013925000
6	-9.417024000	-2.036932000	0.033990000
6	-8.699040000	-3.245002000	0.006928000
6	3.388677000	0.251831000	-0.426825000
6	4.786286000	0.469433000	-0.475090000
6	5.706216000	-0.535464000	-0.279981000
6	5.162496000	-1.842298000	-0.052813000

6	3.738028000	-2.068916000	-0.002090000
7	3.432716000	-3.343503000	0.229711000
7	5.879777000	-2.945444000	0.140493000
16	4.827140000	-4.165417000	0.360536000
6	7.152185000	-0.382413000	-0.370107000
6	7.890655000	0.720245000	-0.120550000
6	9.297900000	0.701497000	-0.389273000
7	10.437697000	0.673551000	-0.587883000
6	7.360609000	2.037350000	0.390436000
8	7.539216000	3.080365000	-0.175908000
8	6.675318000	1.986172000	1.549130000
1	-5.750872000	2.602451000	0.034259000
1	-0.348101000	3.811197000	-0.009144000
1	-1.005624000	6.215764000	0.027359000
1	-3.404360000	6.849841000	0.058831000
1	-5.148879000	5.086018000	0.055362000
1	-9.299215000	0.119641000	0.061443000
1	-6.764387000	-4.187396000	-0.034835000
1	-10.501578000	-2.062609000	0.049880000
1	-9.237671000	-4.186913000	0.002242000
1	2.750392000	1.109103000	-0.608464000
1	5.123823000	1.472568000	-0.711948000
1	7.688432000	-1.272236000	-0.688882000
1	6.654013000	1.085698000	1.902893000
6	-0.748627000	-2.138680000	-0.083225000
6	-1.120532000	-0.813557000	-0.076397000
16	0.300552000	0.217590000	-0.106595000
6	1.368525000	-1.163316000	-0.135204000
6	0.645945000	-2.336631000	-0.112530000
1	-1.484541000	-2.931917000	-0.067300000
1	1.116777000	-3.308698000	-0.119388000

Eelec = -2845.410843
$H_{(298.15\ K)}$ = -2844.984998
$G_{(298.15\ K)}$ = -2845.089231
ZPE (Zero-point energy correction) = 0.392264

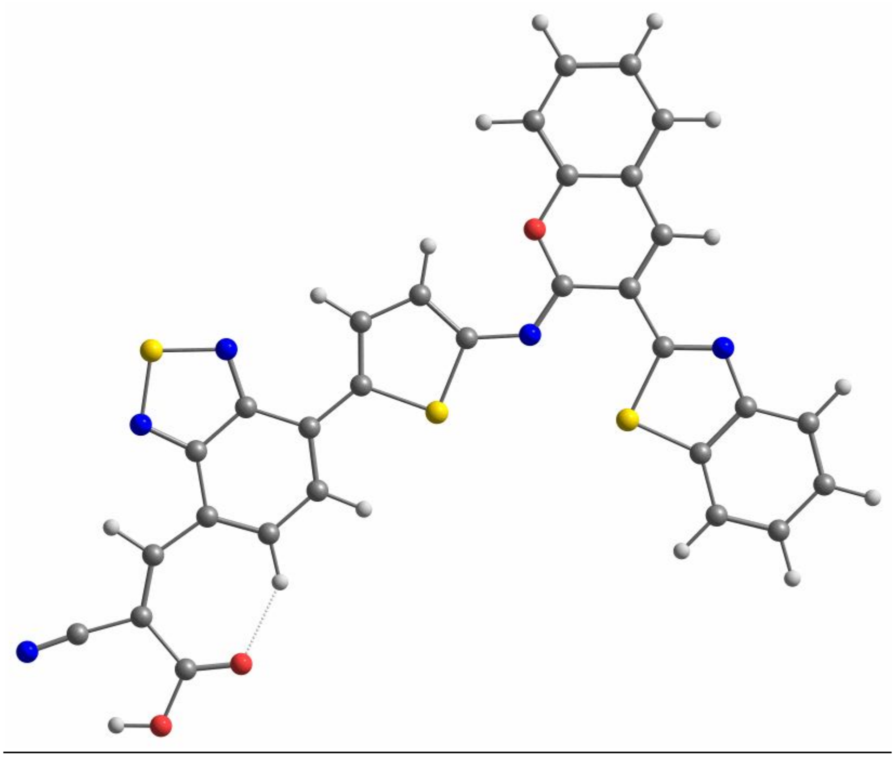

C1-*c*, MPP = 0.01

6	-5.906113000	-0.960617000	0.000565000
6	-4.754254000	-0.247680000	0.000280000
6	-3.478445000	-0.954834000	0.000741000
8	-3.503219000	-2.321110000	0.001727000
6	-4.664494000	-3.044850000	0.001954000
6	-4.558480000	-4.429182000	0.002793000
6	-5.722071000	-5.184841000	0.003028000
6	-6.976128000	-4.564902000	0.002443000
6	-7.063210000	-3.183836000	0.001624000
6	-5.901378000	-2.397521000	0.001368000
7	-2.367781000	-0.326411000	0.000270000
6	2.810573000	-0.701739000	0.000268000
6	-4.819075000	1.225951000	-0.000456000
7	-5.966860000	1.830788000	-0.000524000
16	-3.410323000	2.282211000	-0.001309000
6	-4.487433000	3.649107000	-0.001736000
6	-5.819387000	3.200938000	-0.001236000
6	-6.870652000	4.126806000	-0.001443000
6	-4.183219000	5.012172000	-0.002437000
6	-6.567994000	5.475569000	-0.002140000
6	-5.232936000	5.915613000	-0.002633000
6	3.280173000	0.597907000	0.002174000
6	4.647836000	0.950995000	0.002017000
6	5.664559000	0.013942000	0.000070000

6	5.232112000	-1.363358000	-0.001976000
6	3.833337000	-1.720999000	-0.001915000
7	3.638419000	-3.036968000	-0.004128000
7	6.037501000	-2.419854000	-0.004144000
16	5.098914000	-3.747577000	-0.006002000
6	7.092632000	0.228851000	-0.000211000
6	7.929544000	1.307307000	0.001272000
6	9.335307000	1.014268000	0.000151000
7	10.484662000	0.861472000	-0.000610000
6	7.594373000	2.762556000	0.003921000
8	6.470517000	3.205270000	0.005108000
8	8.635596000	3.613153000	0.005009000
1	-6.848874000	-0.424367000	0.000198000
1	-3.578328000	-4.891935000	0.003227000
1	-5.653947000	-6.267492000	0.003667000
1	-7.878173000	-5.166587000	0.002632000
1	-8.030024000	-2.690276000	0.001170000
1	-7.895259000	3.771492000	-0.001047000
1	-3.154399000	5.356391000	-0.002820000
1	-7.369379000	6.206994000	-0.002305000
1	-5.020886000	6.979737000	-0.003176000
1	2.567892000	1.415686000	0.004064000
1	4.909769000	1.999408000	0.003605000
1	7.626386000	-0.718631000	-0.002067000
1	9.493963000	3.161682000	0.003893000
6	-0.637491000	-2.179485000	0.002061000
6	-1.114669000	-0.884031000	0.000715000
16	0.199257000	0.259034000	-0.000772000
6	1.392507000	-1.012342000	0.000441000
6	0.772824000	-2.244342000	0.001888000
1	-1.278440000	-3.046799000	0.003159000
1	1.329316000	-3.170197000	0.002777000

Eelec = -2845.415840
$H_{(298.15\ K)}$ = -2844.989641
$G_{(298.15\ K)}$ = -2845.093644
ZPE (Zero-point energy correction) = 0.392838

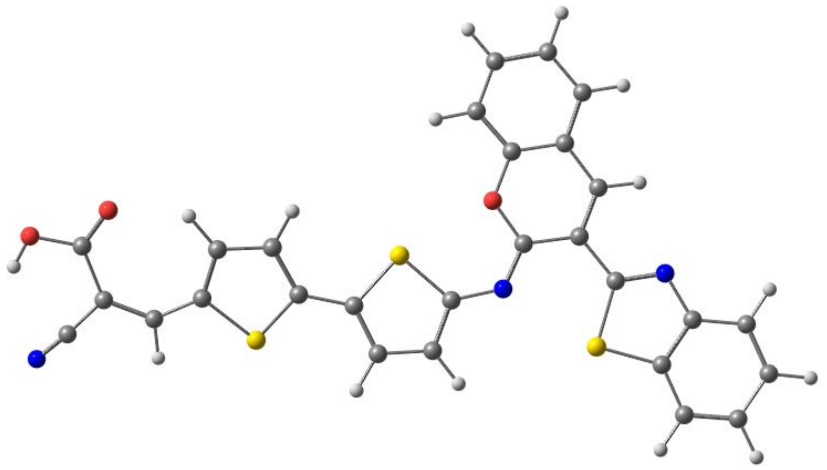

D1-*a*, MPP = 0.19

6	2.224924000	3.088496000	-0.072171000
6	1.312384000	4.133876000	-0.094949000
6	1.795698000	5.434165000	-0.130583000
6	3.171797000	5.685155000	-0.143322000
6	4.066691000	4.629574000	-0.120758000
6	3.603996000	3.305799000	-0.084799000
6	4.465988000	2.155501000	-0.060458000
6	3.969273000	0.895527000	-0.027311000
7	1.993968000	-0.456551000	0.014826000
6	2.524443000	0.702795000	-0.015068000
8	1.728768000	1.813694000	-0.036946000
6	0.655885000	-0.748811000	0.031204000
6	-1.847193000	-0.890128000	0.065321000
6	-1.229202000	-2.119037000	0.069457000
6	0.177790000	-2.039547000	0.050848000
6	-5.772518000	-0.713791000	-0.002952000
6	-5.285728000	0.549521000	0.297213000
6	-3.885019000	0.606931000	0.347446000
6	-3.264209000	-0.602838000	0.088276000
16	-4.434059000	-1.827560000	-0.234501000
6	-7.078485000	-1.267838000	-0.156173000
6	-8.351388000	-0.768369000	-0.081882000
6	4.900003000	-0.247762000	-0.004068000
16	4.397354000	-1.935683000	0.042202000
6	6.076928000	-2.392256000	0.045297000
6	6.879558000	-1.239160000	0.011015000
7	6.181921000	-0.050976000	-0.016082000
6	6.645078000	-3.667621000	0.075557000
6	8.026530000	-3.767754000	0.070977000

6	8.836239000	-2.619350000	0.036703000
6	8.275315000	-1.356259000	0.006652000
6	-8.760613000	0.630588000	0.211303000
8	-7.985739000	1.530088000	0.435786000
8	-10.083502000	0.879829000	0.220123000
6	-9.419951000	-1.693539000	-0.313184000
7	-10.335509000	-2.383677000	-0.489384000
1	0.250572000	3.917437000	-0.085465000
1	3.536099000	6.706046000	-0.171161000
1	5.136926000	4.810849000	-0.130707000
1	5.542288000	2.289013000	-0.069014000
1	-1.774641000	-3.055027000	0.101283000
1	0.847085000	-2.889739000	0.056574000
1	-5.944407000	1.385128000	0.478394000
1	-3.334630000	1.508858000	0.585346000
1	-7.060525000	-2.331332000	-0.387976000
1	6.024288000	-4.556965000	0.102094000
1	8.490421000	-4.748361000	0.094248000
1	9.915643000	-2.729085000	0.033905000
1	8.886183000	-0.460631000	-0.019987000
1	-10.616192000	0.092501000	0.029180000
16	-0.678185000	0.397340000	0.030889000
1	1.094979000	6.262060000	-0.148921000

E_{elec} = -2659.788596

$H_{(298.15\ K)}$ = -2659.388799

$G_{(298.15\ K)}$ = -2659.487103

ZPE (Zero-point energy correction) = 0.368639

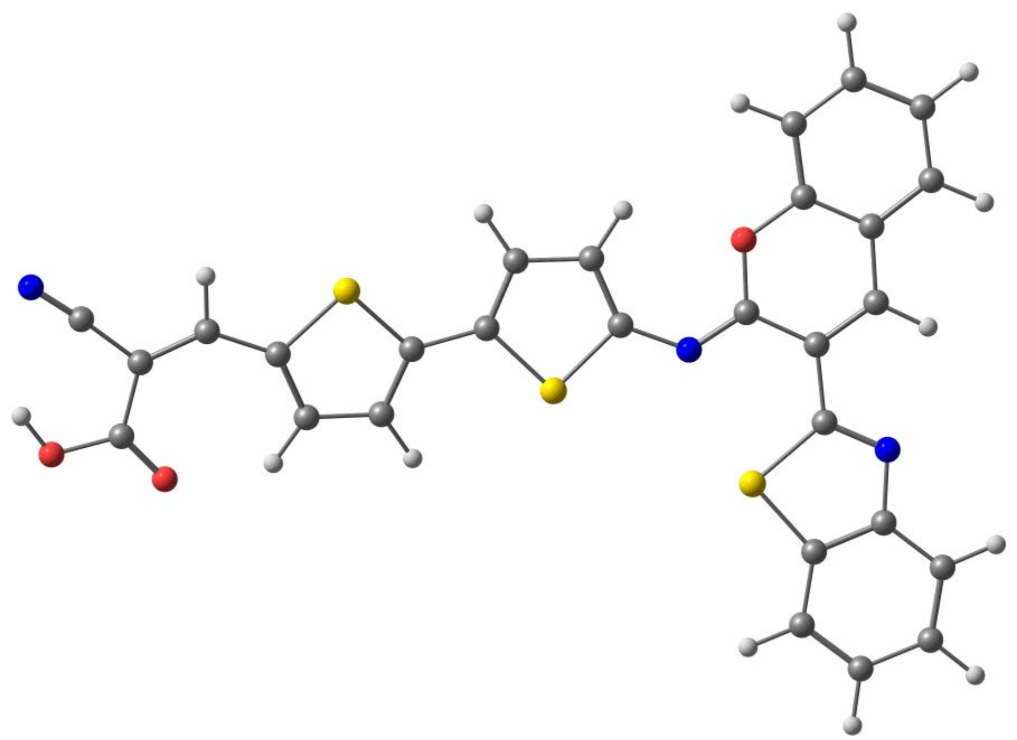

D1-*b*, MPP = 0.14

6	-3.903916000	-3.276032000	-0.040564000
6	-3.667519000	-4.643927000	-0.060451000
6	-4.754350000	-5.506433000	-0.069201000
6	-6.061241000	-5.007941000	-0.058175000
6	-6.278560000	-3.641313000	-0.038467000
6	-5.196414000	-2.748701000	-0.029364000
6	-5.337362000	-1.318641000	-0.009056000
6	-4.258361000	-0.499881000	-0.001646000
7	-1.874353000	-0.353428000	-0.009266000
6	-2.920956000	-1.082708000	-0.014299000
8	-2.816011000	-2.445769000	-0.032096000
6	-0.574446000	-0.791770000	-0.019043000
6	1.927109000	-0.680684000	-0.035167000
6	1.433586000	-1.964250000	-0.033601000
6	0.022125000	-2.036199000	-0.023874000
6	5.816548000	-0.120534000	0.017543000
6	5.203862000	1.101667000	-0.213340000
6	3.803491000	1.022187000	-0.250957000
6	3.309121000	-0.254571000	-0.051251000
16	4.597788000	-1.372586000	0.197582000
6	7.172347000	-0.550374000	0.135250000
6	8.389629000	0.073093000	0.071020000

6	-4.463097000	0.960758000	0.018925000
16	-3.161094000	2.146417000	0.035072000
6	-4.363308000	3.404661000	0.054922000
6	-5.646531000	2.831663000	0.047493000
7	-5.662991000	1.453830000	0.027249000
6	-4.190324000	4.790310000	0.075207000
6	-5.321390000	5.589525000	0.087931000
6	-6.608438000	5.024316000	0.080573000
6	-6.781202000	3.653022000	0.060458000
6	8.657034000	1.516442000	-0.166210000
8	7.794916000	2.344731000	-0.339705000
8	9.949488000	1.892795000	-0.183425000
6	9.545588000	-0.753171000	0.251061000
7	10.525224000	-1.359147000	0.387711000
1	-2.648008000	-5.011863000	-0.068907000
1	-4.584160000	-6.577669000	-0.084775000
1	-6.902370000	-5.692163000	-0.065177000
1	-7.287752000	-3.241528000	-0.029890000
1	-6.326801000	-0.874567000	0.000404000
1	2.070745000	-2.841234000	-0.051247000
1	-0.534517000	-2.959716000	-0.024694000
1	5.773794000	2.007456000	-0.353238000
1	3.163819000	1.877279000	-0.432583000
1	7.260703000	-1.619660000	0.319422000
1	-3.198958000	5.230892000	0.080971000
1	-5.211746000	6.668903000	0.103802000
1	-7.475839000	5.676015000	0.090875000
1	-7.767299000	3.201799000	0.054560000
1	10.559020000	1.153540000	-0.035078000
16	0.628863000	0.472207000	-0.021204000

$Eelec$ = -2659.7866384

$H_{(298.15\ K)}$ = -2659.386762

$G_{(298.15\ K)}$ = -2659.486036

ZPE (Zero-point energy correction) = 0.368669

Condensed phase global minima geometries for dyes of the proposed series A, C & D employing the IEFPCM model for solvent acetonitrile

A1, benzothiadiazole-containing dye with a H atom at the 7-coumarin position at the ground state employing the IEFPCM model and acetonitrile solvent

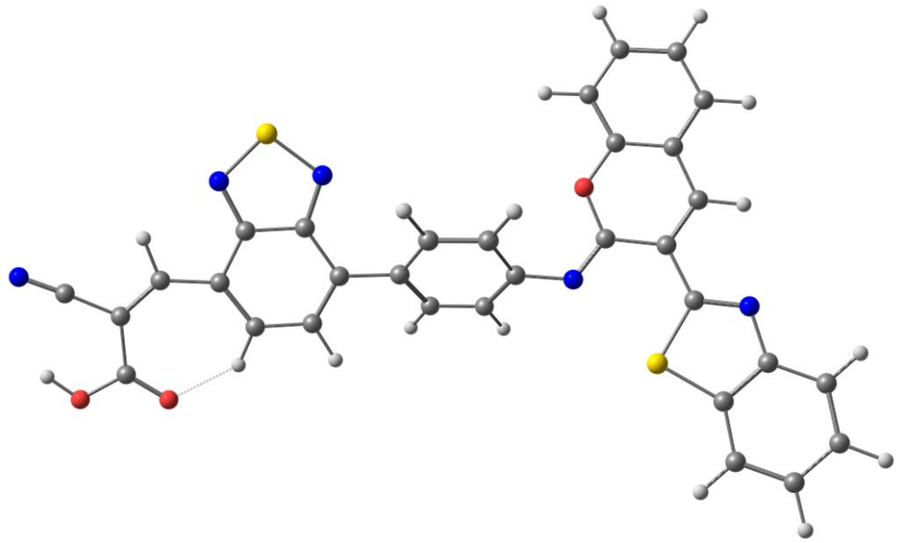

6	-5.632353000	1.695747000	0.127038000
6	-4.842449000	0.596020000	0.090562000
6	-3.389527000	0.759575000	0.148653000
8	-2.895948000	2.028188000	0.245965000
6	-3.690928000	3.140125000	0.284800000
6	-3.063114000	4.374444000	0.383758000
6	-3.851659000	5.516257000	0.424540000
6	-5.247539000	5.426357000	0.367371000
6	-5.856367000	4.187040000	0.269388000
6	-5.080533000	3.018261000	0.226842000
7	-2.597299000	-0.236410000	0.132205000
6	-1.199960000	-0.128272000	0.119655000
6	-0.467380000	-0.908159000	1.020768000
6	0.919198000	-0.893824000	1.001617000
6	1.621529000	-0.113942000	0.073177000
6	0.881519000	0.657473000	-0.833857000
6	-0.504798000	0.648696000	-0.816188000
6	3.095340000	-0.151003000	0.038392000
6	-5.455562000	-0.739074000	-0.015269000
7	-6.746085000	-0.881475000	-0.051063000
16	-4.533689000	-2.239232000	-0.111927000
6	-6.038065000	-3.112650000	-0.211920000
6	-7.111857000	-2.207745000	-0.161190000
6	-8.429810000	-2.680253000	-0.221540000
6	-6.253827000	-4.488297000	-0.321848000

6	-8.643127000	-4.043039000	-0.331142000
6	-7.562683000	-4.941381000	-0.381103000
6	3.796414000	-1.312756000	0.269565000
6	5.210399000	-1.395799000	0.246808000
6	6.020270000	-0.307653000	-0.017303000
6	5.330336000	0.938319000	-0.256635000
6	3.892394000	1.015154000	-0.229596000
7	3.435286000	2.248352000	-0.446916000
7	5.908007000	2.110344000	-0.503090000
16	4.721004000	3.209451000	-0.676642000
6	7.461952000	-0.252442000	-0.081490000
6	8.498509000	-1.131418000	0.059784000
6	9.809439000	-0.570169000	-0.103475000
7	10.890450000	-0.169440000	-0.225384000
6	8.466634000	-2.589098000	0.355906000
8	7.456610000	-3.234356000	0.535369000
8	9.650031000	-3.220393000	0.425493000
1	-6.708678000	1.573459000	0.078778000
1	-1.981258000	4.425395000	0.427659000
1	-3.375966000	6.488026000	0.502159000
1	-5.850237000	6.326980000	0.400470000
1	-6.937471000	4.102540000	0.225117000
1	-0.999408000	-1.518660000	1.742451000
1	1.462329000	-1.486713000	1.730122000
1	1.395719000	1.261432000	-1.571210000
1	-1.055468000	1.240440000	-1.538579000
1	-9.256032000	-1.978408000	-0.181795000
1	-5.422767000	-5.184482000	-0.360000000
1	-9.657028000	-4.426258000	-0.379134000
1	-7.755487000	-6.005630000	-0.467041000
1	3.247934000	-2.229067000	0.455706000
1	5.671974000	-2.353981000	0.432484000
1	7.798783000	0.757337000	-0.301827000
1	10.413575000	-2.641990000	0.270448000

Eelec = -2524.64256
$H_{(298.15\ K)}$ = -2524.183014
$G_{(298.15\ K)}$ = -2524.286958
ZPE (Zero-point energy correction) = 0.425865

A2, benzothiadiazole-containing dye with an OH substituent at the 7-coumarin position at the ground state employing the IEFPCM model and acetonitrile solvent

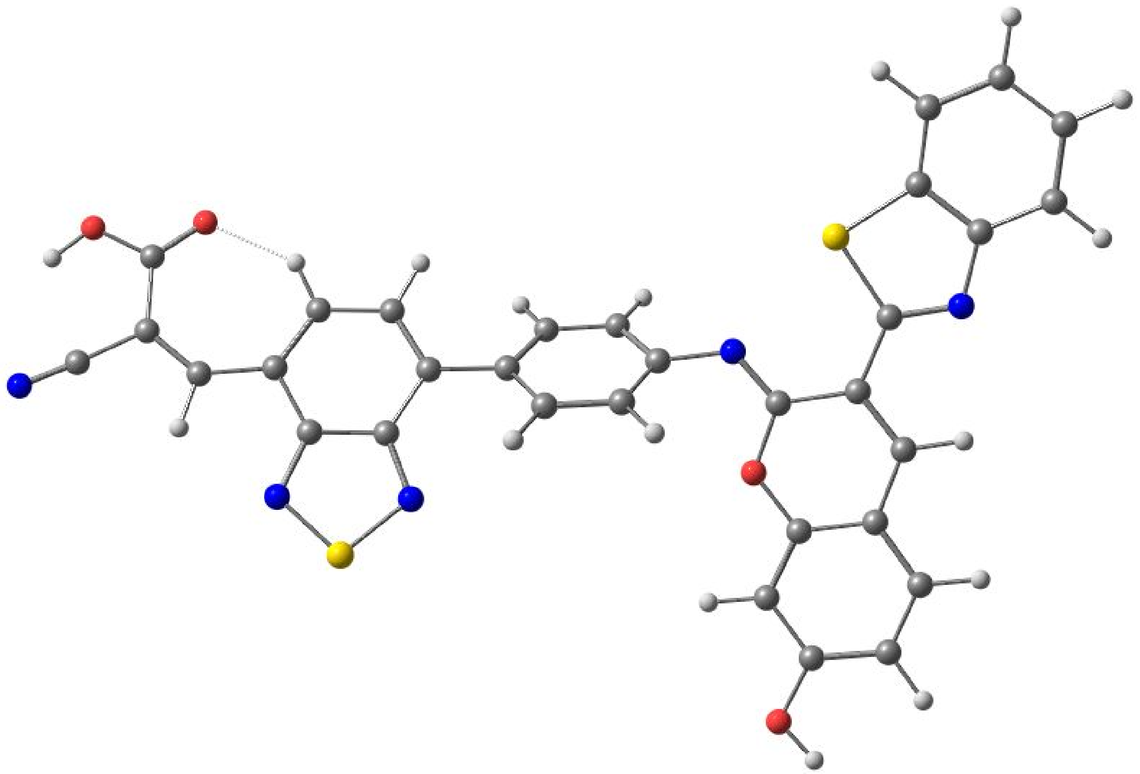

6	5.574886000	1.403343000	0.079936000
6	4.765600000	0.313861000	0.065145000
6	3.319040000	0.504239000	0.130789000
8	2.847784000	1.785443000	0.219286000
6	3.660218000	2.881716000	0.238326000
6	3.049182000	4.120011000	0.331381000
6	3.858580000	5.251516000	0.351755000
6	5.255904000	5.142273000	0.277874000
6	5.836500000	3.893009000	0.187241000
6	5.049224000	2.731146000	0.166612000
7	2.503554000	-0.473307000	0.129456000
6	1.109426000	-0.333145000	0.118772000
6	0.360174000	-1.075260000	1.037997000
6	-1.025790000	-1.029285000	1.019712000
6	-1.711327000	-0.254178000	0.074865000
6	-0.954962000	0.479696000	-0.849763000
6	0.430686000	0.438754000	-0.833387000
6	-3.185528000	-0.258219000	0.041393000
6	5.355231000	-1.030726000	-0.026293000

7	6.643021000	-1.198793000	-0.064284000
16	4.404734000	-2.515441000	-0.102077000
6	5.892483000	-3.418546000	-0.196509000
6	6.983057000	-2.533260000	-0.159800000
6	8.291341000	-3.031738000	-0.218428000
6	6.081814000	-4.798993000	-0.290604000
6	8.478744000	-4.399800000	-0.312377000
6	7.381746000	-5.278067000	-0.348415000
6	-3.912999000	-1.398440000	0.297231000
6	-5.328427000	-1.449821000	0.274783000
6	-6.113280000	-0.349427000	-0.013346000
6	-5.395089000	0.875276000	-0.278045000
6	-3.955776000	0.919923000	-0.251569000
7	-3.470611000	2.137536000	-0.494567000
7	-5.945876000	2.054598000	-0.549815000
16	-4.734105000	3.122550000	-0.745457000
6	-7.553277000	-0.262412000	-0.078187000
6	-8.609420000	-1.116326000	0.072093000
6	-9.907322000	-0.527298000	-0.097110000
7	-10.978847000	-0.103140000	-0.223409000
6	-8.610269000	-2.571678000	0.380781000
8	-7.615019000	-3.237418000	0.568008000
8	-9.807332000	-3.176483000	0.452645000
1	6.648517000	1.260627000	0.024350000
1	1.970888000	4.206200000	0.387962000
1	5.870521000	6.036354000	0.292655000
1	6.916107000	3.799557000	0.131770000
1	0.878957000	-1.681550000	1.772783000
1	-1.581537000	-1.592930000	1.761812000
1	-1.456134000	1.078975000	-1.599810000
1	0.994071000	1.000965000	-1.569519000
1	9.130978000	-2.345404000	-0.189507000
1	5.237460000	-5.479538000	-0.317983000
1	9.485214000	-4.802395000	-0.358757000
1	7.553906000	-6.346763000	-0.422161000
1	-3.385498000	-2.322755000	0.503394000
1	-5.811816000	-2.393127000	0.480126000
1	-7.867165000	0.752168000	-0.310195000
1	-10.557906000	-2.583225000	0.290516000
8	3.235188000	6.450775000	0.445707000
1	3.877503000	7.173451000	0.459163000

$Eelec$ = -2599.853994

$H_{(298.15\ K)}$ = -2599.389135

$G_{(298.15\ K)}$ = -2599.495823
ZPE (Zero-point energy correction) = 0.429976

A3, benzothiadiazole-containing dye with an NH2 substituent at the 7-coumarin position at the ground state employing the IEFPCM model and acetonitrile solvent

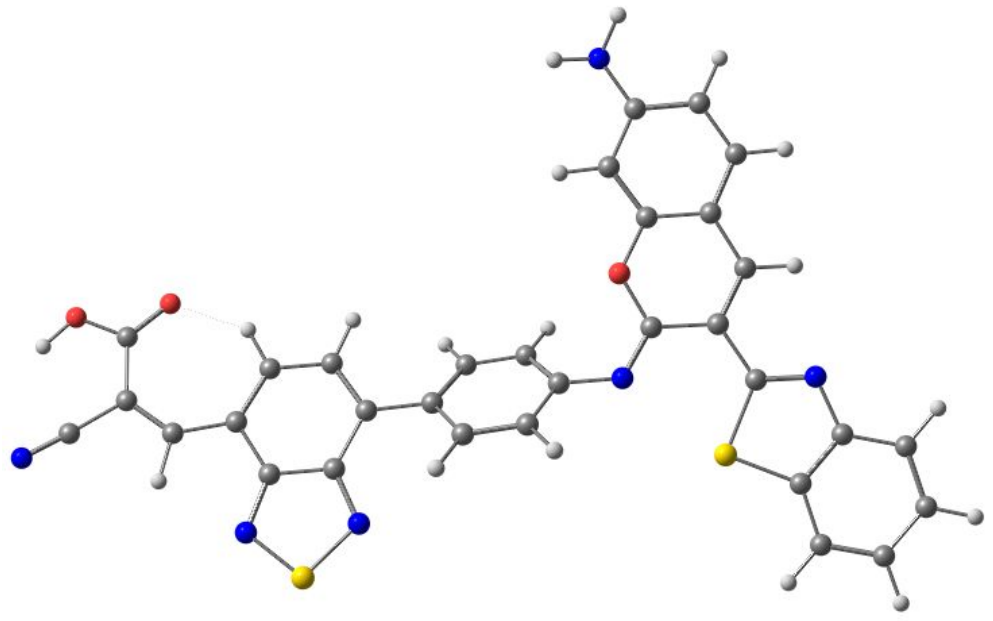

6	5.437889000	1.854521000	0.047453000
6	4.726026000	0.694502000	0.013783000
6	3.271235000	0.759581000	0.067480000
8	2.690649000	1.995166000	0.158692000
6	3.404128000	3.159631000	0.193875000
6	2.686182000	4.336107000	0.287822000
6	3.380392000	5.553543000	0.327976000
6	4.795383000	5.545634000	0.272666000
6	5.482335000	4.357935000	0.180717000
6	4.802975000	3.125610000	0.138060000
7	2.539261000	-0.284383000	0.055842000
6	1.139567000	-0.262935000	0.040420000
6	0.396244000	0.469345000	-0.895612000
6	-0.987230000	0.386493000	-0.912028000
6	-1.679727000	-0.427246000	-0.003728000
6	-0.929977000	-1.171537000	0.916803000
6	0.454613000	-1.092913000	0.935381000
6	-3.153567000	-0.459579000	-0.014378000
6	5.432078000	-0.589701000	-0.080900000

7	6.730169000	-0.646623000	-0.125705000
16	4.614206000	-2.153743000	-0.146088000
6	6.175039000	-2.924632000	-0.242990000
6	7.184199000	-1.947155000	-0.216836000
6	8.530305000	-2.330787000	-0.279929000
6	6.483315000	-4.283629000	-0.330717000
6	8.835964000	-3.678228000	-0.366877000
6	7.819879000	-4.648893000	-0.392260000
6	-3.900016000	0.669242000	-0.267697000
6	-5.315983000	0.688275000	-0.287146000
6	-6.083617000	-0.435498000	-0.045959000
6	-5.345222000	-1.649242000	0.213671000
6	-3.904940000	-1.661017000	0.229878000
7	-3.399614000	-2.872883000	0.460145000
7	-5.876524000	-2.846521000	0.442221000
16	-4.646869000	-3.891384000	0.650768000
6	-7.522320000	-0.554632000	-0.019826000
6	-8.592925000	0.277153000	-0.191975000
6	-9.881533000	-0.340214000	-0.056518000
7	-10.946205000	-0.788264000	0.042438000
6	-8.617761000	1.732715000	-0.498140000
8	-7.632930000	2.420867000	-0.657518000
8	-9.825580000	2.310950000	-0.602326000
1	6.520474000	1.804320000	0.003540000
1	1.603118000	4.305636000	0.329073000
1	5.332763000	6.487591000	0.303958000
1	6.567147000	4.360682000	0.141860000
1	0.907301000	1.093730000	-1.619556000
1	-1.537477000	0.947352000	-1.660498000
1	-1.433688000	-1.812420000	1.629677000
1	1.025124000	-1.674084000	1.652031000
1	9.307451000	-1.574093000	-0.259511000
1	5.701051000	-5.035084000	-0.350113000
1	9.873622000	-3.991578000	-0.416228000
1	8.083856000	-5.699063000	-0.460895000
1	-3.388241000	1.609535000	-0.438166000
1	-5.814651000	1.624753000	-0.487065000
1	-7.819571000	-1.577625000	0.196789000
1	-10.567123000	1.701013000	-0.462029000
7	2.700771000	6.740565000	0.379502000
1	3.205572000	7.575568000	0.635638000
1	1.726180000	6.732008000	0.639910000

Eelec = -2579.9912928

$H_{(298.15\ K)}$ = -2579.513922
$G_{(298.15\ K)}$ = -2579.621142
ZPE (Zero-point energy correction) = 0.442051
A4, benzothiadiazole-containing dye with a CF3 substituent at the 7-coumarin position at the ground state employing the IEFPCM model and acetonitrile solvent

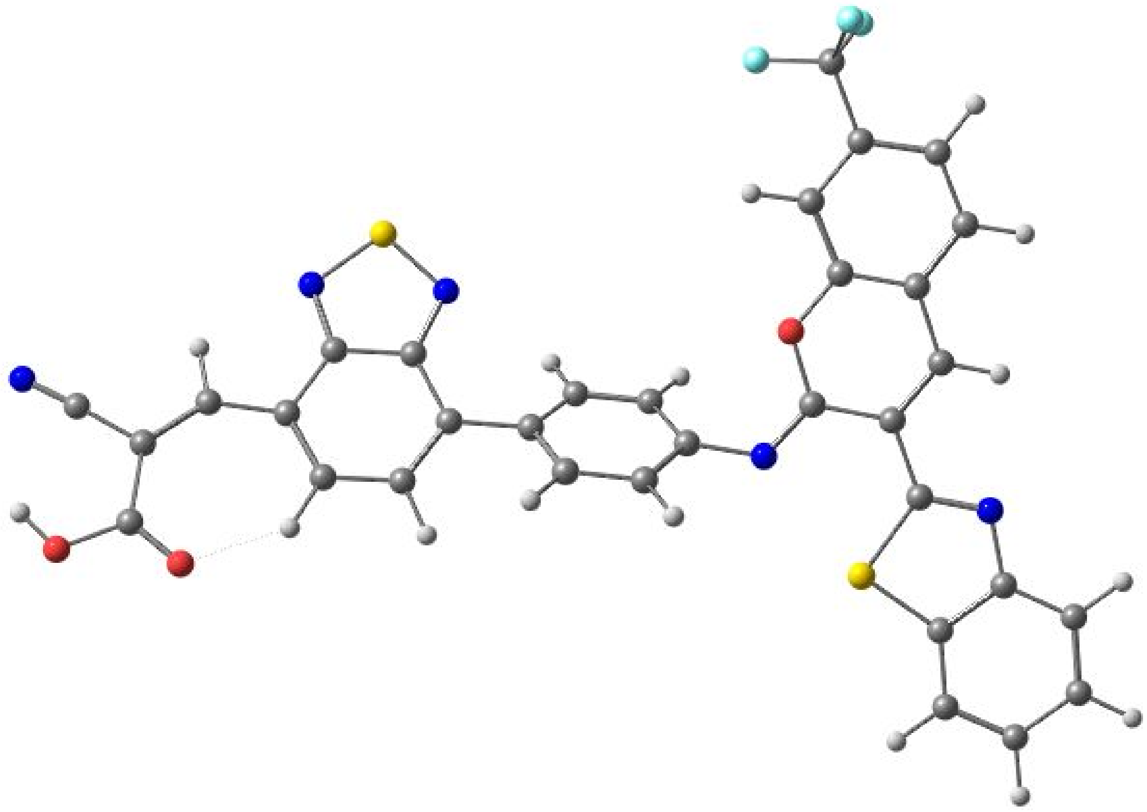

6	5.393524000	0.544396000	0.060391000
6	4.527456000	-0.494309000	0.033398000
6	3.087003000	-0.227686000	0.067774000
8	2.683268000	1.077268000	0.128220000
6	3.555474000	2.124839000	0.157016000
6	3.012676000	3.400882000	0.218617000
6	3.883292000	4.479737000	0.246817000
6	5.270763000	4.297716000	0.215679000
6	5.787420000	3.017237000	0.154727000
6	4.932438000	1.906119000	0.125051000
7	2.226165000	-1.161626000	0.064116000
6	0.840073000	-0.942441000	0.041001000
6	0.049078000	-1.574977000	1.004454000
6	-1.331133000	-1.438007000	0.981232000

6	-1.965846000	-0.681102000	-0.012241000
6	-1.166545000	-0.061594000	-0.982881000
6	0.213629000	-0.192407000	-0.961616000
6	-3.437452000	-0.583034000	-0.041594000
6	5.043902000	-1.872912000	-0.037605000
7	6.321526000	-2.104053000	-0.060872000
16	4.020806000	-3.305915000	-0.104935000
6	5.460661000	-4.283168000	-0.173023000
6	6.594934000	-3.454373000	-0.136556000
6	7.877329000	-4.018483000	-0.177355000
6	5.580138000	-5.672765000	-0.250290000
6	7.995110000	-5.394706000	-0.254027000
6	6.854442000	-6.216517000	-0.290513000
6	-4.241760000	-1.652282000	0.280044000
6	-5.657816000	-1.601510000	0.271493000
6	-6.363795000	-0.464170000	-0.070690000
6	-5.562129000	0.687641000	-0.411375000
6	-4.123322000	0.629692000	-0.395510000
7	-3.554233000	1.793717000	-0.709235000
7	-6.029557000	1.886876000	-0.745331000
16	-4.746270000	2.851750000	-1.008478000
6	-7.794654000	-0.280970000	-0.147644000
6	-8.908017000	-1.023432000	0.127105000
6	-10.161597000	-0.372568000	-0.128625000
7	-11.199525000	0.107899000	-0.317791000
6	-9.009517000	-2.404641000	0.671092000
8	-8.061892000	-3.114166000	0.930047000
8	-10.245530000	-2.882864000	0.888189000
1	6.458961000	0.346747000	0.031903000
1	1.937852000	3.530231000	0.241002000
1	5.934523000	5.154138000	0.235803000
1	6.860245000	2.860141000	0.128028000
1	0.529084000	-2.164920000	1.777852000
1	-1.921170000	-1.913843000	1.757502000
1	-1.629752000	0.519753000	-1.770507000
1	0.812259000	0.282877000	-1.730754000
1	8.750076000	-3.374901000	-0.148679000
1	4.702693000	-6.309760000	-0.278720000
1	8.979820000	-5.848938000	-0.286776000
1	6.973137000	-7.293263000	-0.350967000
1	-3.779804000	-2.599834000	0.532871000
1	-6.205371000	-2.494565000	0.532673000
1	-8.035931000	0.712677000	-0.516584000
1	-10.953918000	-2.260055000	0.660537000

6	3.338742000	5.874971000	0.350601000
9	3.966826000	6.726840000	-0.487970000
9	2.023660000	5.942636000	0.070779000
9	3.497325000	6.384692000	1.595178000

Eelec = -2861.6256678
$H_{(298.15\ K)}$ = -2861.158069
$G_{(298.15\ K)}$ = -2861.272029
ZPE (Zero-point energy correction) = 0.430146

A5, benzothiadiazole-containing dye with an NO2 substituent at the 7-coumarin position at the ground state employing the IEFPCM model and acetonitrile solvent

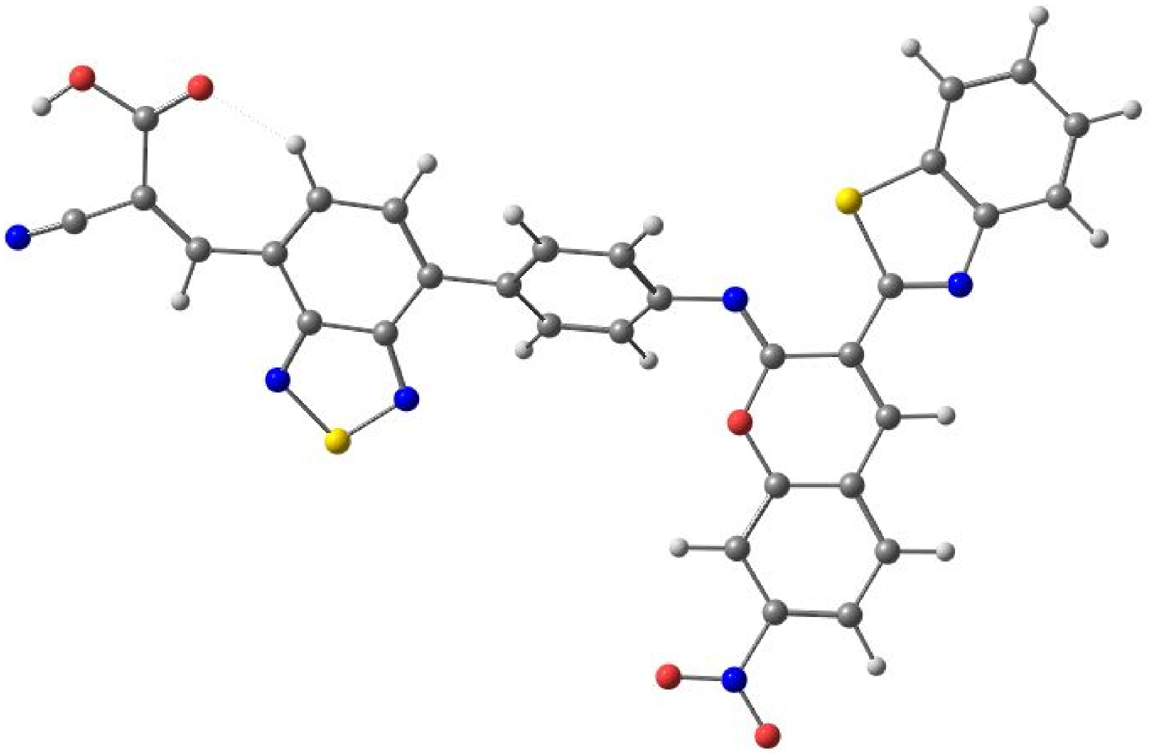

6	5.344663000	4.612968000	-0.194928000
6	3.960246000	4.762315000	-0.245127000
6	3.081642000	3.691375000	-0.232193000
6	3.634123000	2.423066000	-0.166195000
6	5.015418000	2.217328000	-0.115701000
6	5.863754000	3.333102000	-0.130280000
8	2.772056000	1.368525000	-0.152817000
6	3.187880000	0.066825000	-0.087532000

6	4.631374000	-0.185984000	-0.039241000
6	5.488963000	0.859486000	-0.051391000
7	2.339202000	-0.876718000	-0.091328000
6	0.948940000	-0.684683000	-0.080060000
6	0.177428000	-1.387626000	-1.010082000
6	-1.205617000	-1.284093000	-0.997887000
6	-1.862464000	-0.492171000	-0.046995000
6	-1.082458000	0.199408000	0.889982000
6	0.300754000	0.103432000	0.878564000
6	-3.336465000	-0.434915000	-0.021417000
6	-4.108739000	-1.542667000	-0.285758000
6	-5.525635000	-1.534677000	-0.271767000
6	-6.264875000	-0.403358000	0.015767000
6	-5.497584000	0.789160000	0.289457000
6	-4.057652000	0.773422000	0.271620000
6	-7.700757000	-0.256729000	0.076664000
6	-8.790644000	-1.063814000	-0.088493000
6	5.159147000	-1.560559000	0.031194000
7	6.439072000	-1.777908000	0.060799000
6	6.726074000	-3.124986000	0.135927000
6	5.599966000	-3.965374000	0.165196000
16	4.150722000	-3.003356000	0.090674000
6	8.014284000	-3.675706000	0.182596000
6	8.145663000	-5.050484000	0.257859000
6	7.013061000	-5.884061000	0.287070000
6	5.733461000	-5.353937000	0.240945000
7	-5.999892000	1.989557000	0.562906000
16	-4.745581000	3.004625000	0.770651000
7	-3.523028000	1.968413000	0.522953000
7	3.398911000	6.113506000	-0.315619000
8	2.180865000	6.231008000	-0.366093000
8	4.175279000	7.060485000	-0.320744000
6	-8.850772000	-2.512957000	-0.420936000
8	-7.883057000	-3.216068000	-0.615248000
8	-10.071394000	-3.066105000	-0.507799000
6	-10.063728000	-0.424611000	0.087814000
7	-11.116708000	0.042063000	0.219531000
1	5.986687000	5.482974000	-0.207454000
1	2.010172000	3.833377000	-0.272557000
1	6.936799000	3.182947000	-0.090860000
1	6.555643000	0.671836000	-0.011242000
1	0.675078000	-2.007083000	-1.748388000
1	-1.780817000	-1.816043000	-1.748376000
1	-1.562807000	0.810226000	1.644307000

1	0.883351000	0.634302000	1.622895000
1	-3.619087000	-2.487405000	-0.492875000
1	-6.046841000	-2.456093000	-0.483683000
1	-7.972574000	0.767104000	0.320961000
1	8.880356000	-3.023003000	0.159448000
1	9.134673000	-5.494841000	0.295139000
1	7.142712000	-6.959583000	0.346542000
1	4.862644000	-6.000120000	0.263718000
1	-10.797828000	-2.445003000	-0.339898000

Eelec = -2729.0886902
$H_{(298.15\ K)}$ = -2728.624098
$G_{(298.15\ K)}$ = -2728.735767
ZPE (Zero-point energy correction) = 0.428199

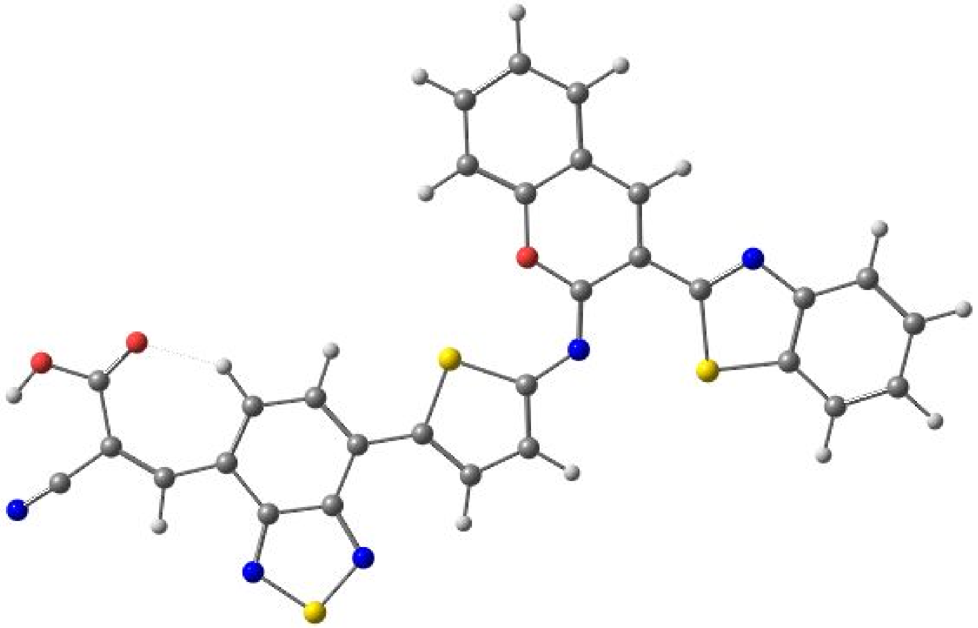

C1, benzothiadiazole- and thiophene-containing dye with a H at the 7-coumarin position at the ground state employing the IEFPCM model and acetonitrile solvent

6	4.753899000	2.401653000	0.030659000
6	4.364382000	1.102188000	0.011223000
6	2.941513000	0.793418000	0.017300000
8	2.060218000	1.828589000	0.045820000
6	2.445134000	3.143209000	0.066298000
6	1.446461000	4.106316000	0.093708000
6	1.821314000	5.442592000	0.113543000
6	3.172828000	5.808818000	0.106230000
6	4.154216000	4.833648000	0.079116000

6	3.801317000	3.474706000	0.058636000
7	2.506411000	-0.410319000	-0.003594000
6	-2.736656000	-0.949805000	-0.005189000
6	5.384843000	0.038810000	-0.015461000
7	6.649835000	0.331251000	-0.016643000
16	5.014871000	-1.684336000	-0.047607000
6	6.725263000	-2.013202000	-0.063676000
6	7.437272000	-0.802009000	-0.043479000
6	8.838499000	-0.812533000	-0.051031000
6	7.386730000	-3.243034000	-0.091541000
6	9.493604000	-2.030973000	-0.078579000
6	8.772968000	-3.238117000	-0.098736000
6	-3.311493000	0.307684000	-0.075995000
6	-4.700738000	0.542692000	-0.085316000
6	-5.638860000	-0.474914000	-0.028003000
6	-5.093861000	-1.809876000	0.048603000
6	-3.671817000	-2.047863000	0.062340000
7	-3.367535000	-3.341181000	0.144148000
7	-5.806294000	-2.929946000	0.117444000
16	-4.759809000	-4.172137000	0.194445000
6	-7.074213000	-0.375441000	-0.044570000
6	-8.003796000	0.629492000	-0.079821000
6	-9.373090000	0.202997000	-0.086807000
7	-10.496164000	-0.086016000	-0.093947000
6	-7.800204000	2.100561000	-0.103073000
8	-6.718745000	2.648999000	-0.113859000
8	-8.904200000	2.867156000	-0.110340000
1	5.813129000	2.632665000	0.025366000
1	0.405569000	3.804777000	0.098840000
1	1.054303000	6.209181000	0.134776000
1	3.449166000	6.857052000	0.121768000
1	5.204940000	5.105009000	0.073207000
1	9.384402000	0.124738000	-0.035230000
1	6.834327000	-4.176468000	-0.107144000
1	10.578192000	-2.058094000	-0.084717000
1	9.309503000	-4.180888000	-0.120277000
1	-2.670778000	1.180202000	-0.134205000
1	-5.047005000	1.563658000	-0.144255000
1	-7.529814000	-1.362178000	-0.020524000
1	-9.732199000	2.361315000	-0.099201000
6	0.815863000	-2.129481000	-0.024418000
6	1.194435000	-0.803722000	-0.004484000
16	-0.220955000	0.234115000	0.018873000
6	-1.299430000	-1.142488000	-0.000575000

6	-0.578756000	-2.319874000	-0.021332000
1	1.544562000	-2.929768000	-0.041642000
1	-1.051717000	-3.290677000	-0.036390000

Eelec = -2845.4385023
$H_{(298.15\ K)}$ = -2845.013112
$G_{(298.15\ K)}$ = -2845.116630
ZPE (Zero-point energy correction) = 0.392000

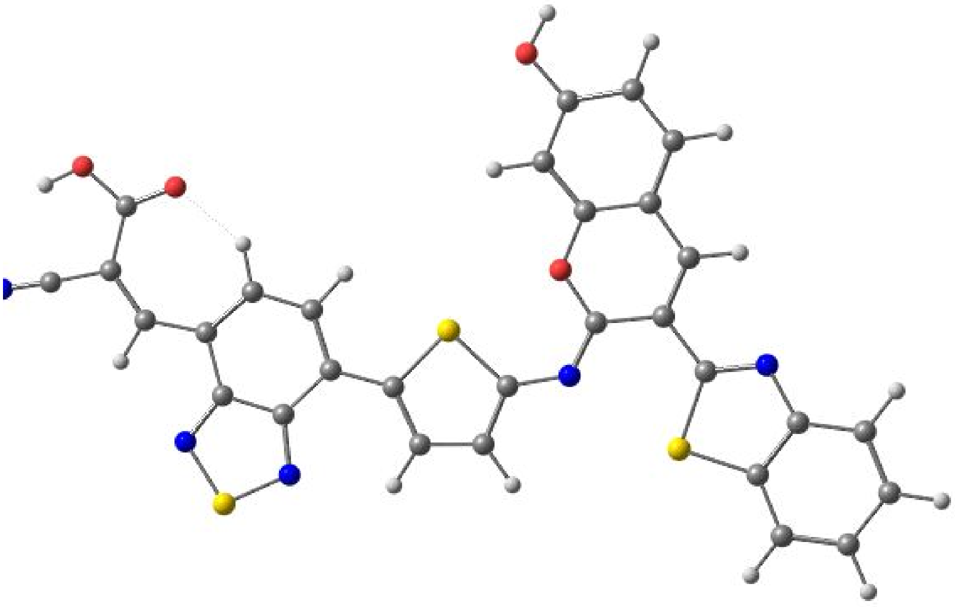

C2, benzothiadiazole- and thiophene-containing dye with an OH at the 7-coumarin position at the ground state employing the IEFPCM model and acetonitrile solvent

6	4.742982000	2.208664000	0.016465000
6	4.348836000	0.907409000	0.002139000
6	2.927528000	0.606439000	0.007930000
8	2.048850000	1.648121000	0.030529000
6	2.437908000	2.958689000	0.046363000
6	1.440762000	3.917070000	0.069403000
6	1.821048000	5.255529000	0.085341000
6	3.176770000	5.621600000	0.077894000
6	4.147716000	4.641720000	0.055019000
6	3.799202000	3.281186000	0.038800000
7	2.481341000	-0.593944000	-0.007641000
6	-2.764839000	-1.100999000	-0.005799000

6	5.365256000	-0.157477000	-0.018179000
7	6.632132000	0.128517000	-0.019944000
16	4.987692000	-1.880089000	-0.041003000
6	6.697107000	-2.217061000	-0.053719000
6	7.414265000	-1.008837000	-0.039759000
6	8.815278000	-1.026204000	-0.046063000
6	7.352880000	-3.449838000	-0.074013000
6	9.465166000	-2.247914000	-0.066138000
6	8.739410000	-3.451754000	-0.080039000
6	-3.332522000	0.160254000	-0.073462000
6	-4.720086000	0.403370000	-0.082056000
6	-5.664596000	-0.608779000	-0.026762000
6	-5.127469000	-1.947158000	0.046599000
6	-3.706794000	-2.193647000	0.059282000
7	-3.410217000	-3.488917000	0.137584000
7	-5.846491000	-3.063116000	0.112993000
16	-4.807402000	-4.311816000	0.186525000
6	-7.098995000	-0.500576000	-0.041787000
6	-8.022751000	0.510060000	-0.075790000
6	-9.394538000	0.091873000	-0.080969000
7	-10.519422000	-0.190197000	-0.086629000
6	-7.810307000	1.979684000	-0.099815000
8	-6.725610000	2.521737000	-0.111948000
8	-8.909760000	2.752998000	-0.106347000
1	5.803554000	2.434129000	0.011526000
1	0.394973000	3.635756000	0.075018000
1	3.453985000	6.670421000	0.090696000
1	5.196844000	4.918295000	0.049434000
1	9.365633000	-0.091432000	-0.035125000
1	6.796129000	-4.380800000	-0.084747000
1	10.549667000	-2.279669000	-0.071184000
1	9.271512000	-4.397156000	-0.095704000
1	-2.686660000	1.029146000	-0.129092000
1	-5.060259000	1.426541000	-0.138451000
1	-7.560588000	-1.484547000	-0.017662000
1	-9.740628000	2.251932000	-0.094277000
6	0.780118000	-2.302075000	-0.021638000
6	1.167416000	-0.978378000	-0.007280000
16	-0.242022000	0.068150000	0.011527000
6	-1.329344000	-1.302124000	-0.001794000
6	-0.615363000	-2.483859000	-0.017784000
1	1.503884000	-3.106892000	-0.035293000
1	-1.094098000	-3.451894000	-0.028645000
8	0.828197000	6.175293000	0.108008000

1	1.186689000	7.073465000	0.119919000

Eelec = -2920.6499841
$H_{(298.15\ K)}$ = -2920.219453
$G_{(298.15\ K)}$ = -2920.325653
ZPE (Zero-point energy correction) = 0.395878

<u>C3, benzothiadiazole- and thiophene-containing dye with a NH2 at the 7-coumarin position at the ground state employing the IEFPCM model and acetonitrile solvent</u>

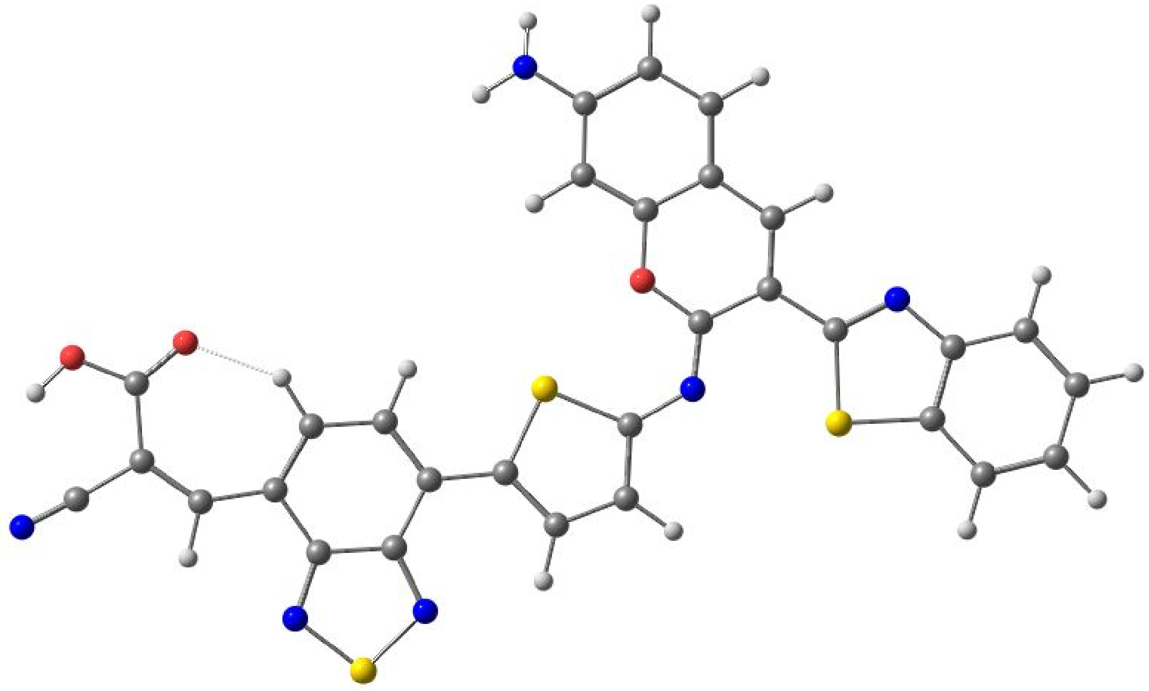

6	-4.730682000	2.218733000	0.008130000
6	-4.347026000	0.908866000	-0.002124000
6	-2.931964000	0.600207000	0.006174000
8	-2.046724000	1.635763000	0.027882000
6	-2.423864000	2.950933000	0.041260000
6	-1.419245000	3.896953000	0.068434000
6	-1.770679000	5.254645000	0.084211000
6	-3.141433000	5.615610000	0.070472000
6	-4.117511000	4.649277000	0.044505000
6	-3.785601000	3.279546000	0.029396000
7	-2.487931000	-0.604240000	-0.006337000
6	2.757021000	-1.112046000	-0.003181000
6	-5.371204000	-0.144943000	-0.020144000
7	-6.637282000	0.147880000	-0.024953000
16	-5.004920000	-1.871756000	-0.035344000
6	-6.717042000	-2.198058000	-0.048880000

6	-7.426196000	-0.985027000	-0.041083000
6	-8.827165000	-0.994323000	-0.049255000
6	-7.380428000	-3.426498000	-0.064776000
6	-9.485060000	-2.212215000	-0.064969000
6	-8.767277000	-3.420503000	-0.072695000
6	3.324753000	0.150532000	-0.067119000
6	4.711179000	0.394222000	-0.076607000
6	5.657588000	-0.617476000	-0.025694000
6	5.121342000	-1.956564000	0.043785000
6	3.700684000	-2.204119000	0.057059000
7	3.405026000	-3.499707000	0.131009000
7	5.841032000	-3.072271000	0.105561000
16	4.802733000	-4.322159000	0.175724000
6	7.090699000	-0.507107000	-0.041647000
6	8.013676000	0.505278000	-0.074629000
6	9.385738000	0.088830000	-0.081819000
7	10.511055000	-0.191844000	-0.089034000
6	7.799546000	1.974070000	-0.096097000
8	6.714325000	2.515621000	-0.104657000
8	8.898247000	2.748933000	-0.104336000
1	-5.790215000	2.450177000	0.001144000
1	-0.381052000	3.585341000	0.077002000
1	-3.411649000	6.666206000	0.080656000
1	-5.164259000	4.936123000	0.036799000
1	-9.371995000	-0.056227000	-0.043095000
1	-6.829276000	-4.360886000	-0.070722000
1	-10.569783000	-2.236800000	-0.071352000
1	-9.305046000	-4.362761000	-0.084995000
1	2.678070000	1.019038000	-0.118588000
1	5.050835000	1.417762000	-0.129875000
1	7.554040000	-1.490343000	-0.019867000
1	9.729553000	2.248549000	-0.095142000
6	-0.786862000	-2.313295000	-0.013221000
6	-1.175850000	-0.988664000	-0.004004000
16	0.235092000	0.057404000	0.010118000
6	1.323316000	-1.313322000	0.001736000
6	0.607776000	-2.494996000	-0.009192000
1	-1.510460000	-3.118312000	-0.023544000
1	1.086128000	-3.463334000	-0.016431000
7	-0.807269000	6.222191000	0.075994000
1	-1.065808000	7.171742000	0.296547000
1	0.145871000	5.970047000	0.288530000

Eelec = -2900.7878262

$H_{(298.15\ K)}$ = -2900.344588
$G_{(298.15\ K)}$ = -2900.451195
ZPE (Zero-point energy correction) = 0.408197

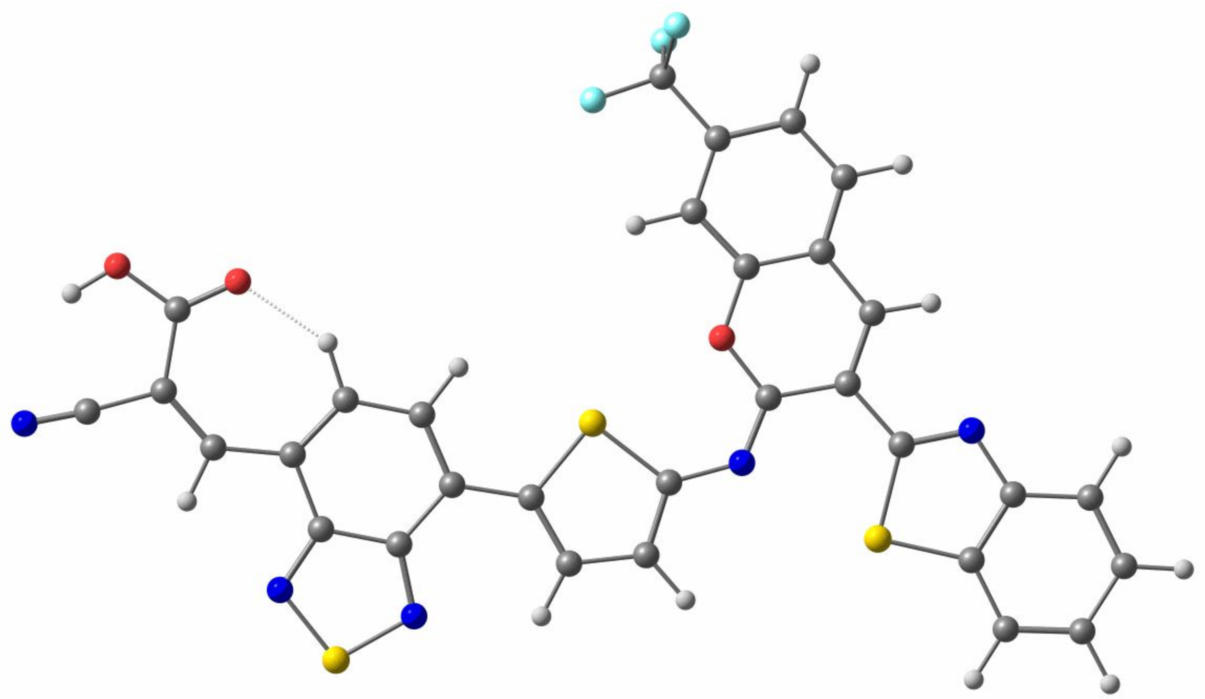

C4, benzothiadiazole- and thiophene-containing dye with a CF3 at the 7-coumarin position at the ground state employing the IEFPCM model and acetonitrile solvent

6	-4.744851000	1.656557000	-0.008704000
6	-4.326248000	0.368114000	-0.014278000
6	-2.895321000	0.087525000	-0.009371000
8	-2.034483000	1.144312000	0.004532000
6	-2.450557000	2.445564000	0.011506000
6	-1.470349000	3.427800000	0.024994000
6	-1.878601000	4.752509000	0.029707000
6	-3.235708000	5.097340000	0.021940000
6	-4.192083000	4.100505000	0.009334000
6	-3.811233000	2.750084000	0.004137000
7	-2.436557000	-1.104429000	-0.017710000
6	2.815344000	-1.553271000	-0.013631000
6	-5.323585000	-0.718508000	-0.024203000
7	-6.593634000	-0.450039000	-0.027622000
16	-4.919515000	-2.432901000	-0.029800000
6	-6.622543000	-2.795431000	-0.036793000
6	-7.358594000	-1.598352000	-0.034747000
6	-8.759541000	-1.636002000	-0.039362000
6	-7.259810000	-4.038539000	-0.043357000
6	-9.390205000	-2.867158000	-0.045777000

6	-8.645620000	-4.060217000	-0.047746000
6	3.367036000	-0.286614000	-0.088004000
6	4.752599000	-0.026446000	-0.094470000
6	5.707713000	-1.026804000	-0.030846000
6	5.186566000	-2.371017000	0.048351000
6	3.769050000	-2.634332000	0.059379000
7	3.487705000	-3.932767000	0.143646000
7	5.918793000	-3.477956000	0.122026000
16	4.894635000	-4.738328000	0.199470000
6	7.142092000	-0.901998000	-0.043509000
6	8.052780000	0.119373000	-0.076550000
6	9.430027000	-0.281567000	-0.079140000
7	10.558407000	-0.548924000	-0.082648000
6	7.822330000	1.587100000	-0.100841000
8	6.730996000	2.115158000	-0.114373000
8	8.912088000	2.373425000	-0.105635000
1	-5.808183000	1.866223000	-0.013083000
1	-0.424047000	3.149999000	0.029331000
1	-3.534668000	6.139189000	0.023371000
1	-5.246731000	4.353391000	0.002064000
1	-9.323409000	-0.709344000	-0.037692000
1	-6.689532000	-4.961242000	-0.044802000
1	-10.474007000	-2.916030000	-0.049259000
1	-9.163770000	-5.013428000	-0.052704000
1	2.711367000	0.574458000	-0.150567000
1	5.080259000	1.000470000	-0.155795000
1	7.614976000	-1.880536000	-0.017523000
1	9.749082000	1.882679000	-0.092455000
6	-0.717336000	-2.794549000	-0.027179000
6	-1.116996000	-1.475886000	-0.016854000
16	0.279467000	-0.413745000	-0.001193000
6	1.380304000	-1.771469000	-0.010153000
6	0.680978000	-2.961194000	-0.022737000
1	-1.432508000	-3.607065000	-0.038299000
1	1.170672000	-3.923696000	-0.030548000
6	-0.857076000	5.852164000	0.071732000
9	-0.857065000	6.490467000	1.265226000
9	0.396662000	5.407530000	-0.130696000
9	-1.097160000	6.798498000	-0.861715000

Eelec = -3182.4212067
$H_{(298.15\ K)}$ = -3181.987710
$G_{(298.15\ K)}$ = -3182.100645
ZPE (Zero-point energy correction) = 0.396375

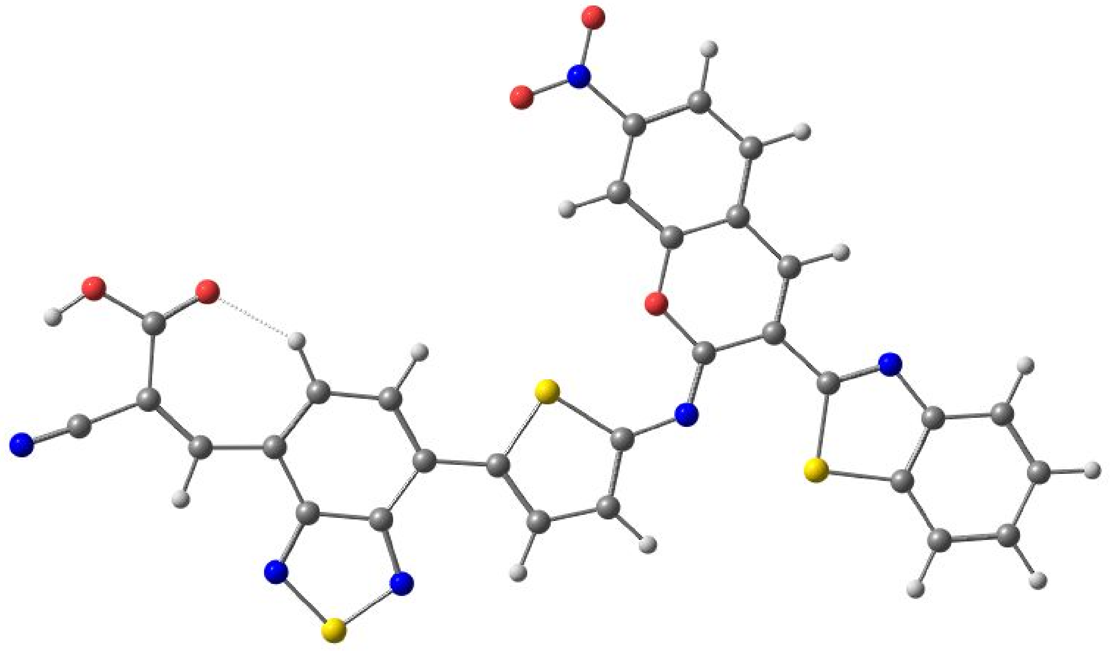

C5, benzothiadiazole- and thiophene-containing dye with a NO2 at the 7-coumarin position at the ground state employing the IEFPCM model and acetonitrile as solvent

6	-4.756781000	1.871956000	0.009524000
6	-4.339122000	0.583805000	-0.003612000
6	-2.907813000	0.299631000	-0.000757000
8	-2.043656000	1.355074000	0.019071000
6	-2.457640000	2.655747000	0.031819000
6	-1.478574000	3.634211000	0.048409000
6	-1.906940000	4.951483000	0.060283000
6	-3.252050000	5.315390000	0.056831000
6	-4.203588000	4.312785000	0.040295000
6	-3.820678000	2.963865000	0.027261000
7	-2.453365000	-0.892345000	-0.016270000
6	2.796108000	-1.368194000	-0.014972000
6	-5.337766000	-0.501722000	-0.019908000
7	-6.607004000	-0.229183000	-0.019178000
16	-4.938347000	-2.216392000	-0.040189000
6	-6.641936000	-2.573901000	-0.047931000
6	-7.374977000	-1.374856000	-0.034723000
6	-8.776230000	-1.408409000	-0.037626000
6	-7.282665000	-3.815285000	-0.064073000
6	-9.409956000	-2.637593000	-0.053545000
6	-8.668337000	-3.832712000	-0.066689000
6	3.354387000	-0.105151000	-0.092450000
6	4.741846000	0.147440000	-0.098580000

6	5.690682000	-0.858126000	-0.031659000
6	5.162027000	-2.199124000	0.050895000
6	3.743164000	-2.454454000	0.061824000
7	3.454448000	-3.751069000	0.150042000
7	5.888087000	-3.309927000	0.128211000
16	4.856882000	-4.564162000	0.208948000
6	7.126369000	-0.742006000	-0.044497000
6	8.042831000	0.273646000	-0.078213000
6	9.417773000	-0.135507000	-0.080744000
7	10.544453000	-0.409729000	-0.084200000
6	7.821010000	1.743025000	-0.102716000
8	6.732737000	2.277084000	-0.117246000
8	8.915248000	2.522810000	-0.106402000
1	-5.819489000	2.083794000	0.006882000
1	-0.428027000	3.377675000	0.051305000
1	-3.532934000	6.359613000	0.066524000
1	-5.258200000	4.564439000	0.036973000
1	-9.337414000	-0.480213000	-0.027328000
1	-6.715022000	-4.739515000	-0.074199000
1	-10.493848000	-2.683796000	-0.055918000
1	-9.189231000	-4.784344000	-0.079047000
1	2.703622000	0.759373000	-0.158603000
1	5.075223000	1.172320000	-0.162687000
1	7.593197000	-1.723397000	-0.017667000
1	9.749513000	2.027435000	-0.092640000
6	-0.743308000	-2.590971000	-0.036430000
6	-1.135255000	-1.270500000	-0.017749000
16	0.266126000	-0.215609000	0.005216000
6	1.359108000	-1.578782000	-0.012174000
6	0.654323000	-2.764950000	-0.032571000
1	-1.462837000	-3.399534000	-0.053031000
1	1.139266000	-3.729745000	-0.046431000
7	-0.890795000	6.006536000	0.076320000
8	0.287589000	5.673140000	0.075824000
8	-1.270075000	7.170653000	0.089335000

Eelec = -3049.8840676
$H_{(298.15\ K)}$ = -3049.453586
$G_{(298.15\ K)}$ = -3049.564084
ZPE (Zero-point energy correction) = 0.394424

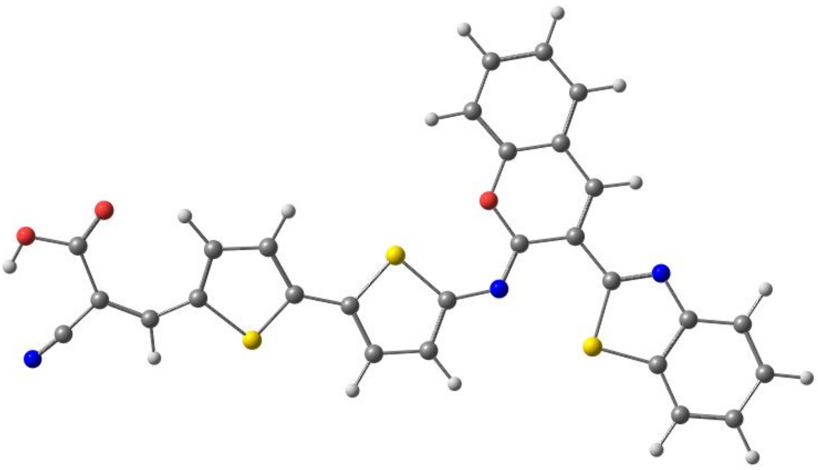

D1, oligothyophene-containing dye with a H at the 7-coumarin position at the ground state employing the IEFPCM model and acetonitrile as solvent

6	2.210112000	3.085014000	-0.038362000
6	1.288842000	4.122594000	-0.049174000
6	1.765451000	5.426187000	-0.068126000
6	3.141049000	5.687170000	-0.076036000
6	4.044320000	4.638711000	-0.065128000
6	3.587661000	3.311234000	-0.046197000
6	4.455753000	2.168055000	-0.034159000
6	3.968223000	0.902415000	-0.015933000
7	1.999006000	-0.462581000	0.009287000
6	2.525460000	0.703100000	-0.007866000
8	1.725036000	1.803738000	-0.019657000
6	0.660094000	-0.753346000	0.019446000
6	-1.844665000	-0.892645000	0.038434000
6	-1.227177000	-2.123069000	0.042727000
6	0.179516000	-2.045180000	0.032332000
6	-5.769235000	-0.717075000	-0.001369000
6	-5.272787000	0.571995000	0.156860000
6	-3.875187000	0.631876000	0.184797000
6	-3.260099000	-0.604881000	0.049241000
16	-4.434240000	-1.854443000	-0.121275000
6	-7.072697000	-1.277135000	-0.083766000
6	-8.350560000	-0.770322000	-0.045408000
6	4.904109000	-0.236421000	-0.004450000
16	4.402567000	-1.926098000	0.031402000
6	6.082587000	-2.386135000	0.027799000
6	6.885619000	-1.233437000	-0.001050000
7	6.187885000	-0.042699000	-0.018484000
6	6.647710000	-3.663349000	0.048043000

6	8.030188000	-3.765325000	0.039046000
6	8.841417000	-2.617293000	0.010098000
6	8.281831000	-1.351971000	-0.010060000
6	-8.759478000	0.640795000	0.109333000
8	-7.987871000	1.569947000	0.229395000
8	-10.080550000	0.900809000	0.116197000
6	-9.410496000	-1.722270000	-0.171344000
7	-10.307011000	-2.452401000	-0.269404000
1	0.227749000	3.902597000	-0.042819000
1	3.497375000	6.710997000	-0.090746000
1	5.113052000	4.826984000	-0.071002000
1	5.529342000	2.318162000	-0.039857000
1	-1.770461000	-3.060694000	0.060757000
1	0.843324000	-2.900053000	0.036637000
1	-5.922580000	1.427550000	0.252094000
1	-3.322086000	1.554612000	0.309399000
1	-7.059402000	-2.358004000	-0.207331000
1	6.025315000	-4.551472000	0.070248000
1	8.492615000	-4.746679000	0.054545000
1	9.920687000	-2.727874000	0.003575000
1	8.898011000	-0.459468000	-0.032465000
1	-10.632313000	0.109030000	0.017570000
16	-0.671927000	0.395337000	0.018661000
1	1.059559000	6.249648000	-0.076854000

Eelec = -2659.809614

$H_{(298.15\ K)}$ = -2659.410456

$G_{(298.15\ K)}$ = -2659.508572

ZPE (Zero-point energy correction) = 0.367927

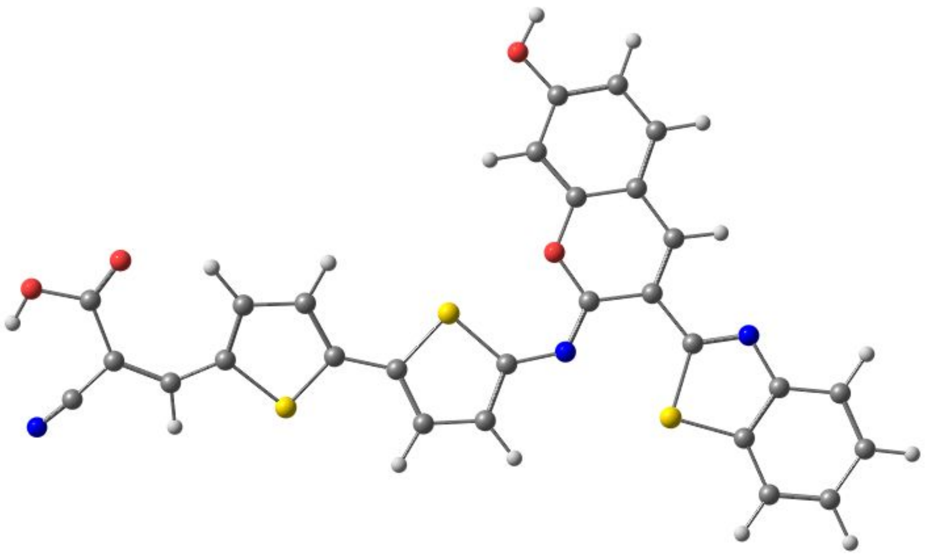

D2, oligothiophene-containing dye with an OH at the 7-coumarin position at the ground state employing the IEFPCM model with acetonitrile as solvent

6	2.198724000	2.883002000	-0.029271000
6	1.277803000	3.914930000	-0.037806000
6	1.759206000	5.220411000	-0.053836000
6	3.138662000	5.482304000	-0.061023000
6	4.031982000	4.430612000	-0.052277000
6	3.580472000	3.100865000	-0.036287000
6	4.440410000	1.959231000	-0.026559000
6	3.948822000	0.691939000	-0.011162000
7	1.971188000	-0.662024000	0.009531000
6	2.508176000	0.499472000	-0.004295000
8	1.710527000	1.605923000	-0.013549000
6	0.630794000	-0.943418000	0.018232000
6	-1.875700000	-1.066395000	0.035670000
6	-1.265941000	-2.300749000	0.036793000
6	0.141108000	-2.232170000	0.027260000
6	-5.799080000	-0.865210000	-0.002129000
6	-5.293718000	0.420888000	0.153635000
6	-3.895942000	0.471618000	0.180525000
6	-3.288855000	-0.769541000	0.046635000
16	-4.471460000	-2.011619000	-0.121114000
6	-7.106048000	-1.416426000	-0.082963000
6	-8.380741000	-0.901106000	-0.043402000
6	4.880871000	-0.447711000	-0.001665000
16	4.371939000	-2.136444000	0.024591000
6	6.050426000	-2.604072000	0.022766000
6	6.858293000	-1.454607000	0.002072000

7	6.166043000	-0.260330000	-0.011179000
6	6.609717000	-3.883661000	0.038035000
6	7.991976000	-3.992316000	0.032286000
6	8.808004000	-2.847866000	0.011497000
6	8.253798000	-1.579780000	-0.003664000
6	-8.780017000	0.512498000	0.111736000
8	-8.002165000	1.436770000	0.229721000
8	-10.099413000	0.781314000	0.121524000
6	-9.446954000	-1.846051000	-0.168082000
7	-10.348302000	-2.570404000	-0.265111000
1	0.213503000	3.714872000	-0.032265000
1	3.494091000	6.507500000	-0.073368000
1	5.099412000	4.624866000	-0.057662000
1	5.514834000	2.104410000	-0.031621000
1	-1.815280000	-3.234931000	0.051731000
1	0.799181000	-3.091468000	0.029448000
1	-5.937781000	1.280893000	0.247803000
1	-3.336685000	1.390928000	0.302936000
1	-7.100189000	-2.497426000	-0.206076000
1	5.983152000	-4.769016000	0.054015000
1	8.449808000	-4.975881000	0.043985000
1	9.886853000	-2.962884000	0.007366000
1	8.874249000	-0.690066000	-0.019746000
1	-10.656499000	-0.006959000	0.024887000
16	-0.694087000	0.213975000	0.020080000
8	0.838976000	6.213328000	-0.061968000
1	1.265720000	7.081057000	-0.073192000

Eelec = -2735.02110

$H_{(298.15\ K)}$ = -2734.616712

$G_{(298.15\ K)}$ = -2734.717444

ZPE (Zero-point energy correction) = 0.371926

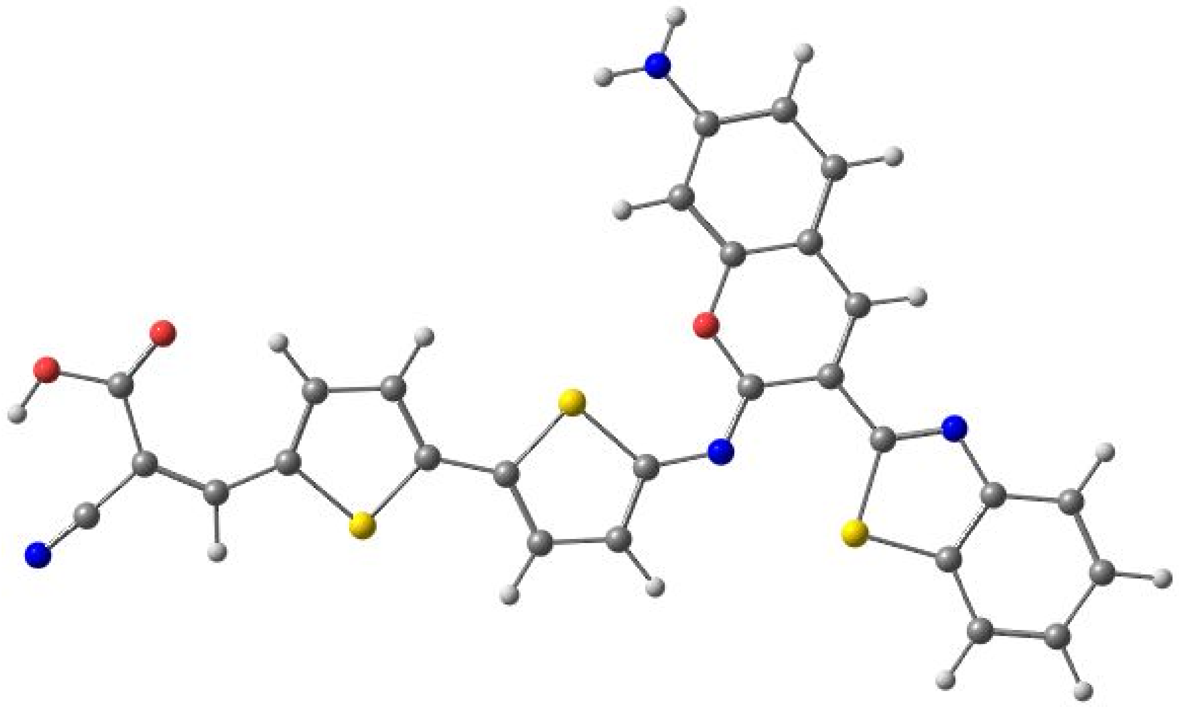

D3, oligothiophene-containing dye with a NH2 at the 7-coumarin position at the ground state employing the IEFPCM model with acetonitrile as solvent

6	2.188435000	2.877871000	-0.026117000
6	1.260395000	3.899566000	-0.040921000
6	1.716323000	5.225870000	-0.055552000
6	3.110766000	5.478920000	-0.054018000
6	4.008880000	4.439136000	-0.040305000
6	3.571319000	3.099819000	-0.026090000
6	4.432257000	1.968618000	-0.015890000
6	3.948949000	0.692868000	-0.005786000
7	1.977480000	-0.671153000	0.007050000
6	2.513286000	0.493895000	-0.003629000
8	1.709842000	1.595791000	-0.013570000
6	0.638570000	-0.952742000	0.012689000
6	-1.870169000	-1.076816000	0.028239000
6	-1.258986000	-2.310824000	0.024276000
6	0.147551000	-2.242049000	0.015992000
6	-5.793778000	-0.876070000	-0.003290000
6	-5.287108000	0.410700000	0.146729000
6	-3.889866000	0.461322000	0.170650000
6	-3.282363000	-0.780828000	0.040309000

16	-4.465943000	-2.023375000	-0.120343000
6	-7.100475000	-1.426266000	-0.079504000
6	-8.375469000	-0.910045000	-0.038191000
6	4.888271000	-0.437407000	0.002218000
16	4.388389000	-2.130542000	0.020697000
6	6.069873000	-2.589660000	0.020936000
6	6.871448000	-1.435653000	0.006749000
7	6.173446000	-0.244535000	-0.003464000
6	6.635651000	-3.866150000	0.032200000
6	8.018750000	-3.968393000	0.029083000
6	8.828482000	-2.819760000	0.014839000
6	8.267427000	-1.554261000	0.003646000
6	-8.773446000	0.503624000	0.114282000
8	-7.995088000	1.428405000	0.226688000
8	-10.092926000	0.773146000	0.128341000
6	-9.442233000	-1.854694000	-0.157724000
7	-10.344153000	-2.578985000	-0.250780000
1	0.201166000	3.669341000	-0.040962000
1	3.461600000	6.505391000	-0.063455000
1	5.075025000	4.642519000	-0.041716000
1	5.506282000	2.118601000	-0.017047000
1	-1.807861000	-3.245433000	0.034556000
1	0.805585000	-3.101377000	0.015110000
1	-5.930959000	1.271131000	0.238756000
1	-3.330139000	1.380953000	0.288251000
1	-7.095753000	-2.507635000	-0.199541000
1	6.013431000	-4.754723000	0.043090000
1	8.481410000	-4.949747000	0.037706000
1	9.908002000	-2.928826000	0.012608000
1	8.883624000	-0.661464000	-0.007424000
1	-10.650456000	-0.015349000	0.036241000
16	-0.687888000	0.204198000	0.018665000
7	0.831924000	6.266797000	-0.034148000
1	-0.139736000	6.092195000	-0.240800000
1	1.163893000	7.192781000	-0.256861000

$Eelec$ = -2715.1587989

$H_{(298.15\ K)}$ = -2714.741870

$G_{(298.15\ K)}$ = -2714.843324

ZPE (Zero-point energy correction) = 0.384012

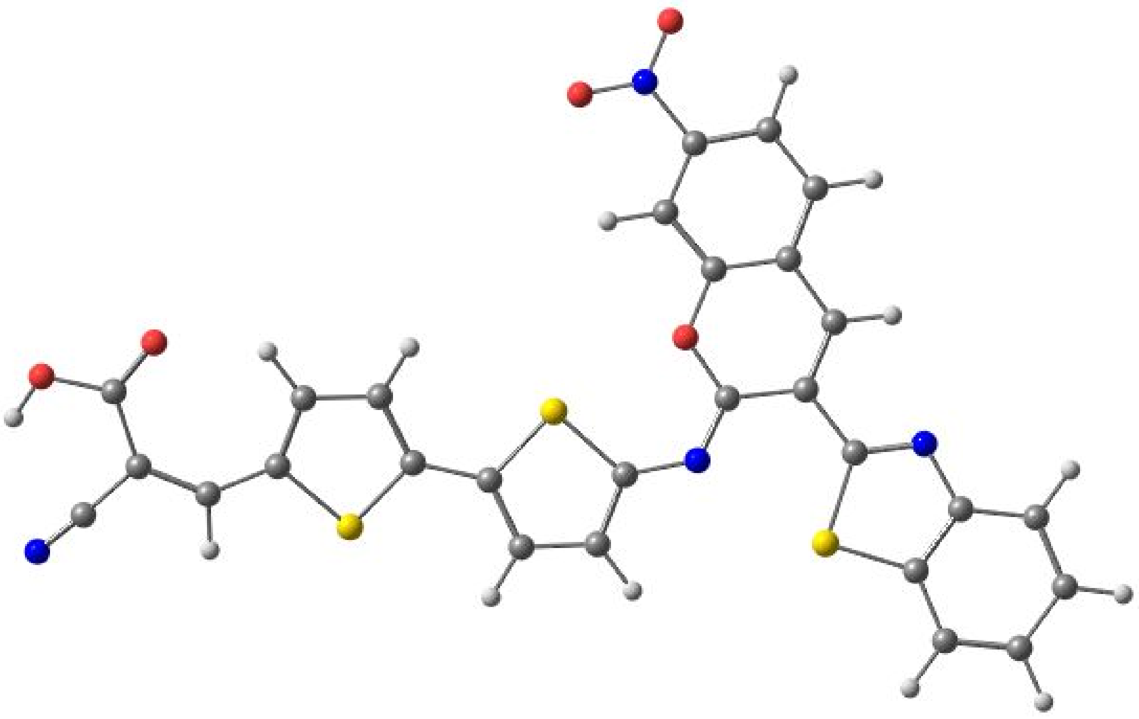

D5, oligothiophene-containing dye with a NO2 at the 7-coumarin position at the ground state employing the IEFPCM model with acetonitrile as solvent

6	2.223542000	2.550126000	-0.037321000
6	1.327107000	3.604657000	-0.051167000
6	1.860702000	4.883064000	-0.068750000
6	3.230747000	5.137005000	-0.072011000
6	4.097907000	4.060421000	-0.057507000
6	3.606953000	2.747229000	-0.040452000
6	4.452786000	1.583731000	-0.025486000
6	3.933068000	0.333467000	-0.011387000
7	1.934280000	-0.985637000	-0.003658000
6	2.483391000	0.164973000	-0.011600000
8	1.705742000	1.287118000	-0.020883000
6	0.589922000	-1.256500000	0.000615000
6	-1.913054000	-1.358913000	0.024364000
6	-1.315536000	-2.598580000	0.001114000
6	0.092648000	-2.540871000	-0.010996000
6	-5.833728000	-1.123196000	-0.018546000
6	-5.322015000	0.139542000	0.254938000

6	-3.922888000	0.177777000	0.291320000
6	-3.325592000	-1.050201000	0.049837000
16	-4.514652000	-2.262557000	-0.238750000
6	-7.145480000	-1.656053000	-0.158163000
6	-8.415836000	-1.140665000	-0.063781000
6	4.840697000	-0.829309000	0.005239000
16	4.303563000	-2.506190000	0.037340000
6	5.972653000	-3.000351000	0.046996000
6	6.800401000	-1.864795000	0.023793000
7	6.127862000	-0.660750000	0.000806000
6	6.510854000	-4.289362000	0.072471000
6	7.890530000	-4.419134000	0.074473000
6	8.726479000	-3.288176000	0.051182000
6	8.194324000	-2.011856000	0.025794000
6	-8.807599000	0.249088000	0.253211000
8	-8.024031000	1.148508000	0.475417000
8	-10.125089000	0.521492000	0.297124000
6	-9.487363000	-2.060603000	-0.292268000
7	-10.391265000	-2.766180000	-0.469017000
1	0.259232000	3.434166000	-0.049043000
1	3.595154000	6.155038000	-0.085754000
1	5.169525000	4.224704000	-0.059408000
1	5.528867000	1.710768000	-0.025447000
1	-1.873375000	-3.527621000	0.005737000
1	0.744392000	-3.404910000	-0.023462000
1	-5.960694000	0.991532000	0.426128000
1	-3.357831000	1.077260000	0.502488000
1	-7.145291000	-2.717701000	-0.395895000
1	5.870112000	-5.164282000	0.090534000
1	8.332531000	-5.409734000	0.094376000
1	9.803063000	-3.421929000	0.053330000
1	8.828797000	-1.132247000	0.007804000
1	-10.687058000	-0.246453000	0.108258000
16	-0.723531000	-0.088423000	0.024717000
7	0.933112000	6.016539000	-0.085306000
8	-0.268376000	5.779498000	-0.078566000
8	1.404860000	7.146288000	-0.105317000

$Eelec$ = -2864.2552633

$H_{(298.15\ K)}$ = -2863.850965

$G_{(298.15\ K)}$ = -2863.956547

ZPE (Zero-point energy correction) = 0.37039

Alternative (*E*)-stereoisomers of the A3, C3 and D3 dyes

(*E*)-A3, benzothiadiazole-containing dye with an NH2 substituent at the 7-coumarin position at the ground state

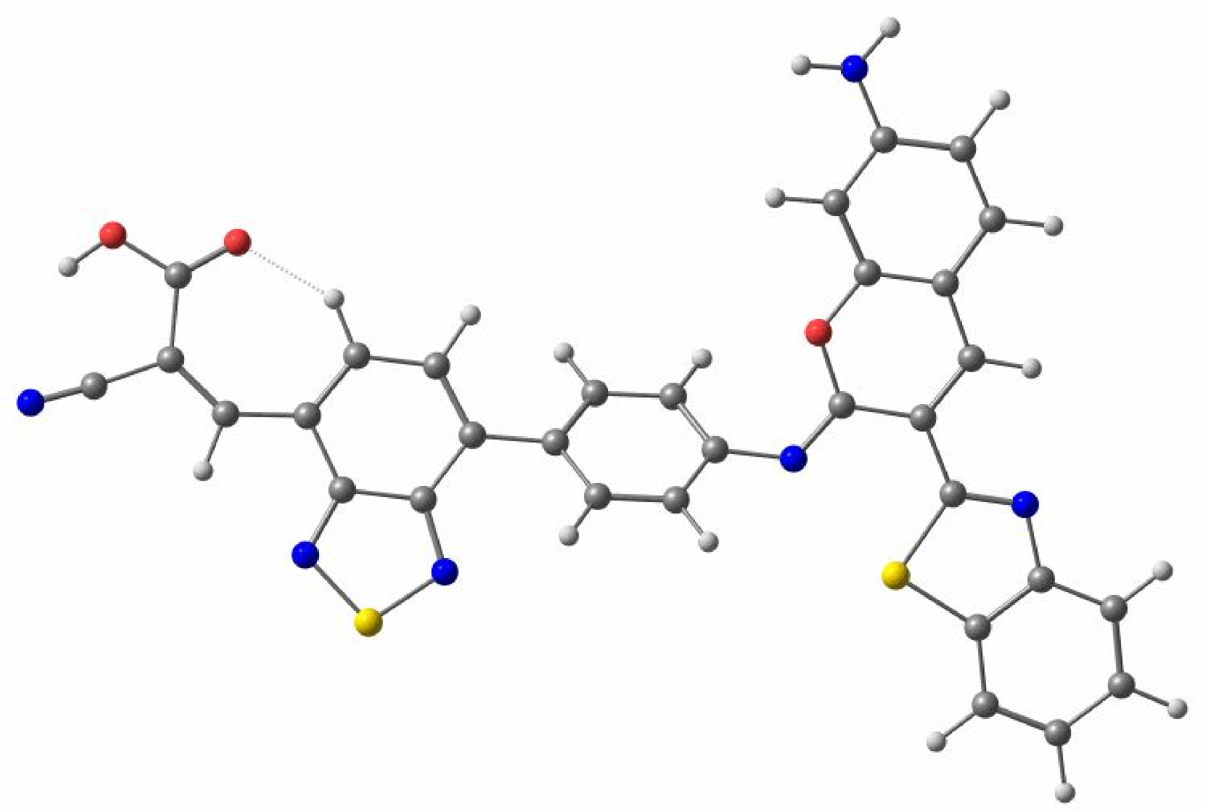

6	5.492280000	1.803677000	0.044679000
6	4.753039000	0.666300000	0.003756000
6	3.297514000	0.763121000	0.050425000
8	2.744233000	2.017145000	0.132176000
6	3.487497000	3.158961000	0.184733000
6	2.798364000	4.354647000	0.283839000
6	3.518097000	5.552127000	0.338399000
6	4.928107000	5.513105000	0.289741000
6	5.588258000	4.308030000	0.193154000
6	4.883978000	3.094185000	0.138603000
7	2.548730000	-0.263700000	0.037896000
6	1.151170000	-0.233278000	0.025131000
6	0.407025000	0.531974000	-0.882308000
6	-0.975871000	0.455871000	-0.892252000
6	-1.668668000	-0.378191000	-0.003037000
6	-0.916333000	-1.158472000	0.884854000
6	0.467670000	-1.089817000	0.894231000
6	-3.142334000	-0.397603000	-0.005035000

6	5.430253000	-0.636291000	-0.089389000
7	6.725729000	-0.709915000	-0.124426000
16	4.584473000	-2.181862000	-0.166859000
6	6.132645000	-2.975277000	-0.254694000
6	7.158441000	-2.015272000	-0.216953000
6	8.498088000	-2.420105000	-0.271737000
6	6.421529000	-4.338249000	-0.346328000
6	8.782642000	-3.770357000	-0.362521000
6	7.751219000	-4.723965000	-0.399741000
6	-3.880121000	0.736331000	-0.266346000
6	-5.296193000	0.765712000	-0.292404000
6	-6.071903000	-0.348284000	-0.048519000
6	-5.350213000	-1.560836000	0.229968000
6	-3.908384000	-1.587479000	0.254998000
7	-3.420118000	-2.800643000	0.512797000
7	-5.901445000	-2.745649000	0.474091000
16	-4.684459000	-3.797232000	0.709835000
6	-7.517929000	-0.407793000	-0.044740000
6	-8.444805000	0.554035000	-0.265456000
6	-8.151367000	1.916908000	-0.569021000
7	-8.002368000	3.039641000	-0.819381000
6	-9.896681000	0.169803000	-0.188324000
8	-10.258813000	-0.950020000	0.063085000
8	-10.793703000	1.145567000	-0.415636000
1	6.573314000	1.717908000	0.006453000
1	1.714400000	4.343146000	0.316370000
1	5.488711000	6.441695000	0.326232000
1	6.673212000	4.286318000	0.159507000
1	0.918440000	1.171642000	-1.592231000
1	-1.527183000	1.035292000	-1.625905000
1	-1.418794000	-1.827007000	1.571959000
1	1.044051000	-1.701945000	1.579191000
1	9.283106000	-1.672416000	-0.241944000
1	5.627804000	-5.077443000	-0.375467000
1	9.815274000	-4.100868000	-0.405804000
1	7.999091000	-5.777948000	-0.471517000
1	-3.357958000	1.670404000	-0.439224000
1	-5.765783000	1.717961000	-0.504224000
1	-7.940489000	-1.386177000	0.171620000
1	-10.377675000	1.999768000	-0.607219000
7	2.860806000	6.760629000	0.398151000
1	3.384804000	7.574388000	0.678206000
1	1.890306000	6.765579000	0.669243000

Eelec = -2579.969726
$H_{(298.15\ K)}$ = -2579.486282
$G_{(298.15\ K)}$ = -2579.535081
ZPE (Zero-point energy correction) = 0.441068

(*E*)-C3 ground-state optimized geometry

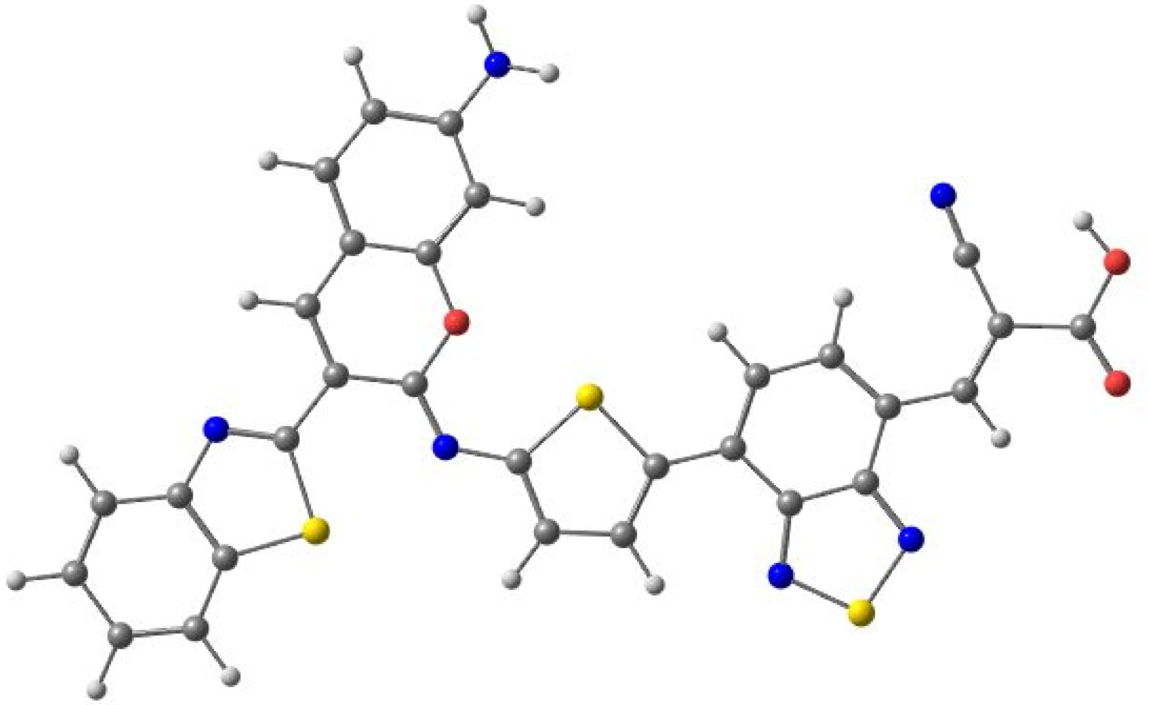

6	-4.789342000	2.188174000	-0.002736000
6	-4.380873000	0.891055000	-0.002053000
6	-2.957563000	0.604156000	-0.004484000
8	-2.088152000	1.660930000	-0.007155000
6	-2.493317000	2.964986000	-0.007884000
6	-1.506774000	3.933593000	-0.008783000
6	-1.880401000	5.281517000	-0.008718000
6	-3.252585000	5.617482000	-0.007212000
6	-4.212253000	4.630857000	-0.005468000
6	-3.858428000	3.270509000	-0.005724000
7	-2.497828000	-0.588527000	-0.003827000
6	2.749468000	-1.059985000	-0.006388000
6	-5.387757000	-0.182530000	0.001540000
7	-6.654328000	0.099180000	0.004415000
16	-4.999212000	-1.902243000	0.002250000
6	-6.706329000	-2.245534000	0.006713000

6	-7.429642000	-1.040632000	0.007355000
6	-8.829880000	-1.065008000	0.010787000
6	-7.358172000	-3.480140000	0.009463000
6	-9.474366000	-2.288467000	0.013498000
6	-8.743649000	-3.488653000	0.012843000
6	3.310406000	0.206231000	-0.007288000
6	4.698031000	0.455087000	-0.006017000
6	5.644949000	-0.551540000	-0.004135000
6	5.120179000	-1.890464000	-0.004553000
6	3.699518000	-2.147901000	-0.005364000
7	3.414508000	-3.447956000	-0.005205000
7	5.852368000	-2.999380000	-0.003778000
16	4.822834000	-4.256041000	-0.004269000
6	7.077970000	-0.384174000	-0.003115000
6	7.841825000	0.736441000	0.007570000
6	7.333968000	2.068798000	0.023404000
7	7.003211000	3.180821000	0.037058000
6	9.334871000	0.575073000	0.004493000
8	9.872956000	-0.501818000	-0.007716000
8	10.066275000	1.704721000	0.016278000
1	-5.854942000	2.392019000	-0.000867000
1	-0.464576000	3.635004000	-0.014297000
1	-3.544424000	6.662757000	-0.012331000
1	-5.264316000	4.898585000	-0.003650000
1	-9.379952000	-0.130378000	0.011182000
1	-6.797765000	-4.409169000	0.008924000
1	-10.558781000	-2.325337000	0.016114000
1	-9.271672000	-4.436585000	0.014981000
1	2.656571000	1.070882000	-0.009258000
1	5.010741000	1.491519000	-0.007229000
1	7.652598000	-1.307385000	-0.011488000
1	9.518488000	2.504188000	0.025165000
6	-0.785720000	-2.283409000	-0.004684000
6	-1.183119000	-0.963672000	-0.005041000
16	0.219824000	0.092406000	-0.006611000
6	1.315742000	-1.269733000	-0.006306000
6	0.610355000	-2.456232000	-0.005421000
1	-1.507874000	-3.089268000	-0.003853000
1	1.098743000	-3.419580000	-0.005260000
7	-0.927762000	6.270343000	-0.047651000
1	-1.199784000	7.214552000	0.173457000
1	0.029333000	6.034897000	0.160390000

Eelec = -2900.7673634

$H_{(298.15\ K)}$ = -2900.323461
$G_{(298.15\ K)}$ = -2900.430929
ZPE (Zero-point energy correction) = 0.408810

(*E*)-D3 ground-state optimized geometry

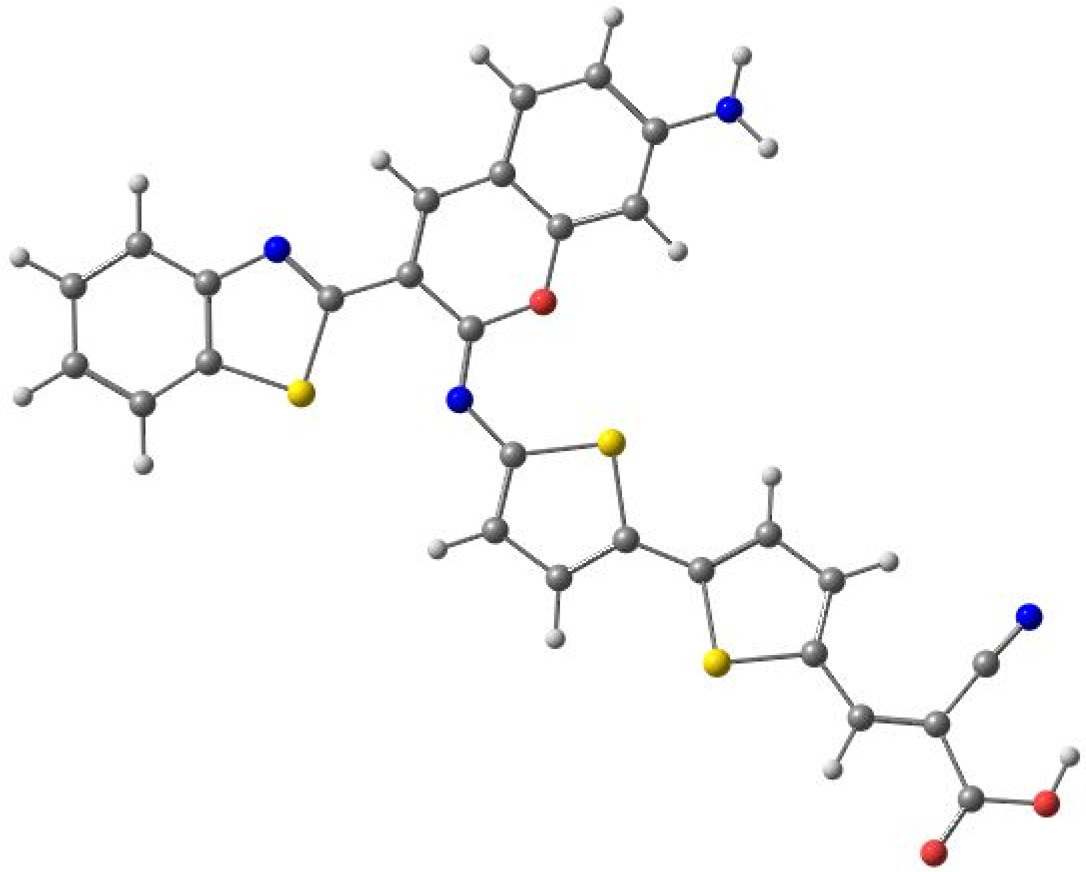

6	2.255684000	2.887008000	-0.062331000
6	1.350852000	3.932175000	-0.086868000
6	1.832196000	5.244916000	-0.121899000
6	3.226381000	5.469152000	-0.130704000
6	4.103284000	4.408033000	-0.107295000
6	3.640745000	3.081547000	-0.072874000
6	4.482373000	1.927607000	-0.050896000
6	3.971255000	0.668460000	-0.021781000
7	1.974618000	-0.653853000	0.015385000
6	2.528717000	0.496149000	-0.010576000
8	1.745956000	1.620216000	-0.029202000
6	0.632957000	-0.921706000	0.032631000

6	-1.874672000	-1.018007000	0.079147000
6	-1.278679000	-2.257314000	0.064556000
6	0.129576000	-2.203266000	0.039328000
6	-5.788780000	-0.772890000	0.017985000
6	-5.286003000	0.465767000	0.378709000
6	-3.883802000	0.501881000	0.433628000
6	-3.285728000	-0.705403000	0.115789000
16	-4.478803000	-1.893334000	-0.268167000
6	-7.129647000	-1.234710000	-0.142609000
6	-8.301626000	-0.559021000	-0.003237000
6	4.888561000	-0.482446000	-0.001862000
16	4.362850000	-2.165074000	0.039164000
6	6.036703000	-2.644911000	0.041537000
6	6.854592000	-1.502499000	0.011042000
7	6.173687000	-0.304191000	-0.012848000
6	6.587167000	-3.927691000	0.068220000
6	7.967453000	-4.047793000	0.063977000
6	8.792283000	-2.910654000	0.033492000
6	8.248267000	-1.639585000	0.006945000
6	-9.577251000	-1.304756000	-0.235630000
8	-9.603678000	-2.470901000	-0.540789000
8	-10.721059000	-0.611562000	-0.088824000
6	-8.387606000	0.817504000	0.349592000
7	-8.529134000	1.933795000	0.632994000
1	0.287824000	3.719429000	-0.075287000
1	3.601670000	6.487236000	-0.152061000
1	5.173572000	4.589476000	-0.116007000
1	5.560806000	2.045600000	-0.058255000
1	-1.840516000	-3.183872000	0.090225000
1	0.783078000	-3.065606000	0.032522000
1	-5.911707000	1.318147000	0.607903000
1	-3.317526000	1.379656000	0.719011000
1	-7.265247000	-2.278465000	-0.419350000
1	5.953832000	-4.808309000	0.091853000
1	8.417478000	-5.034925000	0.084554000
1	9.870214000	-3.034668000	0.030876000
1	8.871658000	-0.752481000	-0.016686000
1	-10.565866000	0.312661000	0.158394000
16	-0.681863000	0.248725000	0.051970000
7	0.961347000	6.308728000	-0.108977000
1	-0.008767000	6.143459000	-0.324724000
1	1.309666000	7.218938000	-0.363886000

$Eelec$ = -2715.1381905

$H_{(298.15\ K)} = -2714.720439$
$G_{(298.15\ K)} = -2714.822213$
ZPE (Zero-point energy correction) = 0.384870

References

(1) Tiwari, G. N.; R, K. M. *Advanced Renewable Energy Sources*; The Royal Society of Chemistry, 2011. https://doi.org/10.1039/9781849736978.
(2) Zero, D. for E. S. & N. *UK Energy in Brief 2023*; 2023.
(3) (EI), E. I. *72nd Energy Institute (EI) Statistical Review of World Energy*; 2023.
(4) Sayigh, A. Solar and Wind Energy Will Supply More Than 50% of World Electricity by 2030. In *Green Buildings and Renewable Energy: Med Green Forum 2019 - Part of World Renewable Energy Congress and Network*; Springer International Publishing: Cham, 2020; pp 385–399. https://doi.org/10.1007/978-3-030-30841-4_27.
(5) Adebayo, T. S.; Alola, A. A. Drivers of Natural Gas and Renewable Energy Utilization in the USA: How about Household Energy Efficiency-Energy Expenditure and Retail Electricity Prices? *Energy* **2023**, *283*, 129022. https://doi.org/10.1016/j.energy.2023.129022.
(6) Tomasi, J.; Mennucci, B.; Cancès, E. The IEF Version of the PCM Solvation Method: An Overview of a New Method Addressed to Study Molecular Solutes at the QM Ab Initio Level. *Journal of Molecular Structure: THEOCHEM* **1999**, *464* (1–3), 211–226. https://doi.org/10.1016/S0166-1280(98)00553-3.
(7) Hara, K.; Wang, Z. S.; Sato, T.; Furube, A.; Katoh, R.; Sugihara, H.; Dan-Oh, Y.; Kasada, C.; Shinpo, A.; Suga, S. Oligothiophene-Containing Coumarin Dyes for Efficient Dye-Sensitized Solar Cells. *Journal of Physical Chemistry B* **2005**, *109* (32), 15476–15482. https://doi.org/10.1021/jp0518557.
(8) Seo, K. D.; Song, H. M.; Lee, M. J.; Pastore, M.; Anselmi, C.; De Angelis, F.; Nazeeruddin, M. K.; Gräetzel, M.; Kim, H. K. Coumarin Dyes Containing Low-Band-Gap Chromophores for Dye-Sensitised Solar Cells. *Dyes and Pigments* **2011**, *90* (3), 304–310. https://doi.org/10.1016/j.dyepig.2011.01.009.
(9) Song, H. M.; Seo, K. D.; Kang, M. S.; Choi, I. T.; Kim, S. K.; Eom, Y. K.; Ryu, J. H.; Ju, M. J.; Kim, H. K. A Simple Triaryl Amine-Based Dual Functioned Co-Adsorbent for Highly Efficient Dye-Sensitized Solar Cells. *J Mater Chem* **2012**, *22* (9), 3786–3794. https://doi.org/10.1039/c2jm16021h.
(10) Zhang, C.-R.; Liu, L.; Liu, Z.-J.; Shen, Y.-L.; Sun, Y.-T.; Wu, Y.-Z.; Chen, Y.-H.; Yuan, L.-H.; Wang, W.; Chen, H.-S. Electronic Structures and Optical Properties of Organic Dye Sensitizer NKX Derivatives for Solar Cells: A Theoretical Approach. *J Mol Graph Model* **2012**, *38*, 419–429. https://doi.org/10.1016/j.jmgm.2012.09.004.
(11) Peach, M. J. G.; Benfield, P.; Helgaker, T.; Tozer, D. J. Excitation Energies in Density Functional Theory: An Evaluation and a Diagnostic Test. *Journal of Chemical Physics* **2008**, *128* (4). https://doi.org/10.1063/1.2831900.
(12) Donon, J.; Habka, S.; Mons, M.; Brenner, V.; Gloaguen, E. Conformational Analysis by UV Spectroscopy: The Decisive Contribution of Environment-Induced Electronic Stark Effects. *Chem Sci* **2021**, *12* (8), 2803–2815. https://doi.org/10.1039/D0SC06074G.
(13) Xie, X.; Li, X.; Luo, H.; Lei, K.; Li, W. Effect of Conformation on UV-Vis Absorption Spectra of Disazo Reactive Red Dyes. *Wuhan University Journal of Natural Sciences* **2016**, *21* (6), 512–518. https://doi.org/10.1007/s11859-016-1204-3.

(14) Domingo, L. R.; Ríos-Gutiérrez, M.; Pérez, P. Applications of the Conceptual Density Functional Theory Indices to Organic Chemistry Reactivity. *Molecules*. MDPI AG June 1, 2016. https://doi.org/10.3390/molecules21060748.
